# Development of Furanopyrimidine-Based Orally Active Third-Generation EGFR Inhibitors for the Treatment of Non-Small Cell Lung Cancer

**DOI:** 10.1021/acs.jmedchem.2c01434

**Published:** 2023-02-07

**Authors:** Mu-Chun Li, Mohane Selvaraj Coumar, Shu-Yu Lin, Yih-Shyan Lin, Guan-Lin Huang, Chun-Hwa Chen, Tzu-Wen Lien, Yi-Wen Wu, Yen-Ting Chen, Ching-Ping Chen, Yu-Chen Huang, Kai-Chia Yeh, Chen-Ming Yang, Bikashita Kalita, Shiow-Lin Pan, Tsu-An Hsu, Teng-Kuang Yeh, Chiung-Tong Chen, Hsing-Pang Hsieh

**Affiliations:** † Institute of Biotechnology and Pharmaceutical Research, National Health Research Institutes, Miaoli County 350401, Taiwan, ROC; ‡ Biomedical Translation Research Center, Academia Sinica, Taipei City 115202, Taiwan, ROC; § Department of Bioinformatics, School of Life Sciences, Pondicherry University, Kalapet 605014, Pondicherry, India; ∥ Graduate Institute of Cancer Biology and Drug Discovery, College of Medical Science and Technology, Taipei Medical University, Taipei City 110301, Taiwan, ROC; ⊥ Ph.D. Program in Drug Discovery and Development Industry, College of Pharmacy, Taipei Medical University, Taipei City 110301, Taiwan, ROC; # Department of Chemistry, National Tsing Hua University, Hsinchu City 300044, Taiwan, ROC

## Abstract

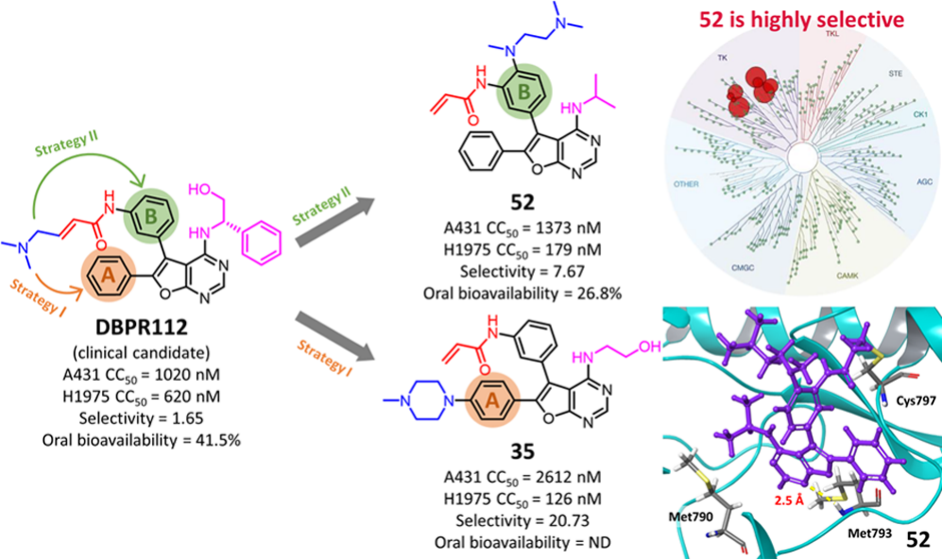

The development of orally bioavailable, furanopyrimidine-based double-mutant (L858R/T790M) EGFR inhibitors is described. First, selectivity for mutant EGFR was accomplished by replacing the (*S*)-2-phenylglycinol moiety of **12** with either an ethanol or an alkyl substituent. Then, the cellular potency and physicochemical properties were optimized through insights from molecular modeling studies by implanting various solubilizing groups in phenyl rings A and B. Optimized lead **52** shows 8-fold selective inhibition of H1975 (EGFR^L858R/T790M^ overexpressing) cancer cells over A431 (EGFR^WT^ overexpressing) cancer cells; western blot analysis further confirmed EGFR mutant-selective target modulation inside the cancer cells by **52**. Notably, **52** displayed *in vivo* antitumor effects in two different mouse xenograft models (BaF3 transfected with mutant EGFR and H1975 tumors) with TGI = 74.9 and 97.5% after oral administration (*F* = 27%), respectively. With an extraordinary kinome selectivity (*S*(10) score of 0.017), **52** undergoes detailed preclinical development.

## Introduction

**Figure 1:**
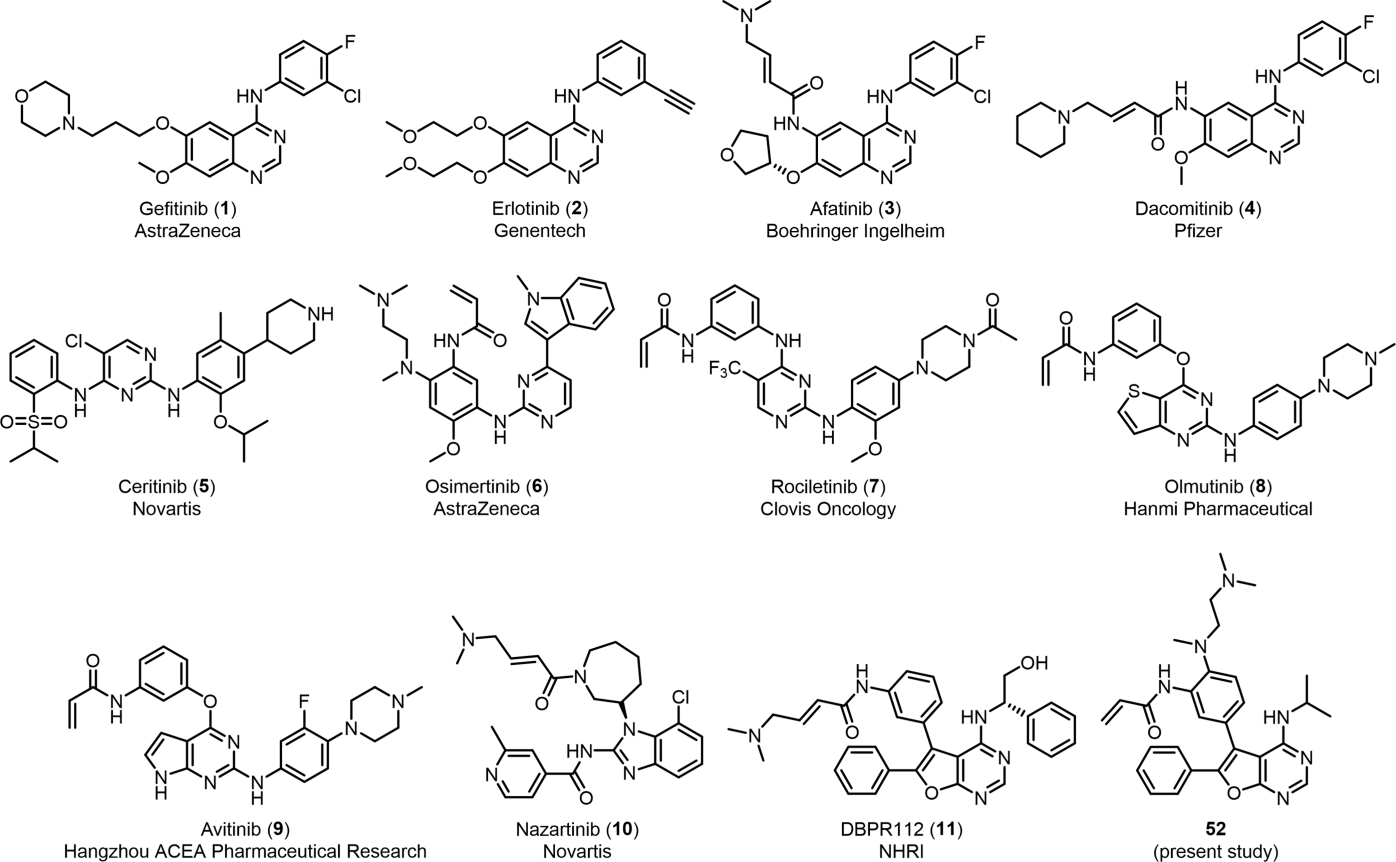
Known EGFR inhibitors approved or in advanced phase of drug development for NSCLC.

Lung cancer is one of the leading causes of cancer deaths worldwide, accounting for a quarter of all cancer deaths.^1,2^ Most lung cancer patients, around 80–85%, have the non-small cell lung cancer (NSCLC) disease, which is characterized by somatic epidermal growth factor receptor (EGFR) activating mutations in 10–15% of Caucasian and 30–50% of Asian patients.^3,4^ Several drugs targeting mutated EGFR have been approved, such as the first-generation EGFR inhibitors gefitinib (**1**) and erlotinib (**2**), which were approved by the US FDA in 2003 and 2004 (Figure 1).^5−10^ Gefitinib and erlotinib effectively target the EGFR exon 19 deletion or exon 21 L858R single point mutation^11,12^ and most patients respond well, but about half of them develop resistance due to a secondary T790M gatekeeper mutation.^13,14^ Several second-generation EGFR inhibitors capable of overcoming resistance due to T790M mutations have also been approved,^15^ including afatinib (**3**),^16^ dacomitinib (**4**),^17^ and ceritinib (**5**). These second-generation inhibitors all bear a Michael acceptor warhead that forms an irreversible covalent bond with the cysteine residue (Cys797) in the active site of EGFR.^18^

However, major drawbacks with the first- and second-generation drugs were their dose-limiting toxicities, a consequence of EGFR wild-type (EGFR^WT^) enzyme inhibition. To address this problem, third-generation EGFR inhibitors capable of overcoming the T790M mutation and sparing the EGFR^WT^ enzyme were developed, including osimertinib (**6**, approved by US FDA),^19−21^ rociletinib (**7**, halted, Phase III),^22,23^ olmutinib (**8**, approved in South Korea),^24^ avitinib (**9**, Phase II),^25^ nazartinib (**10**, Phase II),^26^ etc.^27^

Recently, we disclosed a second-generation furanopyrimidine EGFR inhibitor DBPR112 (**11**) able to overcome the T790M EGFR mutation.^28^ Compound **11** bears a Michael acceptor group that makes a covalent bond with the Cys797 residue of EGFR,^28^ has favorable *in vitro* and *in vivo* anticancer profiles, and is currently undergoing Phase Ib/II clinical trials to treat lung cancer with an exon 20 insertion. Herein, we describe our continuation of the design and development of third-generation mutant-selective EGFR inhibitor **52** with improved *in vitro* and *in vivo* profiles than those of **11**; mainly, compound **52** is EGFR^WT^ sparing and T790M mutant-selective, which would result in lower toxicities during administration to patients. More importantly, inputs from molecular modeling and *in vitro* assay data had guided the development of mutant-selective EGFR inhibitors.

## Results and Discussion

### Preliminary SAR Exploration of 11/12 to Impart EGFR^L858R/T790M^ Selectivity and Cellular Potency

The newly synthesized compounds were initially investigated for their abilities to inhibit the EGFR wild-type and EGFR double-mutant (EGFR^L858R/T790M^) enzymes at 10 and 1 μM concentrations. Compounds with potent inhibition (>90%) at 10 μM were further evaluated in a six-point dose–response assay to determine the IC_50_ values. Furthermore, the compounds were investigated for their antiproliferative activity on two lung cancer cell lines: A431 cells overexpressing EGFR^WT^ and H1975 cells overexpressing double-mutant EGFR^L858R/T790M^. DBPR112 (**11**) was found to inhibit the EGFR^WT^ better than the mutant EGFR kinase (EGFR^WT^ IC_50_ = 15 nM *vs* EGFR^L858R/T790M^ IC_50_ = 48 nM; Table 1). In the cell-based antiproliferative assay, it displayed moderate potency (A431 CC_50_ = 1020 nM and H1975 CC_50_ = 620 nM). Moreover, one of our initial lead **12** without the *N*,*N*-dimethylamino solubilizing functionality was also a nonselective (EGFR^WT^ IC_50_ = 20 nM *vs* EGFR^L858R/T790M^ IC_50_ = 27 nM) inhibitor.^28,29^ The antiproliferative activity of **12** in both cell lines (CC_50_ > 1 μM) was lower than that of **11**. In addition, it was noted that clinical candidate **11** had a molecular weight (Ml. Wt.) of 533.6 and log *D*_7.4_ of 4.0. Hence, lead optimization effort to develop mutant-selective EGFR inhibitors should also optimize the physicochemical properties to improve potent cellular activity and optimal pharmacokinetics profile. With these goals in mind, we choose **12** as the starting point for the lead optimization effort due to its lower Ml. Wt. (476.5). Moreover, the acrylamide functional group in **12** was also present in several other reported irreversible EGFR inhibitors, including **6–9**.

**Table 1:**
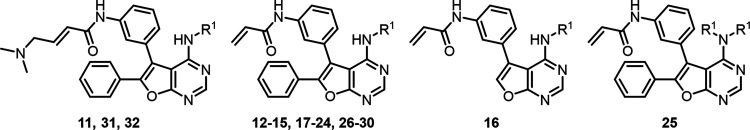

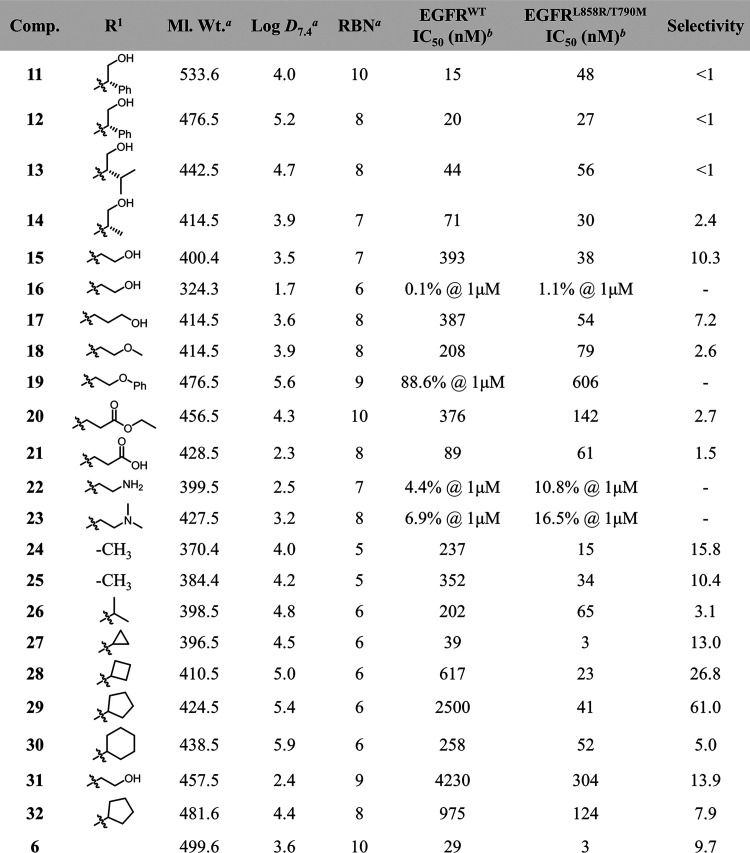
SAR Exploration of the Leads **11** and **12**

a Physicochemical properties were determined using Discovery Studio version 2021.

b IC_50_ values and inhibition ratio of EGFR^WT^ and EGFR^L858R/T790M^ were calculated using the in-house Kinase-Glo assay, and the IC_50_ values represent the mean of at least two independent experiments and are within ±15%.

Having previously established the importance of an acrylamide moiety at the *meta*-position of the phenyl ring for potent EGFR^L858R/T790M^ inhibition,^28^ we retained this functionality and first investigated modifications to the side chain attached to the quinazoline ring 4-position. Molecular docking of **6** and **11** with EGFR^WT^ showed that the phenyl ring in the side chain of **11** established more extensive σ–π hydrophobic interactions in the EGFR back pocket compared to the *N*-methylindole ring of **6**. Hence, we attempted to attenuate this hydrophobic interaction with EGFR^WT^ to improve the selectivity for EGFR^L858R/T790M^. Replacing the phenyl group in the side chain of **12** with the smaller isopropyl group to give **13** (EGFR^WT^ IC_50_ = 44 nM and EGFR^L858R/T790M^ IC_50_ = 56 nM) showed that the presence of an aromatic ring in the side chain is not essential for EGFR inhibition (Table 1). This was further confirmed by synthesizing compounds **14** and **15** bearing a methyl group and hydrogen atom instead of the phenyl group, respectively. Both **14** and **15** potently inhibited EGFR with EGFR^WT^ sparing activity, with **15** (EGFR^WT^ IC_50_ = 393 nM *vs* EGFR^L858R/T790M^ IC_50_ = 38 nM) showing over 10-fold selectivity toward mutant EGFR^L858R/T790M^ over the EGFR^WT^ kinase similar to **6**. Collectively, these results validate the strategy of increasing the inhibition and selectivity for mutant EGFR *vs* EGFR^WT^ by decreasing the σ–π hydrophobic interactions in the back pocket of EGFR. Hence, these investigations spurred us to further explore the role of the *N*-amino ethanol side chain in the 4-position of the furanopyrimidine ring for EGFR selectivity. More importantly, the removal of the phenyl ring from the side chain also resulted in better physicochemical properties like lower Ml. Wt. (400.4) and log *D*_7.4_ (3.5) for **15**, as compared to **12**.

As a next step, **15** was evaluated in an antiproliferative assay using A431 and H1975 lung cancer cell lines. Unexpectedly, **15** showed cellular potency with a CC_50_ > 1 μM in both the cell lines, suggesting that both the enzymatic activity and the compound’s physicochemical properties need to be carefully considered for optimal cellular potency. Hence, to further decrease the Ml. Wt./log *D*_7.4_, the phenyl group attached to the furan ring was removed to obtain **16** (Ml. Wt. = 324.3 and log *D*_7.4_ = 1.7), which resulted in the complete loss of inhibition potency for the EGFR^WT^ and EGFR^L858R/T790M^ kinases (IC_50_ > 1 μM). Subsequently, we focused on the modifications in the *N*-amino ethanol side chain attached to the 4-position quinazoline ring. Increasing the length of the side chain by one carbon (**17**; EGFR^WT^ IC_50_ = 387 nM *vs* EGFR^L858R/T790M^ IC_50_ = 54 nM) did not alter the inhibition as well as selectivity much for the EGFR enzyme. Next, to understand the importance of the hydroxy functionality, it was masked with methyl (**18**) or with a phenyl group (**19**), which resulted in lower levels of EGFR inhibition and the loss of activity was more prominent in the case of bulky phenyl ether **19**. Also, attempts to replace the hydroxy group with an ester (**20**; EGFR^WT^ IC_50_ = 376 nM *vs* EGFR^L858R/T790M^ IC_50_ = 142 nM) and carboxylic acid (**21**; EGFR^WT^ IC_50_ = 89 nM *vs* EGFR^L858R/T790M^ IC_50_ = 61 nM), both resulted in lower levels of EGFR inhibition and a concomitant loss of selectivity toward the mutant EGFR^L858R/T790M^ enzyme. Third, replacement of the hydroxy group of **15** with an amino (**22**) or *N*,*N*-dimethylamino (**23**) group resulted in the loss of EGFR inhibition (IC_50_ > 1 μM) for both the wild-type and mutant enzymes.

Unable to identify a suitable replacement for the hydroxy group, we next investigated if the entire ethanol side chain of **15** could be replaced. Simple *N*-alkyl substitutions such as methyl (**24**), *N*,*N*-dimethyl (**25**), isopropyl (**26**), cyclopropyl (**27**), cyclobutyl (**28**), cyclopentyl (**29**), and cyclohexyl (**30**) groups instead of the ethanol side chain were well-tolerated (EGFR^L858R/T790M^ IC_50_ of 3–65 nM) and also showed varying levels of selectivity (3.1 to 60-fold) toward the mutant EGFR over the EGFR^WT^ (Table 1). It is particularly interesting that **29** with the cyclopentyl substitution at the amino group displayed around 61-fold EGFR^L858R/T790M^ (IC_50_ of 41 nM) mutant-selective kinase inhibition. However, compounds **24–30** were poorly antiproliferative (CC_50_ > 1 μM) in NSCLC cell lines, suggesting that their ability to enter the cells was poor due to unfavorable log *D*_7.4_ of 4.0–5.9 for these compounds.

We next set out to improve the cellular potency of our compounds. Several studies, including ours, have shown that introducing suitable solubilizing functional groups at an appropriate place in the molecules could aid in increasing the cellular potency by altering the physicochemical property, particularly the log *D*_7.4_ values.^30^ For example, the introduction of *N*,*N*-dimethylamino solubilizing group in the acrylamide side chain of the initial lead compound **12** to develop **11** has resulted in a lowering of log *D*_7.4_ value from 5.2 to 4.0. Even though both showed similar levels of EGFR kinase enzyme inhibitions, **11** showed better cellular antiproliferative activity in the H1975 cell line (CC_50_ = 620 nM), but **12** was not active, suggesting that the presence of *N*,*N*-dimethylamino solubilizing group could potentially enhance cellular potency by improving the permeability of the compound into the cells.

Hence, we introduced the *N*,*N*-dimethylamino solubilizing group in the acrylamide side chain in **15** and **29** to prepare two analogues. Both **31** (with the *N*-ethylamino side chain; log *D*_7.4_ = 2.4) and **32** (with the *N*-cyclopentylamino side chain; log *D*_7.4_ = 4.4) showed lower levels of EGFR inhibition (both wild-type and mutant enzymes), compared to **15** and **29**, respectively (Table 1). But both compounds retained selectivity (8 to 14-fold) toward the double-mutant EGFR compared to the EGFR^WT^ enzyme inhibition. Due to lower levels of EGFR enzyme inhibition, both **31** and **32** did not show antiproliferative activity in both the cell lines (CC_50_ > 1 μM). Our preliminary SAR investigations of **11**/**12** suggest that smaller hydrophobic groups such as methyl, isopropyl or cyclopentyl group at the 4-position amino terminus of the furanopyrimidine ring could impart EGFR double-mutant selectivity. However, their enzymatic activity could not be translated to cellular potency due to the poor physicochemical property.

### Molecular Docking and Design Strategy to Improve Cellular Potency

**Figure 2:**
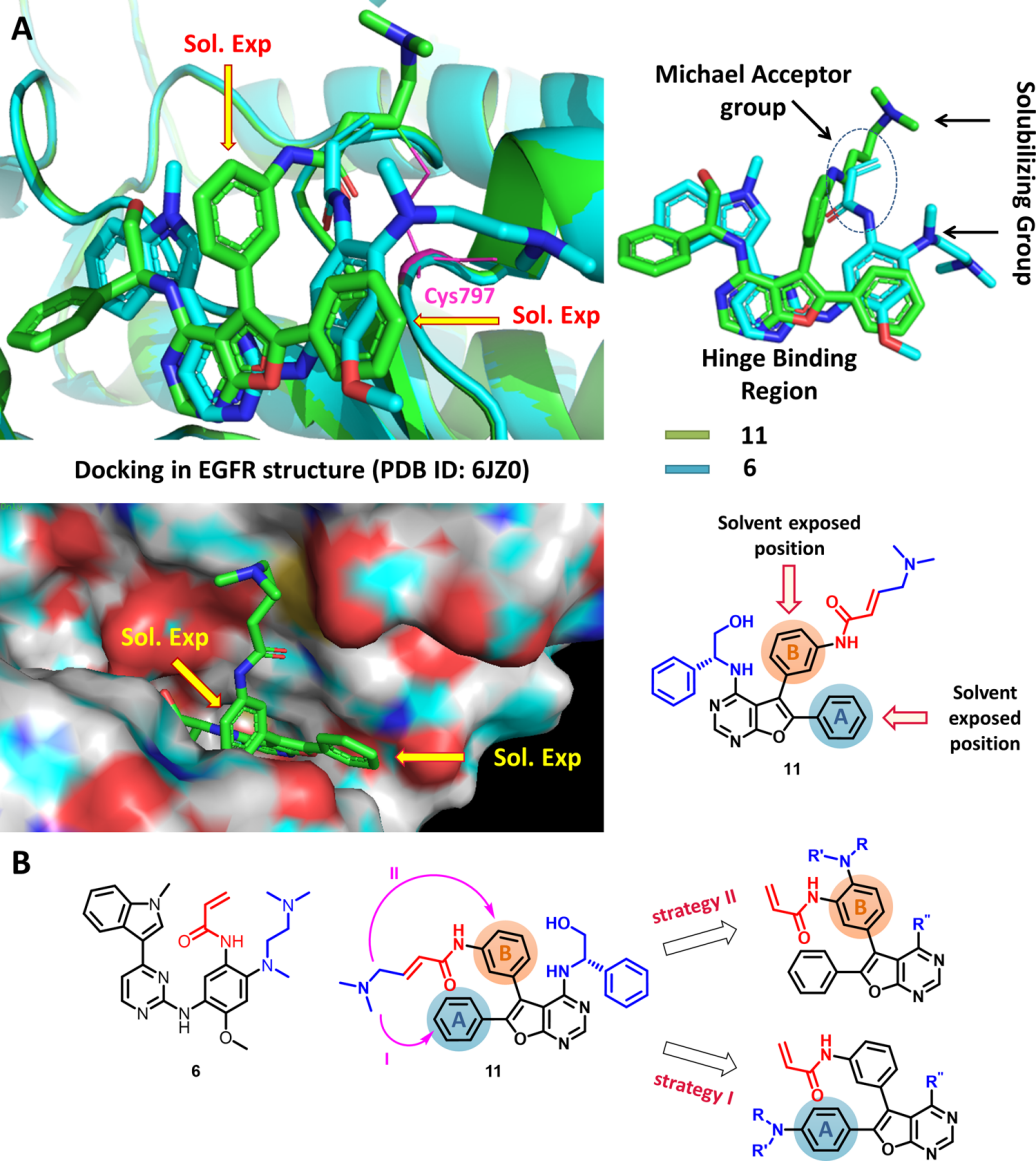
Identification of the suitable substitution site for introducing the solubilizing group in the furanopyrimidine analogues. (A) Docked orientation of **6** superimposed onto the cocrystal binding orientation of **11** in the EGFR structure (PDB ID: 6JZ0). Solvent-exposed regions of **11** suitable for introducing solubilizing groups are shown with arrows. (B) Two strategies (I and II) for introducing solubilizing groups into the phenyl ring of furanopyrimidine analogues are shown.

As the incorporation of a solubilizing functional group in compounds **31** and **32** lowered its EGFR kinase inhibition activity and failed to impart cellular potency, we used molecular docking studies to identify a site capable of bearing a solubilizing group without that group also affecting the EGFR kinase enzyme inhibitory activity. Osimertinib (**6**) was docked onto the crystal structure of DBPR112 (**11**) and their binding orientation in the EGFR protein was compared (Figure 2). It was found that the Michael acceptor group of **6** was placed near the Cys797 residue, similar to **11**. While the solubilizing group *N*,*N*,*N*′-trimethylethyl amino side chain of **6** was placed next to phenyl ring A of **11**, suggesting that this ring could be a suitable option for introducing the solubilizing group instead of phenyl ring B of **11**. Moreover, phenyl ring A is directed toward the solvent-accessible area of the protein, and introducing a solubilizing group in this ring could be well-tolerated without altering the protein–ligand interactions. Additionally, the solubilizing group could also be introduced to the para-position of phenyl ring B, as this position is directed toward the solvent-accessible region of the protein. Hence, it was contemplated to move the solubilizing group to phenyl ring A (strategy I) and the *para*-position of phenyl ring B (strategy II) of furanopyrimidine, as outlined in Figure 2.

### Design Strategy I: SAR Exploration by Introducing Solubilizing Groups in Phenyl Ring A

Based on the results of the molecular docking studies, a selection of solubilizing groups was introduced at the *para*-position of phenyl ring A of **15** to give compounds **33–41**, whose structure–activity relationships (SAR) were then studied (Table 2). Compound **33** bearing an *N*,*N*-dimethylamino group at this position (log *D*_7.4_ = 3.6) was a potent and selective inhibitor of EGFR (EGFR^WT^ IC_50_ = 69 nM *vs* EGFR^L858R/T790M^ IC_50_ = 14 nM) with a mutant selectivity of 5. However, **33** did not exhibit sufficient antiproliferative activity in either cell lines (CC_50_ > 1 μM). Next, morpholine and *N*-methyl piperazine groups were introduced at the *para*-position of phenyl ring A of **15** to give **34** (log *D*_7.4_ = 3.3) and **35** (log *D*_7.4_ = 3.4), respectively, both of which demonstrated potent EGFR enzyme inhibition activity in the low nanomolar range and mutant selectivities of 21-fold for **34** and 3.6-fold for **35**. In the cell-based assay, **34** was poorly antiproliferative (CC_50_ > 1 μM). However, **35** exhibited significantly enhanced antiproliferative activity in the H1975 cell line with an IC_50_ of 126 nM and 21-fold selectivity toward the H1975 cell line as compared to A431 cell line, suggesting a strong EGFR^WT^ sparing cellular antiproliferative activity. With the encouraging results, we have also introduced the solubilizing functional group *N*,*N*,*N*′-trimethylethyl amino side chain, which is present in **6**. Compound **36** (log *D*_7.4_ = 2.6) with this solubilizing group at the *para*-position of phenyl ring A of **15** possessed potent and mutant-selective EGFR enzyme inhibition (EGFR^L858R/T790M^ IC_50_ = 7 nM and 3.6-fold selectivity over EGFR^WT^). However, **36** did not show potent antiproliferative activity in both A431 and H1975 cell lines (CC_50_ > 1 μM). It should be noted that even though **36** had a lower log *D*_7.4_ value, the number of rotatable bonds was more than 10 and could be a possible reason for poor cellular potency.

**Table 2:**
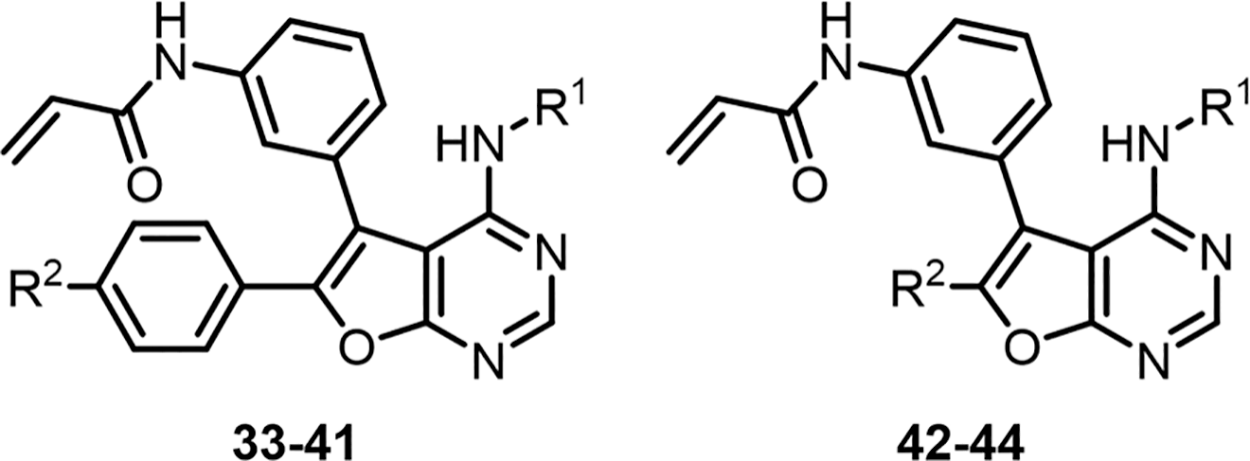

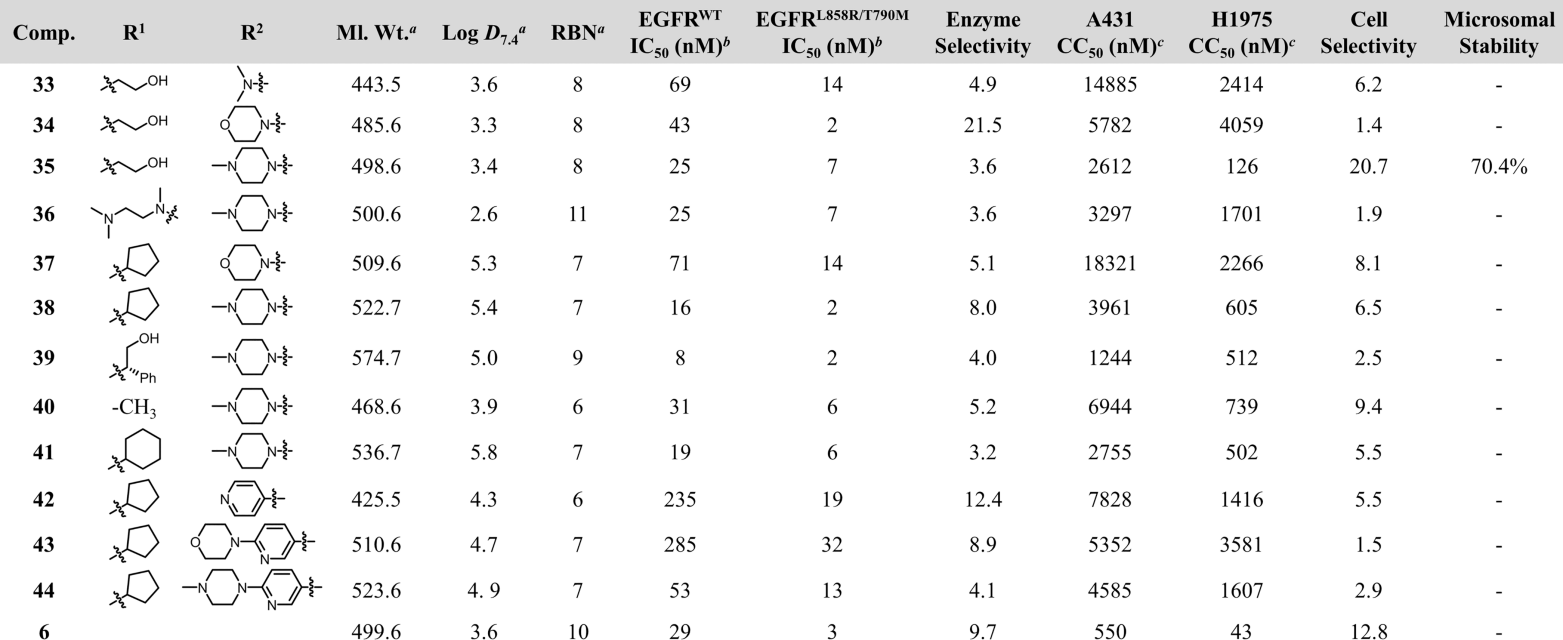
SAR Exploration of **15** and **29** by the Introduction of Solubilizing Groups in Phenyl Ring A

a Physicochemical properties were determined using Discovery Studio version 2021.

b The IC_50_ values and inhibition ratio of EGFR^WT^ and EGFR^L858R/T790M^ were calculated using the in-house Kinase-Glo assay, and the IC_50_ values represent the mean of at least two independent experiments and are within ±15%.

c The CC_50_ values represent the mean of at least two independent experiments and are within ±15%.

Next, we introduced the solubilizing groups morpholine and *N*-methyl piperazine in the *para*-position of phenyl ring A of **29** to prepare **37** and **38**, respectively (Table 2). Both compounds showed excellent EGFR inhibition and 5 to 8-fold selectivity for mutant EGFR over EGFR^WT^. Once again, only the *N*-methyl piperazine analogue **38** showed good cellular potency with a CC_50_ of 600 nM in the H1975 cell line. Interestingly, **38** had a CC_50_ of 3961 nM in A431 cell line overexpressing the EGFR^WT^, suggesting that its selectivity for mutant EGFR over EGFR^WT^ was about 8-fold. As *N*-methyl piperazine bearing compounds **35** and **38** displayed good cellular potency, the *N*-methyl piperazine solubilizing group was also introduced in **12**, **24**, and **30** to synthesize **39**, **40**, and **41**, respectively. All three compounds exhibited potent and selective EGFR enzyme inhibition (EGFR^L858R/T790M^ IC_50_ = 2–6 nM and 3.2 to 5.2-fold selectivity over EGFR^WT^). More importantly, all three compounds exhibited antiproliferative activity in H1975 cell line with a CC_50_ range of 502–739 nM and displayed 2 to 10-fold selectivity over the A431 cell line. In addition, we have also replaced phenyl ring A in **29** with a more hydrophilic 4-pyridine ring in **42**, morpholine-substituted 3-pyridine ring in **43**, and *N*-methyl piperazine-substituted 3-pyridine ring in **44**. These replacements resulted in potent and selective EGFR^L858R/T790M^ inhibition (IC_50_ of 13–32 nM and selectivity of 4.1 to 12-fold over the EGFR^WT^), but did not significantly increase cellular potency (CC_50_ > 1 μM). Overall, the order of cellular enhancement by the solubilizing group in the *para*-position of phenyl ring A is *N*-methyl piperazine > *N*,*N*,*N*′-trimethylethyl amino > morpholine.

### Design Strategy II: SAR Exploration by Introducing Solubilizing Groups in Phenyl Ring B

Next, our attention turned to investigating the second strategy that is focused on introducing solubilizing functional groups in the *para*-position of phenyl ring B of **29**. The introduction of the morpholine group resulted in EGFR^WT^ sparing potent enzyme inhibitor **45** with a selectivity ratio of 14 toward the double-mutant EGFR^L858R/T790M^ (IC_50_ = 8 nM) (Table 3). At the same time, the introduction of *N*-methyl piperazine (**46**) resulted in only a selectivity of 1.8-fold for the double-mutant EGFR^L858R/T790M^ (IC_50_ = 61 nM) over the EGFR^WT^. Furthermore, two other solubilizing functional groups with alkylamino side chains were introduced to the *para*-position of phenyl ring B of **29** through an ether linkage to synthesize compounds **47** and **48**. Even though both maintained good activity and selectivity for the mutant EGFR kinase (IC_50_ of 26–43 nM), they were not active in the cellular antiproliferation assay (CC_50_ > 1 μM). Also, the *N*,*N*,*N*′-trimethylethyl amino side chain, which is present in **6** was introduced in the *para*-position of phenyl ring B of **29**. Compound **49** with this solubilizing group displayed potent double-mutant EGFR^L858R/T790M^ (IC_50_ = 7 nM) activity along with 5-fold selectivity against the EGFR^WT^ enzyme. In the cellular antiproliferation assay, compound **49** selectively inhibited the proliferation of double-mutant overexpressing H1975 NSCLC cells with a CC_50_ of 310 nM and 13-fold selectivity over A431 NSCLC cells overexpressing EGFR^WT^ kinase.

**Table 3:**
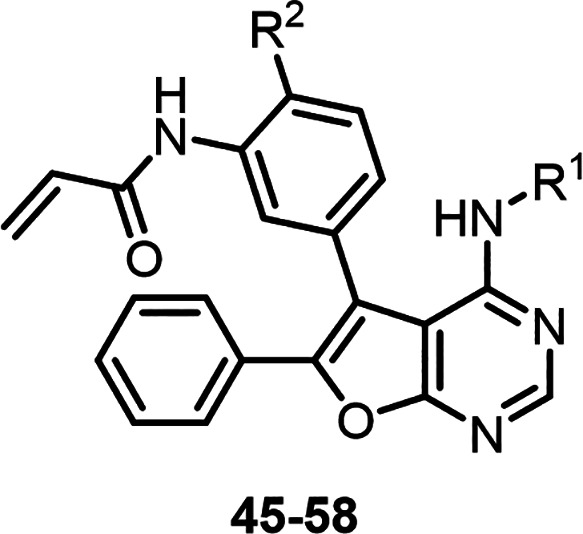

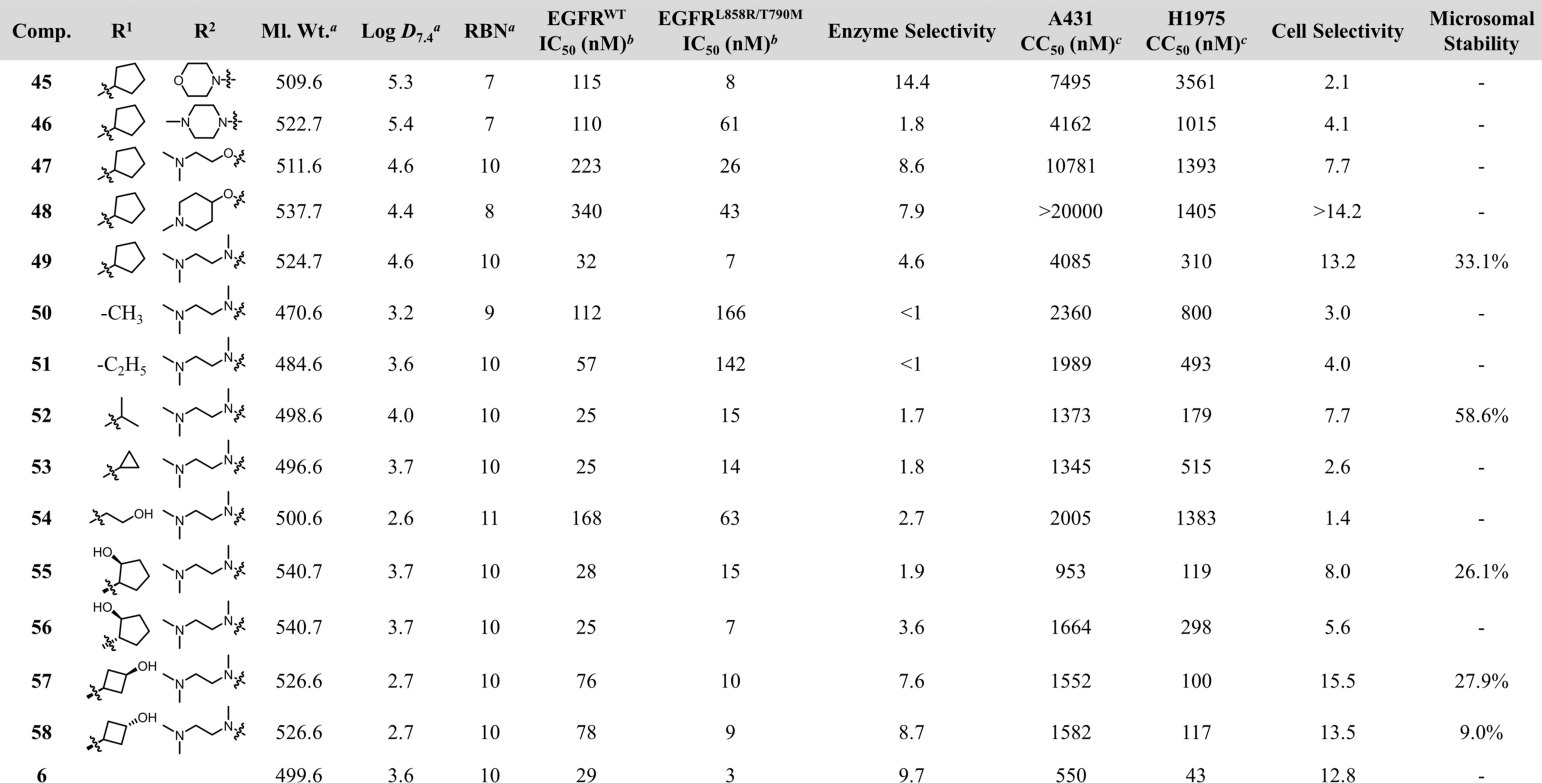
SAR Exploration of **15** and **29** by the Introduction of Solubilizing Groups in Phenyl Ring B

a Physicochemical properties were determined using Discovery Studio version 2021.

b The IC_50_ values and inhibition ratio of EGFR^WT^ and EGFR^L858R/T790M^ were calculated using the in-house Kinase-Glo assay, and the IC_50_ values represent the mean of at least two independent experiments and are within ±15%.

c The CC_50_ values represent the mean of at least two independent experiments and are within ±15%.

With these encouraging results for **49**, we synthesized compounds **50**–**53** bearing methyl (**50**), ethyl (**51**), isopropyl (**52**), and cyclopropyl (**53**) groups in place of the cyclopentyl ring (Table 3). Of these compounds, **52** and **53** showed potent enzymatic and cellular antiproliferative activities and EGFR^WT^ sparing activity. Of particular interest was compound **52**, which displayed over 8-fold selectivity for H1975 over A431 cell lines (CC_50_, A431 = 1373 nM and H1975 = 179 nM). Moreover, we also investigated the *N*-amino ethanol side chain in the furanopyrimidine ring 4-position and found that **54** showed about 2.7-fold selective EGFR^L858R/T790M^ enzyme inhibition over the EGFR^WT^. However, **54** (analogue of **15** bearing the *N*,*N*,*N*′-trimethylethyl amino side chain in the *para*-position of phenyl ring B) showed poor cellular antiproliferative activity in both A431 and H1975 cell lines (CC_50_ > 1 μM).

As a next step, we synthesized stereoisomers **55** and **56**, hydroxylated versions of **49**, to alter the physicochemical properties and improve cellular potency. Compounds **55** and **56** exhibited potent mutant EGFR kinase inhibition (IC_50_ of 15 and 7 nM) and H1975 cellular proliferation inhibition (CC_50_) of 119 and 298 nM, respectively. Moreover, both compounds were EGFR^WT^ sparing, with selectivities of 8.0 and 5.6 in the antiproliferation assay. Similarly, compounds **57** and **58**, bearing a hydroxylated cyclobutyl ring, showed potent mutant EGFR kinase inhibition (IC_50_ of 10 and 9 nM), with a selectivity of 7.6 and 8.7-fold over the EGFR^WT^ kinase enzyme, respectively. More importantly, **57** and **58** similar to **55** and **56** displayed potent (CC_50_, 100 and 117 nM) and selective (15.5 and 13.5-fold over A431) antiproliferative activity in H1975 cell lines.

**Figure 3:**
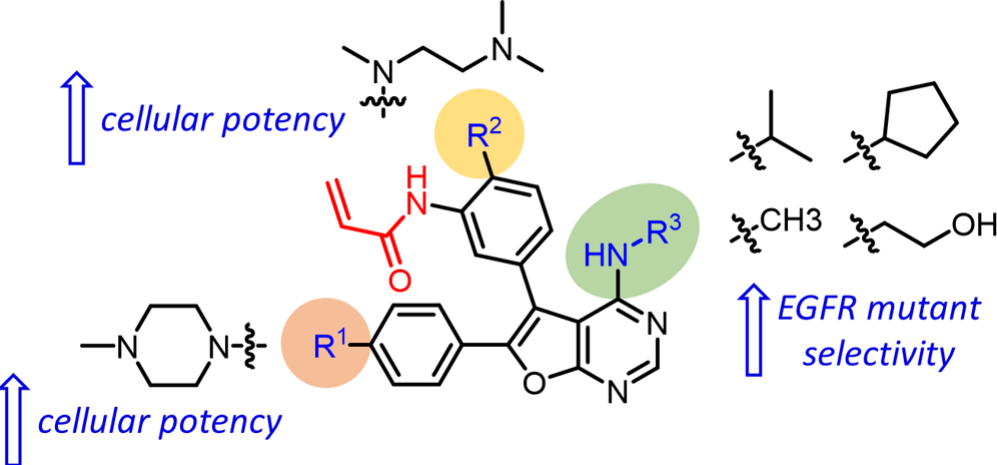
Suitable functional groups identified in this study to improve the EGFR mutant selectivity and cellular potency in furanopyrimidines.

Several compounds bearing a solubilizing functional group in phenyl ring A (**35**, **38**, **39**, **40**, and **41**) and in phenyl ring B (**49**, **50**, **51**, **52**, **53**, **55**, **56**, **57**, and **58**) exhibited EGFR^WT^ sparing potent antiproliferative activity in H1975 cell lines. Analysis of the physicochemical properties of these compounds (Tables 2 and 3) showed that the log *D*_7.4_ values were in the range of 3.4–5.8 for compounds in Table 2 (solubilizing group in phenyl ring A), while it was in the range of 2.7–4.6 for compounds in Table 3 (solubilizing group in phenyl ring B). It is interesting to note that the cellular antiproliferative activities of compounds bearing a solubilizing group on phenyl ring B were better than those bearing that group on phenyl ring A. Our SAR exploration clearly shows the benefit of introducing a solubilizing group in an appropriate position, *N*-methyl piperazine in phenyl ring A and *N*,*N*,*N*′-trimethylethyl amino in phenyl ring B, for modulating the enzymatic and sparing the EGFR^WT^. The SAR findings to improve the EGFR mutant selectivity and cellular potency in furanopyrimidines are summarized in Figure 3.

### *In Vitro* and *In Vivo* Pharmacokinetics Investigations

With several potent compounds in hand, we investigated a few of the most interesting compounds for their ability to resist drug metabolism using an *in vitro* microsomal stability assay. Compounds were individually incubated with rat liver microsomes for 1 h; then, the proportion of unmetabolized compound remaining was estimated using liquid chromatography-tandem mass spectrometry (LC-MS/MS). It was found that **35**, **49**, **52**, **55**, **57**, and **58** were 70.4, 33.1, 58.4, 26.1, 27.9, and 9%, unmetabolized, respectively (Tables 2 and 3). This assay supports the candidacy of **35**, **49**, **52**, and **57** for *in vivo* pharmacokinetics (PK) evaluation as they were more than 25% unmetabolized after 1 h of incubation.

The *in vivo* PK profiles of four compounds, **35**, **49**, **52**, and **57**, as their free base and HCl salts were evaluated in rats (Table 4); the HCl salts were evaluated because they could improve the aqueous solubility of the compounds and, in turn, could improve the PK profile. Neither **35** nor **57** could be detected in plasma after PO administration, presumably due to their high plasma clearance (300 and 82.1 mL/min/kg). However, the HCl salts of **49** and **52** were orally bioavailable with an *F*% of 12.9 and 26.8, respectively. In particular, among the tested compounds, the HCl salt of **52** had the best *T*_1/2_ of 5.1 and 6.9 h after IV and oral administration.

**Table 4:** *In Vivo* Pharmacokinetics Profiles of **35**, **49**, and **52** in Rats^a^

	IV (dose: 5 mg/kg)				PO (dose: 20 mg/kg)				
comp.	*T*_1/2_ (h)	CL (mL/min/kg)	*V*_ss_ (L/kg)	AUC_(0-inf)_ (ng/mL·h)	*C*_max_ (ng/mL)	*T*_max_ (h)	*T*_1/2_ (h)	AUC_(0-inf)_ (ng/mL·h)	*F* (%)
**35**	0.3	300	4.1	278	ND	ND	ND	ND	ND
**49**	2.9	60.6	4.7	1386	68.1	4.0	2.3	603	11
**49S1**	2.2	22	2.2	3912	253	1.7	2.3	2012	12.9
**52S1**	5.1	36.9	7.8	1967	255	0.3	6.9	2107	26.8
**57S1**	2.9	82.1	6.7	894	ND	ND	ND	ND	ND

a S1 means the compound is a HCl salt; ND: not detected.

### Investigation of Cellular Target Modulation by **49** and **52**

The antiproliferative activities of **49** and **52** were investigated in BaF3 cell lines overexpressed with EGFR^L858R/T790M^ mutant kinase. Both compounds showed potent antiproliferative activity with CC_50_ values of 26 and 20 nM, respectively, similar to that of **6**, which had a CC_50_ of 14 nM. The results suggested that both **49** and **52** could inhibit mutant EGFR at the cellular level to produce the antiproliferative activity.

**Figure 4:**
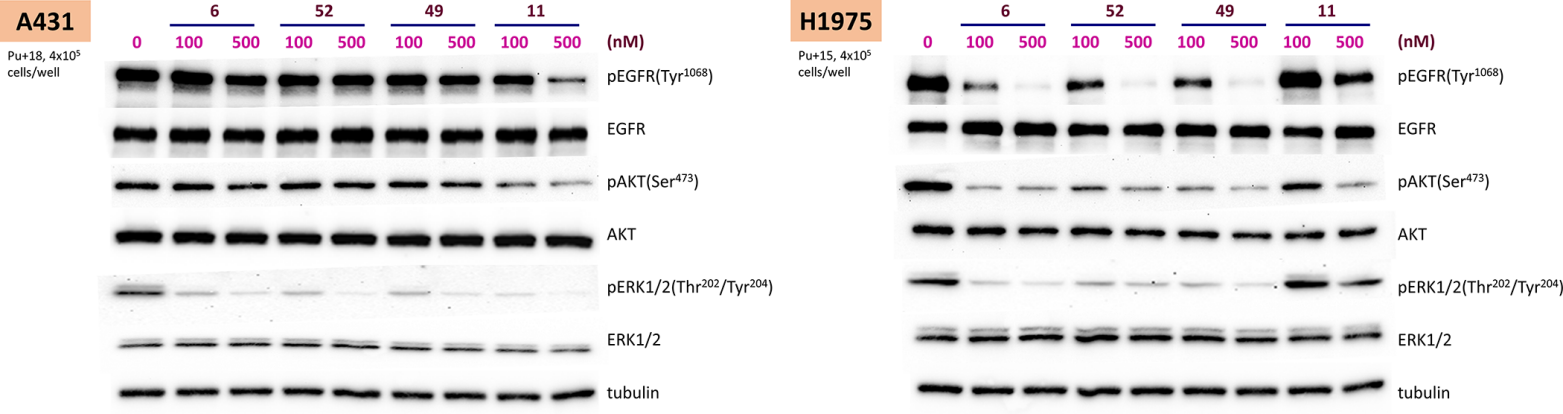
Differential inhibition of wild-type and T790M mutant EGFR in the cellular context by **49** and **52**. Western blot analysis shows that pEGFR(Try^1068^) levels were unaltered in A431 cells expressing EGFR^WT^, but not in the case of H1975 cells expressing EGFR T790M mutant, suggesting mutant-selective inhibition of EGFR kinase activity by **49** and **52** similar to **6** inside the cells.

Further, to confirm the ability of **49** and **52** to inhibit the mutant EGFR selectively inside the cells, A431 and H1975 NSCLC cells were treated with the compounds for 1 h and then the cell extracts were analyzed using western blot for downstream signaling molecule modulation. Treatment with **49**, **52**, and **6** resulted in lower levels of tyrosine^1068^ phosphorylated EGFR [pEGFR(Tyr^1068^)] in the H1975 cell line but not in the A431 cell line (Figure 4). An active EGFR kinase enzyme autophosphorylates the EGFR tyrosine^1068^ residue. Lower levels of pEGFR(Tyr^1068^) in H1975 indicate target (EGFR^L858R/T790M^) inhibition in the cell lines. However, pEGFR(Tyr^1068^) levels were not affected in A431 cell line, suggesting that the target (EGFR^WT^) is not inhibited in this cell line. This further confirms the findings that **49** and **52** are mutant-selective EGFR inhibitors with EGFR^WT^ sparing activity. Concurrent testing of the initial lead molecule **11** showed that it is not a mutant-selective EGFR inhibitor with lower potency. Both **49** and **52** showed similar AKT and ERK1/2 inhibition profiles as that of **6**.

As additional *in vitro* data confirmed that **49** and **52** are potent and EGFR mutant-selective agents, they were subjected to *in vivo* evaluation to determine their ability to inhibit the growth of tumors bearing EGFR^L858R/T790M^ double mutation.

### Evaluation of *In Vivo* Efficacy of Lead Compounds **49** and **52**

**Figure 5:**
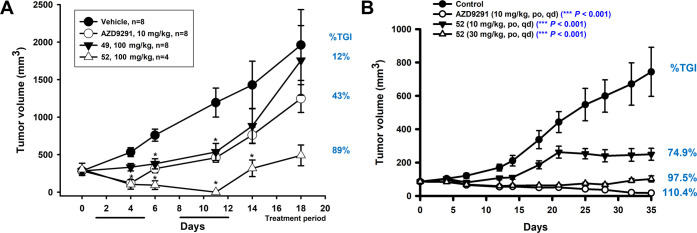
*In vivo* efficacy evaluation of orally administered **49** and **52** in mouse xenograft models. (A) Change in BaF3 EGFR^L858R/T790M^ tumor volume over the treatment period. Drug treatment was for two cycles of 5 days each, with 2 days gap. *P < 0.05, compared to vehicle group. (B) Change in H1975 tumor volume over the treatment period. Tumor growth inhibition (TGI) is shown as percentage, compared to the vehicle-treated group.

For the *in vivo* efficacy evaluation, mice bearing tumors of BaF3 cells overexpressed with EGFR^L858R/T790M^ mutant kinase were used. Animals with the tumor were administered 100 mg/kg of **49** or **52** over two cycles (the first cycle on days 1–5 and the second cycle on days 8–12) of treatment. Tumor volume and body weight changes were measured till 18 days of the treatment initiation. It was found that **52** produced a sustained and significant tumor growth inhibition (TGI) during the treatment cycle (Figure 5A). However, **49** did not show significant TGI upon treatment. Neither treatment altered the body weight of the animals more than 5% (Figure S1). Based on these *in vivo* findings, **52** was chosen for further evaluation in the H1975 lung cancer xenograft mouse model.

For preclinical evaluation in the lung cancer xenograft model, mice were injected with H1975 cells bearing the EGFR^L858R/T790M^ double mutation. After detectable tumor growth, animals were PO-administered with two doses of **52** (10 or 30 mg/kg, QD) daily for 35 days. Tumor volume and body weight were measured during the treatment. The group that received 10 mg/kg of **52** showed 74.9% TGI, while the group administered 30 mg/kg showed 97.5% TGI at the end of the treatment (Figure 5B). The results suggest that **52** can effectively suppress the growth of NSCLC with EGFR double mutation. Moreover, drug treatment did not result in overt body weight loss (<5% change in body weight; Figure S1) during drug treatment, suggesting overall good tolerance for the drug. The *in vivo* pharmacodynamics experiments demonstrate the utility of **52** as an agent to overcome EGFR double-mutant overexpressing non-small cell lung cancer.

### Binding Mode Analysis of **52** by Molecular Docking

To understand the EGFR^WT^ sparing inhibition activity of **52**, the molecular docking of **52** and **11** in EGFR^WT^ (PDB ID: 6JXT) and EGFR^T790M^ (PDB ID: 6JX0) structures was carried out. The wild-type and mutant EGFR proteins were in complex with **6**, and the ligand binding site for docking was designated based on this ligand. Covalent docking of the ligand was carried out with a criterion to undergo nucleophilic addition of the Cys797 thiol group of EGFR protein to the Michael acceptor group present in the ligand. Both the ligands **52** and **11** bound at the active site, forming a H-bond between the furanopyrimidine *N*^1^ and the hinge region Met793 residue. In addition, the protonated *N*,*N*-dimethylamino solubilizing group of **11** made a salt bridge with the Asp800 residue in both wild-type and mutant EGFR, and the protonated *N*,*N*,*N*′-trimethylethyl amino solubilizing group of **52** made salt bridge with the Asp800 residue in the wild-type EGFR and with the Asp855 residue in mutant EGFR (Figure S2). It is interesting to note that the *N*,*N*,*N*′-trimethylethyl amino solubilizing group of **52** bound to the mutant EGFR is projected in the solvent-exposed region of the protein as envisaged in strategy II (Figure 2).

**Figure 6:**
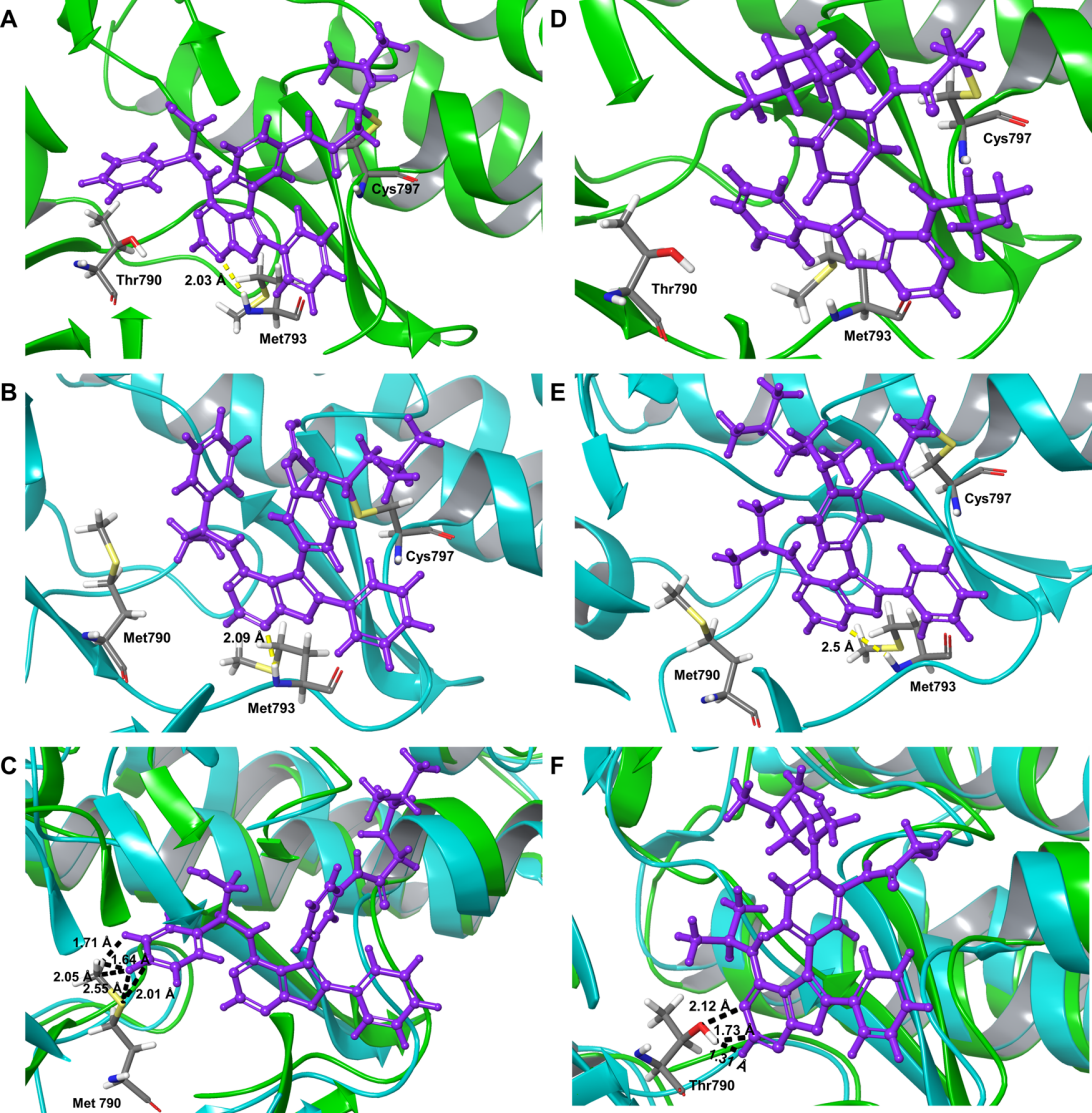
Three-dimensional (3D) docked orientations of **11** (A–C) and **52** (D–F) in EGFR^WT^ (PDB ID: 6JXT) and EGFR^T790M^ (PDB ID: 6JX0) structures. Schrödinger Glide covalent docking module was used to do the docking in the EGFR active site with nucleophilic addition of the Cys797 SH group to the Michael acceptor group of the ligand. (A) **11** in EGFR^WT^, (B) **11** in EGFR^T790M^, (C) superimposition of panels (A) and (B) showing the docked orientation of **11** (in EGFR^WT^) phenyl ring atoms making steric clash (black dotted lines) with Met790 residue of EGFR^T790M^, (D) **52** in EGFR^WT^, (E) **52** in EGFR^T790M^, and (F) superimposition of panels (D) and (E) showing the docked orientation of **52** (in EGFR^T790M^) pyrimidine ring atoms making steric clash (black dotted lines) with the Thr790 residue of EGFR^WT^. H-bond is shown as yellow dotted lines.

Compound **11** docked to EGFR^WT^ with a docking score of −10.927 and to EGFR^T790M^ with a docking score of −7.667, suggesting a better binding to EGFR^WT^ compared to EGFR^T790M^. This could be due to a steric clash between the phenyl ring substituent present in the furanopyrimidine 4-position of **11** and the Met790 residue of EGFR^T790M^, a residue that is not present in wild-type EGFR (Figure 6A–C). In contrast, **52** was bound to EGFR^T790M^ with a better docking score (−8.118) than to EGFR^WT^ (−6.891), suggesting stronger binding to the mutant EGFR than EGFR^WT^. Analysis of the binding orientation shows that **52** was flipped in the ATP binding site of EGFR^WT^ and did not form H-bond with the hinge residue Met793. Further analysis suggests that the pyrimidine ring atoms would make steric clash and/or unfavorable interaction with the polar Thr790 residue, resulting in flipped orientation of the ligand in EGFR^WT^ with concomitant lower binding. However, **52** was bound in the ATP binding site of EGFR^T790M^ with the essential hinge region interaction (Figure 6D–F). The results from covalent docking rationalize the observed EGFR^WT^ sparing activity of **52**.

### Kinase Profiling of Lead **52**

**Figure 7:**
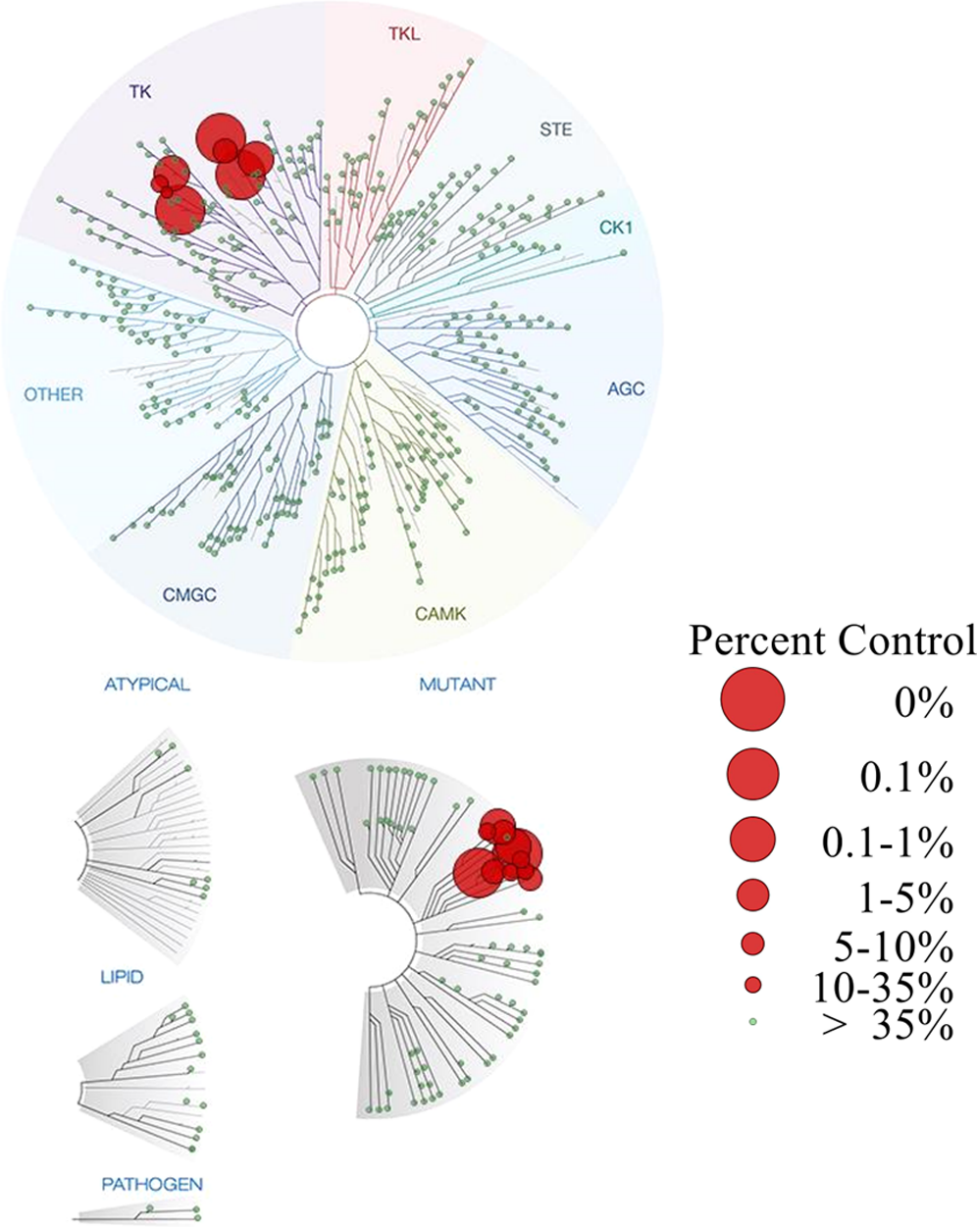
Kinase profiling of **52** using KINOME*scan* technology at 1 μM.

Further, to estimate the specificity and safety profile, a panel of 468 kinases (including 65 mutant kinases) were tested for binding of **52** at a screening concentration of 1 μM using KINOME*scan* technology (Figure 7, Tables S1 and S2). The results revealed that **52** presented an extraordinary kinome selectivity with an *S*(10) score of 0.017 (7/403 nonmutant kinases). Apart from EGFR^WT^ (1.7%), **52** also showed high affinity toward six other kinases bearing a cysteine at the front region including BLK (0.9%), BTK (0%), ERBB2 (0%), ERBB4 (0%), JAK3 (0.4%), and TXK (9.9%).^31^ In addition to these targets, **52** only presented moderate affinity toward another cysteine-containing kinase TEK (14%). The strong affinity between **52** and EGFR^L858R/T790M^ (1.9%) is consistent with the results of enzymatic assays as well. Collectively, the kinase profiling outcome of **52** demonstrated a highly selective EGFR inhibitor, similar to the clinical trial counterpart **11**.

### Chemistry

**Scheme 1:**
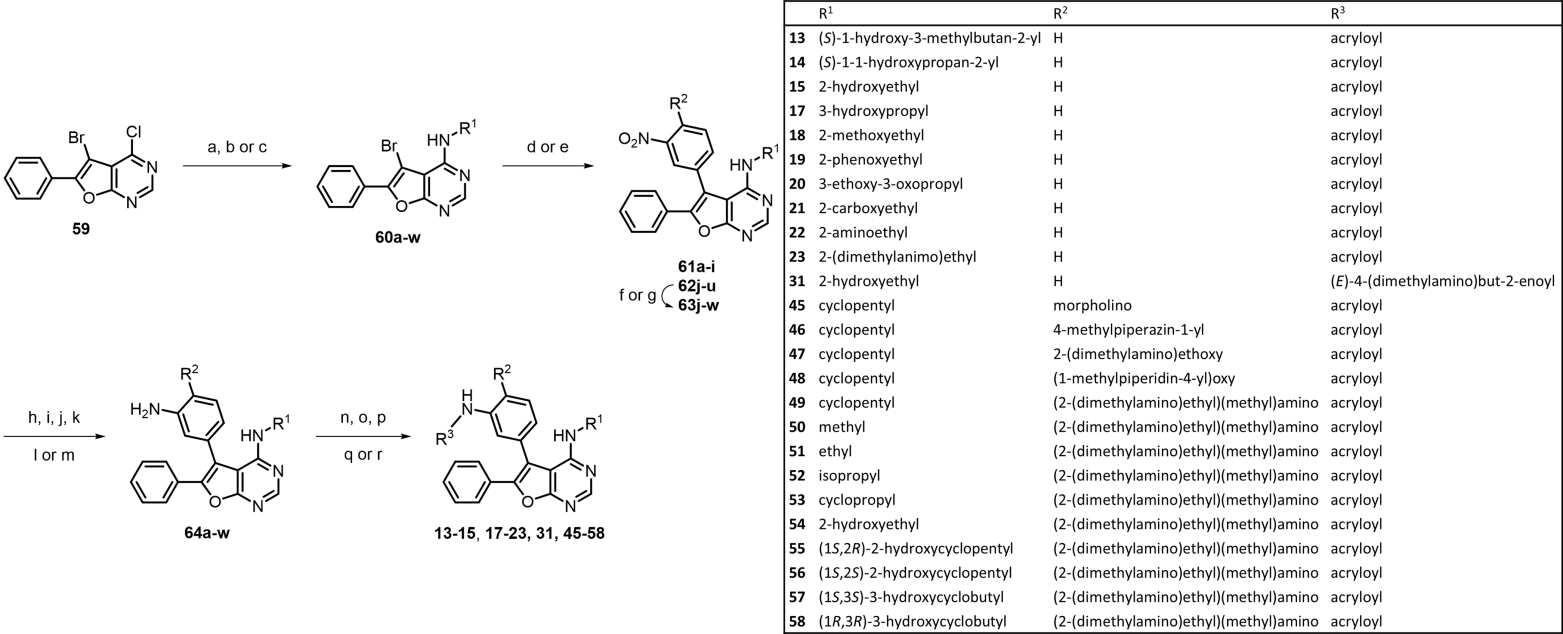
Synthetic Route for EGFR Inhibitors **13–15**, **17–23**, **31**, and **45–58** Reagents and conditions: (a) appropriate amines, Et_3_N, ethanol, reflux, 53–97%; (b) appropriate amines, ^*n*^BuOH, reflux, 62–75%; (c) appropriate amines, DIPEA, ethanol, reflux, 92–98%; (d) appropriate boronic acids or boronic esters, Pd(dppf)Cl_2_, Na_2_CO_3_, 1,4-dioxane, H_2_O, 80 °C, reflux, 2–16 h, 47–93%; (e) (3-nitrophenyl)boronic acid, Pd(dppf)Cl_2_, Na_2_CO_3_, DMF, H_2_O, 150 °C, 1 h, microwave, 60%; (f) appropriate amines, Et_3_N, 1,4-dioxane, 80 °C, reflux, 1–12 h, 60%–quant.; (g) appropriate alcohols, NaH, THF, rt, 16 h, 46–60%; (h) H_2_, Pd/C, methanol or ethanol, rt, 30–99%; (i) from **60d**, (3-aminophenyl)boronic acid, Pd(dppf)Cl_2_, Na_2_CO_3_, 1,4-dioxane, H_2_O, 100 °C, 16 h, 89%; (j) from **60e**, (3-aminophenyl)boronic acid, Pd(dppf)Cl_2_, Na_2_CO_3_, 1,4-dioxane, H_2_O, 150 °C, 1 h, microwave, 43%; (k) SnCl_2_, ethanol, reflux, 1–4 h, 87–94%; (l) iron powder, ethanol, CH_2_Cl_2_, H_2_O, sat. NH_4_Cl_(aq)_, 80 °C, 0.5–16 h, 52–94%; (m) SnCl_2_·2H_2_O, CH_2_Cl_2_, methanol, reflux, 1.5 h, 31–47%; (n) acrylic acid, EDCI, CH_2_Cl_2_, rt, 4–16 h, 14–78%; (o) acryloyl chloride, DIPEA or Et_3_N, CH_2_Cl_2_, rt, 1–2 h, 42–70%; (p) LiOH_(aq)_, THF, rt, 3 h, 96%; (q) TFA, CH_2_Cl_2_, rt, 1 h, 96%; (r) (i) 4-bromocrotonoic acid, EDCI, CH_2_Cl_2_, rt, 16 h; (ii) *N*,*N*-dimethylamine, THF, rt, 4 h, 72%.

Syntheses of compounds **13–15**, **17–23**, **31**, and **45–58** are outlined in Scheme 1. Similar to our previous study, building block **59**(32) was initially subjected to nucleophilic aromatic substitution reaction (S_N_Ar) with various amines to yield the desired compounds **60a–w**. The amino group of **60h** was protected with Boc anhydride to generate **60h′**. Then, the bromo compounds **60a–w** were coupled with 3-nitrophenylboronic acid derivatives or 3-nitro-4-substituted-phenylboronic acid derivatives *via* Suzuki coupling to give compounds **61a–i**, **62j**, **62r**, **62s**, **62u**, and **63j–w**. On the other hand, the 4-fluoro-substituted **62j**, **62r**, and **62s** were reacted with different secondary amines or alcohols under basic conditions to prepare compounds **63j**, **63r**, and **63s**. Reductions of **61a–i** and **63j–w** were carried out using palladium on charcoal in the presence of hydrogen gas, stannic chloride, or iron powder under acidic conditions to give the corresponding amino derivatives **64a–w**. Finally, compounds **64a–w** were reacted with acrylic acid or acryloyl chloride to introduce amide bonds in analogues **13–15**, **17–23**, **31**, and **45–58**.

**Scheme 2:**
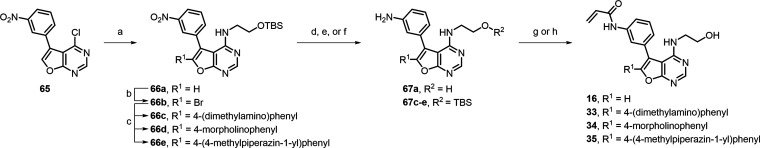
Synthetic Route of EGFR Inhibitors **16** and **33–35** Reagents and conditions: (a) 2-[(*tert*-butyldimethylsilyl)oxy]ethan-1-amine, DIPEA, ethanol, reflux, 64%; (b) NBS, ACN, 100 °C, microwave, time, 99%; (c) 4-substituted-phenylboronic acid, Pd(dppf)Cl_2_·CH_2_Cl_2_, Na_2_CO_3_, 100 °C, 60–80%; (d) SnCl_2_, HCl, CH_2_Cl_2_, reflux, 96%; (e) iron powder, ethanol, CH_2_Cl_2_, sat. NH_4_Cl_(aq)_, 80 °C, 94%; (f) H_2_, Pd/C, ethanol, rt, 60%; (g) acryloyl chloride, DIPEA, THF, rt, 38%; (h) acryloyl chloride, Et_3_N, CH_2_Cl_2_, rt, then TFA, CH_2_Cl_2_, 34–66%.

For the synthesis of final compounds **16** and **33–35** (Scheme 2), previously reported building block **65**(28) was first substituted with the OTBS-protected ethanolamine to give **66a**, which was then brominated at 6-position with *N*-bromosuccinimide in acetonitrile to yield **66b**. Then, Suzuki coupling reaction of **66b** was carried out under microwave irradiation with phenylboronic acids containing different substituents to afford **66c–e**. Reduction of **66a** and **66c–e** was accomplished by either stannic chloride, iron powder, or palladium on charcoal in the presence of hydrogen gas to achieve **67a** and **67c–e**. The acrylation of **67a** with acryloyl chloride in tetrahydrofuran gave the desired compound **16**. On the other hand, the anilines **67c–e** were treated with acryloyl chloride in dichloromethane followed by desilylation with trifluoroacetic acid to obtain the target compounds **33–35**.

**Scheme 3:**
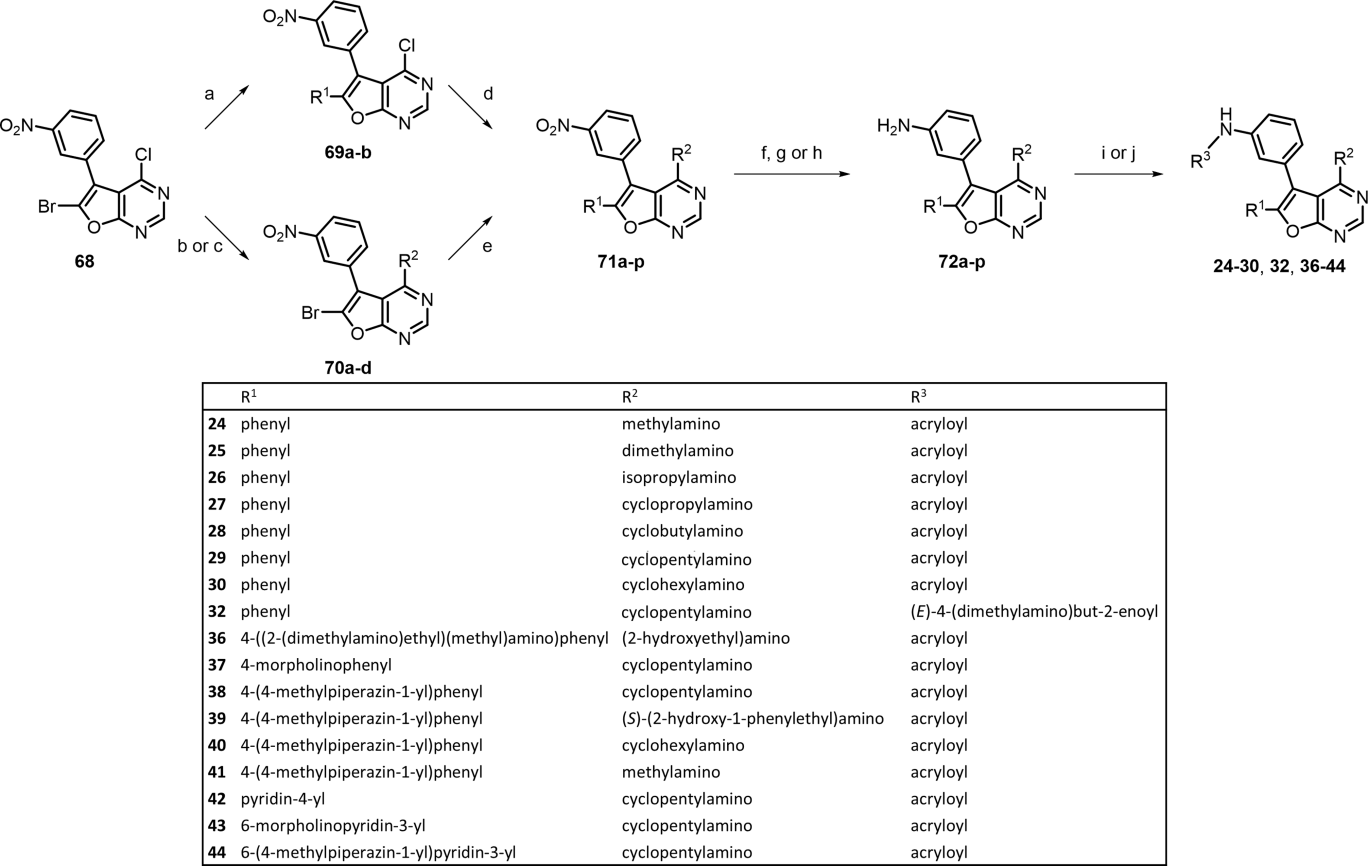
Synthetic Route of EGFR Inhibitors **24–30**, **32**, and **36–44** Reagents and conditions: (a) appropriate boronic acids, Pd(dppf)Cl_2_, Na_2_CO_3_, 1,4-dioxane, H_2_O, 75–80 °C, 3–12 h, 53–55%; (b) appropriate amines, Et_3_N, ethanol or IPA, reflux, 1.5–16 h, 84–95%; (c) TBSCl, Et_3_N, DMF, CH_2_Cl_2_, rt, 16 h, 79%; (d) appropriate amines, Et_3_N or DIPEA, ethanol, reflux, 4–16 h, 51–95%; (e) appropriate boronic acids or boronic esters, Pd(dppf)Cl_2_, Na_2_CO_3_, 1,4-dioxane, H_2_O, 80 °C, reflux, 1–16 h, 65–96%; (f) H_2_, Pd/C, methanol, rt, 1–16 h, 83–98%; (g) SnCl_2_·2H_2_O, CH_2_Cl_2_, methanol or ethanol, 70 °C, reflux, 2–4 h, 64–99%; (h) iron powder, ethanol, CH_2_Cl_2_, H_2_O, sat. NH_4_Cl_(aq)_, 80 °C, 2 h, 38–85%; (i) acrylic acid, EDCI, CH_2_Cl_2_, rt, 1–16 h, 14–86%; (j) (i) 4-bromocrotonoic acid, EDCI, CH_2_Cl_2_, rt, 16 h; (ii) *N*,*N*-dimethylamine, THF, rt, 5 h, 35%.

The final compounds **24–30**, **32**, and **36–44** were obtained from the previously reported building block **68**(28) and the synthetic route is illustrated in Scheme 3. To generate various intermediates **71** efficiently from **68**, two different routes were employed. For compounds **24–29** and **41**, building block **68** was initially coupled with phenylboronic acid or 4-(4-methylpiperazin-1-yl)phenylboronic acid *via* Suzuki cross-coupling to achieve compounds **69a** and **69b**. Then, **69a** was treated with different amines through nucleophilic aromatic substitution to give **71a–f**. The 4-chloride of furanopyrimidine **69b** was coupled with methylamine to afford **71m**. On the other hand, building block **68** was also reacted with various amines under basic conditions to yield amines **70a–d** in which **70b** was subsequently transformed into OTBS-protected **70b′**. Then, **70a–d** was treated with numerous amines to give **71g–o**. The nitro functional group of intermediates **71a–p** was then reduced to amino groups with stannic chloride or iron powder to afford anilines **72a–p**. Finally, amide bond formation was achieved by treatment of **72a–p** with acrylic acid to obtain the desired EGFR inhibitors **24–30**, **32**, and **36–44**.

## Conclusions

The propensity of NSCLC to resist EGFR inhibitors must be addressed if the burden of the disease is to be mitigated. The acquired T790M mutation results in resistance to first-generation EGFR inhibitors, making these drugs ineffective. Second-generation inhibitors targeting Cys797 such as compound **11** can overcome resistance due to the T790M mutation, but their poor selectivity for mutant EGFR kinase over wild-type EGFR kinase may cause clinical toxicity. Here, through structure and property-guided lead optimization efforts, we first attenuated hydrophobic interaction in the back pocket by replacing (*S*)-2-phenylglycinol to *N*-amino ethanol or cyclopentylamino substituent at the 4-position of the furanopyrimidine ring to achieve mutant selectivity. Furthermore, based on molecular modeling studies, two different strategies were applied to enhance cellular potency by implanting solubilizing groups, such as *N*-methyl piperazine in phenyl ring A and *N*,*N*,*N*′-trimethylethyl amino group in phenyl ring B to obtain various EGFR mutant-selective inhibitors (**35**, **38**, **39**, **40** and **41**, **49**, **50**, **51**, **52**, **53**, **55**, **56**, **57**, and **58**) that selectively inhibited the proliferation of H1975 over A431 NSCLC cells. After DMPK assessment by *in vitro* microsomal stability and *in vivo* pharmacokinetics studies, **52** is selected as an orally active agent that effectively inhibits tumor formation in animal models with T790M-mutated EGFR. Further preclinical evaluation of **52** is underway to develop it as a backup compound for clinical trial agent **11** (DBPR112).

## Experimental Section

### General Methods for Chemistry

All commercial chemicals and solvents are of reagent grade and used without further purification unless otherwise stated. All reactions were carried out under dry nitrogen or argon atmosphere and were monitored for completion by TLC using Merck 60 F254 silica gel glass-backed plates or aluminum plates; zones were detected visually under UV irradiation (254 nm) or by spraying with potassium permanganate reagent (Aldrich) followed by heating at 80 °C. Flash column chromatography was carried out using silica gel (Silicycle SiliaFlash P60, R12030B, 230–400 mesh or Merck Grade 9385, 230–400 mesh). ^1^H and ^13^C NMR spectra were recorded with Varian Mercury-300 or Varian Mercury-400 spectrometers or Bruker 400 or 600 MHz AVANCE III spectrometers. Data analysis was done using Mnova software (Mestrelab Research). Chemical shift (δ) was reported in ppm and referenced to solvent residual signals as follows: DMSO-*d*_6_ at 2.50 ppm, CDCl_3_ at 7.26 ppm, and CD_3_OD at 3.31 ppm for ^1^H NMR; DMSO-*d*_6_ at 39.5 ppm and CDCl_3_ at 77.0 ppm for ^13^C NMR. Splitting patterns are indicated as follows: s = singlet; d = doublet; t = triplet; q = quartet; quin = quintet; dd = doublet of doublets; dt = doublet of triplets; dq = doublet of quartet; dquin = doublet of quintet; dset = doublet of septet; td = triplet of doublets; tt = triplet of triplets; qd = quartet of doublets, ddd = doublet of doublets of doublets; br = broad; m = multiplet. Coupling constants (*J*) were given in hertz (Hz). Low-resolution mass spectra (LRMS) data were measured with an Agilent MSD-1100 ESI-MS/MS system or Agilent Infinity II 1290 LC/MS (ESI) systems. High-resolution mass spectra (HRMS) data were measured with a Varian 901-MS FT-ICR HPLC/MS–MS system. Purity of the final compounds was determined using a high-performance liquid chromatography (HPLC) system (Hitachi 2000 series) equipped with a C18 column (Agilent ZORBAX Eclipse XDB-C18 5 μm. 4.6 mm × 150 mm) and operating at 25 °C. For method A, elution was carried out using acetonitrile as mobile phase A and water containing 0.1% formic acid + 10 mmol NH_4_OAc as mobile phase B. Elution conditions: at 0 min, phase A 10% + phase B 90%; at 45 min, phase A 90% + phase B 10%; at 50 min, phase A 10% + phase B 90%; at 60 min, phase A 10% + phase B 90%. For method B, elution was carried out using acetonitrile as mobile phase A and water containing 0.1% formic acid + 2 mmol NH_4_OAc as mobile phase B. Elution conditions: at 0 min, phase A 10% + phase B 90%; at 25 min, phase A 90% + phase B 10%; at 30 min, phase A 90% + phase B 10%; at 30.5 min, phase A 10% + phase B 90%; at 37 min, phase A 10% + phase B 90%. The flowrate of the mobile phase was 0.5 mL/min, and the injection volume of the sample was 10 or 20 μL. Peaks were detected at 254 nm. The purity of all tested compounds was determined and confirmed to be greater than 95% by HPLC analysis except for compounds **15** (84.5%), **19** (91.5%), **23** (92.7%), **25** (93.1%), **27** (93.8%), **32** (91.6%), **36** (89.6%), **39** (83.7%), **40** (94.2%), **49** (91.9%), **50** (92.2%), and **54** (93.4%). IUPAC nomenclature of the compounds was obtained with Mnova software (Mestrelab Research).

#### *N*-[3-(4-{[(2*S*)-1-Hydroxy-3-methylbutan-2-yl]amino}-6-phenylfuro[2,3-*d*]pyrimidin-5-yl)phenyl]prop-2-enamide (**13**)

To a solution of **64a** (200 mg, 0.51 mmol, 1.0 equiv) in dichloromethane (5.0 mL) were added acrylic acid (40 μL, 0.58 mmol, 1.1 equiv) and EDCI (109 mg, 0.57 mmol, 1.1 equiv); then, the reaction mixture was stirred at room temperature. After stirring for 4 h, to the reaction mixture was added H_2_O (10 mL), extracted with dichloromethane (10 mL × 3), and washed with brine (10 mL). The combined organic layers were dried over MgSO_4_, concentrated *in vacuo*, and purified by flash column chromatography (2–5% methanol in dichloromethane) to yield the title compound **13** (33 mg, 0.07 mmol, 14%) as a white solid. ^1^H NMR (600 MHz, DMSO-*d*_6_) δ 10.36 (s, 1H), 8.32 (s, 1H), 7.90 (s, 1H), 7.84 (d, *J* = 8.4 Hz, 1H), 7.56 (t, *J* = 7.8 Hz, 1H), 7.52–7.47 (m, 2H), 7.39–7.34 (m, 2H), 7.32 (ddd, *J* = 8.4, 7.2, 1.8 Hz, 1H), 7.27 (d, *J* = 7.2 Hz, 1H), 6.43 (dd, *J* = 17.1, 10.2 Hz, 1H), 6.26 (dd, *J* = 17.1, 1.8 Hz, 1H), 5.76 (dd, *J* = 10.2, 1.8 Hz, 1H), 4.93 (d, *J* = 9.6 Hz, 1H), 4.64 (t, *J* = 5.4 Hz, 1H), 4.05 (td, *J* = 9.6, 3.6 Hz, 1H), 3.42–3.37 (m, 1H), 3.30–3.24 (m, 1H), 1.86–1.74 (m, 1H), 0.77 (d, *J* = 6.6 Hz, 3H), 0.57 (d, *J* = 6.6 Hz, 3H). ^13^C NMR (150 MHz, DMSO-*d*_6_) δ 164.4, 163.4, 157.5, 154.1, 145.5, 140.3, 132.1, 131.6, 130.4, 129.0, 128.9, 128.7, 127.4, 125.8, 124.3, 119.8, 119.6, 115.1, 102.3, 60.4, 56.1, 27.7, 19.4, 16.8. LRMS (ESI) *m*/*z*: 443.2 [M + H]^+^. HRMS (ESI) *m*/*z*: calcd for C_26_H_27_N_4_O_3_, 443.2083 [M + H]^+^; found, 443.2085. HPLC purity (method A): 100% (*t*_R_ = 32.42 min).

#### *N*-[3-(4-{[(2*S*)-1-Hydroxypropan-2-yl]amino}-6-phenylfuro[2,3-*d*]pyrimidin-5-yl)phenyl]prop-2-enamide (**14**)

To a solution of **64b** (20 mg, 0.06 mmol, 1.0 equiv) in dichloromethane (0.5 mL) were added acrylic acid (4.2 μL, 0.06 mmol, 1.1 equiv) and EDCI (12 mg, 0.06 mmol, 1.1 equiv); then, the reaction mixture was stirred at room temperature. After stirring for 16 h, the reaction mixture was concentrated *in vacuo* and purified by thin-layer chromatography (2–15% methanol in dichloromethane) to yield the title compound **14** (3 mg, 0.01 mmol, 14%) as a white solid. ^1^H NMR (400 MHz, DMSO-*d*_6_) δ 10.33 (s, 1H), 8.35 (s, 1H), 7.90–7.78 (m, 2H), 7.59–7.44 (m, 3H), 7.43–7.29 (m, 3H), 7.24 (d, *J* = 7.6 Hz, 1H), 6.42 (dd, *J* = 17.0, 10.0 Hz, 1H), 6.25 (dd, *J* = 17.0, 2.2 Hz, 1H), 5.77 (dd, *J* = 10.0, 2.2 Hz, 1H), 5.18 (d, *J* = 7.6 Hz, 1H) 4.70 (t, *J* = 5.2 Hz, 1H), 4.23–4.08 (m, 1H), 3.32–3.27 (m, 2H), 1.02 (d, *J* = 6.8 Hz, 3H). ^13^C NMR (150 MHz, DMSO-*d*_6_) δ 164.5, 163.4, 156.7, 154.1, 145.5, 140.1, 131.9, 131.6, 130.3, 129.0, 128.8, 128.7, 127.3, 126.0, 124.3, 119.9, 119.6, 115.1, 102.1, 63.5, 47.6, 17.0. LRMS (ESI) *m*/*z*: 415.2 [M + H]^+^. HRMS (ESI) *m*/*z*: calcd for C_24_H_22_N_4_NaO_3_, 437.1590 [M + Na]^+^; found, 437.1587. HPLC purity (method A): 98.1% (*t*_R_ = 28.29 min).

#### *N*-(3-{4-[(2-Hydroxyethyl)amino]-6-phenylfuro[2,3-*d*]pyrimidin-5-yl}phenyl)prop-2-enamide (**15**)

To a solution of **64c** (100 mg, 0.29 mmol, 1.0 equiv) in dichloromethane (5.0 mL) were added acrylic acid (30 μL, 0.44 mmol, 1.5 equiv) and EDCI (83 mg, 0.43 mmol, 1.5 equiv); then, the reaction mixture was stirred at room temperature. After stirring for 16 h, the reaction mixture was concentrated *in vacuo* and purified by thin-layer chromatography (0–10% methanol in dichloromethane) to yield the title compound **15** (75 mg, 0.19 mmol, 65%) as a white solid. ^1^H NMR (600 MHz, DMSO-*d*_6_) δ 10.32 (s, 1H), 8.34 (s, 1H), 7.86 (ddd, *J* = 8.4, 2.4, 1.5 Hz, 1H), 7.80 (dd, *J* = 2.4, 1.5 Hz, 1H), 7.53 (t, *J* = 8.4 Hz, 1H), 7.48–7.44 (m, 2H), 7.38–7.30 (m, 3H), 7.24–7.21 (m, 1H), 6.43 (dd, *J* = 16.8, 10.2 Hz, 1H), 6.26 (dd, *J* = 16.8, 1.8 Hz, 1H), 5.77 (dd, *J* = 10.2, 1.8 Hz, 1H), 5.50 (t, *J* = 5.4 Hz, 1H), 4.64 (t, *J* = 4.8 Hz, 1H), 3.49–3.41 (m, 4H). ^13^C NMR (150 MHz, DMSO-*d*_6_) δ 164.5, 163.4, 157.2, 154.0, 145.6, 140.1, 131.8, 131.7, 130.4, 129.0, 128.8, 128.7, 127.3, 126.0, 124.4, 120.0, 119.7, 115.2, 102.1, 59.1, 42.8. LRMS (ESI) *m*/*z*: 401.2 [M + H]^+^. HRMS (ESI) *m*/*z*: calcd for C_23_H_21_N_4_O_3_, 401.1614 [M + H]^+^; found, 401.1610. HPLC purity (method A): 84.5% (*t*_R_ = 26.43 min).

#### *N*-(3-{4-[(2-Hydroxyethyl)amino]furo[2,3-*d*]pyrimidin-5-yl}phenyl)prop-2-enamide (**16**)

To a solution of **67a** (59 mg, 0.22 mmol, 1.0 equiv) in THF (10.0 mL) at 0 °C were added triethylamine (36 μL, 0.26 mmol, 1.2 equiv) and a solution of acryloyl chloride (19 μL, 0.24 mmol, 1.1 equiv) in THF (5.0 mL); then, the reaction mixture was stirred at room temperature. After stirring for 3 h, the reaction mixture was concentrated *in vacuo* and purified by flash column chromatography (5% methanol in dichloromethane) to yield the title compound **16** (27 mg, 0.08 mmol, 38%) as a white solid. ^1^H NMR (600 MHz, DMSO-*d*_6_) δ 10.33 (s, 1H), 8.34 (s, 1H), 8.00 (s, 1H), 7.91 (dd, *J* = 2.4, 1.8 Hz, 1H), 7.66 (ddd, *J* = 8.4, 2.4, 1.2 Hz, 1H), 7.48 (dd, *J* = 8.4, 7.2 Hz, 1H), 7.26 (ddd, *J* = 7.2, 1.8, 1.2 Hz, 1H), 6.46 (dd, *J* = 17.1, 10.2 Hz, 1H), 6.29 (dd, *J* = 17.1, 1.5 Hz, 1H), 6.05 (t, *J* = 5.4 Hz, 1H), 5.79 (dd, *J* = 10.2, 1.5 Hz, 1H), 4.70 (td, *J* = 5.4, 1.2 Hz, 1H), 3.60–3.50 (m, 4H). ^13^C NMR (150 MHz, DMSO-*d*_6_) δ 166.4, 163.5, 157,5, 153.8, 139.7, 138.4, 131.7, 131.2, 129.9, 127.3, 123.3, 120.5, 119.2, 119.1, 99.0, 59.2, 43.0. LRMS (ESI) m/z: 325.1 [M + H]^+^. HRMS (ESI) *m*/*z*: calcd for C_17_H_17_N_4_O_3_, 325.1301 [M + H]^+^; found, 325.1303. HPLC purity (method A): 96.0% (*t*_R_ = 18.46 min).

#### *N*-(3-{4-[(3-Hydroxypropyl)amino]-6-phenylfuro[2,3-*d*]pyrimidin-5-yl}phenyl)prop-2-enamide (**17**)

To a solution of **64d** (31 mg, 0.09 mmol, 1.0 equiv) in dichloromethane (10.0 mL) at 0 °C were added DIPEA (36 μL, 0.17 mmol, 2.0 equiv) and a solution of acryloyl chloride (8 μL, 0.10 mmol, 1.2 equiv) in dichloromethane (1.0 mL); then, the reaction mixture was stirred at room temperature. After stirring for 2 h, the reaction mixture was concentrated *in vacuo* and purified by thin-layer chromatography (5% methanol in dichloromethane) to yield the title compound **17** (25 mg, 0.06 mmol, 70%) as a white solid. ^1^H NMR (600 MHz, DMSO-*d*_6_) δ 10.33 (s, 1H), 8.34 (s, 1H), 7.84 (dd, *J* = 8.4, 1.8 Hz, 1H), 7.79 (dd, *J* = 1.8, 1.8 Hz, 1H), 7.53 (t, *J* = 7.8 Hz, 1H), 7.45 (d, *J* = 7.8 Hz, 2H), 7.38–7.29 (m, 3H), 7.21 (d, *J* = 7.8 Hz, 1H), 6.43 (dd, *J* = 17.4, 10.2 Hz, 1H), 6.26 (dd, *J* = 17.4, 2.1 Hz, 1H), 5.77 (dd, *J* = 10.2, 2.1 Hz, 1H), 5.46 (t, *J* = 6.0 Hz, 1H), 4.39 (t, *J* = 5.7, 1H), 3.46 (td, *J* = 6.6, 5.7 Hz, 2H), 3.37–3.33 (m, 2H), 1.56 (quin, *J* = 6.6 Hz, 2H). ^13^C NMR (150 MHz, DMSO-*d*_6_) *δ* 164.5, 163.4, 157.1, 154.0, 145.5, 140.0, 131.8, 131.6, 130.3, 129.0, 128.8, 128.7, 127.3, 126.0, 124.5, 120.1, 119.7, 115.2, 102.1, 58.6, 38.2, 31.6. LRMS (ESI) *m*/*z*: 415.1 [M + H]^+^. HRMS (ESI) *m*/*z*: calcd for C_24_H_23_N_4_O_3_, 415.1770 [M + H]^+^; found, 415.1769. HPLC purity (method A): 99.6% (*t*_R_ = 26.85 min).

#### *N*-(3-{4-[(2-Methoxyethyl)amino]-6-phenylfuro[2,3-*d*]pyrimidin-5-yl}phenyl)prop-2-enamide (**18**)

To a solution of **64e** (150 mg, 0.42 mmol, 1.0 equiv) in dichloromethane (5.0 mL) at 0 °C were added triethylamine (70 μL, 0.50 mmol, 1.2 equiv) and a solution of acryloyl chloride (40 μL, 0.49 mmol, 1.2 equiv) in dichloromethane (2.0 mL); then, the reaction mixture was stirred at room temperature. After stirring for 1 h, the reaction mixture was concentrated *in vacuo* and purified by CombiFlash automated flash chromatography (2% methanol in dichloromethane) to yield the title compound **18** (148 mg, 0.36 mmol, 86%) as a light-yellow solid. ^1^H NMR (600 MHz, DMSO-*d*_6_) δ 10.36 (s, 1H), 8.35 (s, 1H), 7.87 (ddd, *J* = 8.4, 2.4, 1.2 Hz, 1H), 7.83 (dd, *J* = 2.4, 2.4 Hz, 1H), 7.54 (t, *J* = 7.8 Hz, 1H), 7.49 (d, *J* = 8.4 Hz, 2H), 7.39–7.30 (m, 3H), 7.20 (ddd, *J* = 7.8, 2.4, 1.2 Hz, 1H), 6.44 (dd, *J* = 16.8, 10.2 Hz, 1H), 6.26 (dd, *J* = 16.8, 1.8 Hz, 1H), 5.78 (dd, *J* = 10.2, 1.8 Hz, 1H), 5.35 (t, *J* = 5.4 Hz, 1H), 3.52 (td, *J* = 5.4, 5.4 Hz, 2H), 3.10 (s, 3H). ^13^C NMR (150 MHz, DMSO-*d*_6_) δ 164.5, 163.4, 157.0, 154.1, 145.6, 140.2, 131.9, 131.6, 130.4, 129.0, 128.9, 128.8, 127.4, 126.0, 124.4, 119.8, 119.6, 115.1, 102.3, 69.8, 57.9, 39.9. LRMS (ESI) *m*/*z*: 415.1 [M + H]^+^. HRMS (ESI) *m*/*z*: calcd for C_24_H_22_N_4_NaO_3_, 437.1590 [M + Na]^+^; found, 437.1593. HPLC purity (method A): 100% (*t*_R_ = 31.83 min).

#### *N*-(3-{4-[(2-Phenoxyethyl)amino]-6-phenylfuro[2,3-*d*]pyrimidin-5-yl}phenyl)prop-2-enamide (**19**)

To a solution of **64f** (34 mg, 0.08 mmol, 1.0 equiv) in dichloromethane (15.0 mL) at 0 °C were added triethylamine (17 μL, 0.12 mmol, 2.2 equiv) and a solution of acryloyl chloride (8 μL, 0.10 mmol, 1.2 equiv) in dichloromethane (5.0 mL); then, the reaction mixture was stirred at room temperature. After stirring for 1 h, the reaction mixture was concentrated *in vacuo* and purified by flash column chromatography (100% dichloromethane) to yield the title compound **19** (16 mg, 0.03 mmol, 42%) as a yellow solid. ^1^H NMR (600 MHz, DMSO-*d*_6_) δ 10.31 (s, 1H), 8.39 (s, 1H), 7.85–7.81 (m, 2H), 7.51–7.44 (m, 3H), 7.39–7.30 (m, 3H), 7.23 (dd, *J* = 8.7, 7.5 Hz, 2H), 7.19 (ddd, *J* = 7.2, 1.8, 1.2 Hz, 1H), 6.91 (td, *J* = 7.5, 1.2 Hz, 1H), 6.80 (dd, *J* = 8.7, 1.2 Hz, 1H), 6.39 (dd, *J* = 16.8, 10.2 Hz, 1H), 6.25 (dd, *J* = 16.8, 1.8 Hz, 1H), 5.76 (dd, *J* = 10.2, 1.8 Hz, 1H), 5.53 (t, *J* = 6.0 Hz, 1H), 4.03 (t, *J* = 6.0 Hz, 2H), 3.79 (td, *J* = 6.0, 5.4 Hz, 2H). ^13^C NMR (150 MHz, DMSO-*d*_6_) δ 164.5, 163.4, 158.0, 157.0, 154.0, 145.8, 140.1, 131.8, 131.6, 130.3, 129.4, 128.9, 128.83, 128.77, 127,2, 126.0, 124.4, 120.7, 119.9, 119.7, 115.1, 114.4, 102.4, 65.6, 39.5. LRMS (ESI) *m*/*z*: 477.2 [M + H]^+^. HRMS (ESI) *m*/*z*: calcd for C_29_H_25_N_4_O_3_, 477.1927 [M + H]^+^; found, 477.1923. HPLC purity (method A): 91.5% (*t*_R_ = 40.64 min).

#### Ethyl 3-({5-[3-(Acryloylamino)phenyl]-6-phenylfuro[2,3-*d*]pyrimidin-4-yl}amino)propanoate (**20**)

To a solution of **64g** (400 mg, 0.99 mmol, 1.0 equiv) in dichloromethane (10.0 mL) at 0 °C were added triethylamine (170 μL, 1.22 mmol, 1.2 equiv) and a solution of acryloyl chloride (99 μL, 1.23 mmol, 1.2 equiv) in dichloromethane (5.0 mL); then, the reaction mixture was stirred at room temperature. After stirring for 1 h, the reaction mixture was concentrated *in vacuo* and purified by CombiFlash automated flash chromatography (2% methanol in dichloromethane) to yield the title compound **20** (261 mg, 0.57 mmol, 58%) as a brown solid. ^1^H NMR (600 MHz, DMSO-*d*_6_) δ 10.33 (s, 1H), 8.36 (s, 1H), 7.83 (ddd, *J* = 8.4, 2.4, 1.5 Hz, 1H), 7.80 (dd, *J* = 1.8, 1.5 Hz, 1H), 7.52 (t, *J* = 7.8 Hz, 1H), 7.47–7.43 (m, 2H), 7.38–7.30 (m, 3H) 7.18–7.15 (m, 1H), 6.43 (dd, *J* = 17.1, 10.2 Hz, 1H), 6.35 (dd, *J* = 17.1, 1.8 Hz, 1H), 5.77 (dd, *J* = 10.2, 1.8 Hz, 1H), 5.55 (t, *J* = 6.0 Hz, 1H), 3.96 (q, *J* = 7.2 Hz, 2H), 3.63 (td, *J* = 6.0, 6.0 Hz, 2H), 2.52 (t, *J* = 6.6 Hz, 2H), 1.09 (t, *J* = 7.2 Hz, 3H). ^13^C NMR (150 MHz, DMSO-*d*_6_) δ 171.5, 164.6, 163.4, 156.9, 154.0, 145.7, 140.1, 131.7, 131.6, 130.3, 129.0, 128.84, 128.79, 127.3, 126.1, 124.5, 120.0, 119.7, 115.1, 102.3, 60.0, 36.2, 33.3, 14.0. LRMS (ESI) *m*/*z*: 457.1 [M + H]^+^. HRMS (ESI) *m*/*z*: calcd for C_26_H_25_N_4_O_4_, 457.1876 [M + H]^+^; found, 457.1799. HPLC purity (method A): 98.6% (*t*_R_ = 35.47 min).

#### 3-({5-[3-(Acryloylamino)phenyl]-6-phenylfuro[2,3-*d*]pyrimidin-4-yl}amino)propanoic Acid (**21**)

To a solution of **20** (164 mg, 0.36 mmol, 1.0 equiv) in THF (10.0 mL) at 0 °C was added 0.5 M LiOH_(aq)_ (2 mL, 1.02 mmol, 2.8 equiv); then, the reaction mixture was stirred at room temperature. After stirring for 3 h, the reaction mixture was concentrated *in vacuo* and purified by CombiFlash automated flash chromatography (5% methanol in dichloromethane) to yield the title compound **21** (142 mg, 0.33 mmol, 92%) as a light-yellow solid. ^1^H NMR (600 MHz, DMSO-*d*_6_) δ 10.40–10.31 (m, 1H), 8.36 (s, 1H), 7.87 (ddd, *J* = 8.4, 2.4, 1.2 Hz, 1H), 7.76–7.71 (m, 1H), 7.51 (t, *J* = 8.4 Hz, 1H), 7.47–7.43 (m, 2H), 7.39–7.30 (m, 3H) 7.19–7.15 (m, 1H), 6.43 (dd, *J* = 16.8, 10.2 Hz, 1H), 6.26 (dd, *J* = 16.8, 2.1 Hz, 1H), 5.76 (dd, *J* = 10.2, 2.1 Hz, 1H), 5.69–5.63 (m, 1H), 3.60 (td, *J* = 6.6, 6.0 Hz, 2H), 2.47–2.41 (m, 2H). ^13^C NMR (150 MHz, DMSO-*d*_6_) δ 173.3, 164.6, 163.4, 156.9, 154.0, 145.7, 140.0, 131.7, 131.6, 130.3, 129.0, 128.8, 128.7, 127.2, 126.1, 124.4, 120.1, 119.7, 115.2, 102.2, 36.4, 33.4. LRMS (ESI) *m*/*z*: 429.1 [M + H]^+^. HRMS (ESI) *m*/*z*: calcd for C_24_H_21_N_4_O_4_, 429.1563 [M + H]^+^; found, 429.1560. HPLC purity (method A): 98.8% (*t*_R_ = 26.74 min).

#### *N*-(3-{4-[(2-Aminoethyl)amino]-6-phenylfuro[2,3-*d*]pyrimidin-5-yl}phenyl)prop-2-enamide (**22**)

To a solution of **22′** (215 mg, 0.43 mmol, 1.0 equiv) in dichloromethane (5.0 mL) was added trifluoroacetic acid (1.0 mL); then, the reaction mixture was stirred at room temperature. After stirring for 1 h, the reaction mixture was concentrated *in vacuo* and purified by flash column chromatography (3–20% methanol in dichloromethane) to yield the title compound **22** (165 mg, 0.41 mmol, 96%) as a white solid. ^1^H NMR (600 MHz, DMSO-*d*_6_) δ 10.48 (s, 1H), 8.40 (s, 1H), 7.90 (s, 1H), 7.81–7.68 (m, 3H), 7.50 (td, *J* = 7.8, 1.8 Hz, 1H), 7.44 (d, *J* = 7.8 Hz, 2H), 7.40–7.31 (m, 3H), 7.19 (dd, *J* = 7.8, 1.2 Hz, 1H), 6.47 (dd, *J* = 17.1, 10.2 Hz, 1H), 6.29 (dd, *J* = 17.1, 2.1 Hz, 1H), 5.88 (t, *J* = 6.0 Hz, 1H), 5.79 (dd, *J* = 10.2, 2.1 Hz, 1H), 3.66 (td, *J* = 6.3, 6.0 Hz, 2H), 3.02 (td, *J* = 6.3, 1.8 Hz, 2H). ^13^C NMR (150 MHz, DMSO-*d*_6_) δ 164.7, 163.6, 157.3, 153.7, 146.1, 139.8, 131.6, 131.5, 130.2, 128.92, 128.90, 128.8, 127.5, 126.4, 124.9, 120.6, 119.9, 115.3, 102.5, 38.6, 38.2. LRMS (ESI) *m*/*z*: 400.1 [M + H]^+^. HRMS (ESI) *m*/*z*: calcd for C_23_H_22_N_5_O_2_, 400.1774 [M + H]^+^; found, 400.1779. HPLC purity (method A): 96.9% (*t*_R_ = 19.93 min).

#### *N*-(3-(4-{[2-(Dimethylamino)ethyl]amino}-6-phenylfuro[2,3-*d*]pyrimidin-5-yl)phenyl)prop-2-enamide (**23**)

To a solution of **64i** (324 mg, 0.87 mmol, 1.0 equiv) in dichloromethane (5.0 mL) were added triethylamine (160 μL, 1.15 mmol, 1.3 equiv) and acryloyl chloride (85 μL, 1.05 mmol, 1.2 equiv); then, the reaction mixture was stirred at room temperature. After stirring for 4 h, the reaction mixture was concentrated *in vacuo* and purified by flash column chromatography (1–5% methanol in dichloromethane) to yield the title compound **23** (185 mg, 0.43 mmol, 50%) as a pale orange solid. ^1^H NMR (600 MHz, DMSO-*d*_6_) δ 10.62 (s, 1H), 8.39 (s, 1H), 7.93 (dd, *J* = 2.4, 1.8 Hz, 1H), 7.85 (d, *J* = 7.8 Hz, 1H), 7.49 (t, *J* = 8.4 Hz, 1H), 7.45 (d, *J* = 7.2 Hz, 2H), 7.39–7.31 (m, 3H), 7.18 (ddd, *J* = 7.8, 1.8, 1.2 Hz, 1H), 6.53 (dd, *J* = 17.4, 10.2 Hz, 1H), 6.27 (dd, *J* = 17.4, 2.1 Hz, 1H), 5.89 (t, *J* = 6.0 Hz, 1H), 5.77 (dd, *J* = 10.2, 2.1 Hz, 1H), 3.73 (s, 2H), 3.11 (s, 2H), 2.64 (s, 6H). ^13^C NMR (150 MHz, DMSO-*d*_6_) δ 164.7, 163.5, 157.1, 153.8, 146.0, 140.1, 131.7, 131.4, 130.2, 128.9, 128.82, 128.81, 127.2, 126.2, 124.8, 120.2, 119.7, 115.3, 102.5, 55.8, 42.8, 40.0, 36.2. LRMS (ESI) *m*/*z*: 428.2 [M + H]^+^. HRMS (ESI) *m*/*z*: calcd for C_25_H_26_N_5_O_2_, 428.2087 [M + H]^+^; found, 428.2089. HPLC purity (method A): 92.7% (*t*_R_ = 21.41 min).

#### *N*-{3-[4-(Methylamino)-6-phenylfuro[2,3-*d*]pyrimidin-5-yl]phenyl}prop-2-enamide (**24**)

To a solution of **72a** (40 mg, 0.13 mmol, 1.0 equiv) in dichloromethane (3.0 mL) were added DIPEA (4 μL, 0.02 mmol, 18 mol %), acrylic acid (11 μL, 0.16 mmol, 1.3 equiv), and EDCI (29 mg, 0.15 mmol, 1.2 equiv); then, the reaction mixture was stirred at room temperature. After stirring for 16 h, to the reaction mixture was added ethyl acetate (5 mL) and washed with NaHCO_3(aq)_ (10 mL) and brine (10 mL). The combined organic layers were dried over MgSO_4_, concentrated *in vacuo* and purified by flash column chromatography (33–66% ethyl acetate in hexane) to yield the title compound **24** (32 mg, 0.09 mmol, 68%) as a white solid. ^1^H NMR (600 MHz, DMSO-*d*_6_) δ 10.34 (s, 1H), 8.36 (s, 1H), 7.85 (ddd, *J* = 8.4, 2.1, 1.2 Hz, 1H), 7.75 (dd, *J* = 2.1, 1.8 Hz, 1H), 7.52 (t, *J* = 7.8 Hz, 1H), 7.43 (d, *J* = 7.2 Hz, 2H), 7.38–7.29 (m, 3H), 7.17 (ddd, *J* = 7.8, 1.8, 1.2 Hz, 1H), 6.44 (dd, *J* = 17.1, 10.2 Hz, 1H), 6.27 (dd, *J* = 17.1, 2.4 Hz, 1H), 5.77 (dd, *J* = 10.2, 2.4 Hz, 1H), 5.54 (q, *J* = 4.8 Hz, 1H), 2.89 (d, *J* = 4.8 Hz, 3H). ^13^C NMR (150 MHz, DMSO-*d*_6_) δ 164.5, 163.4, 157.6, 154.0, 145.5, 140.0, 131.8, 131.6, 130.3, 129.0, 128.8, 128.7, 127.3, 126.1, 124.7, 120.2, 119.8, 115.3, 102.1, 28.0. LRMS (ESI) *m*/*z*: 371.1 [M + H]^+^. HRMS (ESI) *m*/*z*: calcd for C_22_H_17_N_4_O_2_, 369.1352 [M – H]^+^; found, 369.1351. HPLC purity (method B): 97.0% (*t*_R_ = 21.52 min).

#### *N*-{3-[4-(Dimethylamino)-6-phenylfuro[2,3-*d*]pyrimidin-5-yl]phenyl}prop-2-enamide (**25**)

To a solution of **72b** (40 mg, 0.12 mmol, 1.0 equiv) in dichloromethane (3.0 mL) were added DIPEA (4 μL, 0.02 mmol, 18 mol %), acrylic acid (11 μL, 0.16 mmol, 1.3 equiv), and EDCI (28 mg, 0.15 mmol, 1.2 equiv); then, the reaction mixture was stirred at room temperature. After stirring for 16 h, to the reaction mixture was added ethyl acetate (5 mL) and washed with NaHCO_3(aq)_ (10 mL) and brine (10 mL). The combined organic layers were dried over MgSO_4_, concentrated *in vacuo*, and purified by flash column chromatography (17–66% ethyl acetate in hexane) to yield the title compound **25** (27 mg, 0.07 mmol, 58%) as a light-yellow solid. ^1^H NMR (600 MHz, DMSO-*d*_6_) δ 10.29 (s, 1H), 8.36 (s, 1H), 7.85 (ddd, *J* = 8.4, 2.1, 1.2 Hz, 1H), 7.69 (dd, *J* = 2.1, 1.8 Hz, 1H), 7.47 (t, *J* = 7.8 Hz, 1H), 7.41 (d, *J* = 7.2 Hz, 2H), 7.37–7.29 (m, 3H), 7.14 (ddd, *J* = 7.2, 1.8, 1.2 Hz, 1H), 6.42 (dd, *J* = 16.8, 10.2 Hz, 1H), 6.24 (dd, *J* = 16.8, 1.8 Hz, 1H), 5.76 (dd, *J* = 10.2, 1.8 Hz, 1H), 2.71 (s, 6H). ^13^C NMR (150 MHz, DMSO-*d*_6_) δ 166.3, 163.3, 159.8, 152.3, 145.6, 139.7, 134.2, 131.7, 129.8, 129.3, 128.7, 128.6, 127.2, 126.5, 125.4, 120.7, 119.2, 116.1, 103.5, 40.2. LRMS (ESI) *m*/*z*: 385.1 [M + H]^+^. HRMS (ESI) *m*/*z*: calcd for C_23_H_21_N_4_O_2_, 385.1665 [M + H]^+^; found, 385.1677. HPLC purity (method B): 93.1% (*t*_R_ = 23.09 min).

#### *N*-{3-[6-Phenyl-4-(propan-2-ylamino)furo[2,3-*d*]pyrimidin-5-yl]phenyl}prop-2-enamide (**26**)

To a solution of **72c** (30 mg, 0.09 mmol, 1.0 equiv) in dichloromethane (3.0 mL) were added DIPEA (3 μL, 0.02 mmol, 20 mol %), acrylic acid (8 μL, 0.12 mmol, 1.4 equiv), and EDCI (20 mg, 0.10 mmol, 1.2 equiv); then, the reaction mixture was stirred at room temperature. After stirring for 16 h, to the reaction mixture was added ethyl acetate (5 mL) and washed with NaHCO_3(aq)_ (10 mL) and brine (10 mL). The combined organic layers were dried over MgSO_4_, concentrated *in vacuo*, and purified by flash column chromatography (17–50% ethyl acetate in hexane) to yield the title compound **26** (27 mg, 0.07 mmol, 58%) as a white solid. ^1^H NMR (600 MHz, DMSO-*d*_6_) δ 10.38 (s, 1H), 8.36 (s, 1H), 7.93 (dd, *J* = 2.1, 1.8 Hz, 1H), 7.79 (ddd, *J* = 7.8, 2.1, 1.2 Hz, 1H), 7.56 (t, *J* = 8.4 Hz, 1H), 7.50 (d, *J* = 7.8 Hz, 2H), 7.40–7.31 (m, 3H), 7.24 (ddd, *J* = 7.8, 1.8, 1.2 Hz, 1H), 6.43 (dd, *J* = 16.8, 10.2 Hz, 1H), 6.27 (dd, *J* = 16.8, 2.1 Hz, 1H), 5.78 (dd, *J* = 10.2, 2.1 Hz, 1H), 4.85 (d, *J* = 7.2 Hz, 1H), 4.18 (dset, *J* = 7.2, 6.3 Hz, 1H), 1.03 (d, *J* = 6.3 Hz, 6H). ^13^C NMR (150 MHz, DMSO-*d*_6_) δ 164.5, 163.5, 156.4, 154.1, 145.5, 140.1, 132.0, 131.6, 130.3, 128.94, 128.86, 128.8, 127.4, 126.0, 124.5, 119.9, 119.7, 115.0, 102.0, 42.3, 22.0. LRMS (ESI) *m*/*z*: 399.1 [M + H]^+^. HRMS (ESI) *m*/*z*: calcd for C_24_H_23_N_4_O_2_, 399.1821 [M + H]^+^; found, 399.1814. HPLC purity (method B): 99.1% (*t*_R_ = 25.66 min).

#### *N*-{3-[4-(Cyclopropylamino)-6-phenylfuro[2,3-*d*]pyrimidin-5-yl]phenyl}prop-2-enamide (**27**)

To a solution of **72d** (30 mg, 0.09 mmol, 1.0 equiv) in dichloromethane (3.0 mL) were added DIPEA (3 μL, 0.02 mmol, 20 mol %mol %), acrylic acid (8 μL, 0.12 mmol, 1.3 equiv), and EDCI (20 mg, 0.10 mmol, 1.2 equiv); then, the reaction mixture was stirred at room temperature. After stirring for 16 h, to the reaction mixture was added ethyl acetate (5 mL) and washed with NaHCO_3(aq)_ (10 mL) and brine (10 mL). The combined organic layers were dried over MgSO_4_, concentrated *in vacuo*, and purified by flash column chromatography (33–66% ethyl acetate in hexane) to yield the title compound **27** (27 mg, 0.07 mmol, 78%) as a white solid. ^1^H NMR (600 MHz, DMSO-*d*_6_) δ 10.35 (s, 1H), 8.42 (s, 1H), 7.84 (dd, *J* = 2.4, 1.8 Hz, 1H), 7.79 (ddd, *J* = 8.4, 2.4, 1.2 Hz, 1H), 7.53 (t, *J* = 7.8 Hz, 1H), 7.48 (d, *J* = 10.2 Hz, 2H), 7.39–7.31 (m, 3H), 7.20 (ddd, *J* = 7.8, 1.8, 1.2 Hz, 1H), 6.43 (dd, *J* = 17.1, 10.2 Hz, 1H), 6.27 (dd, *J* = 17.1, 2.1 Hz, 1H), 5.78 (dd, *J* = 10.2, 2.1 Hz, 1H), 5.26 (d, *J* = 3.3 Hz, 1H), 2.79 (dset, *J* = 3.6, 3.3 Hz, 1H), 0.73–0.67 (m, 2H), 0.34–0.29 (m, 2H). ^13^C NMR (150 MHz, DMSO-*d*_6_) δ 164.4, 163.5, 158.1, 154.0, 145.8, 140.0, 131.9, 131.6, 130.3, 128.9, 128.84, 128.83, 127.4, 126.1, 124.6, 120.0, 119.7, 115.0, 102.4, 23.9, 7.0. LRMS (ESI) *m*/*z*: 397.1 [M + H]^+^. HRMS (ESI) *m*/*z*: calcd for C_24_H_21_N_4_O_2_, 397.1665 [M + H]^+^; found, 397.1663. HPLC purity (method B): 93.8% (*t*_R_ = 22.85 min).

#### *N*-{3-[4-(Cyclobutylamino)-6-phenylfuro[2,3-*d*]pyrimidin-5-yl]phenyl}prop-2-enamide (**28**)

To a solution of **72e** (38 mg, 0.11 mmol, 1.0 equiv) in dichloromethane (3.0 mL) were added DIPEA (4 μL, 0.02 mmol, 22 mol %), acrylic acid (10 μL, 0.15 mmol, 1.4 equiv), and EDCI (27 mg, 0.14 mmol, 1.3 equiv); then, the reaction mixture was stirred at room temperature. After stirring for 16 h, to the reaction mixture was added ethyl acetate (5 mL) and washed with NaHCO_3(aq)_ (10 mL) and brine (10 mL). The combined organic layers were dried over MgSO_4_, concentrated *in vacuo*, and purified by flash column chromatography (17–33% ethyl acetate in hexane) to yield the title compound **28** (31 mg, 0.08 mmol, 71%) as a white solid. ^1^H NMR (600 MHz, DMSO-*d*_6_) δ 10.40 (s, 1H), 8.34 (s, 1H), 7.93 (dd, *J* = 2.1, 1.8 Hz, 1H), 7.80 (ddd, *J* = 8.4, 2.1, 1.2 Hz, 1H), 7.57 (t, *J* = 8.4 Hz, 1H), 7.50 (d, *J* = 7.2 Hz, 2H), 7.40–7.32 (m, 3H), 7.25 (ddd, *J* = 7.2, 1.8, 1.2 Hz, 1H), 6.44 (dd, *J* = 17.1, 10.2 Hz, 1H), 6.27 (dd, *J* = 17.1, 1.8 Hz, 1H), 5.78 (dd, *J* = 10.2, 1.8 Hz, 1H), 5.26 (d, *J* = 7.5 Hz, 1H), 4.50 (dquin, *J* = 7.8, 7.5 Hz, 1H), 2.27–2.20 (m, 2H), 1.70–1.57 (m, 4H). ^13^C NMR (150 MHz, DMSO-*d*_6_) δ 164.6, 163.5, 156.1, 154.0, 145.7, 140.0, 132.0, 131.6, 130.3, 128.9, 128.85, 128.81, 127.4, 126.1, 124.6, 120.1, 119.8, 115.0, 102.0, 45.5, 30.5, 14.6. LRMS (ESI) *m*/*z*: 411.2 [M + H]^+^. HRMS (ESI) *m*/*z*: calcd for C_25_H_23_N_4_O_2_, 411.1821 [M + H]^+^; found, 411.1813. HPLC purity (method B): 99.2% (*t*_R_ = 26.92 min).

#### *N*-{3-[4-(Cyclopentylamino)-6-phenylfuro[2,3-*d*]pyrimidin-5-yl]phenyl}prop-2-enamide (**29**)

To a solution of **72f** (37 mg, 0.10 mmol, 1.0 equiv) in dichloromethane (3.0 mL) were added DIPEA (3 μL, 0.02 mmol, 17 mol %), acrylic acid (9 μL, 0.14 mmol, 1.4 equiv), and EDCI (23 mg, 0.12 mmol, 1.2 equiv); then, the reaction mixture was stirred at room temperature. After stirring for 16 h, to the reaction mixture was added ethyl acetate (5 mL) and washed with NaHCO_3(aq)_ (10 mL) and brine (10 mL). The combined organic layers were dried over MgSO_4_, concentrated *in vacuo*, and washed with cold methanol (5 mL) to yield the title compound **29** (20 mg, 0.05 mmol, 47%) as a white solid. ^1^H NMR (600 MHz, DMSO-*d*_6_) δ 10.39 (s, 1H), 8.36 (s, 1H), 7.93 (dd, *J* = 2.1, 1.8 Hz, 1H), 7.80 (ddd, *J* = 8.4, 2.1, 1.2 Hz, 1H), 7.56 (t, *J* = 7.8 Hz, 1H), 7.52 (d, *J* = 7.8 Hz, 2H), 7.40–7.32 (m, 3H), 7.25 (ddd, *J* = 7.8, 1.8, 1.2 Hz, 1H), 6.43 (dd, *J* = 16.8, 10.2 Hz, 1H), 6.26 (dd, *J* = 16.8, 1.8 Hz, 1H), 5.78 (dd, *J* = 10.2, 1.8 Hz, 1H), 4.89 (d, *J* = 7.2 Hz, 1H), 4.39–4.30 (m, 1H), 1.87–1.77 (m, 2H), 1.54–1.44 (m, 2H), 1.43–1.33 (m, 2H), 1.26–1.17 (m, 2H). ^13^C NMR (150 MHz, DMSO-*d*_6_) δ 164.4, 163.5, 156.6, 154.2, 145.4, 140.2, 132.2, 131.5, 130.4, 128.94, 128.89, 128.79, 127.5, 125.9, 124.5, 119.8, 119.6, 115.0, 102.3, 52.0, 32.5, 22.7. LRMS (ESI) *m*/*z*: 425.2 [M + H]^+^. HRMS (ESI) *m*/*z*: calcd for C_26_H_25_N_4_O_2_, 425.1978 [M + H]^+^; found, 425.1920. HPLC purity (method B): 97.8% (*t*_R_ = 27.60 min).

#### *N*-(3-(4-(Cyclohexylamino)-6-phenylfuro[2,3-*d*]pyrimidin-5-yl)phenyl)prop-2-enamide (**30**)

To a solution of **72g** (66 mg, 0.17 mmol, 1.0 equiv) in dichloromethane (0.9 mL) were added DIPEA (6 μL, 0.03 mmol, 20 mol %), acrylic acid (14 μL, 0.20 mmol, 1.2 equiv), and EDCI (49 mg, 0.26 mmol, 1.5 equiv); then, the reaction mixture was stirred at room temperature. After stirring for 16 h, the reaction mixture was concentrated *in vacuo* and purified by flash column chromatography (22% ethyl acetate in hexane) to yield the title compound **30** (43 mg, 0.10 mmol, 57%) as a white solid. ^1^H NMR (600 MHz, DMSO-*d*_6_) δ 10.39 (s, 1H), 8.34 (s, 1H), 7.95 (dd, *J* = 2.1, 1.8 Hz, 1H), 7.80 (ddd, *J* = 8.4, 2.1, 1.2 Hz, 1H), 7.57 (t, *J* = 7.8 Hz, 1H), 7.51 (d, *J* = 7.2 Hz, 2H), 7.40–7.31 (m, 3H), 7.26 (ddd, *J* = 7.8, 1.8, 1.2 Hz, 1H), 6.44 (dd, *J* = 16.8, 10.2 Hz, 1H), 6.26 (dd, *J* = 16.8, 1.8 Hz, 1H), 5.78 (dd, *J* = 10.2, 1.8 Hz, 1H), 4.93 (d, *J* = 7.8 Hz, 1H), 4.04–3.95 (m, 1H), 1.78–1.69 (m, 2H), 1.45–1.20 (m, 4H), 1.19–1.03 (m, 2H). ^13^C NMR (150 MHz, DMSO-*d*_6_) δ 164.5, 163.5, 156.3, 154.2, 145.5, 140.2, 132.1, 131.5, 130.4, 128.94, 128.87, 128.77, 127.4, 125.9, 124.5, 119.74, 119.66, 115.0, 102.1, 47.9, 31.3, 25.0, 23.1. LRMS (ESI) *m*/*z*: 439.2 [M + H]^+^. HRMS (ESI) *m*/*z*: calcd for C_27_H_26_N_4_NaO_2_, 461.1953 [M + Na]^+^; found, 461.1951. HPLC purity (method B): 99.5% (*t*_R_ = 28.82 min).

#### (2*E*)-4-(Dimethylamino)-*N*-(3-{4-[(2-hydroxyethyl)amino]-6-phenylfuro[2,3-*d*]pyrimidin-5-yl}phenyl)but-2-enamide (**31**)

To a solution of **64c** (100 mg, 0.29 mmol, 1.0 equiv) in dichloromethane (5.0 mL) were added 4-bromocrotonoic acid (71 mg, 0.43 mmol, 1.5 equiv) and EDCI (83 mg, 0.43 mmol, 1.5 equiv); then, the reaction mixture was stirred at room temperature. After stirring for 16 h, the reaction mixture was concentrated *in vacuo*, dissolved in THF (5.0 mL), and added 2 M *N*,*N*-dimethylamine (250 μL, 0.50 mmol, 1.7 equiv) in THF. Then, the reaction mixture was stirred at room temperature for 4 h, concentrated *in vacuo*, and purified by thin-layer chromatography (0–15% methanol in dichloromethane) to yield the title compound **31** (95 mg, 0.21 mmol, 72%) as a white solid. ^1^H NMR (600 MHz, DMSO-*d*_6_) δ 10.42 (s, 1H), 8.34 (s, 1H), 7.85 (d, *J* = 8.4 Hz, 1H), 7.82 (s, 1H), 7.51 (t, *J* = 7.8 Hz, 1H), 7.46 (d, *J* = 6.9 Hz, 2H), 7.36 (dd, *J* = 7.8, 6.9 Hz, 2H), 7.34–7.30 (m, 1H), 7.19 (d, *J* = 7.8 Hz, 1H), 6.73 (dt, *J* = 15.3, 5.7 Hz, 1H), 6.30 (d, *J* = 15.3 Hz, 1H), 5.50 (t, *J* = 5.4 Hz, 1H), 4.69 (s, 1H), 3.49–3.39 (m, 4H), 3.04 (d, *J* = 5.7 Hz, 2H), 2.16 (s, 6 H). ^13^C NMR (150 MHz, DMSO-*d*_6_) δ 164.5, 163.5, 157.2, 154.0, 145.5, 141.8, 131.7, 130.3, 129.0, 128.8, 128.7, 126.0, 125.7, 124.1, 119.9, 119.6, 115.3, 102.1, 59.7, 59.1, 45.1, 42.8. LRMS (ESI) *m*/*z*: 458.2 [M + H]^+^. HRMS (ESI) *m*/*z*: calcd for C_26_H_27_N_5_NaO_3_, 480.2012 [M + Na]^+^; found, 480.2008. HPLC purity (method A): 97.4% (*t*_R_ = 19.17 min).

#### (2*E*)-*N*-{3-[4-(Cyclopentylamino)-6-phenylfuro[2,3-*d*]pyrimidin-5-yl]phenyl}-4-(dimethylamino)but-2-enamide (**32**)

To a solution of **72f** (50 mg, 0.13 mmol, 1.0 equiv) in dichloromethane (3.0 mL) were added 4-bromocrotonoic acid (29 mg, 0.18 mmol, 1.3 equiv) and EDCI (83 mg, 0.43 mmol, 1.5 equiv); then, the reaction mixture was stirred at room temperature. After stirring for 16 h, to the reaction mixture was added ethyl acetate (5 mL) and washed with brine (10 mL). The combined organic layers were dried over MgSO_4_ and concentrated *in vacuo*. The residue was dissolved in THF (5.0 mL), cooled to 0 °C, and added 2 M *N*,*N*-dimethylamine (202 μL, 0.41 mmol, 3.0 equiv) in THF. The reaction mixture was stirred at room temperature for 5 h, concentrated *in vacuo*, and purified by flash column chromatography (3–9% methanol in dichloromethane) to yield the title compound **32** (23 mg, 0.05 mmol, 35%) as a yellow solid. ^1^H NMR (600 MHz, DMSO-*d*_6_) δ 8.35 (s, 1H), 7.86–7.81 (m, 3H), 7.59 (t, *J* = 8.4 Hz, 1H), 7.52 (d, *J* = 7.2 Hz, 2H), 7.40–7.29 (m, 5H), 6.38 (d, *J* = 8.4 Hz, 1H), 5.70 (t, *J* = 6.0 Hz, 1H), 4.84 (d, *J* = 7.2 Hz, 1H), 4.40–4.26 (m, 1H), 3.32 (s, 6H), 1.86–1.78 (m, 2H), 1.52–1.36 (m, 4H), 1.26–1.17 (m, 2H). ^13^C NMR (150 MHz, DMSO-*d*_6_) δ 173.6, 164.3, 156.7, 154.1, 145.4, 139.3, 131.9, 130.0, 129.0, 128.8, 128.7, 125.9, 125.8, 125.7, 122.3, 122.1, 114.9, 102.4, 52.0, 32.4, 32.3, 29.8, 28.0, 22.8. LRMS (ESI) *m*/*z*: 482.2 [M + H]^+^. HRMS (ESI) *m*/*z*: calcd for C_29_H_32_N_5_O_2_, 482.2556 [M + H]^+^; found, 482.2557. HPLC purity (method B): 91.6% (*t*_R_ = 16.99 min).

#### *N*-(3-{6-[4-(Dimethylamino)phenyl]-4-[(2-hydroxyethyl)amino]furo[2,3-*d*]pyrimidin-5-yl}phenyl)prop-2-enamide (**33**)

To a solution of **67c** (155 mg, 0.31 mmol, 1.0 equiv) in dichloromethane (15.0 mL) were added triethylamine (60 μL, 0.43 mmol, 1.4 equiv) and a solution of acryloyl chloride (30 μL, 0.37 mmol, 1.2 equiv) in dichloromethane (15.0 mL) dropwise at 0 °C, then the reaction mixture was stirred at room temperature. After stirring for 6 h, to the reaction mixture was added H_2_O (10 mL) and extracted with dichloromethane (10 mL × 3). The organic layers were combined, washed with brine (10 mL), dried over MgSO_4_, concentrated *in vacuo*, and purified by thin-layer chromatography (2% methanol in dichloromethane) to yield the TBS-protected compound **33′**. To the TBS-protected compound **33′** in dichloromethane (8.0 mL) was added trifluoroacetic acid (0.4 mL). The reaction mixture was stirred at room temperature for 6 h and then concentrated *in vacuo*. The obtained residue was neutralized with 10% NH_4_OH in methanol, then concentrated *in vacuo* again and purified by flash column chromatography (3% methanol in dichloromethane) to yield the title compound **33** (90 mg, 0.20 mmol, 66%) as a light-yellow solid. ^1^H NMR (600 MHz, DMSO-*d*_6_) δ 10.30 (s, 1H), 8.28 (s, 1H), 7.85 (d, *J* = 8.4 Hz, 1H), 7.76 (dd, *J* = 1.8, 1.8 Hz, 1H), 7.51 (dd, *J* = 8.4, 7.8 Hz, 1H), 7.29 (d, *J* = 9.0 Hz, 2H), 7.20 (ddd, *J* = 7.8, 1.8, 1.2 Hz, 1H), 6.65 (d, *J* = 9.0 Hz, 2H), 6.43 (dd, *J* = 17.1, 10.2 Hz, 1H), 6.26 (dd, *J* = 17.1, 2.1 Hz, 1H), 5.77 (dd, *J* = 10.2, 2.1 Hz, 1H), 5.39 (t, *J* = 5.4 Hz, 1H), 4.62 (t, *J* = 4.8 Hz, 1H), 3.50–3.40 (m, 4H), 2.90 (s, 6H). ^13^C NMR (150 MHz, DMSO-*d*_6_) δ 164.1, 163.4, 156.7, 152.9, 150.2, 147.0, 139.9, 132.5, 131.7, 130.2, 127.2, 127.1, 124.6, 120.3, 119.3, 116.1, 111.8, 102.4, 59.2, 42.8, 39.9. LRMS (ESI) *m*/*z*: 324.1 [M + H]^+^. HRMS (ESI) *m*/*z*: calcd for C_25_H_25_N_5_NaO_3_, 466.4968 [M + Na]^+^; found, 466.1994. HPLC purity (method A): 97.5% (*t*_R_ = 27.85 min).

#### *N*-(3-{4-[(2-Hydroxyethyl)amino]-6-[4-(morpholin-4-yl)phenyl]furo[2,3-*d*]pyrimidin-5-yl}phenyl)prop-2-enamide (**34**)

To a solution of **67d** (94 mg, 0.17 mmol, 1.0 equiv) in dichloromethane (8.0 mL) were added triethylamine (34 μL, 0.25 mmol, 1.4 equiv) and a solution of acryloyl chloride (17 μL, 0.21 mmol, 1.2 equiv) in dichloromethane (8.0 mL) dropwise at 0 °C, then the reaction mixture was stirred at room temperature. After stirring for 6 h, to the reaction mixture was added H_2_O (10 mL) and extracted with dichloromethane (10 mL × 3). The organic layers were combined, washed with brine (10 mL), dried over MgSO_4_, and concentrated *in vacuo* to yield the TBS-protected compound **34′**. To the TBS-protected compound **34′** in dichloromethane (4.0 mL) was added trifluoroacetic acid (0.2 mL); then, the reaction mixture was stirred at room temperature. After stirring for 12 h, the reaction mixture was concentrated *in vacuo*, neutralized with 10% NH_4_OH in methanol, then concentrated *in vacuo* again and purified by flash column chromatography (3% methanol in dichloromethane) to yield the title compound **34** (40 mg, 0.08 mmol, 48%) as a light-yellow solid. ^1^H NMR (600 MHz, DMSO-*d*_6_) δ 10.32 (s, 1H), 8.29 (s, 1H), 7.86 (ddd, *J* = 8.1, 2.4, 1.2 Hz, 1H), 7.77 (dd, *J* = 2.4, 1.8 Hz, 1H), 7.51 (dd, *J* = 8.1, 7.8 Hz, 1H), 7.32 (d, *J* = 9.0 Hz, 2H), 7.20 (ddd, *J* = 7.8, 1.8, 1.2 Hz, 1H), 6.90 (d, *J* = 9.0 Hz, 2H), 6.44 (dd, *J* = 16.8, 10.2 Hz, 1H), 6.26 (dd, *J* = 16.8, 2.1 Hz, 1H), 5.77 (dd, *J* = 10.2, 2.1 Hz, 1H), 5.42 (t, *J* = 5.4 Hz, 1H), 4.63 (t, *J* = 4.8 Hz, 1H), 3.74–3.66 (m, 4H), 3.49–3.40 (m, 4H), 3.18–3.10 (m, 4H). ^13^C NMR (150 MHz, DMSO-*d*_6_) δ 164.2, 163.4, 156.8, 153.2, 150.8, 146.4, 140.0, 132.3, 131.7, 130.2, 127.2, 127.0, 124.5, 120.2, 119.4, 119.0, 114.3, 112.4, 102.3, 65.9, 59.1, 47.3, 42.8. LRMS (ESI) *m*/*z*: 486.2 [M + H]^+^. HRMS (ESI) *m*/*z*: calcd for C_27_H_27_N_5_NaO_4_, 508.1961 [M + Na]^+^; found, 508.1961. HPLC purity (method A): 97.6% (*t*_R_ = 24.57 min).

#### *N*-(3-{4-[(2-Hydroxyethyl)amino)-6-[4-(4-methylpiperazin-1-yl)phenyl]furo[2,3-*d*]pyrimidin-5-yl}phenyl)prop-2-enamide (**35**)

To a solution of **67e** (140 mg, 0.25 mmol, 1.0 equiv) in dichloromethane (7.5 mL) were added triethylamine (40 μL, 0.29 mmol, 1.1 equiv) and a solution of acryloyl chloride (20 μL, 0.25 mmol, 1.0 equiv) in dichloromethane (7.5 mL) dropwise at 0 °C, then the reaction mixture was stirred at room temperature. After stirring for 3 h, to the reaction mixture was added H_2_O (10 mL) and extracted with dichloromethane (10 mL × 3). The organic layers were combined, washed with brine (10 mL), dried over MgSO_4_, concentrated *in vacuo*, and purified by flash column chromatography (2.5% methanol in dichloromethane with 0.3% NH_4_OH) to yield the TBS-protected compound **35′**. To the TBS-protected compound **35′** in dichloromethane (4.0 mL) was added trifluoroacetic acid (0.4 mL); then, the reaction mixture was stirred at room temperature. After stirring for 8 h, the reaction mixture was concentrated *in vacuo*, neutralized with 10% NH_4_OH in methanol, then concentrated *in vacuo* again and purified by flash column chromatography (5% methanol in dichloromethane with 0.5% NH_4_OH) to yield the title compound **35** (43 mg, 0.09 mmol, 34%) as a white solid. ^1^H NMR (600 MHz, DMSO-*d*_6_) δ 10.30 (s, 1H), 8.29 (s, 1H), 7.86 (dd, *J* = 8.4, 2.4 Hz, 1H), 7.76 (dd, *J* = 2.4, 1.8 Hz, 1H), 7.51 (dd, *J* = 8.4, 7.5 Hz, 1H), 7.30 (d, *J* = 9.0 Hz, 2H), 7.20 (ddd, *J* = 7.5, 1.8, 1.2 Hz, 1H), 6.88 (d, *J* = 9.0 Hz, 2H), 6.43 (dd, *J* = 17.1, 10.2 Hz, 1H), 6.26 (dd, *J* = 17.1, 1.8 Hz, 1H), 5.77 (dd, *J* = 10.2, 1.8 Hz, 1H), 5.41 (t, *J* = 5.4 Hz, 1H), 4.63 (s, 1H), 3.50–3.40 (m, 4H), 3.17 (dd, *J* = 4.8, 4.8 Hz, 4H), 2.39 (dd, *J* = 4.8, 4.8 Hz, 4H), 2.19 (s, 3H). ^13^C NMR (150 MHz, DMSO-*d*_6_) δ 164.2, 163.4, 156.8, 153.2, 150.7, 146.5, 140.0, 132.3, 131.7, 130.2, 127.2, 127.0, 124.5, 120.2, 119.4, 118.5, 114.5, 112.2, 102.3, 59.1, 54.3, 47.0, 45.7, 42.8. LRMS (ESI) *m*/*z*: 499.2 [M + H]^+^. HRMS (ESI) *m*/*z*: calcd for C_28_H_31_N_6_O_3_, 499.2458 [M + H]^+^; found, 499.2460. HPLC purity (method A): 99.2% (*t*_R_ = 15.46 min).

#### *N*-{3-[6-(4-{[2-(Dimethylamino)ethyl](methyl)amino}phenyl)-4-[(2-hydroxyethyl)amino]furo[2,3-*d*]pyrimidin-5-yl]phenyl}prop-2-enamide (**36**)

To a solution of **72h** (125 mg, 0.28 mmol, 1.0 equiv) in dichloromethane (3.0 mL) were added acrylic acid (21 μL, 0.31 mmol, 1.1 equiv) and EDCI (80 mg, 0.42 mmol, 1.5 equiv); then, the reaction mixture was stirred at room temperature. After stirring for 16 h, the reaction mixture was quenched with NaHCO_3(aq)_ (10 mL) and extracted with ethyl acetate (10 mL × 3). The combined organic layers were dried over MgSO_4_, concentrated *in vacuo*, and purified by thin-layer chromatography (5% methanol in dichloromethane with 0.1% NH_4_OH) to yield the title compound **36** (40 mg, 0.08 mmol, 29%) as a yellow solid. ^1^H NMR (400 MHz, DMSO-*d*_6_) δ 8.33 (s, 1H), 7.80 (s, 1H), 7.73 (s, 1H), 7.58 (d, *J* = 8.0 Hz, 1H), 7.44 (dd, *J* = 8.0, 8.0 Hz, 1H), 7.38 (d, *J* = 8.8 Hz, 2H), 7.28–7.24 (m, 1H), 6.55 (d, *J* = 9.2 Hz, 2H), 6.47 (dd, *J* = 16.8, 1.4 Hz, 1H), 6.28 (dd, *J* = 16.8, 10.4 Hz, 1H), 5.82 (dd, *J* = 10.4, 1.4 Hz, 1H), 5.46 (t, *J* = 5.0 Hz, 1H), 3.76 (dd, *J* = 5.8, 5.0 Hz, 2H), 3.63 (s, 1H), 3.43 (dd, *J* = 7.6, 7.4 Hz, 2H), 2.95 (s, 3H), 2.44 (dd, *J* = 7.6, 7.4 Hz, 2H), 2.27 (s, 6H), 0.88 (dd, *J* = 6.8, 5.8 Hz, 2H). ^13^C NMR (150 MHz, DMSO-*d*_6_) δ 164.1, 163.4, 156.7, 152.9, 149.0, 147.0, 140.0, 132.6, 131.7, 130.2, 127.3, 127.2, 124.6, 120.3, 119.3, 115.7, 111.4, 111.2, 102.5, 59.2, 55.6, 49.5, 45.5, 42.8, 30.9. LRMS (ESI) *m*/*z*: 501.2 [M + H]^+^. HRMS (ESI) *m*/*z*: calcd for C_28_H_32_N_6_NaO_3_, 523.2434 [M + Na]^+^; found, 523.2434. HPLC purity (method B): 89.6% (*t*_R_ = 12.57 min).

#### *N*-{3-[4-(Cyclopentylamino)-6-[4-(morpholin-4-yl)phenyl]furo[2,3-*d*]pyrimidin-5-yl]phenyl}prop-2-enamide (**37**)

To a solution of **72i** (84 mg, 0.18 mmol, 1.0 equiv) in dichloromethane (3.0 mL) were added DIPEA (6 μL, 0.03 mmol, 19 mol %), acrylic acid (16 μL, 0.24 mmol, 1.3 equiv), and EDCI (42 mg, 0.22 mmol, 1.2 equiv); then, the reaction mixture was stirred at room temperature. After stirring for 16 h, to the reaction mixture was added ethyl acetate (5 mL) and washed with NaHCO_3(aq)_ (10 mL) and brine (10 mL). The combined organic layers were dried over MgSO_4_, concentrated *in vacuo*, and purified by flash column chromatography (25–66% ethyl acetate in hexane) to yield the title compound **37** (45 mg, 0.09 mmol, 48%) as a yellow solid. ^1^H NMR (600 MHz, DMSO-*d*_6_) δ 10.37 (s, 1H), 8.30 (s, 1H), 7.89 (dd, *J* = 2.1, 1.8 Hz, 1H), 7.79 (dd, *J* = 7.8, 2.1 Hz, 1H), 7.55 (dd, *J* = 7.8, 7.8 Hz, 1H), 7.37 (d, *J* = 9.0 Hz, 2H), 7.24–7.21 (m, 1H), 6.92 (d, *J* = 9.0 Hz, 2H), 6.43 (dd, *J* = 16.8, 10.2 Hz, 1H), 6.27 (dd, *J* = 16.8, 2.1 Hz, 1H), 5.78 (dd, *J* = 10.2, 2.1 Hz, 1H), 4.83 (d, *J* = 7.2 Hz, 1H), 4.33 (dquin, *J* = 7.2, 4.8 Hz, 1H), 3.70 (dd, *J* = 12.0, 4.2 Hz, 4H), 3.15 (dd, *J* = 12.0, 3.6 Hz, 4H), 1.86–1.77 (m, 2H), 1.53–1.33 (m, 4H), 1.26–1.15 (m, 2H). ^13^C NMR (150 MHz, DMSO-*d*_6_) δ 164.1, 163.4, 156.2, 153.4, 150.9, 146.2, 140.1, 132.6, 131.6, 130.3, 127.4, 126.9, 124.6, 119.9, 119.4, 119.0, 114.4, 112.2, 102.4, 65.9, 51.9, 47.3, 32.5, 22.7. LRMS (ESI) *m*/*z*: 510.2 [M + H]^+^. HRMS (ESI) m/z: calcd for C_30_H_31_N_5_NaO_3_, 532.2325 [M + Na]+; found, 532.2318. HPLC purity (method B): 99.7% (*t*_R_ = 26.25 min).

#### *N*-{3-[4-(Cyclopentylamino)-6-[4-(4-methylpiperazin-1-yl)phenyl]furo[2,3-*d*]pyrimidin-5-yl]phenyl}prop-2-enamide (**38**)

To a solution of **72j** (40 mg, 0.09 mmol, 1.0 equiv) in dichloromethane (5 mL) at 0 °C were added acrylic acid (9 μL, 0.13 mmol, 1.5 equiv) and EDCI (25 mg, 0.42 mmol, 1.5 equiv); then, the reaction mixture was stirred at room temperature. After stirring for 16 h, the reaction mixture was quenched with NaHCO_3(aq)_ (10 mL) and extracted with dichloromethane (10 mL × 3). The combined organic layers were dried over MgSO_4_, concentrated *in vacuo*, and purified by flash column chromatography (5% methanol in dichloromethane with 0.1% NH_4_OH) to yield the title compound **38** (35 mg, 0.07 mmol, 78%) as a yellow solid. ^1^H NMR (600 MHz, DMSO-*d*_6_) δ 10.37 (s, 1H), 8.30 (s, 1H), 7.89 (dd, *J* = 2.1, 1.8 Hz, 1H), 7.79 (dd, *J* = 8.1, 2.1, 1.2 Hz, 1H), 7.54 (dd, *J* = 8.1, 7.5 Hz, 1H), 7.35 (d, *J* = 9.0 Hz, 2H), 7.22 (ddd, *J* = 7.5, 1.8, 1.2 Hz 1H), 6.90 (d, *J* = 9.0 Hz, 2H), 6.44 (dd, *J* = 16.8, 10.2 Hz, 1H), 6.27 (dd, *J* = 16.8, 2.1 Hz, 1H), 5.78 (dd, *J* = 10.2, 2.1 Hz, 1H), 4.82 (d, *J* = 6.9 Hz, 1H), 4.33 (dquin, *J* = 6.9, 5.4 Hz, 1H), 3.18 (dd, *J* = 5.4, 5.1 Hz, 4H), 2.40 (dd, *J* = 5.1, 4.8 Hz, 4H), 2.19 (s, 3H), 1.86–1.77 (m, 2H), 1.52–1.32 (m, 4H), 1.25–1.16 (m, 2H). ^13^C NMR (150 MHz, DMSO-*d*_6_) δ 164.1, 163.4, 156.2, 153.3, 150.8, 146.3, 140.1, 132.7, 131.6, 130.2, 127.4, 126.9, 124.6, 119.9, 119.4, 118.5, 114.6, 112.0, 102.4, 54.3, 51.9, 47.0, 45.7, 32.5, 22.7. LRMS (ESI) *m*/*z*: 523.3 [M + H]^+^. HRMS (ESI) *m*/*z*: calcd for C_31_H_35_N_6_O_2_, 523.2822 [M + H]+; found, 523.2836. HPLC purity (method B): 96.9% (*t*_R_ = 16.13 min).

#### *N*-(3-{4-[((1S)-2-Hydroxy-1-phenylethyl)amino]-6-[4-(4-methylpiperazin-1-yl)phenyl]furo[2,3-*d*]pyrimidin-5-yl}phenyl)prop-2-enamide (**39**)

To a solution of **72k** (100 mg, 0.19 mmol, 1.0 equiv) in dichloromethane (2 mL) at 0 °C were added acrylic acid (16 μL, 0.23 mmol, 1.2 equiv) and EDCI (51 mg, 0.27 mmol, 1.4 equiv); then, the reaction mixture was stirred at room temperature. After stirring for 16 h, the reaction mixture was quenched with NaHCO_3(aq)_ (10 mL) and extracted with dichloromethane (10 mL × 3). The combined organic layers were dried over MgSO_4_, concentrated *in vacuo*, and purified by thin-plate chromatography (5% methanol in dichloromethane with 0.1% NH_4_OH) to yield the title compound **39** (35 mg, 0.06 mmol, 32%) as a light-yellow solid. ^1^H NMR (400 MHz, DMSO-*d*_6_) δ 10.36 (s, 1H), 8.23 (s, 1H), 7.94 (s, 1H), 7.85 (d, *J* = 8.0 Hz, 1H), 7.56 (dd, *J* = 8.0, 7.6 Hz, 1H), 7.40–7.10 (m, 8H), 6.92 (d, *J* = 8.8 Hz, 2H), 6.45 (dd, *J* = 17.0, 10.2 Hz, 1H), 6.28 (dd, *J* = 17.0, 2.0 Hz, 1H), 5.78 (dd, *J* = 10.2, 2.0 Hz, 1H), 5.66 (d, *J* = 7.6 Hz, 1H), 5.24–5.16 (m, 1H), 4.82 (t, *J* = 5.2 Hz, 1H), 3.62–3.54 (m, 1H), 3.47–3.38 (m, 1H), 3.32–3.14 (m, 4H), 2.44–2.36 (m, 4H), 2.20 (s, 3H). ^13^C NMR (150 MHz, DMSO-*d*_6_) δ 164.2, 163.4, 156.2, 153.2, 150.8, 146.6, 140.8, 140.2, 132.5, 131.7, 130.4, 128.1, 127.3, 126.9, 126.8, 126.5, 124.5, 119.9, 119.3, 118.5, 114.6, 112.1, 102.8, 64.4, 55.3, 54.3, 47.0, 45.7. LRMS (ESI) *m*/*z*: 575.3 [M + H]^+^. HRMS (ESI) *m*/*z*: calcd for C_34_H_34_N_6_NaO_3_, 597.2590 [M + Na]^+^; found, 597.2593. HPLC purity (method B): 83.7% (*t*_R_ = 15.16 min).

#### *N*-{3-[4-(Cyclohexylamino)-6-[4-(4-methylpiperazin-1-yl)phenyl]furo[2,3-*d*]pyrimidin-5-yl]phenyl}prop-2-enamide (**40**)

To a solution of **72l** (130 mg, 0.27 mmol, 1.0 equiv) in dichloromethane (5 mL) at 0 °C were added acrylic acid (28 μL, 0.41 mmol, 1.5 equiv) and EDCI (77 mg, 0.40 mmol, 1.5 equiv); then, the reaction mixture was stirred at room temperature. After stirring for 16 h, the reaction mixture was quenched with NaHCO_3(aq)_ (10 mL) and extracted with dichloromethane (10 mL × 3). The combined organic layers were dried over MgSO_4_, concentrated *in vacuo*, and purified by flash column chromatography (5% methanol in dichloromethane with 0.1% NH_4_OH) to yield the title compound **40** (67 mg, 0.12 mmol, 46%) as a yellow solid. ^1^H NMR (600 MHz, DMSO-*d*_6_) δ 10.37 (s, 1H), 8.28 (s, 1H), 7.91 (dd, *J* = 2.4, 1.8 Hz, 1H), 7.80 (dd, *J* = 8.1, 1.8 Hz, 1H), 7.55 (dd, *J* = 8.1, 7.8 Hz, 1H), 7.34 (d, *J* = 9.0 Hz, 2H), 7.22 (d, *J* = 7.8 Hz 1H), 6.90 (d, *J* = 9.0 Hz, 2H), 6.44 (dd, *J* = 16.8, 10.2 Hz, 1H), 6.27 (dd, *J* = 16.8, 2.1 Hz, 1H), 5.77 (dd, *J* = 10.2, 2.1 Hz, 1H), 4.86 (d, *J* = 7.8 Hz, 1H), 4.02–3.92 (m, 1H), 3.17 (dd, *J* = 5.4, 4.8 Hz, 4H), 2.40 (dd, *J* = 5.4, 4.8 Hz, 4H), 2.19 (s, 3H), 1.77–1.69 (m, 2H), 1.45–1.22 (m, 5H), 1.17–1.02 (m, 3H). ^13^C NMR (150 MHz, DMSO-*d*_6_) δ 164.1, 163.4, 156.0, 153.4, 150.8, 146.4, 140.1, 132.7, 131.6, 130.3, 127.4, 126.9, 124.6, 119.9, 119.4, 118.5, 114.5, 112.0, 102.3, 54.3, 47.8, 47.0, 45.7, 31.4, 25.0, 23.1. LRMS (ESI) *m*/*z*: 537.2 [M + H]^+^. HRMS (ESI) *m*/*z*: calcd for C_32_H_37_N_6_O_2_, 537.2978 [M + H]^+^; found, 537.2941. HPLC purity (method B): 94.2% (*t*_R_ = 17.49 min).

#### *N*-{3-[4-(Methylamino)-6-[4-(4-methylpiperazin-1-yl)phenyl]furo[2,3-*d*]pyrimidin-5-yl]phenyl}prop-2-enamide (**41**)

To a solution of **72m** (58 mg, 0.14 mmol, 1.0 equiv) in dichloromethane (1 mL) were added acrylic acid (12 μL, 0.18 mmol, 1.3 equiv) and EDCI (40 mg, 0.21 mmol, 1.5 equiv); then, the reaction mixture was stirred at room temperature. After stirring for 1 h, the reaction mixture was quenched with NaHCO_3(aq)_ (10 mL) and extracted with ethyl acetate (10 mL × 3). The combined organic layers were dried over MgSO_4_, concentrated *in vacuo*, and purified by flash column chromatography (5% methanol in dichloromethane) to yield the title compound **41** (27 mg, 0.06 mmol, 41%) as colorless oil. ^1^H NMR (600 MHz, DMSO-*d*_6_) δ 10.34 (s, 1H), 8.31 (s, 1H), 7.84 (ddd, *J* = 8.1, 1.8, 1.2 Hz, 1H), 7.72 (dd, *J* = 1.8, 1.8 Hz, 1H), 7.50 (dd, *J* = 8.1, 7.8 Hz, 1H), 7.27 (d, *J* = 9.0 Hz, 2H), 7.15 (d, *J* = 7.8, 1.8, 1.2 Hz 1H), 6.88 (d, *J* = 9.0 Hz, 2H), 6.44 (dd, *J* = 16.8, 10.2 Hz, 1H), 6.27 (dd, *J* = 16.8, 1.8 Hz, 1H), 5.77 (dd, *J* = 10.2, 1.8 Hz, 1H), 5.42 (q, *J* = 4.8 Hz, 1H), 3.17 (dd, *J* = 5.4, 4.8 Hz, 4H), 2.87 (d, *J* = 4.8 Hz, 3H), 2.39 (dd, *J* = 5.4, 4.8 Hz, 4H), 2.19 (s, 3H). ^13^C NMR (150 MHz, DMSO-*d*_6_) δ 164.1, 163.4, 157.3, 153.2, 150.7, 146.4, 139.9, 132.3, 131.7, 130.2, 127.2, 127.0, 124.8, 120.4, 119.6, 118.5, 114.4, 112.4, 102.3, 54.3, 46.9, 45.7, 27.9. LRMS (ESI) *m*/*z*: 469.2 [M + H]^+^. HRMS (ESI) *m*/*z*: calcd for C_27_H_28_N_6_NaO_2_, 491.2171 [M + Na]^+^; found, 491.2173. HPLC purity (method B): 99.4% (*t*_R_ = 13.31 min).

#### *N*-{3-[4-(Cyclopentylamino)-6-(pyridin-4-yl)furo[2,3-*d*]pyrimidin-5-yl]phenyl}prop-2-enamide (**42**)

To a solution of **72n** (71 mg, 0.19 mmol, 1.0 equiv) in dichloromethane (5 mL) were added acrylic acid (19 μL, 0.28 mmol, 1.4 equiv) and EDCI (52 mg, 0.27 mmol, 1.4 equiv); then, the reaction mixture was stirred at room temperature. After stirring for 16 h, the reaction mixture was quenched with NaHCO_3(aq)_ (10 mL) and extracted with dichloromethane (10 mL × 3). The combined organic layers were dried over MgSO_4_, concentrated *in vacuo*, and purified by flash column chromatography (5% methanol in dichloromethane with 0.1% NH_4_OH) to yield the title compound **42** (70 mg, 0.16 mmol, 86%) as a yellow solid. ^1^H NMR (600 MHz, DMSO-*d*_6_) δ 10.43 (s, 1H), 8.55 (d, *J* = 6.0 Hz, 2H), 8.41 (s, 1H), 7.97 (dd, *J* = 2.4, 1.8 Hz, 1H), 7.82 (ddd, *J* = 8.1, 2.4, 1.2 Hz, 1H), 7.61 (dd, *J* = 8.1, 7.8 Hz, 1H), 7.41 (d, *J* = 6.0 Hz, 2H), 7.30 (ddd, *J* = 7.8, 1.8, 1.2 Hz 1H), 6.44 (dd, *J* = 16.8, 10.2 Hz, 1H), 6.27 (dd, *J* = 16.8, 1.8 Hz, 1H), 5.79 (dd, *J* = 10.2, 1.8 Hz, 1H), 4.97 (d, *J* = 6.9 Hz, 1H), 4.35 (dquin, *J* = 6.9, 4.8 Hz, 1H), 1.88–1.77 (m, 2H), 1.53–1.33 (m, 4H), 1.27–1.17 (m, 2H). ^13^C NMR (150 MHz, DMSO-*d*_6_) δ 164.7, 163.5, 156.9, 155.2, 150.3, 142.6, 140.3, 135.9, 131.5, 131.3, 130.6, 127.5, 124.2, 120.1, 119.5, 119.2, 119.0, 102.2, 52.1, 32.4, 22.7. LRMS (ESI) *m*/*z*: 426.2 [M + H]^+^. HRMS (ESI) *m*/*z*: calcd for C_25_H_23_N_5_NaO_2_, 448.1749 [M + Na]^+^; found, 448.1756. HPLC purity (method B): 96.9% (*t*_R_ = 23.20 min).

#### *N*-{3-[4-(Cyclopentylamino)-6-[6-(morpholin-4-yl)pyridin-3-yl]furo[2,3-*d*]pyrimidin-5-yl]phenyl}prop-2-enamide (**43**)

To a solution of **72o** (60 mg, 0.13 mmol, 1.0 equiv) in dichloromethane (0.7 mL) were added acrylic acid (12 μL, 0.18 mmol, 1.3 equiv) and EDCI (42 mg, 0.22 mmol, 1.7 equiv); then, the reaction mixture was stirred at room temperature. After stirring for 2 h, the reaction mixture was concentrated *in vacuo* and purified by flash column chromatography (5% methanol in dichloromethane) to yield the title compound **43** (17 mg, 0.03 mmol, 25%) as a yellow solid. ^1^H NMR (600 MHz, DMSO-*d*_6_) δ 10.38 (s, 1H), 8.32 (s, 1H), 8.24 (dd, *J* = 2.1, 0.9 Hz, 1H), 7.92 (dd, *J* = 2.1, 1.8 Hz, 1H), 7.70 (ddd, *J* = 8.1, 2.1, 1.2 Hz, 1H), 7.60 (dd, *J* = 9.3, 2.4 Hz, 1H), 7.55 (dd, *J* = 8.1, 7.8 Hz, 1H), 7.23 (ddd, *J* = 7.8, 1.8, 1.2 Hz, 1H), 6.85 (dd, *J* = 9.3, 0.9 Hz, 1H), 6.43 (dd, *J* = 16.8, 10.2 Hz, 1H), 6.27 (dd, *J* = 16.8, 1.8 Hz, 1H), 5.78 (dd, *J* = 10.2, 1.8 Hz, 1H), 4.90 (d, *J* = 6.9 Hz, 1H), 4.34 (dquin, *J* = 6.9, 4.8 Hz, 1H), 3.65 (dd, *J* = 6.0, 5.1 Hz, 4H), 3.48 (dd, *J* = 6.0, 5.1 Hz, 4H), 1.87–1.77 (m, 2H), 1.54–1.36 (m, 4H), 1.26–1.18 (m, 2H). ^13^C NMR (150 MHz, DMSO-*d*_6_) δ 164.3, 163.4, 158.2, 156.3, 153.6, 145.5, 144.6, 140.1, 134.9, 132.1, 131.5, 130.3, 127.4, 124.5, 119.9, 119.5, 114.5, 112.8, 106.6, 102.1, 65.8, 52.0, 44.6, 32.5, 22.7. LRMS (ESI) *m*/*z*: 511.3 [M + H]^+^. HRMS (ESI) *m*/*z*: calcd for C_29_H_30_N_6_NaO_3_, 533.2277 [M + Na]^+^; found, 533.2278. HPLC purity (method B): 99.8% (*t*_R_ = 24.56 min).

#### *N*-{3-[4-(Cyclopentylamino)-6-[6-(4-methylpiperazin-1-yl)pyridin-3-yl]furo[2,3-*d*]pyrimidin-5-yl]phenyl}prop-2-enamide (**44**)

To a solution of **72p** (75 mg, 0.16 mmol, 1.0 equiv) in dichloromethane (0.8 mL) were added acrylic acid (13 μL, 0.19 mmol, 1.2 equiv) and EDCI (46 mg, 0.24 mmol, 1.5 equiv); then, the reaction mixture was stirred at room temperature. After stirring for 2 h, the reaction mixture was concentrated *in vacuo*, dissolved in ethyl acetate, and washed with NaHCO_3(aq)_ (10 mL) and brine (10 mL). The combined organic layers were dried over MgSO_4_, concentrated *in vacuo*, and purified by flash column chromatography (5% methanol in dichloromethane) to yield the title compound **44** (12 mg, 0.02 mmol, 14%) as a yellow solid. ^1^H NMR (600 MHz, DMSO-*d*_6_) δ 10.38 (s, 1H), 8.31 (s, 1H), 8.22 (d, *J* = 2.4 Hz, 1H), 7.91 (dd, *J* = 1.8, 1.8 Hz, 1H), 7.77 (dd, *J* = 8.4, 1.8 Hz, 1H), 7.57–7.53 (m, 2H), 7.24–7.21 (m, 1H), 6.84 (d, *J* = 9.0 Hz, 1H), 6.43 (dd, *J* = 17.1, 10.2 Hz, 1H), 6.27 (dd, *J* = 17.1, 1.8 Hz, 1H), 5.78 (dd, *J* = 10.2, 1.8 Hz, 1H), 4.89 (d, *J* = 7.2 Hz, 1H), 4.34 (dquin, *J* = 7.2, 4.8 Hz, 1H), 3.51 (dd, *J* = 5.1, 4.8 Hz, 4H), 2.35 (dd, *J* = 5.1, 4.8 Hz, 4H), 2.19 (s, 3H), 1.87–1.77 (m, 2H), 1.53–1.35 (m, 4H), 1.26–1.17 (m, 2H). ^13^C NMR (150 MHz, DMSO-*d*_6_) δ 164.3, 163.5, 158.0, 156.2, 153.5, 145.5, 144.7, 140.1, 134.8, 132.2, 131.6, 130.3, 127.4, 124.5, 119.9, 119.5, 114.0, 112.6, 106.6, 102.2, 54.2, 52.0, 45.7, 44.1, 32.5, 22.7. LRMS (ESI) *m*/*z*: 524.3 [M + H]^+^. HRMS (ESI) *m*/*z*: calcd for C_30_H_33_N_7_NaO_2_, 546.2593 [M + Na]^+^; found, 546.2593. HPLC purity (method B): 95.7% (*t*_R_ = 15.56 min).

#### *N*-{5-[4-(Cyclopentylamino)-6-phenylfuro[2,3-*d*]pyrimidin-5-yl]-2-(morpholin-4-yl)phenyl}prop-2-enamide (**45**)

To a solution of **64j** (47 mg, 0.10 mmol, 1.0 equiv) in dichloromethane (3.0 mL) were added DIPEA (4 μL, 0.02 mmol, 22 mol %), acrylic acid (9 μL, 0.14 mmol, 1.3 equiv), and EDCI (24 mg, 0.13 mmol, 1.2 equiv); then, the reaction mixture was stirred at room temperature. After stirring for 16 h, to the reaction mixture was added ethyl acetate (5 mL) and washed with NaHCO_3(aq)_ (10 mL) and brine (10 mL). The combined organic layers were dried over MgSO_4_, concentrated *in vacuo*, and purified by flash column chromatography (33–50% ethyl acetate in hexane) to yield the title compound **45** (32 mg, 0.06 mmol, 61%) as a white solid. ^1^H NMR (600 MHz, DMSO-*d*_6_) δ 9.35 (s, 1H), 8.34 (s, 1H), 8.31 (d, *J* = 1.8 Hz, 1H), 8.17 (s, 1H), 7.56–7.53 (m, 3H), 7.40–7.31 (m, 4H), 7.26 (dd, *J* = 8.4, 1.8 Hz, 1H), 6.71 (dd, *J* = 16.8, 10.2 Hz, 1H), 6.24 (dd, *J* = 16.8, 1.8 Hz, 1H), 5.78 (dd, *J* = 10.2, 1.8 Hz, 1H), 4.92 (d, *J* = 6.9 Hz, 1H), 4.34 (dquin, *J* = 6.9, 4.8 Hz, 1H), 3.85 (dd, *J* = 4.8, 4.2 Hz, 4H), 2.91 (dd, *J* = 4.8, 4.2 Hz, 4H), 1.84–1.76 (m, 2H), 1.52–1.43 (m, 2H), 1.42–1.32 (m, 2H), 1.31–1.23 (m, 2H). ^13^C NMR (150 MHz, DMSO-*d*_6_) δ 164.3, 163.4, 156.6, 154.1, 145.3, 132.7, 131.8, 129.1, 128.9, 128.7, 127.3, 126.5, 126.1, 125.8, 123.1, 121.0, 114.9, 102.5, 79.2, 66.0, 54.9, 52.0, 51.5, 32.3, 22.7. LRMS (ESI) *m*/*z*: 510.2 [M + H]^+^. HRMS (ESI) *m*/*z*: calcd for C_30_H_32_N_5_O_3_, 510.2505 [M + H]^+^; found, 510.2457. HPLC purity (method B): 98.6% (*t*_R_ = 25.93 min).

#### *N*-{5-[4-(Cyclopentylamino)-6-phenylfuro[2,3-*d*]pyrimidin-5-yl]-2-(4-methylpiperazin-1-yl)phenyl}prop-2-enamide (**46**)

To a solution of **64k** (51 mg, 0.10 mmol, 1.0 equiv) in dichloromethane (5 mL) were added acrylic acid (8 μL, 0.11 mmol, 1.1 equiv) and EDCI (31 mg, 0.16 mmol, 1.6 equiv); then, the reaction mixture was stirred at room temperature. After stirring for 16 h, the reaction mixture was concentrated *in vacuo* and purified by thin-plate chromatography (5% methanol in dichloromethane with 0.1% NH_4_OH) to yield the title compound **46** (18 mg, 0.03 mmol, 34%) as a yellow solid. ^1^H NMR (600 MHz, DMSO-*d*_6_) δ 9.24 (s, 1H), 8.34 (s, 1H), 8.12 (s, 1H), 7.55 (d, *J* = 7.2 Hz, 2H), 7.40–7.30 (m, 4H), 7.24 (dd, *J* = 8.4, 2.4 Hz, 1H), 6.67 (dd, *J* = 16.8, 10.2 Hz, 1H), 6.23 (dd, *J* = 16.8, 1.8 Hz, 1H), 5.77 (dd, *J* = 10.2, 1.8 Hz, 1H), 4.91 (d, *J* = 6.9 Hz, 1H), 4.34 (dquin, *J* = 6.9, 2.4 Hz, 1H), 2.92 (s, 4H), 2.58 (s, 4H), 2.27 (s, 3H), 1.84–1.75 (m, 2H), 1.52–1.22 (m, 6H). ^13^C NMR (150 MHz, DMSO-*d*_6_) δ 164.3, 163.3, 156.6, 154.1, 145.3, 144.2, 132.5, 131.8, 129.1, 128.8, 128.7, 127.2, 126.2, 126.0, 125.8, 123.1, 120.9, 114.9, 102.6, 54.5, 52.0, 50.9, 45.7, 32.4, 22.7. LRMS (ESI) *m*/*z*: 523.4 [M + H]^+^. HRMS (ESI) *m*/*z*: calcd for C_31_H_35_N_6_O_2_, 523.2822 [M + H]^+^; found, 523.2829. HPLC purity (method B): 98.6% (*t*_R_ = 16.83 min).

#### *N*-{5-[4-(Cyclopentylamino)-6-phenylfuro[2,3-*d*]pyrimidin-5-yl]-2-[2-(dimethylamino)ethoxy]phenyl}prop-2-enamide (**47**)

To a solution of **64l** (16 mg, 0.03 mmol, 1.0 equiv) in dichloromethane (5 mL) were added acrylic acid (4 μL, 0.06 mmol, 1.7 equiv) and EDCI (10 mg, 0.05 mmol, 1.5 equiv); then, the reaction mixture was stirred at room temperature. After stirring for 16 h, to the reaction mixture were added water (15 mL), dichloromethane (5 mL), and sat. NaHCO_3(aq)_ (2 mL) and extracted with dichloromethane (10 mL × 3). The combined organic layers were washed with brine (10 mL), dried over MgSO_4_, concentrated *in vacuo*, and purified by flash column chromatography (5% methanol in dichloromethane with 0.1% NH_4_OH) to yield the title compound **47** (14 mg, 0.03 mmol, 78%) as a yellow oil. ^1^H NMR (600 MHz, DMSO-*d*_6_) δ 9.76 (s, 1H), 8.34 (s, 1H), 8.31 (d, *J* = 2.4 Hz, 1H), 7.54 (d, *J* = 7.5 Hz, 2H), 7.37 (dd, *J* = 8.4, 7.5 Hz, 2H), 7.35–7.30 (m, 2H), 7.21 (dd, *J* = 8.4, 2.4 Hz, 1H), 6.60 (dd, *J* = 16.8, 10.2 Hz, 1H), 6.23 (dd, *J* = 16.8, 1.8 Hz, 1H), 5.77 (dd, *J* = 10.2, 1.8 Hz, 1H), 4.99 (d, *J* = 7.2 Hz, 1H), 4.35 (dquin, *J* = 7.2, 5.4 Hz, 1H), 4.25 (t, *J* = 6.0 Hz, 2H), 2.69 (t, *J* = 6.0 Hz, 2H), 2.27 (s, 3H), 1.87–1.77 (m, 2H), 1.52–1.36 (m, 4H), 1.30–1.22 (m, 2H). ^13^C NMR (150 MHz, DMSO-*d*_6_) δ 164.3, 163.4, 156.7, 154.1, 148.8, 145.4, 131.7, 129.5, 129.1, 128.8, 128.6, 127.2, 125.83, 125.76, 123.9, 122.1, 115.5, 114.9, 102.4, 67.9, 57.2, 52.0, 45.2, 32.4, 22.8. LRMS (ESI) *m*/*z*: 512.3 [M + H]^+^. HRMS (ESI) *m*/*z*: calcd for C_30_H_34_N_5_O_3_, 512.2662 [M + H]^+^; found, 512.2658. HPLC purity (method B): 99.0% (*t*_R_ = 16.14 min).

#### *N*-{5-[4-(Cyclopentylamino)-6-phenylfuro[2,3-*d*]pyrimidin-5-yl)-2-[(1-methylpiperidin-4-yl)oxy]phenyl]prop-2-enamide (**48**)

To a solution of **64m** (16 mg, 0.03 mmol, 1.0 equiv) in dichloromethane (5 mL) were added acrylic acid (3 μL, 0.04 mmol, 1.3 equiv) and EDCI (10 mg, 0.05 mmol, 1.6 equiv); then, the reaction mixture was stirred at room temperature. After stirring for 16 h, to the reaction mixture were added water (15 mL), dichloromethane (5 mL), and sat. NaHCO_3(aq)_ (2 mL) and extracted with dichloromethane (10 mL × 3). The combined organic layers were washed with brine (10 mL), dried over MgSO_4_, concentrated *in vacuo*, and purified by flash column chromatography (5% methanol in dichloromethane with 0.1% NH_4_OH) to yield the title compound **48** (9 mg, 0.02 mmol, 51%) as a yellow oil. ^1^H NMR (600 MHz, DMSO-*d*_6_) δ 9.36 (s, 1H), 8.34 (s, 1H), 8.21 (s, 1H), 7.55 (d, *J* = 7.2 Hz, 2H), 7.37 (dd, *J* = 7.8, 7.2 Hz, 2H), 7.35–7.30 (m, 2H), 7.20 (dd, *J* = 8.4, 2.4 Hz, 1H), 6.72 (dd, *J* = 17.4, 10.2 Hz, 1H), 6.22 (dd, *J* = 17.4, 2.1 Hz, 1H), 5.76 (dd, *J* = 10.2, 2.1 Hz, 1H), 4.95 (d, *J* = 7.2 Hz, 1H), 4.53 (dquin, *J* = 8.4, 4.8 Hz, 1H), 4.38–4.33 (m, 1H), 2.66 (s, 1H), 2.18 (s, 3H), 2.00–1.92 (m, 2H), 1.86–1.75 (m, 4H), 1.53–1.32 (m, 6H), 1.30–1.20 (m, 2H). ^13^C NMR (150 MHz, DMSO-*d*_6_) δ 164.3, 163.5, 156.7, 154.1, 147.8, 145.3, 131.8, 129.2, 129.1, 128.8, 128.6, 127.1, 125.8, 125.7, 123.2, 123.0, 115.5, 114.9, 102.6, 54.9, 52.4, 51.9, 45.8, 32.4, 30.3, 22.8. LRMS (ESI) *m*/*z*: 538.3 [M + H]^+^. HRMS (ESI) *m*/*z*: calcd for C_32_H_35_N_5_NaO_3_, 560.2638 [M + Na]^+^; found, 560.2641. HPLC purity (method B): 96.1% (*t*_R_ = 17.10 min).

#### *N*-{5-[4-(Cyclopentylamino)-6-phenylfuro[2,3-*d*]pyrimidin-5-yl]-2-[(2-(dimethylamino)ethyl](methyl)amino]phenyl}prop-2-enamide (**49**)

To a solution of **64n** (28 mg, 0.06 mmol, 1.0 equiv) in dichloromethane (3.0 mL) were added DIPEA (2 μL, 0.01 mmol, 19 mol %), acrylic acid (5 μL, 0.08 mmol, 1.3 equiv), and EDCI (14 mg, 0.07 mmol, 1.2 equiv); then, the reaction mixture was stirred at room temperature. After stirring for 16 h, to the reaction mixture was added ethyl acetate (5 mL) and washed with NaHCO_3(aq)_ (10 mL) and brine (10 mL). The combined organic layers were dried over MgSO_4_, concentrated *in vacuo*, and purified by flash column chromatography (2–3% methanol in dichloromethane) to yield the title compound **49** (13 mg, 0.02 mmol, 42%) as a white solid. ^1^H NMR (600 MHz, DMSO-*d*_6_) δ 10.22 (s, 1H), 8.43 (s, 1H), 8.34 (s, 1H), 7.56 (d, *J* = 7.2 Hz, 2H), 7.47 (d, *J* = 8.4 Hz, 1H), 7.40–7.31 (m, 3H), 7.22 (dd, *J* = 8.4, 2.4 Hz, 1H), 6.52 (s, 1H), 6.25 (dd, *J* = 16.8, 1.8 Hz, 1H), 5.80 (dd, *J* = 10.2, 1.8 Hz, 1H), 4.95 (d, *J* = 6.6 Hz, 1H), 4.35 (dquin, *J* = 6.6, 4.2 Hz, 1H), 2.97 (s, 2H), 2.76 (s, 1H), 2.36–2.20 (m, 6H), 1.86–1.77 (m, 2H), 1.54–1.32 (m, 4H), 1.31–1.18 (m, 2H). ^13^C NMR (150 MHz, DMSO-*d*_6_) δ 164.3, 163.4, 156.6, 154.1, 145.3, 143.6, 131.6, 129.1, 128.8, 128.7, 127.3, 126.9, 125.8, 125.3, 122.8, 115.0, 102.4, 54.9, 51.9, 41.4, 32.5, 22.8. LRMS (ESI) *m*/*z*: 525.3 [M + H]^+^. HRMS (ESI) *m*/*z*: calcd for C_31_H_37_N_6_O_2_, 525.2978 [M + H]^+^; found, 525.2954. HPLC purity (method B): 96.1% (*t*_R_ = 17.10 min).

#### *N*-(2-{[2-(Dimethylamino)ethyl](methyl)amino}-5-[4-(methylamino)-6-phenylfuro[2,3-*d*]pyrimidin-5-yl]phenyl)prop-2-enamide (**50**)

To a solution of **64o** (26 mg, 0.06 mmol, 1.0 equiv) in dichloromethane (5 mL) were added acrylic acid (6 μL, 0.09 mmol, 1.4 equiv) and EDCI (17 mg, 0.09 mmol, 1.4 equiv); then, the reaction mixture was stirred at room temperature. After stirring for 4 h, to the reaction mixture were added water (3 mL), dichloromethane (10 mL), and sat. NaHCO_3(aq)_ (1 mL) and extracted with dichloromethane (10 mL × 3). The combined organic layers were washed with brine (10 mL), dried over MgSO_4_, concentrated *in vacuo*, and purified by flash column chromatography (5% methanol in dichloromethane with 0.1% NH_4_OH) to yield the title compound **50** (12 mg, 0.03 mmol, 41%) as a yellow solid. ^1^H NMR (400 MHz, DMSO-*d*_6_) δ 10.41 (s, 1H), 8.54 (s, 1H), 8.43 (s, 1H), 7.58–7.53 (m, 3H), 7.30–7.20 (m, 4H), 7.10 (dd, *J* = 9.8, 2.4 Hz, 1H), 6.45 (d, *J* = 16.8 Hz, 1H), 5.75 (dd, *J* = 9.8 Hz, 1H), 5.41 (s, 1H), 3.05 (d, *J* = 4.8 Hz, 3H), 2.95 (s, 2H), 2.80 (s, 3H), 2.50–2.26 (m, 8H). ^13^C NMR (150 MHz, DMSO-*d*_6_) δ 164.5, 163.6, 157.7, 153.9, 145.4, 133.7, 131.6, 129.2, 128.7, 128.6, 128.4, 128.2, 127.3, 126.2, 125.6, 122.5, 122.4, 115.4, 102.2, 56.7, 55.4, 45.5, 28.1, 24.9. LRMS (ESI) *m*/*z*: 471.2 [M + H]^+^. HRMS (ESI) *m*/*z*: calcd for C_27_H_31_N_6_O_2_, 471.2509 [M + H]^+^; found, 471.2539. HPLC purity (method B): 92.2% (*t*_R_ = 14.44 min).

#### *N*-(2-{[2-(Dimethylamino)ethyl](methyl)amino}-5-[4-(ethylamino)-6-phenylfuro[2,3-*d*]pyrimidin-5-yl]phenyl)prop-2-enamide (**51**)

To a solution of **64p** (18 mg, 0.04 mmol, 1.0 equiv) in dichloromethane (5 mL) were added acrylic acid (4 μL, 0.06 mmol, 1.4 equiv) and EDCI (12 mg, 0.06 mmol, 1.5 equiv); then, the reaction mixture was stirred at room temperature. After stirring for 4 h, to the reaction mixture were added water (3 mL), dichloromethane (10 mL), and sat. NaHCO_3(aq)_ (1 mL) and extracted with dichloromethane (10 mL × 3). The combined organic layers were washed with brine (10 mL), dried over MgSO_4_, concentrated *in vacuo*, and purified by flash column chromatography (5% methanol in dichloromethane with 0.1% NH_4_OH) to yield the title compound **51** (10 mg, 0.02 mmol, 49%) as a yellow solid. ^1^H NMR (400 MHz, DMSO-*d*_6_) δ 10.33 (s, 1H), 8.69 (s, 1H), 8.40 (s, 1H), 7.58 (d, *J* = 8.0 Hz, 2H), 7.32–7.23 (m, 5H), 7.11 (dd, *J* = 8.4, 2.4 Hz, 1H), 6.44 (dd, *J* = 17.2, 2.0 Hz, 1H), 6.35 (s, 1H), 5.74 (dd, *J* = 10.0, 2.4 Hz, 1H), 5.07 (s, 1H), 3.49 (dq, *J* = 7.2, 5.2 Hz, 2H), 2.94 (s, 2H), 2.80 (s, 3H), 2.50–2.24 (m, 8H), 1.09 (t, *J* = 7.2 Hz, 3H). ^13^C NMR (150 MHz, DMSO-*d*_6_) δ 164.5, 163.5, 157.0, 154.0, 145.4, 144.0, 133.9, 131.6, 129.2, 128.8, 128.7, 127.3, 126.1, 125.6, 122.5, 115.2, 102.1, 56.6, 55.2, 45.4, 41.0, 35.4, 14.2. LRMS (ESI) *m*/*z*: 485.3 [M + H]^+^. HRMS (ESI) *m*/*z*: calcd for C_28_H_33_N_6_O_2_, 485.2665 [M + H]^+^; found, 485.2665. HPLC purity (method B): 98.0% (*t*_R_ = 15.32 min).

#### *N*-(2-{[2-(Dimethylamino)ethyl](methyl)amino}-5-[6-phenyl-4-(propan-2-ylamino)furo[2,3-*d*]pyrimidin-5-yl]phenyl)prop-2-enamide (**52**)

To a solution of **64q** (19 mg, 0.04 mmol, 1.0 equiv) in dichloromethane (5.0 mL) were added acrylic acid (4 μL, 0.06 mmol, 1.4 equiv) and EDCI (12 mg, 0.06 mmol, 1.5 equiv); then, the reaction mixture was stirred at room temperature. After stirring for 4 h, to the reaction mixture were added water (3 mL), dichloromethane (10 mL), and sat. NaHCO_3(aq)_ (1 mL) and extracted with dichloromethane (10 mL × 3). The combined organic layers were washed with brine (10 mL), dried over MgSO_4_, concentrated *in vacuo*, and purified by flash column chromatography (5% methanol in dichloromethane with 0.1% NH_4_OH) to yield the title compound **52** (10 mg, 0.02 mmol, 47%) as a yellow solid. ^1^H NMR (600 MHz, DMSO-*d*_6_) δ 10.27 (s, 1H), 8.41 (d, *J* = 2.1 Hz, 1H), 8.34 (s, 1H), 7.55 (d, *J* = 6.6 Hz, 2H), 7.45 (d, *J* = 8.4 Hz, 1H), 7.41–7.31 (m, 3H), 7.19 (dd, *J* = 8.4, 2.1 Hz, 1H), 6.47 (dd, *J* = 17.4, 10.2 Hz, 1H), 6.25 (dd, *J* = 17.4, 2.1 Hz, 1H), 5.80 (dd, *J* = 10.2, 2.1 Hz, 1H), 4.93 (d, *J* = 7.2 Hz, 1H), 4.17 (dset, *J* = 7.2, 6.6, 6.3 Hz, 1H), 2.93 (t, *J* = 6.0 Hz, 2H), 2.78 (s, 3H), 2.38 (t, *J* = 6.0 Hz, 2H), 2.21 (s, 6H), 1.03 (d, *J* = 6.3 Hz, 6H). ^13^C NMR (150 MHz, DMSO-*d*_6_) δ 164.4, 163.4, 156.5, 154.1, 145.3, 143.7, 134.4, 131.6, 129.1, 128.8, 128.7, 127.3, 126.6, 126.0, 125.4, 122.7, 121.5, 115.1, 102.2, 56.7, 55.5, 45.4, 42.3, 41.3, 22.0. LRMS (ESI) *m*/*z*: 499.3 [M + H]^+^. HRMS (ESI) *m*/*z*: calcd for C_29_H_35_N_6_O_2_, 499.2822 [M + H]^+^; found, 499.2824. HPLC purity (method B): 99.0% (*t*_R_ = 16.31 min).

#### *N*-{5-[4-(Cyclopropylamino)-6-phenylfuro[2,3-*d*]pyrimidin-5-yl]-2-{[2-(dimethylamino)ethyl](methyl)amino}phenyl}prop-2-enamide (**53**)

To a solution of **64r** (29 mg, 0.07 mmol, 1.0 equiv) in dichloromethane (5.0 mL) were added acrylic acid (7 μL, 0.10 mmol, 1.6 equiv) and EDCI (19 mg, 0.10 mmol, 1.5 equiv); then, the reaction mixture was stirred at room temperature. After stirring for 2.5 h, to the reaction mixture were added water (3 mL), dichloromethane (10 mL), and sat. NaHCO_3(aq)_ (1 mL) and extracted with dichloromethane (10 mL × 3). The combined organic layers were washed with brine (10 mL), dried over MgSO_4_, concentrated *in vacuo*, and purified by flash column chromatography (3% methanol in dichloromethane with 0.1% NH_4_OH) to yield the title compound **53** (17 mg, 0.03 mmol, 52%) as a yellow oil. ^1^H NMR (600 MHz, DMSO-*d*_6_) δ 10.25 (s, 1H), 8.40 (s, 1H), 8.32 (d, *J* = 2.4 Hz, 1H), 7.53 (d, *J* = 6.6 Hz, 2H), 7.41 (d, *J* = 8.4 Hz, 1H), 7.38–7.31 (m, 3H), 7.15 (dd, *J* = 8.4, 2.1 Hz, 1H), 6.47 (dd, *J* = 16.8, 10.2 Hz, 1H), 6.25 (dd, *J* = 16.8, 1.8 Hz, 1H), 5.80 (dd, *J* = 10.2, 1.8 Hz, 1H), 5.32 (d, *J* = 3.6 Hz, 1H), 2.93 (t, *J* = 6.0 Hz, 2H), 2.86–2.80 (m, 1H), 2.77 (s, 3H), 2.40 (t, *J* = 6.0 Hz, 2H), 2.22 (s, 6H), 0.72–0.66 (m, 2H), 0.35–0.32 (m, 2H). ^13^C NMR (150 MHz, DMSO-*d*_6_) δ 164.4, 163.4, 158.1, 154.0, 145.6, 143.8, 134.1, 131.6, 129.1, 128.8, 128.7, 127.3, 126.4, 126.1, 125.4, 122.6, 121.7, 115.1, 102.6, 56.7, 55.4, 45.5, 41.1, 23.9, 6.8. LRMS (ESI) *m*/*z*: 497.4 [M + H]^+^. HRMS (ESI) *m*/*z*: calcd for C_29_H_33_N_6_O_2_, 497.2665 [M + H]^+^; found, 497.2659. HPLC purity (method B): 96.4% (*t*_R_ = 14.46 min).

#### *N*-(2-{[2-(Dimethylamino)ethyl](methyl)amino}-5-{4-[(2-hydroxyethyl)amino]-6-phenylfuro[2,3-*d*]pyrimidin-5-yl}phenyl)prop-2-enamide (**54**)

To a solution of **64s** (56 mg, 0.13 mmol, 1.0 equiv) in dichloromethane (5.0 mL) were added acrylic acid (9 μL, 0.13 mmol, 1.1 equiv) and EDCI (36 mg, 0.19 mmol, 1.5 equiv); then, the reaction mixture was stirred at room temperature. After stirring for 16 h, the reaction mixture was concentrated *in vacuo* and purified by flash column chromatography (2–5% methanol in dichloromethane with 0.1% NH_4_OH) to yield the title compound **54** (36 mg, 0.07 mmol, 57%) as a yellow solid. ^1^H NMR (600 MHz, DMSO-*d*_6_) δ 10.32 (s, 1H), 8.36 (d, *J* = 2.1 Hz, 1H), 8.33 (s, 1H), 7.52 (d, *J* = 7.2 Hz, 2H), 7.41 (d, *J* = 8.1 Hz, 1H), 7.38–7.30 (m, 3H), 7.19 (dd, *J* = 8.1, 2.1 Hz, 1H), 6.44 (dd, *J* = 17.1, 10.2 Hz, 1H), 6.24 (dd, *J* = 17.1, 2.1 Hz, 1H), 5.79 (dd, *J* = 10.2, 2.1 Hz, 1H), 5.50 (t, *J* = 5.4 Hz, 1H), 3.50–3.38 (m, 4H), 2.91 (t, *J* = 6.0 Hz, 2H), 2.76 (s, 3H), 2.40 (t, *J* = 6.0 Hz, 2H), 2.23 (s, 6H). ^13^C NMR (150 MHz, DMSO-*d*_6_) δ 164.5, 163.4, 157.2, 154.0, 145.3, 143.7, 134.4, 131.7, 129.2, 128.8, 128.6, 127.2, 126.5, 126.0, 125.1, 122.7, 121.6, 115.3, 102.3, 59.1, 56.7, 55.7, 45.5, 42.8, 41.1. LRMS (ESI) *m*/*z*: 501.4 [M + H]^+^. HRMS (ESI) *m*/*z*: calcd for C_28_H_33_N_6_O_3_, 501.2614 [M + H]^+^; found, 501.2593. HPLC purity (method B): 93.4% (*t*_R_ = 13.60 min).

#### *N*-(2-{[2-(Dimethylamino)ethyl](methyl)amino}-5-{4-[((1S,2R)-2-hydroxycyclopentyl)amino]-6-phenylfuro[2,3-*d*]pyrimidin-5-yl}phenyl)prop-2-enamide (**55**)

To a solution of **64t** (88 mg, 0.18 mmol, 1.0 equiv) in dichloromethane (0.9 mL) were added acrylic acid (15 μL, 0.22 mmol, 1.2 equiv) and EDCI (52 mg, 0.27 mmol, 1.5 equiv); then, the reaction mixture was stirred at room temperature. After stirring for 16 h, the reaction mixture was concentrated *in vacuo* and purified by flash column chromatography (3% methanol in dichloromethane) to yield the title compound **55** (55 mg, 0.10 mmol, 56%) as a yellow solid. ^1^H NMR (600 MHz, DMSO-*d*_6_) δ 10.32 (s, 1H), 8.45 (s, 1H), 8.31 (s, 1H), 7.55 (d, *J* = 8.4 Hz, 2H), 7.44 (d, *J* = 8.4 Hz, 1H), 7.38–7.29 (m, 3H), 7.20 (dd, *J* = 8.4, 2.4 Hz, 1H), 6.41 (dd, *J* = 16.8, 10.2 Hz, 1H), 6.22 (dd, *J* = 16.8, 2.1 Hz, 1H), 5.77 (dd, *J* = 10.2, 2.1 Hz, 1H), 5.63 (d, *J* = 7,2 Hz, 1H), 4.63 (s, 1H), 4.15 (t, *J* = 7.2 Hz, 1H), 3.94–3.88 (m, 1H), 2.95–2.84 (m, 2H), 2.76 (s, 3H), 2.39 (t, *J* = 5.4 Hz, 2H), 2.33 (s, 6H), 2.00–1.85 (m, 1H), 1.76–1.68 (m 1H), 1.64–1.54 (m, 1H), 1.46–1.29 (m, 3H). ^13^C NMR (150 MHz, DMSO-*d*_6_) δ 164.4, 163.3, 156.8, 154.1, 145.1, 143.3, 134.9, 131.8, 129.2, 128.8, 128.6, 127.1, 126.8, 125.8, 124.8, 122.9, 115.5, 102.5, 70.3, 56.7, 55.9, 54.4, 45.4, 41.3, 32.3, 29.4, 20.0. LRMS (ESI) *m*/*z*: 541.3 [M + H]^+^. HRMS (ESI) *m*/*z*: calcd for C_31_H_37_N_6_O_3_, 541.2927 [M + H]^+^; found, 541.2916. HPLC purity (method B): 98.2% (*t*_R_ = 15.07 min).

#### *N*-(2-{[2-(Dimethylamino)ethyl](methyl)amino}-5-{4-[((1R,2R)-2-hydroxycyclopentyl)amino]-6-phenylfuro[2,3-*d*]pyrimidin-5-yl}phenyl)prop-2-enamide (**56**)

To a solution of **64u** (54 mg, 0.11 mmol, 1.0 equiv) in dichloromethane (1.5 mL) were added acrylic acid (9 μL, 0.13 mmol, 1.2 equiv) and EDCI (32 mg, 0.17 mmol, 1.5 equiv); then, the reaction mixture was stirred at room temperature. After stirring for 16 h, the reaction mixture was concentrated *in vacuo* and purified by flash column chromatography (5% methanol in dichloromethane) to yield the title compound **56** (38 mg, 0.07 mmol, 63%) as colorless oil. ^1^H NMR (600 MHz, DMSO-*d*_6_) δ 10.30 (s, 1H), 8.45 (d, *J* = 2.4 Hz, 1H), 8.35 (s, 1H), 7.55 (d, *J* = 6.6 Hz, 2H), 7.45 (d, *J* = 8.1 Hz, 1H), 7.38–7.30 (m, 3H), 7.20 (dd, *J* = 8.1, 2.4 Hz, 1H), 6.46 (dd, *J* = 16.8, 10.2 Hz, 1H), 6.24 (dd, *J* = 16.8, 1.8 Hz, 1H), 5.80 (dd, *J* = 10.2, 1.8 Hz, 1H), 4.90–4.80 (m, 2H), 4.16–4.10 (m, 1H), 3.74–3.67 (m, 1H), 2.90 (t, *J* = 6.0 Hz, 2H), 2.76 (s, 3H), 2.40 (t, *J* = 6.0 Hz, 2H), 2.22 (s, 6H), 2.03–1.95 (m, 1H), 1.65–1.55 (m 1H), 1.54–1.46 (m, 1H), 1.42–1.34 (m, 1H), 1.32–1.16 (m, 1H). ^13^C NMR (150 MHz, DMSO-*d*_6_) δ 164.3, 163.4, 156.7, 154.1, 145.4, 143.6, 134.6, 131.6, 129.1, 128.8, 128.7, 127.4, 126.8, 125.8, 125.2, 122.8, 121.0, 115.0, 102.5, 75.8, 58.7, 56.7, 55.6, 45.5, 41.2, 31.6, 29.4, 20.2. LRMS (ESI) *m*/*z*: 541.5 [M + H]^+^. HRMS (ESI) *m*/*z*: calcd for C_31_H_37_N_6_O_3_, 541.2927 [M + H]^+^; found, 541.2907. HPLC purity (method B): 95.2% (*t*_R_ = 14.85 min).

#### *N*-(2-{[2-(Dimethylamino)ethyl](methyl)amino}-5-{4-[(3-hydroxycyclobutyl)amino]-6-phenylfuro[2,3-*d*]pyrimidin-5-yl}phenyl)prop-2-enamide (**57**)

To a solution of **64v** (75 mg, 0.16 mmol, 1.0 equiv) in dichloromethane (0.8 mL) were added acrylic acid (13 μL, 0.19 mmol, 1.2 equiv) and EDCI (46 mg, 0.24 mmol, 1.5 equiv); then, the reaction mixture was stirred at room temperature. After stirring for 16 h, the reaction mixture was concentrated *in vacuo* and purified by flash column chromatography (3% methanol in dichloromethane) to yield the title compound **57** (35 mg, 0.07 mmol, 42%) as a white solid. ^1^H NMR (600 MHz, DMSO-*d*_6_) δ 10.34 (s, 1H), 8.40 (d, *J* = 2.4 Hz, 1H), 8.33 (s, 1H), 7.54 (d, *J* = 7.2 Hz, 2H), 7.46 (d, *J* = 8.1 Hz, 1H), 7.39–7.31 (m, 3H), 7.21 (dd, *J* = 8.1, 2.4 Hz, 1H), 6.46 (dd, *J* = 17.4, 10.2 Hz, 1H), 6.27 (dd, *J* = 17.4, 1.8 Hz, 1H), 5.80 (dd, *J* = 10.2, 1.8 Hz, 1H), 5.21 (d, *J* = 7,2 Hz, 1H), 5.00 (s, 1H), 4.03–3.95 (m, 1H), 3.81 (ddd, *J* = 7.2, 7.2, 7.2 Hz, 1H), 2.94 (t, *J* = 6.0 Hz, 2H), 2.79 (s, 3H), 2.60–2.53 (m, 2H), 2.41 (t, *J* = 6.0 Hz, 2H), 2.22 (s, 6H), 1.51–1.44 (m 2H). ^13^C NMR (150 MHz, DMSO-*d*_6_) δ 164.5, 163.5, 156.3, 154.0, 145.5, 143.9, 134.3, 131.6, 129.1, 128.8, 128.7, 127.4, 126.5, 126.0, 125.4, 122.6, 121.7, 115.1, 102.3, 59.1, 56.8, 55.6, 45.5, 41.4, 41.2, 37.3. LRMS (ESI) *m*/*z*: 527.3 [M + H]^+^. HRMS (ESI) *m*/*z*: calcd for C_30_H_34_N_6_NaO_3_, 549.2590 [M + Na]^+^; found, 549.2589. HPLC purity (method B): 98.1% (*t*_R_ = 14.06 min).

#### *N*-(2-{[2-(Dimethylamino)ethyl](methyl)amino}-5-{4-[(3-hydroxycyclobutyl)amino]-6-phenylfuro[2,3-*d*]pyrimidin-5-yl}phenyl)prop-2-enamide (**58**)

To a solution of **64w** (66 mg, 0.16 mmol, 1.0 equiv) in dichloromethane (0.7 mL) were added acrylic acid (11 μL, 0.16 mmol, 1.1 equiv) and EDCI (40 mg, 0.21 mmol, 1.5 equiv); then, the reaction mixture was stirred at room temperature. After stirring for 16 h, the reaction mixture was concentrated *in vacuo* and purified by flash column chromatography (5% methanol in dichloromethane) to yield the title compound **58** (16 mg, 0.03 mmol, 22%) as a white solid. ^1^H NMR (600 MHz, DMSO-*d*_6_) δ 10.32 (s, 1H), 8.42 (d, *J* = 2.4 Hz, 1H), 8.34 (s, 1H), 7.56 (d, *J* = 6.6 Hz, 2H), 7.45 (d, *J* = 8.1 Hz, 1H), 7.40–7.31 (m, 3H), 7.21 (dd, *J* = 8.1, 2.4 Hz, 1H), 6.47 (dd, *J* = 17.4, 10.5 Hz, 1H), 6.26 (dd, *J* = 17.4, 2.4 Hz, 1H), 5.80 (dd, *J* = 10.5, 2.4 Hz, 1H), 5.30 (d, *J* = 6.6 Hz, 1H), 5.02 (s, 1H), 4.50–4.42 (m, 1H), 4.12–4.05 (m, 1H), 2.93 (t, *J* = 6.0 Hz, 2H), 2.78 (s, 3H), 2.40 (t, *J* = 6.0 Hz, 2H), 2.22 (s, 6H), 2.17–2.11 (m, 2H), 1.95–1.87 (m 2H). ^13^C NMR (150 MHz, DMSO-*d*_6_) δ 164.4, 163.4, 156.5, 154.0, 145.4, 143.8, 134.3, 131.6, 129.1, 128.8, 128.7, 127.3, 126.6, 126.0, 125.4, 122.7, 121.5, 115.1, 102.6, 63.0, 56.7, 55.6, 45.5, 45.4, 42.1, 41.2. LRMS (ESI) *m*/*z*: 527.3 [M + H]^+^. HRMS (ESI) *m*/*z*: calcd for C_30_H_35_N_6_O_3_, 527.2771 [M + H]^+^; found, 527.2781. HPLC purity (method B): 98.2% (*t*_R_ = 14.10 min).

### Molecular Docking Studies

For carrying out the noncovalent docking of ligand **6** in EGFR^WT^ (PDB ID: 6JZ0), Schrodinger Glide XP^33^ was used. Ligands were prepared at pH 7 ± 2 using Ligprep module before docking in the active site defined by the cocrystal ligand **11** present in the EGFR protein. For carrying out the covalent docking of **11** and **52** to EGFR^WT^ (PDB ID: 6JXT) and EGFR^T790M^ (PDB ID: 6JX0), covalent docking module of Schrodinger was used. Ligands were prepared at pH 7±2 using Ligprep module before docking in the active site defined by the cocrystal ligand **6** present in the EGFR protein. Nucleophilic addition reaction and Cys797 residue were chosen to perform the covalent docking. Top scoring poses were retrieved and analyzed.

### EGFR^WT^ and EGFR^L858R/T790M^ Enzyme Inhibition Assays

Kinase-Glo Plus Luminescent Kinase Assay (Promega) was used to test the inhibition potential of the newly synthesized compounds for GST-EGFR (G696-G1022) wild-type and L858R/T790M double-mutant recombinant proteins. Detailed methodology for carrying out the enzyme inhibition assay could be found in our previous publication.^28^

### Cell Proliferation Assay and Western Blot Analysis

A431 (wild-type EGFR overexpressing) and H1975 (double-mutant overexpressing) cells were exposed to test compounds for 96 h and the cell proliferation was determined by the MTS method. Western blot analysis was performed by exposing the cells (A431 and H1975) to the test compound for 1 h. Drug-treated cells were lysed and subjected to western blot analysis. Refer to our previous publication for detailed methods of cell proliferation and western blot analysis.^28,34^

### *In Vitro* Microsomal Stability Assay

*In vitro* drug metabolism (hCyt450) assay was performed as reported earlier by us.^35^

### *In Vivo* Pharmacokinetics Study

Male Sprague Dawley rats (300–400 g) were obtained from BioLASCO Taiwan Co., Ltd. (Ilan, Taiwan) and used for pharmacokinetics studies as reported earlier.^35^ The animal studies were conducted according to NHRI institutional animal care and committee-approved procedures.

### *In Vivo* BaF3 EGFR^L858R/T790M^ Xenograft Study

Adult male nude mice (Nu-Fox1nu) purchased from BioLasco, Taiwan Co., Ltd. were used for this study. Animals had access to food and water ad libitum. Institutional Animal Care and Use Committees (IACUC) of the National Health Research Institutes approved the study using animals. EGFR^L858R/T790M^ expressing human BaF3 cells were cultured in RPMI-1640 supplemented with 10% (v/v) fetal bovine serum (FBS) at 37 °C in a humidified atmosphere consisting of 5% CO_2_. Each 9-week-old nude mouse was inoculated subcutaneously with 1 × 10^6^ BaF3 EGFR^L858R/T790M^ cells in 50% Matrigel (Becton Bickinson) in 0.1 mL injection volume *via* a 24-gauge needle. Tumor volume was measured with the help of an electronic caliper and calculated using the formula, length × width^2^ × 0.5. When the size of a growing tumor reached ∼280 mm^3^, the tumor-bearing mice were administered compound **49** or **52** (dissolved in 20% 2-hydroxypropyl-β-cyclodextrin in water) by oral gavage 5 days a week for 2 consecutive weeks at 100 mg/kg doses. **6** (AZD9291) was administered orally at 10 mg/kg doses in the same regimen for comparison. The control group received vehicle alone (20% 2-hydroxypropyl-β-cyclodextrin in water) by oral gavage.

### *In Vivo* H1975 Xenograft Study

To assess the antitumor activity of **52**, five-week-old male nude mice (National Laboratory Animal Center, NLAC, Taipei, Taiwan) were subcutaneously injected with 1 × 10^7^ H1975 cells. When the tumor sizes reached 100 mm^3^, mice were divided into four groups randomly (control, **6** (AZD9291) 10 mg/kg, **52** 10 and 30 mg/kg groups, with 9–10 mice in each group). Mice in control groups were dosed with the vehicle (20% 2-hydroxypropyl-β-cyclodextrin, HP-β-CD) by oral administration (po) once daily (qd); **6** (AZD9291) group was dosed with 10 mg/kg of **6** (AZD9291) po, qd; **52** groups were dosed with 10 or 30 mg/kg of **52** po, qd. The tumor size and body weight were measured twice per week during the experiment, and the tumor volume (mm^3^) was calculated as mentioned above. Tumor growth inhibition (% TGI) = [1 – (*T_t_* – *T*_0_)/(*C_t_* – *C*_0_) × 100], where *C*_0_ and *C_t_* are mean tumor volumes of the control group by first data point and day *t*, respectively, while *T*_0_ and *T_t_* are mean tumor volumes of the treatment group by first data point and day *t*, respectively. This study was approved by Animal Use and Management Committee of Taipei Medical University (IACUC number LAC-2017-0444).

## Supporting Information Available

^1^H NMR, ^13^C NMR, and HPLC purity spectra of final compounds **13–58**, synthesis procedure and compound characterization data for intermediate compounds **60–64**, **66**, **67**, and **69–72**, rat body weight change during drug treatment, and kinase profiling data of **52** (PDF)
SMILES string for compounds **13**–**58** (CSV)
6JX0_11 (Docking pose of 11 in EGFR T790M) (PDB)
6JX0_52 (Docking pose of 52 in EGFR T790M) (PDB)
6JXT_11 (Docking pose of 11 in EGFR WT) (PDB)
6JXT_52 (Docking pose of 52 in EGFR WT) (PDB)
6JZ0_6 (Docking pose of 6 in EGFR WT) (PDB)

## Supplementary Material

jm2c01434_si_002.csv

jm2c01434_si_003.pdb

jm2c01434_si_004.pdb

jm2c01434_si_005.pdb

jm2c01434_si_006.pdb

jm2c01434_si_007.pdb

## Abbreviations Used

3D: three-dimensional
ACN: acetonitrile
ATP: adenosine triphosphate
AUC: area under the curve
BLK: B lymphocyte kinase
Boc: di-*tert*-butyl dicarbonate
BTK: Bruton’s tyrosine kinase
^*n*^BuOH: *n*-butanol
CC_50_: half-maximal cytotoxic concentration
DIPEA: *N*,*N*-diisopropylethylamine
DMF: *N*,*N*-dimethylformamide
DMPK: drug metabolism and pharmacokinetics
DMSO: dimethyl sulfoxide
EDCI: 1-ethyl-3-(3-dimethylaminopropyl)carbodiimide
EGFR: epidermal growth factor receptor
equiv: equivalence
ERBB2: receptor tyrosine-protein kinase erbB-2
ERBB4: receptor tyrosine-protein kinase erbB-4
ESI: electrospray ionization
Et_3_N: triethylamine
F: oral bioavailability
FBS: fetal bovine serum
FDA: food and drug administration
GI: gastrointestinal
HCl: hydrochloride salt
HPLC: high-performance liquid chromatography
HRMS: high-resolution mass spectrometry
IC_50_: half-maximal inhibitory concentration
IPA: isopropyl alcohol
IV: intravenous administration
JAK3: Janus kinase 3
LC-MS: liquid chromatograph/mass spectrometer
LRMS: low-resolution mass spectrometry
MHz: megahertz
Ml. Wt.: molecular weight
NBS: *N*-bromo succinimide
ND: not detected/determined
NHRI: National Health Research Institutes
NMR: nuclear magnetic resonance
NSCLC: non-small cell lung cancer
OTBS: *tert*-butyldimethylsilyl ether
PDB: protein data bank
PK: pharmacokinetics
PO: oral administration
QD: once daily
RBN: number of rotatable bonds
rt: room temperature
SAR: structure–activity relationship
S_N_Ar: aromatic substitution reaction
TBSCl: *tert*-butyldimethylsilyl chloride
TFA: trifluoroacetic acid
TGI: tumor growth inhibition
THF: tetrahydrofuran
TLC: thin-layer chromatography
vs: versus
WT: wild-type
