# Supplementary material for: Development of Furanopyrimidine-Based Orally Active Third-Generation EGFR Inhibitors for the Treatment of Non-Small Cell Lung Cancer
Source: J Med Chem. 2023 Feb 7;66(4):2566–88. doi: 10.1021/acs.jmedchem.2c01434 (PMC9969398; doi:10.1021/acs.jmedchem.2c01434)
Supplement: Supplementary file 1 — jm2c01434_si_001.pdf [file jm2c01434_si_001.pdf]

Supporting Information

Development of Furanopyrimidine-Based Orally Active Third-Generation EGFR Inhibitors for the Treatment of Non-Small Cell Lung Cancer

Mu-Chun Li,[a,b#] Mohane Selvaraj Coumar,[c#] Shu-Yu Lin,[a] Yih-Shyan Lin,[a] Guan-Lin Huang,[a] Chun-Hwa Chen,[a] Tzu-Wen Lien,[a] Yi-Wen Wu,[d] Yen-Ting Chen,[a] Ching-Ping Chen,[a] Yu-Chen Huang,[a] Kai-Chia Yeh,[a] Chen-Ming Yang,[a] Bikashita Kalita,[c] Shiow-Lin Pan,[d,e] Tsu-An Hsu,[a] Teng-Kuang Yeh,[a] Chiung-Tong Chen,[a] and Hsing-Pang Hsieh[a,b,f*]

[a]Institute of Biotechnology and Pharmaceutical Research, National Health Research Institutes, Miaoli County 350401, Taiwan, ROC.
[b]Biomedical Translation Research Center, Academia Sinica, Taipei City 115202, Taiwan, ROC.
[c]Department of Bioinformatics, School of Life Sciences, Pondicherry University, Kalapet, Pondicherry 605014, India.
[d]Graduate Institute of Cancer Biology and Drug Discovery, College of Medical Science and Technology, Taipei Medical University, Taipei City 110301, Taiwan, ROC.
[e]Ph.D. Program in Drug Discovery and Development Industry, College of Pharmacy, Taipei Medical University, Taipei City 110301, Taiwan, ROC
[f]Department of Chemistry, National Tsing Hua University, Hsinchu City 300044, Taiwan, ROC.

[#]M.-C. Li and M. S. Coumar contributed equally to this work.

Corresponding author:
Prof. Hsing-Pang Hsieh
E-mail: hphsieh@nhri.edu.tw; alexhsieh@gate.sinica.edu.tw
Phone: +886-37-206-166 ext. 35708

Table of Contents

1. **Supplementary Figures** **S3**
2. **Experimental procedures and compound characterization data for 22'–72p** **S5**
3. **^{1}H and ^{13}C spectra of compounds 13–58** **S55**
4. **Kinase profiling data for 52** **S101**
5. **HPLC trace of 49 and 52** **S108**
6. **References** **S110**

1. Supplementary Figures

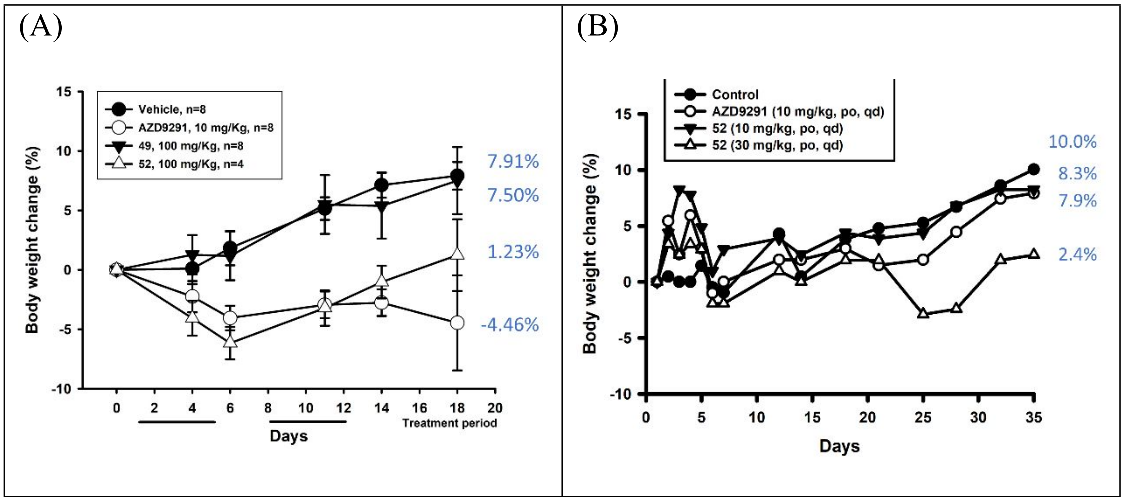

Figure S1. Rat body weight change during in vivo efficacy evaluation of **49** and **52** in mouse xenograft models. (A) % Body weight change in BaF3 EGFR$^{L858R/T790M}$ tumor bearing mice (B) % Body weight change in H1975 tumor bearing mice.

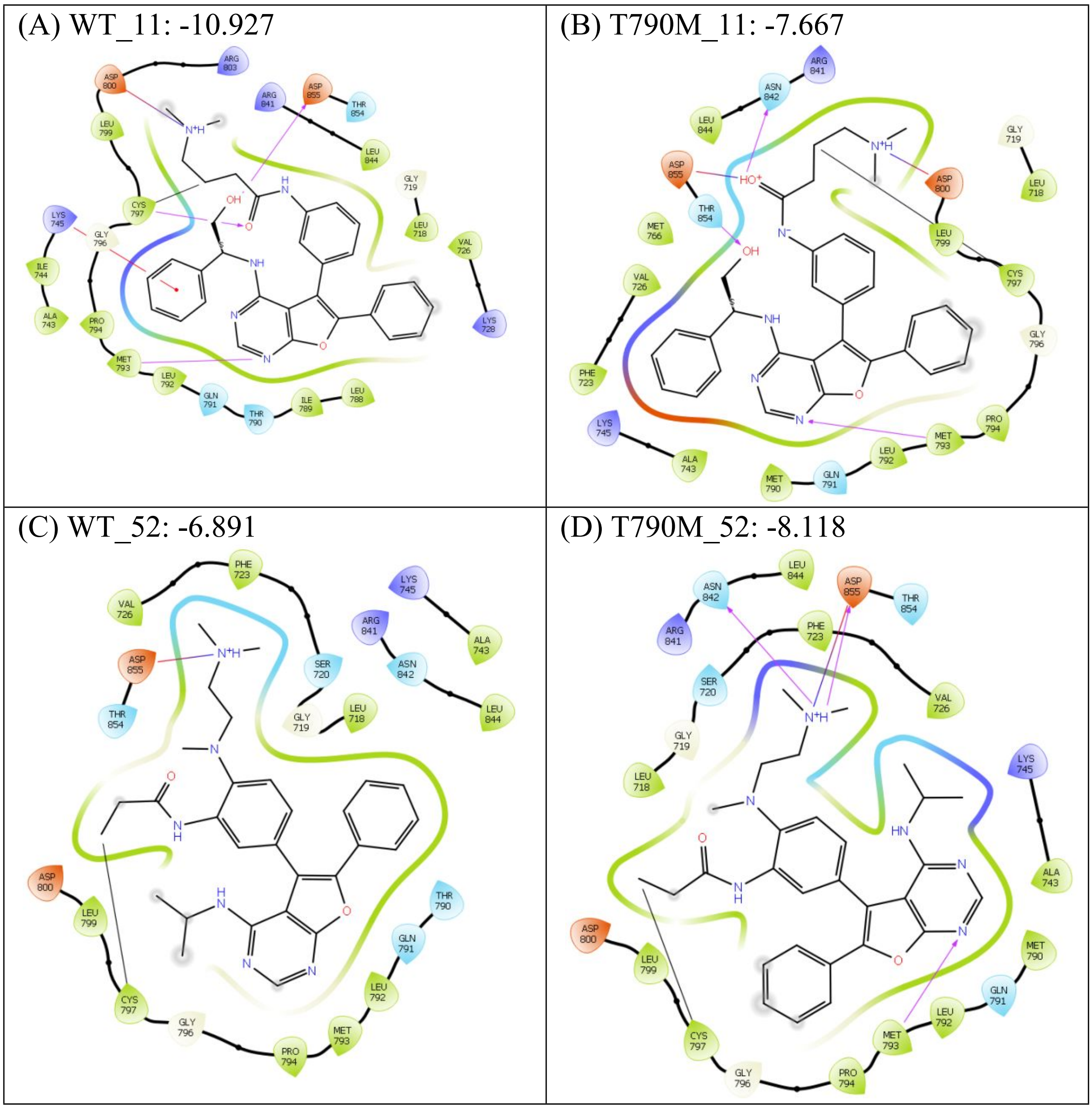

Figure S2. 2D Docked orientation of **11** and **52** in EGFR^{WT} (PDB ID: 6JXT) and EGFR^{T790M} (PDB ID: 6JX0) structures. (A) **11** in EGFR^{WT}, (B) **11** in EGFR^{T790M,}, (C) **52** in EGFR^{WT} and (D) **52** in EGFR^{T790M}.

2. Experimental procedures and compound characterization data for 22’–72p

tert-Butyl [2-({5-[3-(acryloylamino)phenyl]-6-phenylfuro[2,3-_d_]pyrimidin-4-yl}amino)ethyl]carbamate (22’).

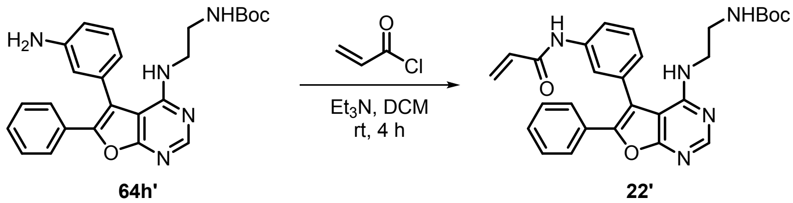

64h' **22'**

To a solution of **64h’** (298 mg, 0.67 mmol, 1.0 equiv.) in dichloromethane (5.0 mL) at 0 °C was added triethylamine (120 µL, 0.86 mmol, 1.3 equiv.) and acryloyl chloride (65 µL, 0.80 mmol, 1.2 equiv.) then the reaction was stirred at room temperature. After stirred for 4 hours, the reaction mixture was concentrated *in vacuo* and purified by flash column chromatography (1% methanol in dichloromethane) to yield the title compound **22’** (215 mg, 0.43 mmol, 64%) as yellow solid. LRMS (ESI) *m*/*z*: 500.2 $[M+H]^+$.

(2*S*)-2-[(5-Bromo-6-phenylfuro[2,3-*d*]pyrimidin-4-yl)amino]-3-methylbutan-1-ol (60a).

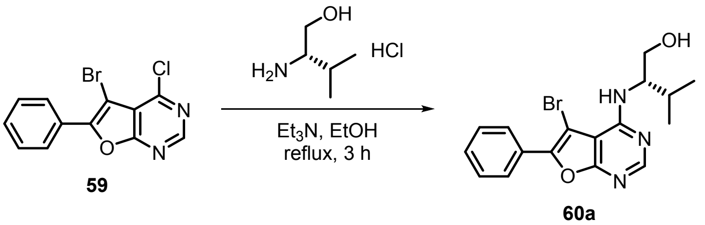

59 **60a**

To a solution of 5-bromo-4-chloro-6-phenylfuro[2,3-*d*]pyrimidine (**59**)[1] (1000 mg, 3.23 mmol, 1.0 equiv.) in ethanol (9.0 mL) was added 2-amino-3-methyl-butan-1-ol hydrochloride (545 mg, 3.55 mmol, 1.1 equiv.) and triethylamine (1 mL, 7.17 mmol, 2.2 equiv.) then the reaction mixture was stirred at reflux. After stirred for 3 hours, the reaction mixture was cooled down to room temperature, the resulting precipitate was washed with cold ethanol (10 mL) to yield the title compound **60a** (1000 mg, 2.56 mmol,79%) as light yellow solid. LRMS (ESI) *m*/*z*: 376.2 $[M+H]^+$.

(2*S*)-2-[(5-Bromo-6-phenylfuro[2,3-*d*]pyrimidin-4-yl)amino]propan-1-ol (60b).

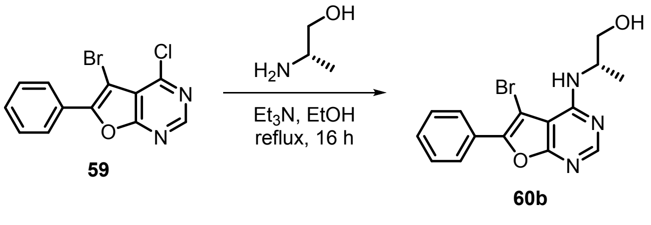

59 **60b**

To a solution of 5-bromo-4-chloro-6-phenylfuro[2,3-*d*]pyrimidine (**59**)[1] (1000 mg, 3.23 mmol, 1.0 equiv.) in ethanol (40 mL) was added triethylamine (0.5 mL, 3.60 mmol, 1.1 equiv.) and (*S*)-2-aminopropan-1-ol (291 mg, 3.87 mmol, 1.2 equiv.) then the reaction mixture was stirred at reflux. After stirred for 16 hours, the reaction mixture was concentrated *in vacuo* and purified by flash column chromatography (0–10% methanol in dichloromethane) to yield the title compound **60b** (802 mg, 2.30 mmol, 71%) as white solid. LRMS (ESI) *m*/*z*: 348.2 $[M+H]^+$.

2-[(5-Bromo-6-phenylfuro[2,3-*d*]pyrimidin-4-yl)amino]ethanol (60c).

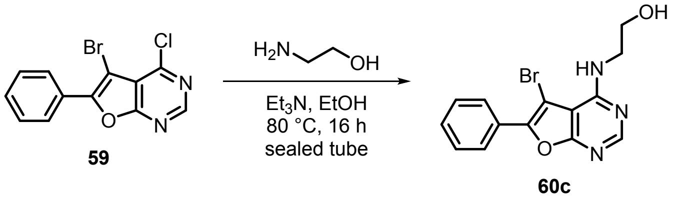

To a solution of 5-bromo-4-chloro-6-phenylfuro[2,3-*d*]pyrimidine (**59**) (100 mg, 0.32 mmol, 1.0 equiv.) in ethanol (2.0 mL) was added 2-aminoethan-1-ol (60 mg, 0.98 mmol, 3.0 equiv.) and triethylamine (58 mg, 0.57 mmol, 1.8 equiv.) then the reaction mixture was stirred at 80 °C in a sealed tube. After stirred for 16 hours, the reaction mixture was cooled down to room temperature, concentrated *in vacuo* and purified by flash column chromatography (5% methanol in dichloromethane with 0.1% NH_4OH) to yield the title compound **60c** (100 mg, 0.30 mmol, 93%) as white solid. LRMS (ESI) *m*/*z*: 348.2 $[M+H]^+$.

3-[(5-Bromo-6-phenylfuro[2,3-*d*]pyrimidin-4-yl)amino]propan-1-ol (60d).

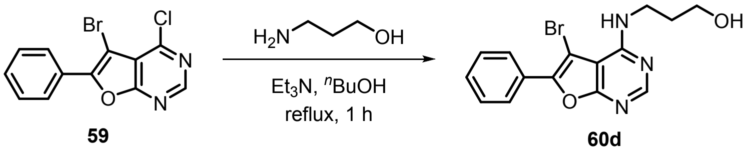

To a solution of 5-bromo-4-chloro-6-phenylfuro[2,3-*d*]pyrimidine (**59**) (50 mg, 0.16 mmol, 1.0 equiv.) in *n*-butanol (2.0 mL) was added 3-amino-1-propanol (24 mg, 0.32 mmol, 2.0 equiv.) and triethylamine (49 mg, 0.48 mmol, 3.0 equiv.) then the reaction mixture was stirred at reflux. After stirred for 1 hour, the reaction mixture was cooled down to room temperature, concentrated *in vacuo* and purified by thin-plate chromatography (5% methanol in dichloromethane) to yield the title compound **60d** (42 mg, 0.12 mmol, 75%) as white solid. LRMS (ESI) *m*/*z*: 348.2 $[M+H]^+$.

5-Bromo-*N*-(2-methoxyethyl)-6-phenylfuro[2,3-*d*]pyrimidin-4-amine (60e).

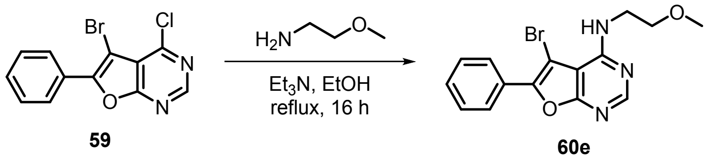

To a solution of 5-bromo-4-chloro-6-phenylfuro[2,3-*d*]pyrimidine (**59**) (500 mg, 1.62 mmol, 1.0 equiv.) in ethanol (5.0 mL) was added 2-methoxyethanamine (60 mg, 0.98 mmol, 3.0 equiv.) and triethylamine (560 μL, 4.02 mmol, 2.5 equiv.) then the reaction mixture was stirred at reflux. After stirred for 16 hours, the reaction mixture was cooled down to room temperature, the resulting precipitate was washed with cold ethanol (10 mL) to yield the title compound **60e** (347 mg, 1.00 mmol, 62%) as light yellow solid. LRMS (ESI) *m*/*z*: 347.2 $[M+H]^+$.

5-Bromo-*N*-(2-phenoxyethyl)-6-phenylfuro[2,3-*d*]pyrimidin-4-amine (60f).

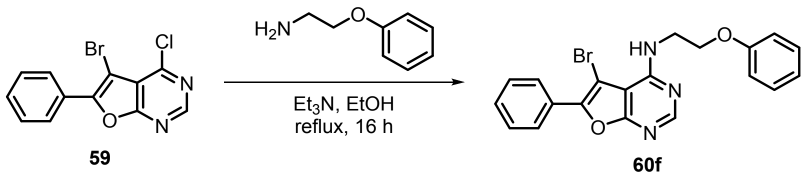

To a solution of 5-bromo-4-chloro-6-phenylfuro[2,3-*d*]pyrimidine (**59**) (500 mg, 1.62 mmol, 1.0 equiv.) in ethanol (10.0 mL) was added 2-phenoxyethylamine (240 mg, 1.75 mmol, 1.1 equiv.) and triethylamine (560 μL, 4.02 mmol, 2.5 equiv.) then the reaction mixture was stirred at reflux. After stirred for 16 hours, the reaction mixture was cooled down to room temperature, the resulting precipitate was collected, washed with cold ethanol (5.0 mL) and ether (2.0 mL) and concentrated *in vacuo* to yield the title compound **60f** (445 mg, 1.08 mmol, 67%) as white solid. LRMS (ESI) *m*/*z*: 410.2 $[M+H]^+$.

Ethyl 3-[(5-bromo-6-phenylfuro[2,3-*d*]pyrimidin-4-yl)amino]propanoate (60g).

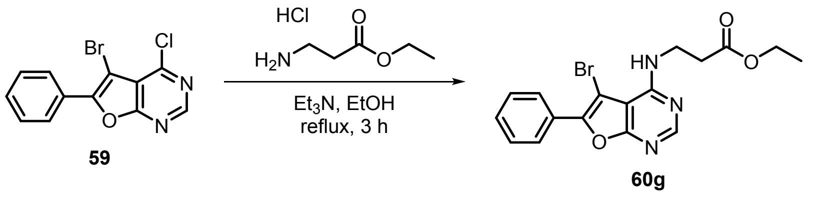

To a solution of 5-bromo-4-chloro-6-phenylfuro[2,3-*d*]pyrimidine (**59**) (1000 mg, 3.23 mmol, 1.0 equiv.) in ethanol (9.0 mL) was added ethyl 3-aminopropanoate hydrochloride (545 mg, 3.55 mmol, 1.1 equiv.) and triethylamine (1 mL, 7.17 mmol, 2.2 equiv.) then the reaction mixture was stirred at reflux. After stirred for 3 hours, the reaction mixture was cooled down to room temperature, the resulting precipitate was

washed with cold ethanol (10 mL) to yield the title compound **60g** (1000 mg, 2.56 mmol, 79%) as light yellow solid. LRMS (ESI) *m*/*z*: 390.2 $[M+H]^+$.

***N*^1-(5-Bromo-6-phenylfuro[2,3-*d*]pyrimidin-4-yl)ethane-1,2-diamine (60h).**

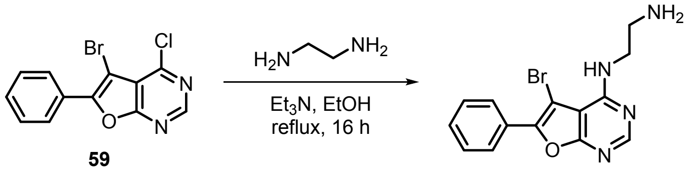

59 **60h**

To a solution of 5-bromo-4-chloro-6-phenylfuro[2,3-*d*]pyrimidine (**59**) (1.00 g, 3.23 mmol, 1.0 equiv.) in ethanol (10.0 mL) was added triethylamine (660 µL, 4.73 mmol mL, 1.5 equiv.) and ethane-1,2-diamine (240 µL, 3.59 mmol, 1.1 equiv.) then the reaction mixture was stirred at reflux. After stirred for 16 hours, the reaction mixture was cooled down to room temperature, added H_2O (50.0 mL), the resulting precipitate was filtered, washed with H_2O (10.0 mL) and hexane (10.0 mL) and dried *in vacuo* to yield the titled compound **60h** (809 mg, 2.43 mmol, 75%) as pale brown solid. LRMS (ESI) *m*/*z*: 333.0 $[M+H]^+$.

***N*^1-(5-Bromo-6-phenylfuro[2,3-*d*]pyrimidin-4-yl)ethane-1,2-diamine (60h').**

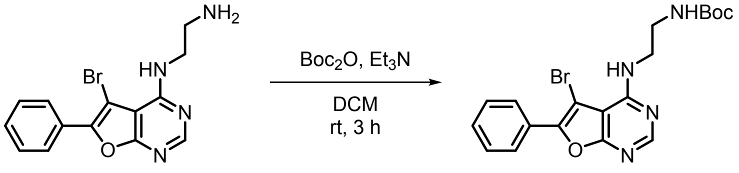

60h **60h'**

To a solution of **60h** (809 mg, 2.43 mmol, 1.0 equiv.) in dichloromethane (2.0 mL) was added triethylamine (440 µL, 3.15 mmol, 1.3 equiv.) and di-*tert*-butyl dicarbonate (840 µL, 3.66 mmol, 1.5 equiv.) then the reaction mixture was stirred at room temperature. After stirred for 3 hours, the reaction mixture was concentrated *in vacuo* and purified by flash column chromatography (5% methanol in dichloromethane) to yield the titled compound **60h'** (393 mg, 0.91 mmol, 37%) as pale brown solid. LRMS (ESI) *m*/*z*: 433.1 $[M+H]^+$.

***N*^1-(5-Bromo-6-phenylfuro[2,3-*d*]pyrimidin-4-yl)ethane-1,2-diamine (60i).**

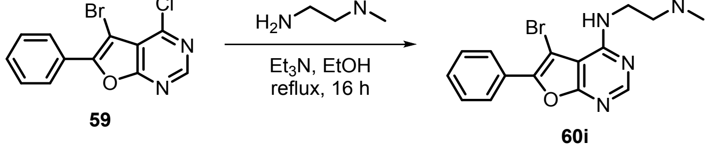

59 **60i**

To a solution of 5-bromo-4-chloro-6-phenylfuro[2,3-*d*]pyrimidine (**59**) (990 mg, 3.20 mmol, 1.0 equiv.) in ethanol (10.0 mL) was added triethylamine (660 µL, 4.73 mmol mL, 1.5 equiv.) and *N*,*N*-dimethylethane-1,2-diamine (380 µL, 3.48 mmol, 1.1 equiv.) then the reaction mixture was stirred at reflux. After stirred for 16 hours, the reaction mixture was cooled down to room temperature, concentrated *in vacuo* and purified by flash column chromatography (3–12% methanol in dichloromethane) to yield the title compound **60i** (864 mg, 2.39 mmol, 75%) as brown solid. LRMS (ESI) *m*/*z*: 361.0 $[M+H]^+$.

5-Bromo-*N*-cyclopentyl-6-phenylfuro[2,3-*d*]pyrimidin-4-amine (60j).

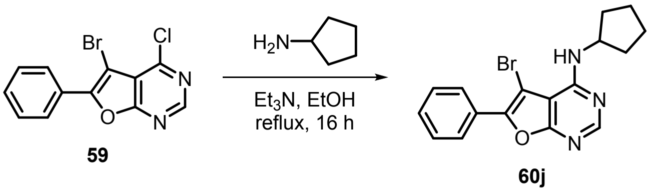

To a solution of 5-bromo-4-chloro-6-phenylfuro[2,3-*d*]pyrimidine (**59**) (300 mg, 0.97 mmol) in ethanol (3.2 mL) was added cyclopentylamine (120 µL, 1.22 mmol, 1.3 equiv.) and triethylamine (200 µL, 1.43 mmol, 1.5 equiv.) then the reaction mixture was stirred at reflux. After stirred for 16 hours, the reaction mixture was cooled down to room temperature, and the resulting precipitate was collected, washed with cold ethanol (5 mL) to yield the title compound **60j** (185 mg, 0.52 mmol, 53%) as yellow solid without further purification. LRMS (ESI) *m*/*z*: 358.1 $[M+H]^+$.

5-Bromo-*N*-methyl-6-phenylfuro[2,3-*d*]pyrimidin-4-amine (60o).

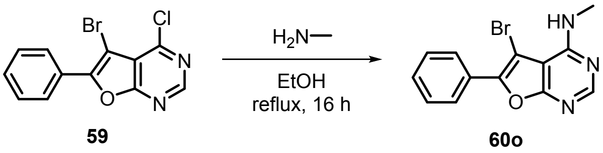

To a solution of 5-bromo-4-chloro-6-phenylfuro[2,3-*d*]pyrimidine (**59**) (150 mg, 0.48 mmol, 1.0 equiv) in ethanol (5.0 mL) was added methylamine (50 µL, 0.58 mmol, 1.2 equiv., 40% in ethanol) and triethylamine (88 µL, 0.63 mmol, 1.3 equiv.) then the reaction mixture was stirred at reflux. After stirred for 16 hours, the reaction mixture was cooled down to room temperature, and the resulting precipitate was collected, washed with cold ethanol (5 mL) to yield the title compound **60o** (120 mg, 0.39 mmol, 81%) as yellow solid without further purification. LRMS (ESI) *m*/*z*: 304.0 $[M+H]^+$.

5-Bromo-*N*-ethyl-6-phenylfuro[2,3-*d*]pyrimidin-4-amine (60p).

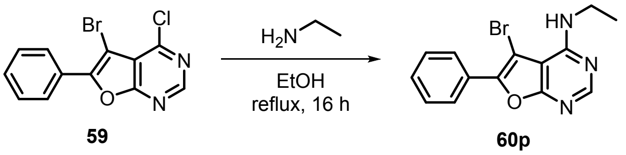

To a solution of 5-bromo-4-chloro-6-phenylfuro[2,3-*d*]pyrimidine (**59**) (150 mg, 0.48 mmol, 1.0 equiv) in ethanol (5.0 mL) was added ethylamine (290 μL, 0.58 mmol, 1.2 equiv., 2M in THF) and triethylamine (88 μL, 0.63 mmol, 1.3 equiv.) then the reaction mixture was stirred at reflux. After stirred for 16 hours, the reaction mixture was cooled down to room temperature, and the resulting precipitate was collected, washed with cold ethanol (5 mL) to yield the title compound **60p** (123 mg, 0.39 mmol, 80%) as yellow solid without further purification. LRMS (ESI) *m*/*z*: 318.1 $[M+H]^+$.

5-Bromo-6-phenyl-*N*-(propan-2-yl)furo[2,3-*d*]pyrimidin-4-amine (60q).

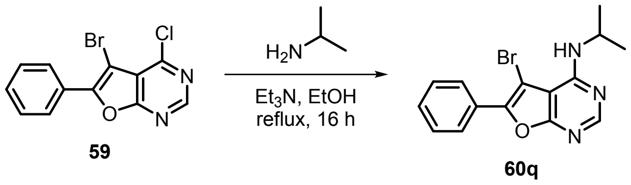

To a solution of 5-bromo-4-chloro-6-phenylfuro[2,3-*d*]pyrimidine (**59**) (150 mg, 0.48 mmol, 1.0 equiv) in ethanol (5.0 mL) was added isopropylamine (50 μL, 0.58 mmol, 1.2 equiv.) and triethylamine (88 μL, 0.63 mmol, 1.3 equiv.) then the reaction mixture was stirred at reflux. After stirred for 16 hours, the reaction mixture was cooled down to room temperature, and the resulting precipitate was collected, washed with cold ethanol (5 mL) to yield the title compound **60q** (123 mg, 0.37 mmol, 76%) as yellow solid without further purification. LRMS (ESI) *m*/*z*: 332.0 $[M+H]^+$.

5-Bromo-*N*-cyclopropyl-6-phenylfuro[2,3-*d*]pyrimidin-4-amine (60r).

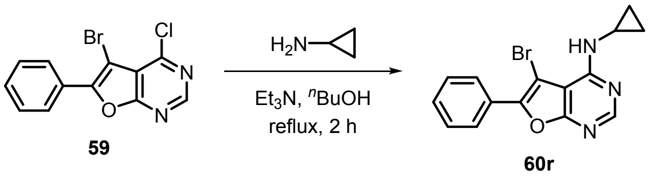

To a solution of 5-bromo-4-chloro-6-phenylfuro[2,3-*d*]pyrimidine (**59**) (200 mg, 0.65 mmol, 1.0 equiv) in *n*-butanol (5.0 mL) was added cyclopropylamine (50 μL, 0.72 mmol, 1.2 equiv.) and triethylamine (100 μL, 0.72 mmol, 1.1 equiv.) then the reaction mixture was stirred at reflux. After stirred for 2 hours, the reaction mixture was cooled down to room temperature, and the resulting precipitate was collected, washed with cold ethanol (5 mL) to yield the title compound **60r** (132 mg, 0.40 mmol, 62%) as yellow solid without further purification. LRMS (ESI) *m*/*z*: 330.1 $[M+H]^+$.

(1*R*,2*S*)-2-[(5-Bromo-6-phenylfuro[2,3-*d*]pyrimidin-4-yl)amino]cyclopentanol (60t).

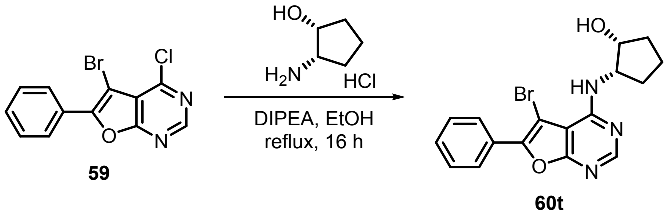

To a solution of 5-bromo-4-chloro-6-phenylfuro[2,3-*d*]pyrimidine (**59**) (100 mg, 0.32 mmol, 1.0 equiv) in ethanol (1.7 mL) was added (1*R*,2*S*)-*cis*-2-aminocyclopentanol hydrochloride (89 mg, 0.65 mmol, 2.0 equiv.) and DIPEA (230 μL, 1.32 mmol, 1.4 equiv.) then the reaction mixture was stirred at reflux. After stirred for 16 hours, the reaction mixture was cooled down to room temperature, concentrated *in vacuo*, and purified by flash chromatography (33% ethyl acetate in hexane) to yield the title compound **60t** (111 mg, 0.30 mmol, 92%) as white solid. LRMS (ESI) *m*/*z*: 374.0 $[M+H]^+$.

(1*R*,2*R*)-2-[(5-Bromo-6-phenylfuro[2,3-*d*]pyrimidin-4-yl)amino]cyclopentanol (60u).

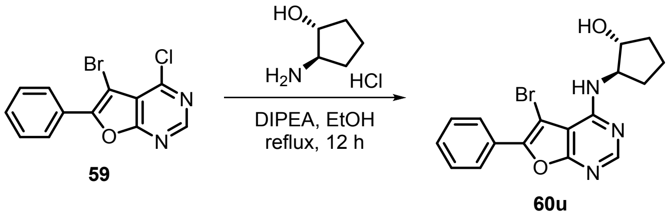

To a solution of 5-bromo-4-chloro-6-phenylfuro[2,3-*d*]pyrimidine (**59**) (150 mg, 0.48 mmol, 1.0 equiv) in ethanol (2.0 mL) was added (1*R*,2*R*)-*trans*-2-aminocyclopentanol hydrochloride (100 mg, 0.73 mmol, 1.5 equiv.) and triethylamine (200 μL, 1.43 mmol, 3.0 equiv.) then the reaction mixture was stirred at reflux. After stirred for 12 hours, the reaction mixture was cooled down to room temperature, concentrated *in vacuo*. Then the mixture was dissolved in ethyl acetate (10 mL), washed with water (10 mL) and brine (10 mL). The combined organic layers were dried over $MgSO_4$, concentrated *in vacuo*, and purified by flash chromatography (40–45% ethyl acetate in hexane) to yield the title compound **60u** (175 mg, 0.47 mmol, 97%) as white solid. LRMS (ESI) *m*/*z*: 374.0 $[M+H]^+$.

3-[(5-Bromo-6-phenylfuro[2,3-*d*]pyrimidin-4-yl)amino]cyclobutanol (60v).

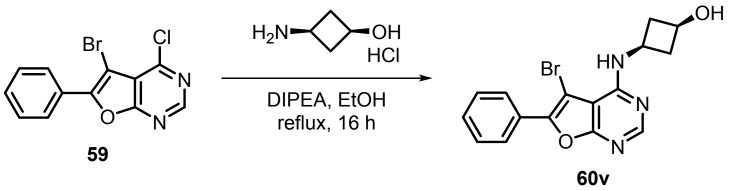

To a solution of 5-bromo-4-chloro-6-phenylfuro[2,3-*d*]pyrimidine (**59**) (80 mg, 0.26 mmol, 1.0 equiv) in ethanol (1.3 mL) was added *cis*-3-aminocyclobutanol hydrochloride (64 mg, 0.52 mmol, 2.0 equiv.) and DIPEA (180 μL, 1.03 mmol, 4.0 equiv.) then the reaction mixture was stirred at reflux. After stirred for 16 hours, the reaction mixture was cooled down to room temperature, concentrated *in vacuo*, and purified by flash chromatography (50% ethyl acetate in hexane) to yield the title compound **60v** (90 mg, 0.25 mmol, 97%) as white solid. LRMS (ESI) *m*/*z*: 360.0 $[M+H]^+$.

3-[(5-Bromo-6-phenylfuro[2,3-*d*]pyrimidin-4-yl)amino]cyclobutanol (60w).

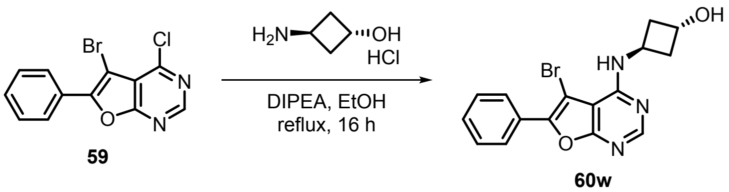

To a solution of 5-bromo-4-chloro-6-phenylfuro[2,3-*d*]pyrimidine (**59**) (80 mg, 0.26 mmol, 1.0 equiv) in ethanol (1.3 mL) was added *trans*-3-aminocyclobutanol hydrochloride (64 mg, 0.52 mmol, 2.0 equiv.) and DIPEA (180 μL, 1.03 mmol, 4.0 equiv.) then the reaction mixture was stirred at reflux. After stirred for 16 hours, the reaction mixture was cooled down to room temperature, concentrated *in vacuo*, and purified by flash chromatography (50% ethyl acetate in hexane) to yield the title compound **60w** (91 mg, 0.25 mmol, 98%) as white solid. LRMS (ESI) *m*/*z*: 360.0 $[M+H]^+$.

(2*S*)-3-Methyl-2-{[5-(3-nitrophenyl)-6-phenylfuro[2,3-*d*]pyrimidin-4-yl]amino}butan-1-ol (61a).

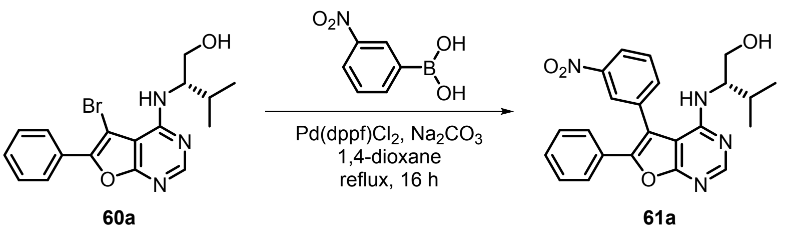

To a solution of **60a** (698 mg, 1.86 mmol, 1.0 equiv.) in 1,4-dioxane (5.0 mL) was added 3-nitrophenylboronic acid (151 mg, 0.90 mmol, 0.49 equiv.), Pd(dppf)Cl_2 (136

mg, 0.18 mmol, 10 mol%) and 2.0 M $Na_2CO_{3(aq)}$ (5.0 mL). The reaction mixture was degassed for 30 minutes, refilled with argon and stirred at reflux. After stirred for 16 hours, the reaction mixture was cooled down to room temperature, quenched with water and extracted into dichloromethane (10 mL × 3). The combined organic layers were dried over $MgSO_4$, concentrated *in vacuo* and purified by flash column chromatography (0–10% methanol in dichloromethane) to yield the title compound **61a** (388 mg, 0.93 mmol, 50%) as yellow solid. LRMS (ESI) *m/z*: 419.2 $[M+H]^+$.

(2*S*)-2-{[5-(3-Nitrophenyl)-6-phenylfuro[2,3-*d*]pyrimidin-4-yl]amino}propan-1-ol (61b).

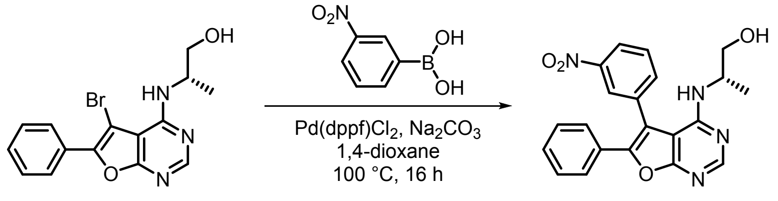

To a solution of **60b** (600 mg, 1.72 mmol, 1.0 equiv.) in 1,4-dioxane (8.0 mL) was added 3-nitrophenylboronic acid (431 mg, 2.58 mmol, 1.5 equiv.), Pd(dppf)Cl_2 (140 mg, 0.17 mmol, 10 mol%) and 2.0 M $Na_2CO_{3(aq)}$ (4.0 mL). The reaction mixture was degassed for 30 minutes, refilled with argon and stirred at 100 °C. After stirred for 16 hours, the reaction mixture was cooled down to room temperature, quenched with water and extracted into dichloromethane (10 mL × 3). The combined organic layers were dried over $MgSO_4$, concentrated *in vacuo* and purified by flash column chromatography (0–10% methanol in dichloromethane) to yield the title compound **61b** (480 mg, 1.23 mmol, 71%) as yellow solid. LRMS (ESI) *m/z*: 391.2 $[M+H]^+$.

2-{[5-(3-Nitrophenyl)-6-phenylfuro[2,3-*d*]pyrimidin-4-yl]amino}ethanol (61c).

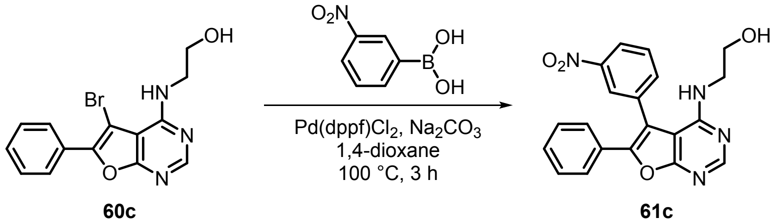

To a solution of **60c** (670 mg, 2.00 mmol, 1.0 equiv.) in 1,4-dioxane (5.0 mL) and H_2O (2.0 mL) was added 3-nitrophenylboronic acid (500 mg, 3.00 mmol, 1.5 equiv.), Pd(dppf)Cl_2 (163 mg, 0.20 mmol, 10 mol%) and 2.0 M $Na_2CO_{3(aq)}$ (5.0 mL). The reaction mixture was degassed for 30 minutes, refilled with argon and stirred at 100 °C. After stirred for 3 hours, the reaction mixture was cooled down to room temperature, filtered through Celite, concentrated *in vacuo*, washed with ethyl acetate (10 mL), added H_2O (10 mL), extracted into ethyl acetate (3 × 10 mL). The combined

organic layers were washed with brine (10 mL), dried over $MgSO_4$, concentrated *in vacuo* and purified by flash column chromatography (0–10% methanol in dichloromethane) to yield the title compound **61c** (395 mg, 1.05 mmol, 52%) as light yellow solid. LRMS (ESI) *m*/*z*: 377.1 $[M+H]^+$.

5-(3-Nitrophenyl)-*N*-(2-phenoxyethyl)-6-phenylfuro[2,3-*d*]pyrimidin-4-amine (61f).

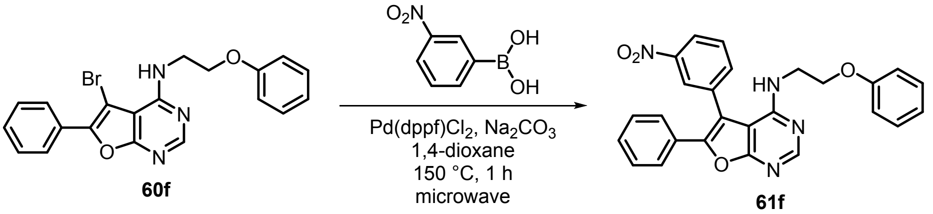

60f 61f

To a solution of **60f** (436 mg, 1.06 mmol, 1.0 equiv.) in *N*,*N*-dimethylformamide (5.0 mL) and H_2O (1.0 mL) was added 3-nitrophenylboronic acid (240 mg, 1.44 mmol, 1.4 equiv.), Pd(dppf)Cl_2 (80 mg, 0.11 mmol, 10 mol%) and Na_2CO_3 (400 mg, 2.89 mmol, 2.7 equiv.) then the reaction mixture was stirred at 150 °C under microwave irradiation. After stirred for 1 hour, the reaction mixture was cooled down to room temperature, filtered through Celite, concentrated *in vacuo* and purified by flash column chromatography (50% ethyl acetate in hexanes) to yield the title compound **61f** (289 mg, 0.64 mmol, 60%) as white solid. LRMS (ESI) *m*/*z*: 453.1 $[M+H]^+$.

Ethyl 3-{[5-(3-nitrophenyl)-6-phenylfuro[2,3-*d*]pyrimidin-4-yl]amino}propanoate (61g).

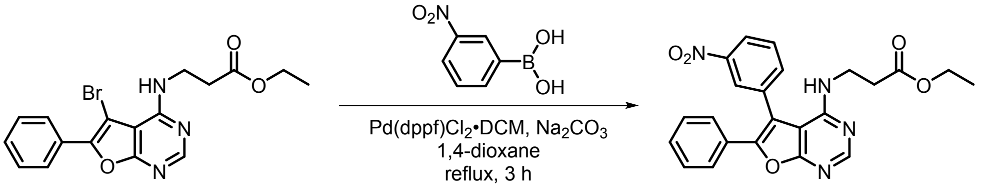

60g 61g

To a solution of **60g** (1000 mg, 2.56 mmol, 1.0 equiv.) in 1,4-dioxane (15.0 mL) and H_2O (1.5 mL) was added 3-nitrophenylboronic acid (641 mg, 3.84 mmol, 1.5 equiv.), Pd(dppf)$Cl_2 \cdot CH_2Cl_2$ (209 mg, 0.26 mmol, 10 mol%) and $Na_2CO_{3(aq)}$ (407 mg, 3.83 mmol, 1.5 equiv.). The reaction mixture was degassed for 30 minutes, refilled with argon, and stirred at reflux. After stirred for 3 hours, the reaction mixture was cooled down to room temperature, filtered through Celite, concentrated *in vacuo*, added ethyl acetate (10 mL) and H_2O (10 mL), extracted into ethyl acetate (3 × 10 mL). The combined organic layers were washed with brine (10 mL), dried over $MgSO_4$, concentrated *in vacuo* and purified by flash column chromatography (25% ethyl

acetate in hexane) to yield the title compound **61g** (939 mg, 2.17 mmol, 85%) as light yellow solid. LRMS (ESI) *m*/*z*: 433.1 [M+H]$^+$.

***tert*-Butyl (2-{[5-(3-nitrophenyl)-6-phenylfuro[2,3-*d*]pyrimidin-4-yl]amino}ethyl)carbamate (61h').**

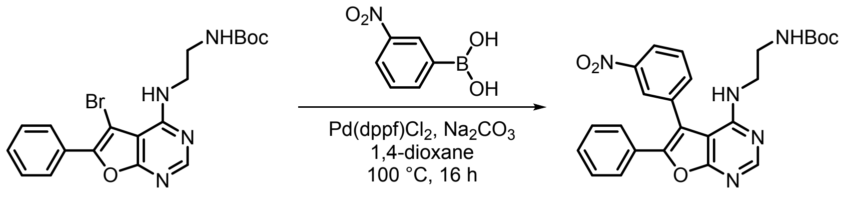

60h'

61h'

To a solution of **60h'** (393 mg, 0.91 mmol, 1.0 equiv.) in a mixture of 1,4-dioxane (5.0 mL) and H_2O (1.0 mL) was added 3-nitrophenylboronic acid (222 mg, 1.33 mmol, 1.5 equiv.), Pd(dppf)Cl_2 (332 mg, 0.45 mmol, 50 mol%) and Na_2CO_3 (144 mg, 1.36 mmol, 1.5 equiv.). The reaction mixture was degassed for 30 minutes, refilled with argon and stirred at 100 °C. After stirred for 16 hours, the reaction mixture was cooled down to room temperature, filtered through Celite, concentrated *in vacuo*, washed with ethyl acetate (10 mL), added H_2O (10 mL) and extracted into ethyl acetate (10 mL × 3). The organic layers were combined, washed with brine (10 mL), dried over $MgSO_4$, concentrated *in vacuo* and purified by flash column chromatography (0–5% methanol in dichloromethane) to yield the title compound **61h**' (327 mg, 0.69 mmol, 76%) as pale brown solid. LRMS (ESI) *m*/*z*: 476.1 [M+H]$^+$.

***N*^{1},*N*^{1}-Dimethyl-*N*^{2}-[5-(3-nitrophenyl)-6-phenylfuro[2,3-*d*]pyrimidin-4-yl]ethane-1,2-diamine (61i)**.

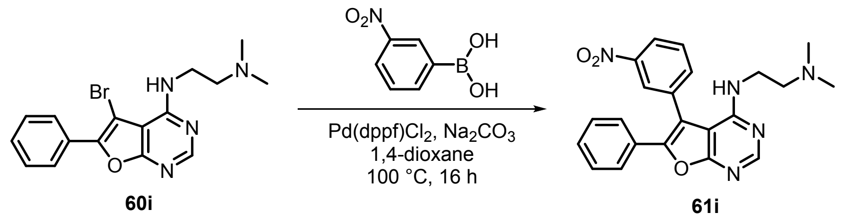

60i

61i

To a solution of **60i** (864 mg, 2.39 mmol, 1.0 equiv.) in a mixture of 1,4-dioxane (5.0 mL) and H_2O (1.0 mL) was added 3-nitrophenylboronic acid (598 mg, 3.58 mmol, 1.5 equiv.), Pd(dppf)Cl_2 (875 mg, 1.20 mmol, 50 mol%) and Na_2CO_3 (371 mg, 3.50 mmol, 1.5 equiv.). The reaction mixture was degassed for 30 minutes, refilled with argon and stirred at 100 °C. After stirred for 16 hours, the reaction mixture was cooled down to room temperature, filtered through Celite, concentrated *in vacuo*, washed with ethyl acetate (10.0 mL), added H_2O (10.0 mL) and extracted into ethyl acetate (3 × 10.0 mL). The organic layers were combined, washed with brine (10.0

mL), dried over $MgSO_4$, concentrated *in vacuo* and purified by flash column chromatography (1% methanol in dichloromethane) to yield the title compound **61i** (457 mg, 1.13 mmol, 47%) as pale brown solid. LRMS (ESI) *m*/*z*: 404.1 $[M+H]^+$.

***N*-Cyclopentyl-5-(4-fluoro-3-nitrophenyl)-6-phenylfuro[2,3-*d*]pyrimidin-4-amine (62j).**

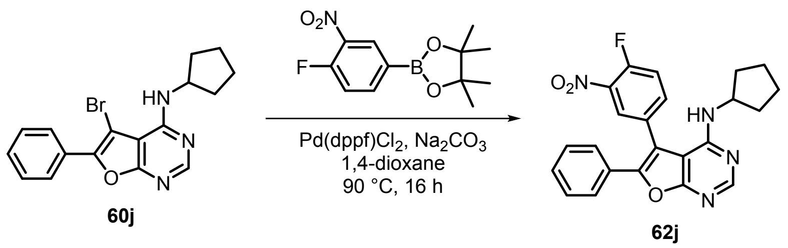

To a solution of **60j** (45 mg, 0.13 mmol, 1.0 equiv.) in 1,4-dioxane (5.0 mL) was added 2-(4-fluoro-3-nitrophenyl)-4,4,5,5-tetramethyl-1,3,2-dioxaborolane (50 mg, 0.19 mmol, 1.5 equiv.), Pd(dppf)Cl_2 (18 mg, 0.02 mmol, 20 mol%) and 2.0 M $Na_2CO_{3(aq)}$ (0.09 mL, 0.19 mmol, 1.5 equiv.). The reaction mixture was degassed for 30 minutes, refilled with argon and stirred at 90 °C. After stirred for 16 hours, the reaction mixture was cooled down to room temperature, filtered through Celite, extracted into ethyl acetate (10 mL × 3), and washed with brine (10 mL). The combined organic layers were dried over $MgSO_4$, concentrated *in vacuo*, and purified by flash chromatography (17–25% ethyl acetate in hexane) to yield the title compound **62j** (45 mg, 0.11 mmol, 86%) as yellow solid. LRMS (ESI) m/z: 419.1 $[M+H]^+$.

***N*-Cyclopropyl-5-(4-fluoro-3-nitrophenyl)-6-phenylfuro[2,3-*d*]pyrimidin-4-amine (62r).**

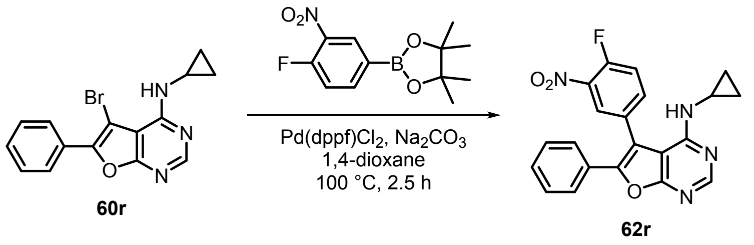

To a solution of **60r** (60 mg, 0.18 mmol, 1.0 equiv.) in 1,4-dioxane (2.0 mL) was added 2-(4-fluoro-3-nitrophenyl)-4,4,5,5-tetramethyl-1,3,2-dioxaborolane (73 mg, 0.27 mmol, 1.5 equiv.), Pd(dppf)Cl_2 (13 mg, 0.02 mmol, 10 mol%) and 2.0 M $Na_2CO_{3(aq)}$ (58 mg, 0.55 mmol, 3.0 equiv.). The reaction mixture was degassed for 30 minutes, refilled with argon and stirred at 100 °C. After stirred for 2.5 hours, the reaction mixture was cooled down to room temperature, filtered through Celite,

concentrated *in vacuo*, and purified by flash chromatography (2% methanol in dichloromethane with 0.1% NH_4OH) to yield the title compound **62r** (65 mg, 0.17 mmol, 92%) as yellow solid. LRMS (ESI) *m*/*z*: 391.2 $[M+H]^+$.

2-{[5-(4-Fluoro-3-nitrophenyl)-6-phenylfuro[2,3-*d*]pyrimidin-4-yl]amino}ethanol (62s).

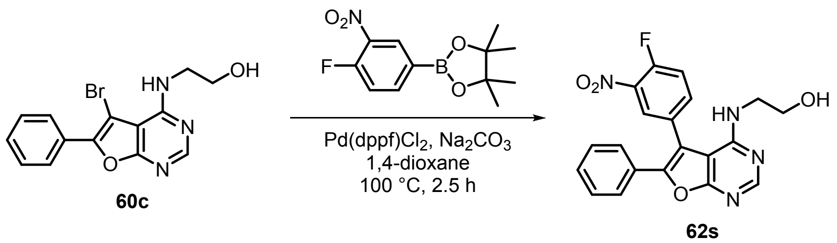

To a solution of **60c** (100 mg, 0.30 mmol, 1.0 equiv.) in 1,4-dioxane (5.0 mL) was added 2-(4-fluoro-3-nitrophenyl)-4,4,5,5-tetramethyl-1,3,2-dioxaborolane (120 mg, 0.45 mmol, 1.5 equiv.), Pd(dppf)Cl_2 (22 mg, 0.03 mmol, 10 mol%) and Na_2CO_3 (95 mg, 0.90 mmol, 3.0 equiv.). The reaction mixture was degassed for 30 minutes, refilled with argon and stirred at 100 °C. After stirred for 2.5 hours, the reaction mixture was cooled down to room temperature, filtered through Celite, concentrated *in vacuo*, and purified by flash chromatography (2% methanol in dichloromethane with 0.1% NH_4OH) to yield the title compound **62s** (110 mg, 0.28 mmol, 93%) as yellow solid. LRMS (ESI) *m*/*z*: 395.2 $[M+H]^+$.

(1*R*,2*R*)-2-{[5-(4-Fluoro-3-nitrophenyl)-6-phenylfuro[2,3-*d*]pyrimidin-4-yl]amino}cyclopentanol (62u).

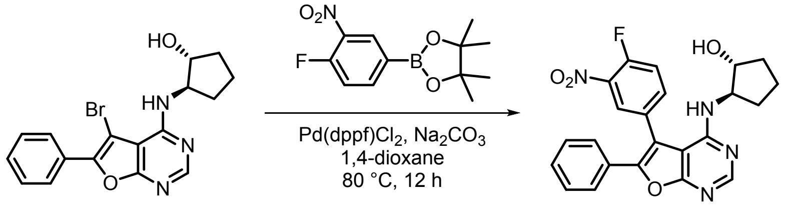

To a solution of **60u** (175 mg, 0.47 mmol, 1.0 equiv.) in 1,4-dioxane (3.0 mL) was added 2-(4-fluoro-3-nitrophenyl)-4,4,5,5-tetramethyl-1,3,2-dioxaborolane (187 mg, 0.70 mmol, 1.5 equiv.), Pd(dppf)Cl_2 (34 mg, 0.05 mmol, 10 mol%) and 2.0 M $Na_2CO_{3(aq)}$ (470 μL, 0.93 mmol, 2.0 equiv.). The reaction mixture was degassed for 30 minutes, refilled with argon and stirred at 80 °C. After stirred for 12 hours, the reaction mixture was cooled down to room temperature, filtered through Celite, concentrated *in vacuo*, and purified by flash chromatography (30–35% ethyl acetate in

hexane) to yield the title compound **62u** (150 mg, 0.35 mmol, 74%) as brown solid. LRMS (ESI) *m*/*z*: 435.1 $[M+H]^+$.

_N_-Cyclopentyl-5-[4-(morpholin-4-yl)-3-nitrophenyl]-6-phenylfuro[2,3-*d*]pyrimidin-4-amine (63j).

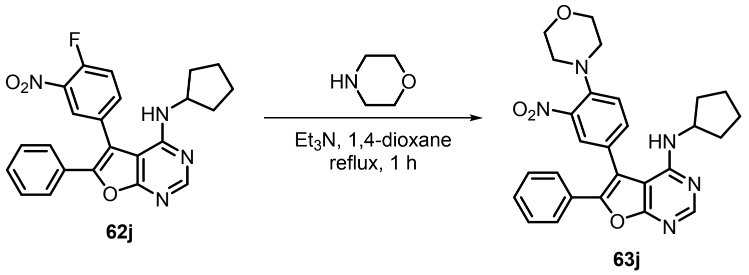

To a solution of **62j** (50 mg, 0.12 mmol, 1.0 equiv.) in 1,4-dioxane (3.0 mL) was added morpholine (50 mg, 0.35 mmol, 3.0 equiv.) and triethylamine (53 μL, 0.38 mmol, 3.2 equiv.) then the reaction mixture was stirred at reflux. After stirred for 1 hour, the reaction mixture was cooled down to room temperature, concentrated *in vacuo*, and purified by flash chromatography (17–25% ethyl acetate in hexane) to yield the title compound **63j** (49 mg, 0.10 mmol, 84%) as yellow solid. LRMS (ESI) m/z: 486.1 $[M+H]^+$.

_N_-Cyclopentyl-5-[4-(4-methylpiperazin-1-yl)-3-nitrophenyl]-6-phenylfuro[2,3-*d*]pyrimidin-4-amine (63k).

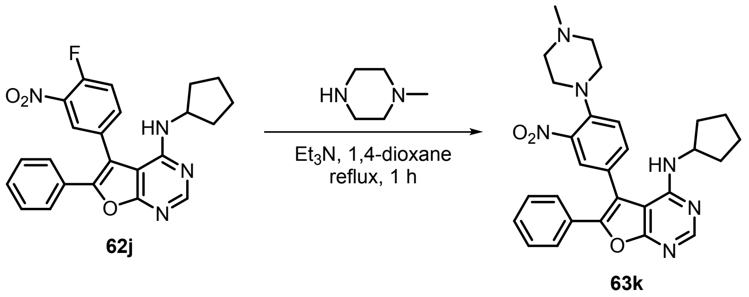

To a solution of **62j** (50 mg, 0.12 mmol, mmol) in 1,4-dioxane (5 mL) was added 1-methyl piperazine (40 μL, 0.36 mmol, 3.0 equiv.) and triethylamine (50 μL, 0.36 mmol, 3.0 equiv.) then the reaction mixture was stirred at reflux. After stirred for 1 hour, the reaction mixture was cooled down to room temperature, concentrated *in vacuo*, and purified by flash chromatography (5% methanol in dichloromethane with 0.1% NH_4OH) to yield the title compound **63k** (58 mg, 0.12 mmol, quant.) as yellow solid. LRMS (ESI) *m*/*z*: 499.1 $[M+H]^+$.

_N_-Cyclopentyl-5-{4-[2-(dimethylamino)ethoxy]-3-nitrophenyl}-6-phenylfuro[2,3-*d*]pyrimidin-4-amine (63l).

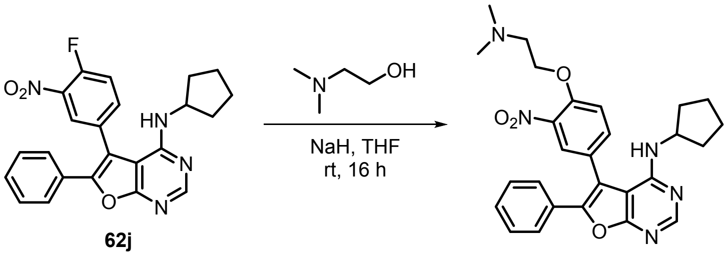

62j **63l**

To a solution of **62j** (30 mg, 0.07 mmol, 1.0 equiv.) in THF (2 mL) at 0 °C was NaH (4 mg, 0.10 mmol, 1.4 equiv.) then the reaction mixture was stirred at 0 °C. After stirred for 15 minutes, the reaction mixture was added 2-dimethylaminoethanol (14 μL, 0.07 mmol, 1.0 equiv.) then the reaction mixture was stirred at room temperature. After stirred for 16 hours, the reaction mixture was concentrated *in vacuo*, dissolved in dichloromethane, added 0.5 N $HCl_{(aq)}$ (20 mL), quenched with sat. $NaHCO_{3(aq)}$ (20 mL) and extracted into dichloromethane (10 mL × 3). The combined organic layers were washed with brine (10 mL), dried over $MgSO_4$, concentrated *in vacuo*, and purified by flash chromatography (5% methanol in dichloromethane with 0.1% NH_4OH) to yield the title compound **63l** (16 mg, 0.03 mmol, 46%) as yellow solid. LRMS (ESI) *m/z*: 488.3 $[M+H]^+$.

***N*-Cyclopentyl-5-{4-[(1-methylpiperidin-4-yl)oxy]-3-nitrophenyl}-6-phenylfuro[2,3-*d*]pyrimidin-4-amine (63m).**

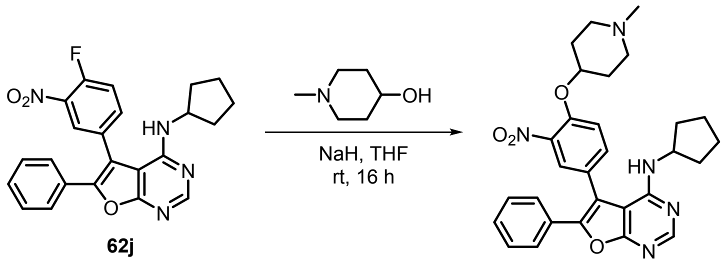

62j **63m**

To a solution of **62j** (30 mg, 0.07 mmol, 1.0 equiv.) in THF (2 mL) at 0 °C was added NaH (4 mg, 0.10 mmol, 1.4 equiv.) then the reaction mixture was stirred at 0 °C. After stirred for 15 minutes, the reaction mixture was added 1-methyl-4-piperidinol (9 mg, 0.08 mmol, 1.1 equiv.) then the reaction mixture was stirred at room temperature. After stirred for 16 hours, the reaction mixture was concentrated *in vacuo*, dissolved in dichloromethane, added 0.5 N $HCl_{(aq)}$ (20 mL), quenched with sat. $NaHCO_{3(aq)}$ (20 mL) and extracted into dichloromethane (10 mL × 3). The combined organic layers were washed with brine (10 mL), dried over $MgSO_4$, concentrated *in vacuo*, and purified by flash chromatography (5% methanol in dichloromethane with 0.1% NH_4OH) to yield the title compound **63m** (22 mg, 0.04 mmol, 60%) as yellow solid. LRMS (ESI) *m/z*: 514.3 $[M+H]^+$.

N^1-{4-[4-(Cyclopentylamino)-6-phenylfuro[2,3-*d*]pyrimidin-5-yl]-2-nitrophenyl}-N^1,N^2,N^2-trimethylethane-1,2-diamine (63n).

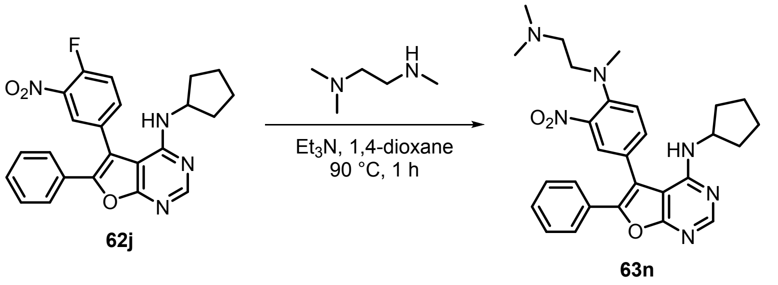

To a solution of **62j** (30 mg, 0.07 mmol, 1.0 equiv.) in 1,4-dioxane (3.0 mL) was added N^1,N^1,N^2-trimethylethane-1,2-diamine (28 µL, 0.22 mmol, 3.0 equiv.) and triethylamine (32 µL, 0.23 mmol, 3.2 equiv.) then the reaction mixture was stirred at 90 °C. After stirred for 1 hour, the reaction mixture was cooled down to room temperature, concentrated *in vacuo*, and purified by flash chromatography (2–5% methanol in dichloromethane) to yield the title compound **63n** (27 mg, 0.05 mmol, 75%) as orange gum. LRMS (ESI) *m*/*z*: 501.1 $[M+H]^+$.

N^1,N^1,N^2-Trimethyl-N^2-{4-[4-(methylamino)-6-phenylfuro[2,3-*d*]pyrimidin-5-yl]-2-nitrophenyl}ethane-1,2-diamine (63o).

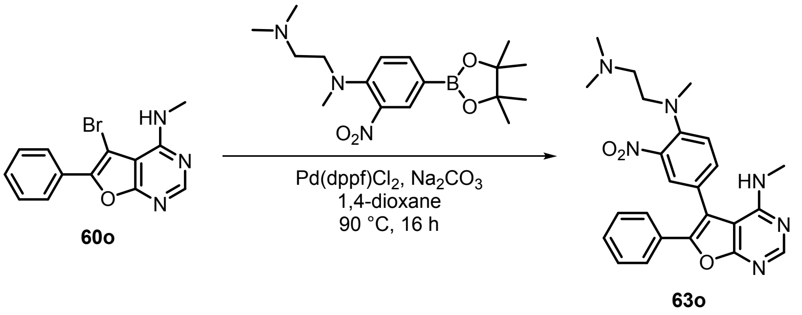

To a solution of **60o** (60 mg, 0.20 mmol, 1.0 equiv.) in 1,4-dioxane (2.0 mL) was added N^1,N^1,N^2-trimethyl-N^2-[4-(4,4,5,5-tetramethyl-1,3,2-dioxaborolan-2-yl)phenyl]ethane-1,2-diamine (76 mg, 0.22 mmol, 1.1 equiv.), $Pd(dppf)Cl_2$ (12 mg, 0.02 mmol, 8 mol%) and 2.0 M $Na_2CO_{3(aq)}$ (63 mg, 0.59 mmol, 3.0 equiv.). The reaction mixture was degassed for 30 minutes, refilled with argon and stirred at 80 °C. After stirred for 16 hours, the reaction mixture was cooled down to room temperature, filtered through Celite, concentrated *in vacuo*, and purified by flash chromatography (5% methanol in dichloromethane with 0.1% NH_4OH) to yield the title compound **63o** (59 mg, 0.13 mmol, 67%) as yellow solid. LRMS (ESI) *m*/*z*: 447.2 $[M+H]^+$.

N^1-{4-[4-(Ethylamino)-6-phenylfuro[2,3-*d*]pyrimidin-5-yl]-2-nitrophenyl}-N^1,N^2,N^2-trimethylethane-1,2-diamine (63p).

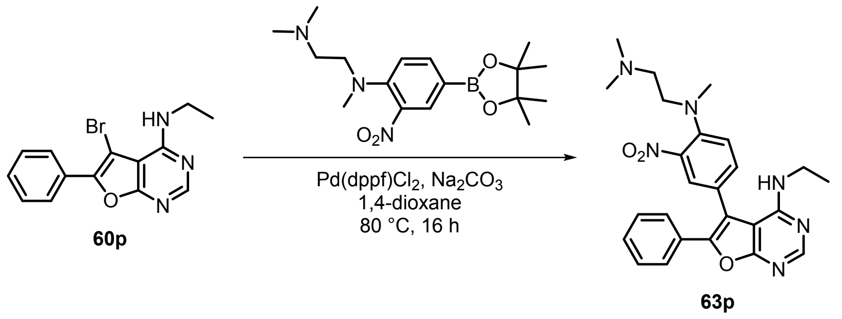

To a solution of **60p** (60 mg, 0.19 mmol, 1.0 equiv.) in 1,4-dioxane (2.0 mL) was added *N*[1],*N*[1],*N*[2]-trimethyl-*N*[2]-[4-(4,4,5,5-tetramethyl-1,3,2-dioxaborolan-2-yl)phenyl]ethane-1,2-diamine (72 mg, 0.21 mmol, 1.1 equiv.), Pd(dppf)Cl_2 (11 mg, 0.02 mmol, 8 mol%) and Na_2CO_3 (60 mg, 0.57 mmol, 3.0 equiv.). The reaction mixture was degassed for 30 minutes, refilled with argon and stirred at 80 °C. After stirred for 16 hours, the reaction mixture was cooled down to room temperature, filtered through Celite, concentrated *in vacuo*, and purified by flash chromatography (2–5% methanol in dichloromethane with 0.1% NH_4OH) to yield the title compound **63p** (63 mg, 0.14 mmol, 73%) as yellow solid. LRMS (ESI) *m*/*z*: 461.2 $[M+H]^+$.

***N*¹-{4-[4-(Ethylamino)-6-phenylfuro[2,3-*d*]pyrimidin-5-yl]-2-nitrophenyl}-*N*¹,*N*²,*N*²-trimethylethane-1,2-diamine (63q)**.

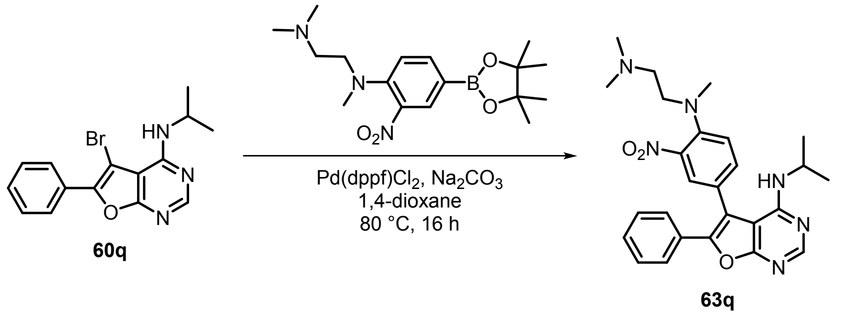

To a solution of **60q** (50 mg, 0.15 mmol, 1.0 equiv.) in 1,4-dioxane (2.0 mL) was added *N*[1],*N*[1],*N*[2]-trimethyl-*N*[2]-[4-(4,4,5,5-tetramethyl-1,3,2-dioxaborolan-2-yl)phenyl]ethane-1,2-diamine (58 mg, 0.17 mmol, 1.1 equiv.), Pd(dppf)Cl_2 (9 mg, 0.01 mmol, 8 mol%) and Na_2CO_3 (48 mg, 0.45 mmol, 3.0 equiv.). The reaction mixture was degassed for 30 minutes, refilled with argon and stirred at 80 °C. After stirred for 16 hours, the reaction mixture was cooled down to room temperature, filtered through Celite, concentrated *in vacuo*, and purified by flash chromatography (2–5% methanol in dichloromethane with 0.1% NH_4OH) to yield the title compound **63q** (50 mg, 0.11 mmol, 70%) as yellow solid. LRMS (ESI) *m*/*z*: 475.2 $[M+H]^+$.

***N*¹-{4-[4-(Cyclopropylamino)-6-phenylfuro[2,3-*d*]pyrimidin-5-yl]-2-nitrophenyl}-*N*¹,*N*²,*N*²-trimethylethane-1,2-diamine (63r).**

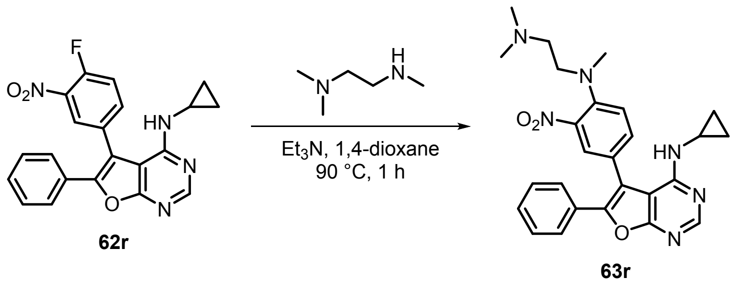

To a solution of **62r** (65 mg, 0.17 mmol, 1.0 equiv.) in 1,4-dioxane (2.0 mL) was added *N*[1],*N*[1],*N*[2]-trimethylethane-1,2-diamine (60 μL, 0.46 mmol, 2.8 equiv.) and triethylamine (70 μL, 0.50 mmol, 3.0 equiv.) then the reaction mixture was stirred at 90 °C. After stirred for 1 hour, the reaction mixture was cooled down to room temperature, concentrated *in vacuo*, and purified by flash chromatography (2–5% methanol in dichloromethane) to yield the title compound **63r** (59 mg, 0.12 mmol, 75%) as yellow solid. LRMS (ESI) *m*/*z*: 473.3 $[M+H]^+$.

2-{[5-(4-{[2-(Dimethylamino)ethyl](methyl)amino}-3-nitrophenyl)-6-phenylfuro[2,3-*d*]pyrimidin-4-yl]amino}ethanol (63s).

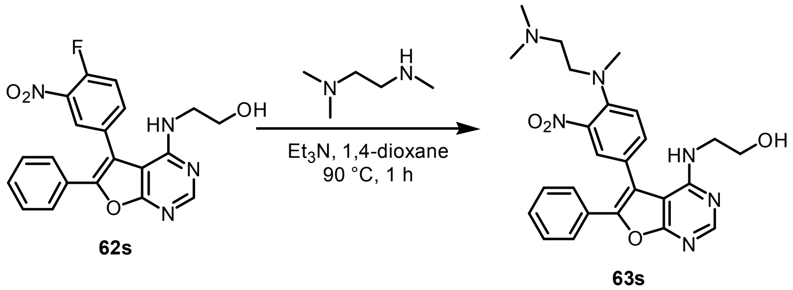

To a solution of **62s** (110 mg, 0.28 mmol, 1.0 equiv.) in 1,4-dioxane (5.0 mL) was added *N*[1],*N*[1],*N*[2]-trimethylethane-1,2-diamine (110 μL, 0.85 mmol, 3.0 equiv.) and triethylamine (120 μL, 0.86 mmol, 3.1 equiv.) then the reaction mixture was stirred at 90 °C. After stirred for 1 hour, the reaction mixture was cooled down to room temperature, concentrated *in vacuo*, and purified by flash chromatography (2–5% methanol in dichloromethane) to yield the title compound **63s** (108 mg, 0.23 mmol, 81%) as yellow solid. LRMS (ESI) *m*/*z*: 477.3 $[M+H]^+$.

(1*R*,2*S*)-2-{[5-(4-{[2-(Dimethylamino)ethyl](methyl)amino}-3-nitrophenyl)-6-phenylfuro[2,3-*d*]pyrimidin-4-yl]amino}cyclopentanol (63t).

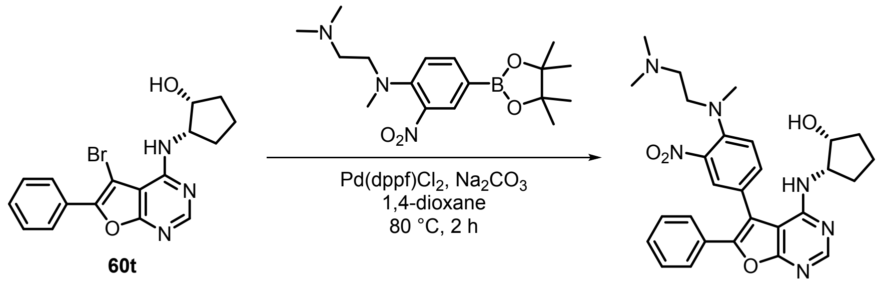

To a solution of **60t** (127 mg, 0.34 mmol, 1.0 equiv.) in 1,4-dioxane (2.1 mL) was added *N*[1],*N*[1],*N*[2]-trimethyl-*N*[2]-[4-(4,4,5,5-tetramethyl-1,3,2-dioxaborolan-2-yl)phenyl]ethane-1,2-diamine (178 mg, 0.51 mmol, 1.5 equiv.), Pd(dppf)Cl_2 (25 mg, 0.03 mmol, 10 mol%) and 2M $Na_2CO_{3(aq)}$ (370 μL, 0.75 mmol, 2.2 equiv.). The reaction mixture was degassed for 30 minutes, refilled with argon and stirred at 80 °C. After stirred for 2 hours, the reaction mixture was cooled down to room temperature, filtered through Celite, concentrated *in vacuo*, and purified by flash chromatography (3% methanol in dichloromethane) to yield the title compound **63t** (98 mg, 0.19 mmol, 56%) as yellow solid. LRMS (ESI) *m*/*z*: 517.2 $[M+H]^+$.

(1*R*,2*R*)-2-{[5-(4-{[2-(Dimethylamino)ethyl](methyl)amino}-3-nitrophenyl)-6-phenylfuro[2,3-*d*]pyrimidin-4-yl]amino}cyclopentanol (63u).

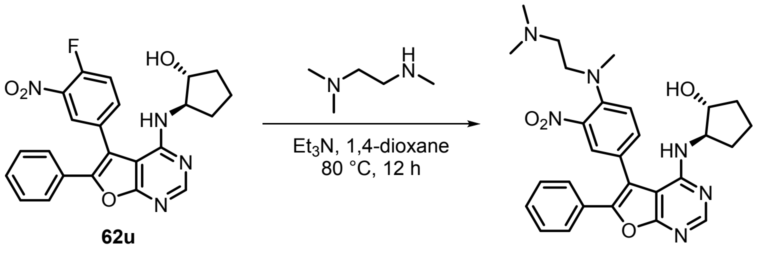

To a solution of **62u** (140 mg, 0.32 mmol, 1.0 equiv.) in 1,4-dioxane (5.0 mL) was added *N*[1],*N*[1],*N*[2]-trimethylethane-1,2-diamine (120 μL, 0.92 mmol, 2.9 equiv.) and triethylamine (150 μL, 1.08 mmol, 3.3 equiv.) then the reaction mixture was stirred at 80 °C. After stirred for 12 hours, the reaction mixture was cooled down to room temperature, concentrated *in vacuo*, and purified by flash chromatography (5–7% methanol in dichloromethane) to yield the title compound **63u** (140 mg, 0.27 mmol, 84%) as brown solid. LRMS (ESI) *m*/*z*: 517.2 $[M+H]^+$.

3-{[5-(4-{[2-(Dimethylamino)ethyl](methyl)amino}-3-nitrophenyl)-6-phenylfuro[2,3-*d*]pyrimidin-4-yl]amino}cyclobutanol (63v).

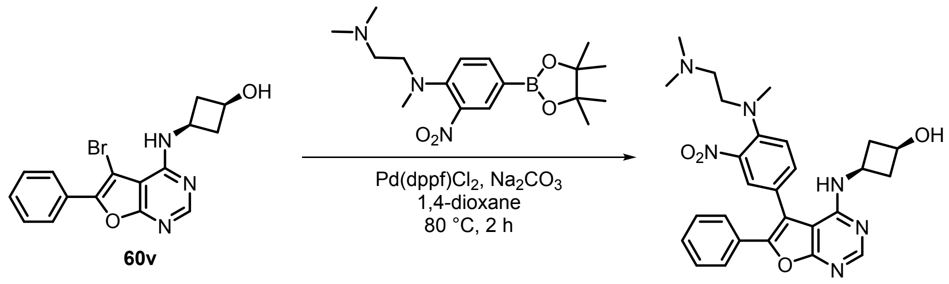

To a solution of **60v** (87 mg, 0.24 mmol, 1.0 equiv.) in 1,4-dioxane (1.5 mL) was added *N*[1],*N*[1],*N*[2]-trimethyl-*N*[2]-[4-(4,4,5,5-tetramethyl-1,3,2-dioxaborolan-2-yl)phenyl]ethane-1,2-diamine (127 mg, 0.36 mmol, 1.5 equiv.), Pd(dppf)Cl_2 (18 mg, 0.02 mmol, 10 mol%) and 2M $Na_2CO_{3(aq)}$ (270 μL, 0.53 mmol, 2.2 equiv.). The reaction mixture was degassed for 30 minutes, refilled with argon and stirred at 80 °C. After stirred for 2 hours, the reaction mixture was cooled down to room temperature, filtered through Celite, concentrated *in vacuo*, and purified by flash chromatography (3% methanol in dichloromethane) to yield the title compound **63v** (91 mg, 0.18 mmol, 75%) as yellow solid. LRMS (ESI) *m*/*z*: 503.2 $[M+H]^+$.

3-{[5-(4-{[2-(Dimethylamino)ethyl](methyl)amino}-3-nitrophenyl)-6-phenylfuro[2,3-*d*]pyrimidin-4-yl]amino}cyclobutanol (63w).

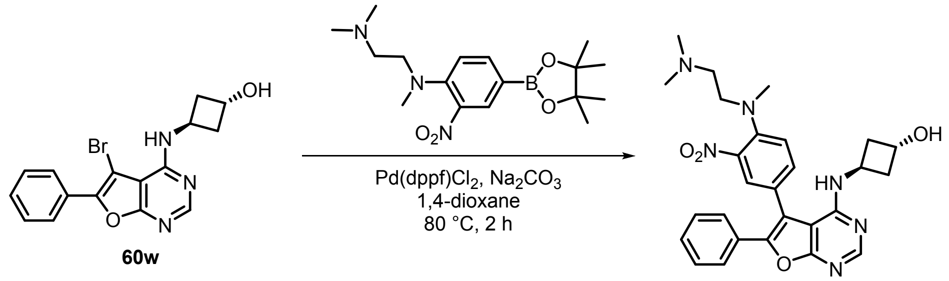

To a solution of **60w** (88 mg, 0.24 mmol, 1.0 equiv.) in 1,4-dioxane (1.5 mL) was added *N*[1],*N*[1],*N*[2]-trimethyl-*N*[2]-[4-(4,4,5,5-tetramethyl-1,3,2-dioxaborolan-2-yl)phenyl]ethane-1,2-diamine (128 mg, 0.37 mmol, 1.5 equiv.), Pd(dppf)Cl_2 (18 mg, 0.02 mmol, 10 mol%) and 2M $Na_2CO_{3(aq)}$ (270 μL, 0.54 mmol, 2.2 equiv.). The reaction mixture was degassed for 30 minutes, refilled with argon and stirred at 80 °C. After stirred for 2 hours, the reaction mixture was cooled down to room temperature, filtered through Celite, concentrated in vacuo, and purified by flash chromatography (3% methanol in dichloromethane) to yield the title compound **63w** (84 mg, 0.17 mmol, 68%) as yellow solid. LRMS (ESI) *m*/*z*: 503.2 $[M+H]^+$.

(2*S*)-2-{[5-(3-Aminophenyl)-6-phenylfuro[2,3-*d*]pyrimidin-4-yl]amino}-3-methylbutan-1-ol (64a).

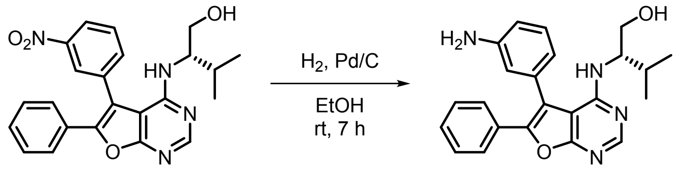

61a **64a**

To a solution of **61a** (582 mg, 1.39 mmol, 1.0 equiv.) in ethanol (5.0 mL) was added palladium on carbon (148 mg, 0.14 mmol, 10 mol%) then the reaction was stirred under an atmosphere of hydrogen at room temperature. After stirred for 7 hours, the reaction mixture was filtered through Celite, concentrated *in vacuo* and purified by flash column chromatography (2–15% methanol in dichloromethane) to yield the title compound **64a** (486 mg, 1.25 mmol, 90%) as a white solid. LRMS (ESI) *m*/*z*: 389.2 $[M+H]^+$.

(2*S*)-2-{[5-(3-Aminophenyl)-6-phenylfuro[2,3-*d*]pyrimidin-4-yl]amino}propan-1-ol (64b).

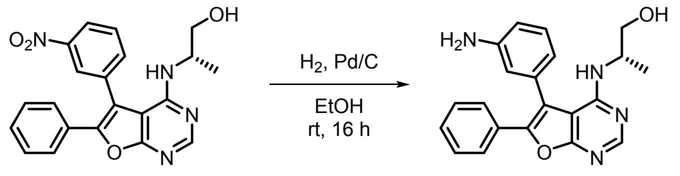

61b **64b**

To a solution of **61b** (450 mg, 1.45 mmol, 1.0 equiv.) in ethanol (50 mL) was added palladium on carbon (40 mg, 0.04 mmol, 31 mol%) then the reaction was stirred under an atmosphere of hydrogen at room temperature. After stirred for 16 hours, the reaction mixture was filtered through Celite, concentrated *in vacuo* and purified by flash column chromatography (0–10% methanol in dichloromethane) to yield the title compound **64b** (410 mg, 1.14 mmol, 99%) as light-yellow solid. LRMS (ESI) *m*/*z*: 361.2 $[M+H]^+$.

2-{[5-(3-Aminophenyl)-6-phenylfuro[2,3-*d*]pyrimidin-4-yl]amino}ethanol (64c).

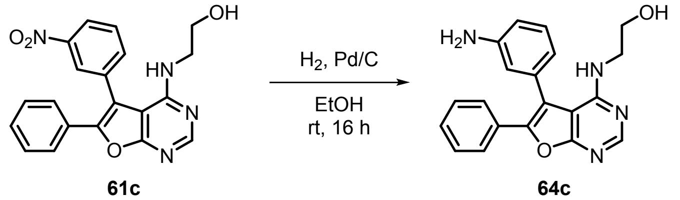

61c **64c**

To a solution of **61c** (300 mg, 0.80 mmol, 1.0 equiv.) in ethanol (20.0 mL) was added palladium on carbon (20 mg, 0.02 mmol, 2 mol%) then the reaction was stirred under an atmosphere of hydrogen at room temperature. After stirred for 16 hours, the reaction mixture was filtered through Celite, concentrated *in vacuo* and purified by

flash column chromatography (0–10% methanol in dichloromethane) to yield the title compound **64c** (254 mg, 0.73 mmol, 92%) as yellow solid. LRMS (ESI) *m*/*z*: 347.1 $[M+H]^+$.

3-{[5-(3-Aminophenyl)-6-phenylfuro[2,3-*d*]pyrimidin-4-yl]amino}propan-1-ol (64d).

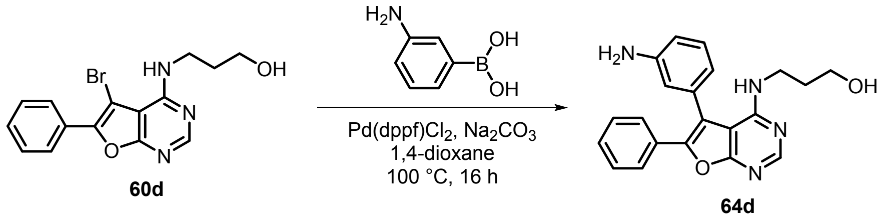

To a solution of **60d** (39 mg, 0.11 mmol, 1.0 equiv.) in 1,4-dioxane (1.0 mL) was added (3-aminophenyl)boronic acid (23 mg, 0.17 mmol, 1.5 equiv.), Pd(dppf)Cl_2 (9 mg, 0.01 mmol, 11 mol%) and 2.0 M $Na_2CO_{3(aq)}$ (220 μL, 0.45 mmol, 4.0 equiv.). The reaction mixture was degassed for 30 minutes, refilled with argon and stirred at 100 °C. After stirred for 16 hours, the reaction mixture was cooled down to room temperature, filtered through Celite, concentrated *in vacuo*, and purified by thin-plate chromatography (5% methanol in dichloromethane) to yield the title compound **64d** (36 mg, 0.10 mmol, 89%) as light yellow solid. LRMS (ESI) *m*/*z*: 361.1 $[M+H]^+$.

5-(3-Aminophenyl)-*N*-(2-methoxyethyl)-6-phenylfuro[2,3-*d*]pyrimidin-4-amine (64e).

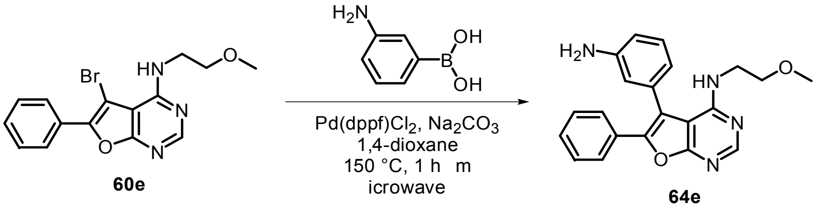

To a solution of **60e** (349 mg, 1.00 mmol, 1.0 equiv.) in 1,4-dioxane (5.0 mL) and H_2O (1.0 mL) was added 3-aminophenylboronic acid (249 mg, 1.49 mmol, 1.5 equiv.), Pd(dppf)Cl_2 (72 mg, 0.10 mmol, 10 mol%) and 2.0 M $Na_2CO_{3(aq)}$ (410 mg, 2.97 mmol, 3.0 equiv.). The reaction mixture was degassed for 30 minutes, refilled with argon, and stirred at 150 °C under microwave irradiation. After stirred for 1 hour, the reaction mixture was cooled down to room temperature, filtered through Celite, concentrated *in vacuo*, and purified by Combiflash automated flash chromatography (3% methanol in dichloromethane) to yield the title compound **64e** (150 mg, 0.42 mmol, 43%) as yellow solid. LRMS (ESI) *m*/*z*: 361.1 $[M+H]^+$.

5-(3-Aminophenyl)-*N*-(2-phenoxyethyl)-6-phenylfuro[2,3-*d*]pyrimidin-4-amine (64f).

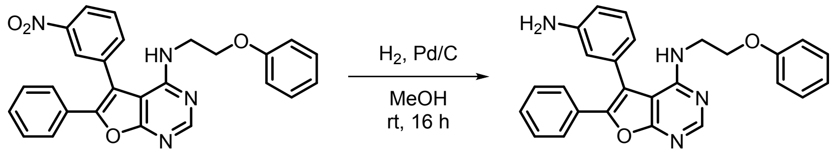

61f **64f**

To a solution of **61f** (289 mg, 0.64 mmol, 1.0 equiv.) in methanol (25.0 mL) was added palladium on carbon (60 mg, 0.06 mmol, 9 mol%) then the reaction was stirred under an atmosphere of hydrogen at room temperature. After stirred for 16 hours, the reaction mixture was filtered through Celite, concentrated *in vacuo* and purified by flash column chromatography (50% ethyl acetate in hexanes with 2.5% triethylamine) to yield the title compound **64f** (80 mg, 0.19 mmol, 30%) as yellow solid. LRMS (ESI) *m*/*z*: 423.1 $[M+H]^+$.

Ethyl 3-{[5-(3-aminophenyl)-6-phenylfuro[2,3-*d*]pyrimidin-4-yl]amino}propanoate (64g).

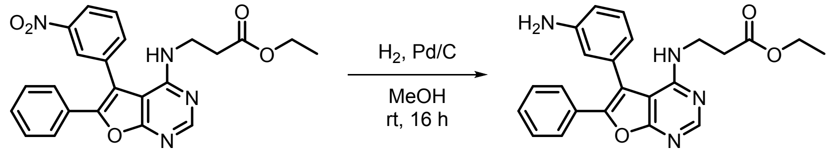

61g **64g**

To a solution of **61g** (938 mg, 2.17 mmol, 1.0 equiv.) in methanol (30 mL) was added palladium on carbon (120 mg, 0.11 mmol, 5 mol%) then the reaction was stirred under an atmosphere of hydrogen at room temperature. After stirred for 16 hours, the reaction mixture was filtered through Celite, concentrated *in vacuo* to yield the title compound **64g** (861 mg, 2.14 mmol, 99%) as brown solid. LRMS (ESI) *m*/*z*: 403.1 $[M+H]^+$.

***tert*-Butyl (2-{[5-(3-aminophenyl)-6-phenylfuro[2,3-*d*]pyrimidin-4-yl]amino}ethyl)carbamate (64h').**

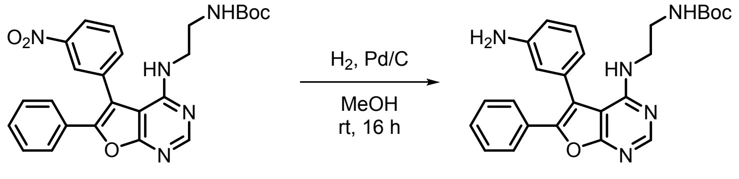

61h' **64h'**

To a solution of **61h'** (322 mg, 0.68 mmol, 1.0 equiv.) in methanol (5.0 mL) was added palladium on carbon (75 mg, 0.07 mmol, 10 mol%) then the reaction was stirred under an atmosphere of hydrogen at room temperature. After stirred for 16 hours, the reaction mixture was filtered through Celite, concentrated *in vacuo* to yield

the title compound **64h'** (298 mg, 0.67 mmol, 99%) as pale brown solid. LRMS (ESI) *m*/*z*: 446.2 $[M+H]^+$.

N^1-[5-(3-Aminophenyl)-6-phenylfuro[2,3-*d*]pyrimidin-4-yl]-N^2,N^2-dimethylethane-1,2-diamine (64i).

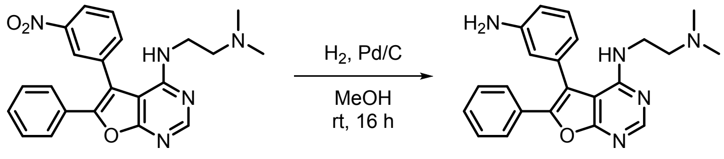

61i **64i**

To a solution of **61i** (457 mg, 1.13 mmol, 1.0 equiv.) in methanol (10.0 mL) was added palladium on carbon (121 mg, 0.11 mmol, 10 mol%) then the reaction was stirred under an atmosphere of hydrogen at room temperature. After stirred for 16 hours, the reaction mixture was filtered through Celite, concentrated *in vacuo* to yield the title compound **64i** (324 mg, 0.87 mmol, 77%) as pale brown solid. LRMS (ESI) *m*/*z*: 374.1 $[M+H]^+$.

5-[3-Amino-4-(morpholin-4-yl)phenyl]-*N*-cyclopentyl-6-phenylfuro[2,3-*d*]pyrimidin-4-amine (64j).

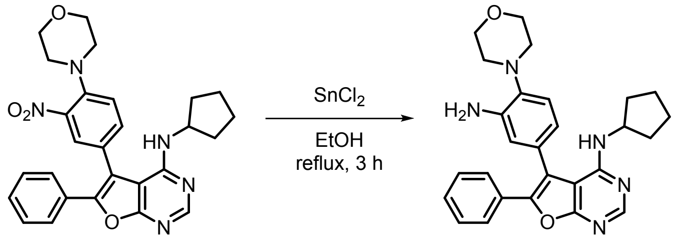

63j **64j**

To a solution of **63j** (49 mg, 0.10 mmol, 1.0 equiv.) in ethanol (3.0 mL) was added tin(II) chloride (46 mg, 0.20 mmol, 2.0 equiv.) then the reaction mixture was stirred at reflux. After stirred for 3 hours, the reaction mixture was cooled down to room temperature, filtered through Celite, and concentrated *in vacuo*. Then the mixture was dissolved in ethyl acetate (10 mL), washed with $NaHCO_{3(aq)}$ (20 mL) and brine (10 mL). The combined organic layers were dried over $MgSO_4$, concentrated *in vacuo* to yield the title compound **64j** (43 mg, 0.10 mmol, 94%) as yellow solid. LRMS (ESI) *m*/*z*: 456.1 $[M+H]^+$.

5-[3-Amino-4-(4-methylpiperazin-1-yl)phenyl]-*N*-cyclopentyl-6-phenylfuro[2,3-*d*]pyrimidin-4-amine (64k).

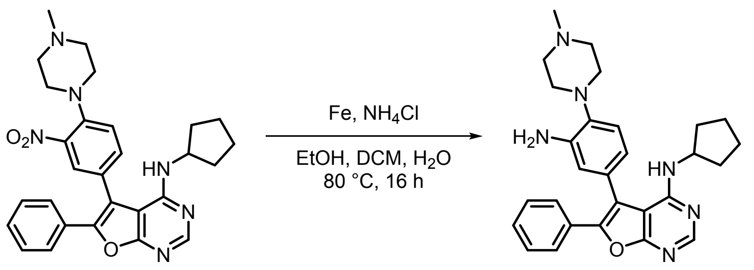

63k **64k**

To a solution of **63k** (58 mg, 0.12 mmol, 1.0 equiv.) in ethanol (3 mL), dichloromethane (3 mL) and water (0.6 mL) was added iron powder (19 mg, 0.34 mmol, 3.0 equiv.) and sat. $NH_4Cl_{(aq)}$ (0.3 mL) then the reaction mixture was stirred at 80 °C. After stirred for 16 hours, the reaction mixture was cooled down to room temperature, filtered through Celite, and concentrated *in vacuo*. Then the mixture was dissolved in dichloromethane (10 mL), washed with $NaHCO_{3(aq)}$ (20 mL) and brine (10 mL). The combined organic layers were dried over $MgSO_4$, concentrated *in vacuo*, and purified by flash chromatography (3–5% methanol in dichloromethane with 0.1% NH_4OH) to yield the title compound **64k** (51 mg, 0.11 mmol, 94%) as yellow solid. LRMS (ESI) *m*/*z*: 469.4 $[M+H]^+$.

5-{3-Amino-4-[2-(dimethylamino)ethoxy]phenyl}-*N*-cyclopentyl-6-phenylfuro[2,3-*d*]pyrimidin-4-amine (64l).

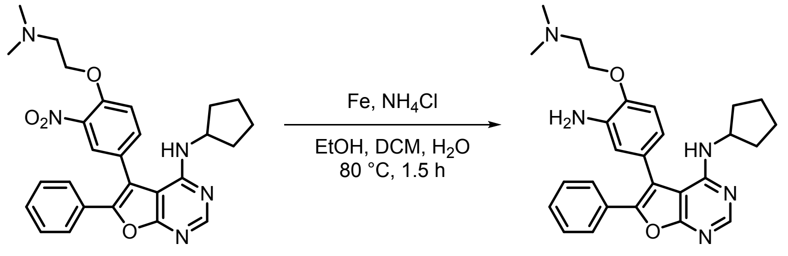

63l **64l**

To a solution of **63l** (16 mg, 0.03 mmol, 1.0 equiv.) in ethanol (3 mL), dichloromethane (3 mL) and water (0.6 mL) was added iron powder (5 mg, 0.09 mmol, 2.9 equiv.) and sat. $NH_4Cl_{(aq)}$ (0.3 mL) then the reaction mixture was stirred at 80 °C. After stirred for 1.5 hours, the reaction mixture was cooled down to room temperature, filtered through Celite, and concentrated *in vacuo*. Then the mixture was dissolved in dichloromethane (10 mL), washed with $NaHCO_{3(aq)}$ (20 mL) and brine (10 mL). The combined organic layers were dried over MgSO4, concentrated *in vacuo*, and purified by flash chromatography (5% methanol in dichloromethane with 0.1% NH_4OH) to yield the title compound **64l** (14 mg, 0.03 mmol, 93%) as yellow solid. LRMS (ESI) *m*/*z*: 469.4 $[M+H]^+$.

5-{3-Amino-4-[(1-methylpiperidin-4-yl)oxy]phenyl}-*N*-cyclopentyl-6-phenylfuro[2,3-*d*]pyrimidin-4-amine (64m).

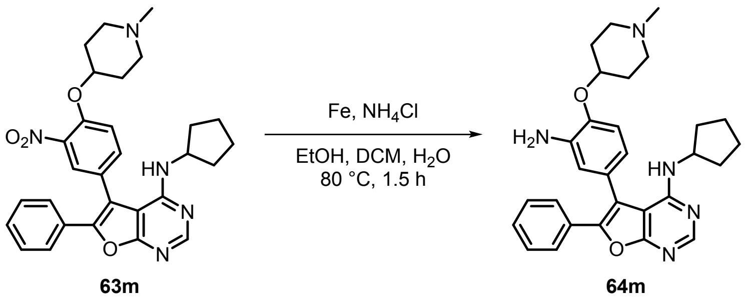

To a solution of **63m** (22 mg, 0.04 mmol, 1.0 equiv.) in ethanol (3 mL), dichloromethane (3 mL) and water (0.6 mL) was added iron powder (7 mg, 0.13 mmol, 2.9 equiv.) and sat. $NH_4Cl_{(aq)}$ (0.3 mL) then the reaction mixture was stirred at 80 °C. After stirred for 1.5 hours, the reaction mixture was cooled down to room temperature, filtered through Celite, and concentrated *in vacuo*. Then the mixture was dissolved in dichloromethane (10 mL), washed with $NaHCO_{3(aq)}$ (20 mL) and brine (10 mL). The combined organic layers were dried over $MgSO_4$, concentrated *in vacuo*, and purified by flash chromatography (5% methanol in dichloromethane with 0.1% NH4OH) to yield the title compound **64m** (16 mg, 0.03 mmol, 77%) as yellow solid. LRMS (ESI) *m*/*z*: 484.3 $[M+H]^+$.

4-[4-(Cyclopentylamino)-6-phenylfuro[2,3-*d*]pyrimidin-5-yl]-*N*^{1}-[2-(dimethylamino)ethyl]-*N*^{1}-methylbenzene-1,2-diamine (64n).

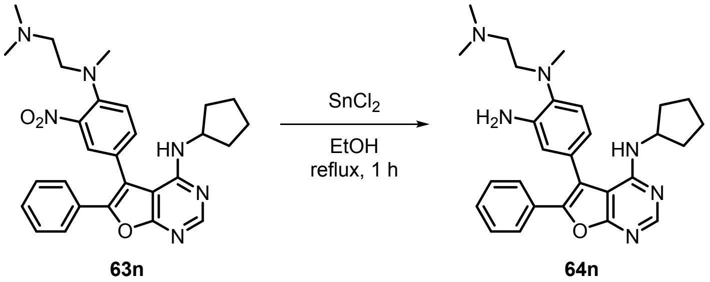

To a solution of **63n** (27 mg, 0.05 mmol, 1.0 equiv.) in ethanol (5.0 mL) was added tin(II) chloride (24 mg, 0.11 mmol, 2.0 equiv.) then the reaction mixture was stirred at reflux. After stirred for 1 hour, the reaction mixture was cooled down to room temperature, filtered through Celite, and concentrated *in vacuo*. Then the mixture was dissolved in ethyl acetate (10 mL), washed with $NaHCO_{3(aq)}$ (20 mL) and brine (10 mL). The combined organic layers were dried over $MgSO_4$, concentrated *in vacuo* to yield the title compound **64n** (22 mg, 0.05 mmol, 87%) as light yellow solid. LRMS (ESI) *m*/*z*: 471.1 $[M+H]^+$.

***N*¹-[2-(Dimethylamino)ethyl]-*N*¹-methyl-4-[4-(methylamino)-6-phenylfuro[2,3-*d*]pyrimidin-5-yl]benzene-1,2-diamine (64o).**

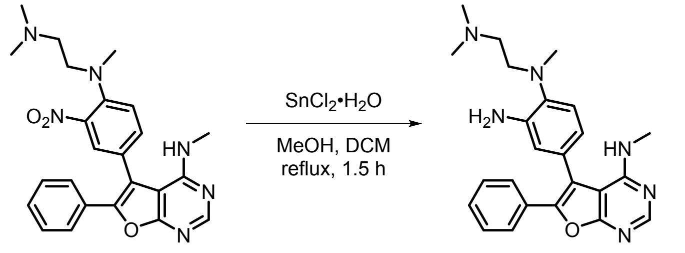

63o **64o**

To a solution of **63o** (59 mg, 0.14 mmol, 1.0 equiv.) in methanol (3.0 mL) and dichloromethane (3.0 mL) was added $SnCl_2 \cdot 2H_2O$ (123 mg, 0.55 mmol, 3.8 equiv.) then the reaction mixture was stirred at reflux. After stirred for 1.5 hours, the reaction mixture was cooled down to room temperature, filtered through Celite, and concentrated *in vacuo*. Then the mixture was dissolved in dichloromethane (10 mL), washed with $NaHCO_{3(aq)}$ (20 mL) and brine (10 mL). The combined organic layers were dried over $MgSO_4$, concentrated *in vacuo*, and purified by flash chromatography (2–5% methanol in dichloromethane with 0.1% NH_4OH) to yield the title compound **64o** (26 mg, 0.06 mmol, 47%) as yellow solid. LRMS (ESI) *m*/*z*: 417.2 $[M+H]^+$.

***N*¹-[2-(Dimethylamino)ethyl]-4-[4-(ethylamino)-6-phenylfuro[2,3-*d*]pyrimidin-5-yl]-*N*¹-methylbenzene-1,2-diamine (64p).**

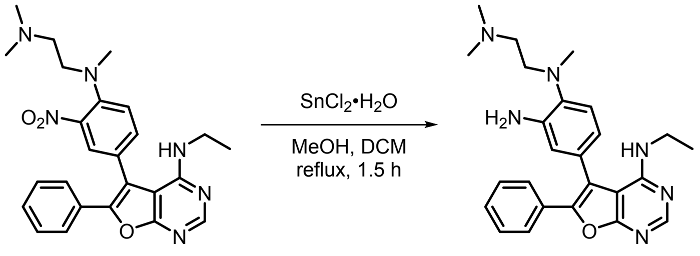

63p **64p**

To a solution of **63p** (63 mg, 0.14 mmol, 1.0 equiv.) in methanol (3.0 mL) and dichloromethane (3.0 mL) was added $SnCl_2 \cdot 2H_2O$ (117 mg, 0.52 mmol, 3.8 equiv.) then the reaction mixture was stirred at reflux. After stirred for 1.5 hours, the reaction mixture was cooled down to room temperature, filtered through Celite, and concentrated *in vacuo*. Then the mixture was dissolved in dichloromethane (10 mL), washed with $NaHCO_{3(aq)}$ (20 mL) and brine (10 mL). The combined organic layers were dried over $MgSO_4$, concentrated *in vacuo*, and purified by flash chromatography (2–5% methanol in dichloromethane with 0.1% NH_4OH) to yield the title compound **64p** (18 mg, 0.04 mmol, 31%) as yellow solid. LRMS (ESI) *m*/*z*: 431.2 $[M+H]^+$.

***N*¹-[2-(Dimethylamino)ethyl]-*N*¹-methyl-4-[6-phenyl-4-(propan-2-ylamino)furo[2,3-*d*]pyrimidin-5-yl]benzene-1,2-diamine (64q).**

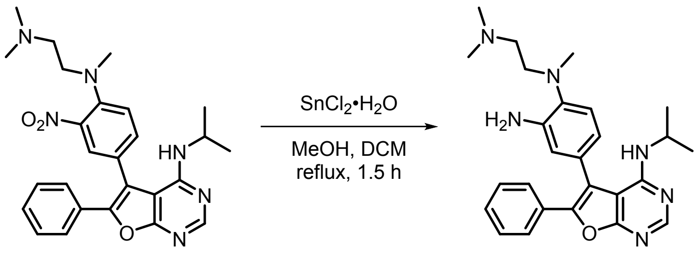

63q **64q**

To a solution of **63q** (50 mg, 0.11 mmol, 1.0 equiv.) in methanol (3.0 mL) and dichloromethane (3.0 mL) was added $SnCl_2 \cdot 2H_2O$ (95 mg, 0.42 mmol, 4.0 equiv.) then the reaction mixture was stirred at reflux. After stirred for 1.5 hours, the reaction mixture was cooled down to room temperature, filtered through Celite, and concentrated *in vacuo*. Then the mixture was dissolved in dichloromethane (10 mL), washed with $NaHCO_{3(aq)}$ (20 mL) and brine (10 mL). The combined organic layers were dried over $MgSO_4$, concentrated *in vacuo*, and purified by flash column chromatography (2–5% methanol in dichloromethane with 0.1% NH_4OH) to yield the title compound **64q** (19 mg, 0.04 mmol, 41%) as yellow solid. LRMS (ESI) *m*/*z*: 445.2 $[M+H]^+$.

4-[4-(Cyclopropylamino)-6-phenylfuro[2,3-*d*]pyrimidin-5-yl]-*N*^{1}-[2-(dimethylamino)ethyl]-*N*^{1}-methylbenzene-1,2-diamine (64r).

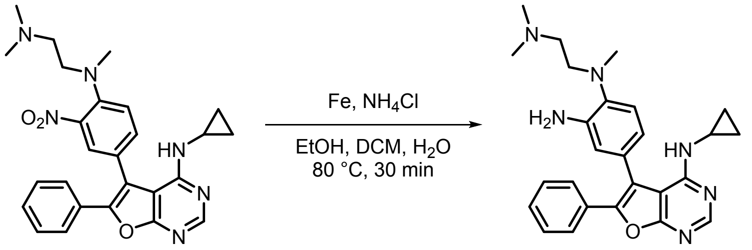

63r **64r**

To a solution of **63r** (59 mg, 0.12 mmol, 1.0 equiv.) in ethanol (3 mL), dichloromethane (3 mL) and water (0.3 mL) was added iron powder (21 mg, 0.38 mmol, 3.0 equiv.) and sat. $NH_4Cl_{(aq)}$ (0.3 mL) then the reaction mixture was stirred at 80 °C. After stirred for 30 minutes, the reaction mixture was cooled down to room temperature, filtered through Celite, and concentrated *in vacuo*. Then the mixture was dissolved in dichloromethane (10 mL), washed with $NaHCO_{3(aq)}$ (20 mL) and brine (10 mL). The combined organic layers were dried over $MgSO_4$, concentrated *in vacuo* to yield the title compound **64r** (29 mg, 0.07 mmol, 52%) as brown oil without further purification. LRMS (ESI) *m*/*z*: 443.3 $[M+H]^+$.

2-{[5-(3-Amino-4-{[2-(dimethylamino)ethyl](methyl)amino}phenyl)-6-phenylfuro[2,3-*d*]pyrimidin-4-yl]amino}ethanol (64s).

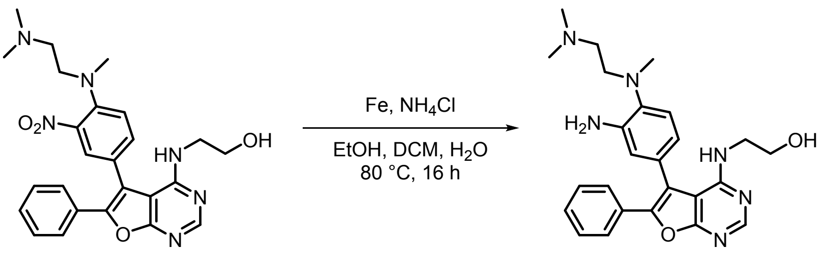

To a solution of **63s** (100 mg, 0.21 mmol, 1.0 equiv.) in ethanol (3 mL), dichloromethane (3 mL) and water (0.6 mL) was added iron powder (35 mg, 0.63 mmol, 3.0 equiv.) and sat. $NH_4Cl_{(aq)}$ (0.3 mL) then the reaction mixture was stirred at 80 °C. After stirred for 16 hours, the reaction mixture was cooled down to room temperature, filtered through Celite, and concentrated *in vacuo*. Then the mixture was dissolved in dichloromethane (10 mL), washed with $NaHCO_{3(aq)}$ (20 mL) and brine (10 mL). The combined organic layers were dried over $MgSO_4$, concentrated *in vacuo*, and purified by flash chromatography (3–5% methanol in dichloromethane with 0.1% NH_4OH) to yield the title compound **64s** (56 mg, 0.13 mmol, 60%) as yellow solid. LRMS (ESI) *m*/*z*: 447.3 $[M+H]^+$.

(1*R*,2*S*)-2-{[5-(3-Amino-4-{[2-(dimethylamino)ethyl](methyl)amino}phenyl)-6-phenylfuro[2,3-*d*]pyrimidin-4-yl]amino}cyclopentanol (64t).

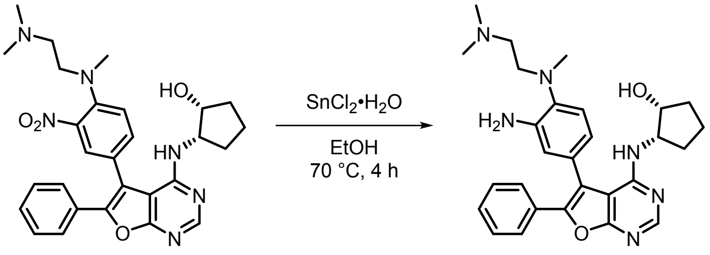

To a solution of **63t** (95 mg, 0.18 mmol, 1.0 equiv.) in ethanol (4.8 mL) was added $SnCl_2 \cdot 2H_2O$ (83 mg, 0.37 mmol, 2.0 equiv.) then the reaction mixture was stirred at 70 °C. After stirred for 4 hours, the reaction mixture was cooled down to room temperature, filtered through Celite, and concentrated *in vacuo*. Then the mixture was dissolved in ethyl acetate (10 mL), washed with $NaHCO_{3(aq)}$ (20 mL) and brine (10 mL). The combined organic layers were dried over $MgSO_4$, concentrated *in vacuo*, and purified by flash chromatography (5% methanol in dichloromethane) to yield the title compound **64t** (81 mg, 0.17 mmol, 91%) as yellow oil. LRMS (ESI) *m*/*z*: 487.3 $[M+H]^+$.

(1*R*,2*R*)-2-{[5-(3-Amino-4-{[2-(dimethylamino)ethyl](methyl)amino}phenyl)-6-phenylfuro[2,3-*d*]pyrimidin-4-yl]amino}cyclopentanol (64u).

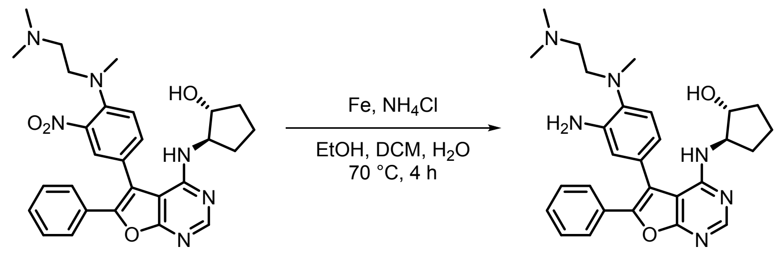

To a solution of **63u** (140 mg, 0.27 mmol, 1.0 equiv.) in ethanol (3 mL), dichloromethane (3 mL) and water (0.6 mL) was added iron powder (46 mg, 0.82 mmol, 3.0 equiv.) and sat. $NH_4Cl_{(aq)}$ (0.3 mL) then the reaction mixture was stirred at 80 °C. After stirred for 12 hours, the reaction mixture was cooled down to room temperature, filtered through Celite, concentrated *in vacuo*, and purified by flash chromatography (5–7% methanol in dichloromethane) to yield the title compound **64u** (85 mg, 0.17 mmol, 64%) as brown solid. LRMS (ESI) *m*/*z*: 487.5 $[M+H]^+$.

3-{[5-(3-Amino-4-{[2-(dimethylamino)ethyl](methyl)amino}phenyl)-6-phenylfuro[2,3-*d*]pyrimidin-4-yl]amino}cyclobutanol (64v).

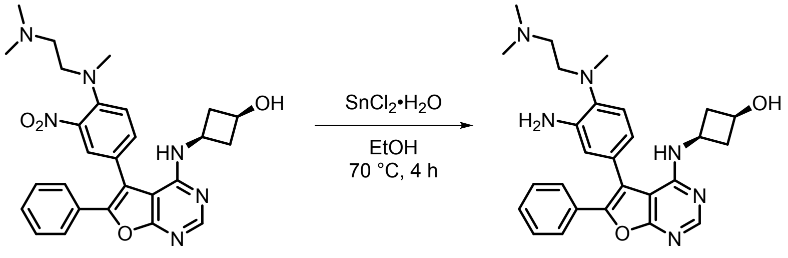

To a solution of **63v** (88 mg, 0.18 mmol, 1.0 equiv.) in ethanol (4.4 mL) was added $SnCl_2 \cdot 2H_2O$ (79 mg, 0.35 mmol, 2.0 equiv.) then the reaction mixture was stirred at 70 °C. After stirred for 4 hours, the reaction mixture was cooled down to room temperature, filtered through Celite, and concentrated *in vacuo*. Then the mixture was dissolved in ethyl acetate (10 mL), washed with $NaHCO_{3(aq)}$ (20 mL) and brine (10 mL). The combined organic layers were dried over $MgSO_4$, concentrated *in vacuo*, and purified by flash chromatography (5% methanol in dichloromethane) to yield the title compound **64v** (78 mg, 0.17 mmol, 94%) as yellow oil. LRMS (ESI) *m*/*z*: 473.3 $[M+H]^+$.

3-{[5-(3-Amino-4-{[2-(dimethylamino)ethyl](methyl)amino}phenyl)-6-phenylfuro[2,3-*d*]pyrimidin-4-yl]amino}cyclobutanol (64w).

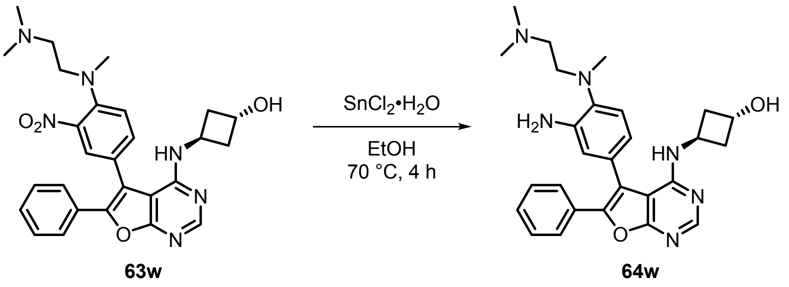

To a solution of **63w** (81 mg, 0.16 mmol, 1.0 equiv.) in ethanol (4.1 mL) was added $SnCl_2 \cdot 2H_2O$ (73 mg, 0.32 mmol, 2.0 equiv.) then the reaction mixture was stirred at 70 °C. After stirred for 4 hours, the reaction mixture was cooled down to room temperature, filtered through Celite, and concentrated *in vacuo*. Then the mixture was dissolved in ethyl acetate (10 mL), washed with $NaHCO_{3(aq)}$ (20 mL) and brine (10 mL). The combined organic layers were dried over $MgSO_4$, concentrated *in vacuo*, and purified by flash chromatography (5% methanol in dichloromethane) to yield the title compound **64w** (69 mg, 0.15 mmol, 91%) as yellow solid. LRMS (ESI) *m*/*z*: 473.3 $[M+H]^+$.

***N*-(2-{[*tert*-Butyl(dimethyl)silyl]oxy}ethyl)-5-(3-nitrophenyl)furo[2,3-*d*]pyrimidin-4-amine (66a).**

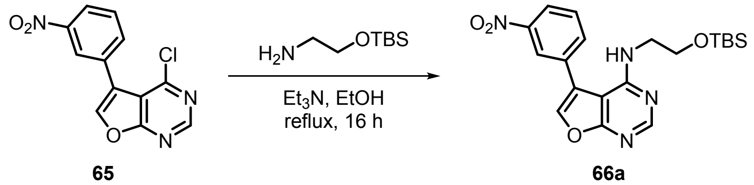

To a solution of 4-chloro-5-(3-nitrophenyl)furo[2,3-*d*]pyrimidine (**65**)[2] (120 mg, 0.44 mmol, 1.0 equiv.) in ethanol (2.0 mL) was added 2-[(*tert*-butyldimethylsilyl)oxy]ethan-1-amine (88 mg, 0.50 mmol, 1.2 equiv.) and triethylamine (58 mg, 0.50 mmol, 1.2 equiv.) then the reaction mixture was stirred at reflux. After stirred for 16 hours, the reaction mixture was cooled down to room temperature, concentrated *in vacuo*, added H_2O (10 mL) and extracted into dichloromethane (10 mL × 3). The combined organic layers were washed with brine (10 mL), dried over $MgSO_4$, concentrated *in vacuo*. The crude title compound **66a** was yielded (115 mg, 0.28 mmol, 64%) as yellow solid without further purification. LRMS (ESI) *m*/*z*: 415.2 $[M+H]^+$.

6-Bromo-*N*-(2-{[*tert*-butyl(dimethyl)silyl]oxy}ethyl)-5-(3-nitrophenyl)furo[2,3-*d*]pyrimidin-4-amine (66b).

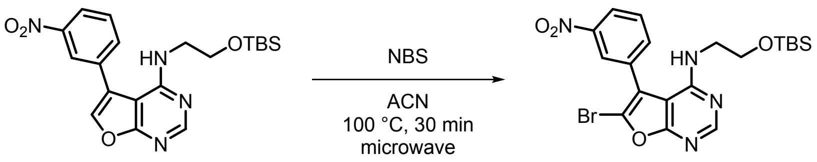

66a **66b**

To a solution of **66a** (3.00 g, 7.24 mmol, 1.0 equiv.) in acetonitrile (12.0 mL) was added *N*-bromosuccinimide (1.35 g, 7.58 mmol, 1.1 equiv.) then the reaction mixture was stirred at 100 °C under microwave irradiation. After stirred for 30 minutes, the reaction mixture was cooled down to room temperature, concentrated *in vacuo*, added H_2O (10 mL) and extracted into dichloromethane (3 × 10 mL). The combined organic layers were washed with brine (10 mL), dried over $MgSO_4$, concentrated *in vacuo*. The crude title compound **66b** was yielded (3.54 g, 7.17 mmol, 99%) as yellow solid without further purification. LRMS (ESI) *m*/*z*: 493.1 $[M+H]^+$.

***N*-(2-{[*tert*-Butyl(dimethyl)silyl]oxy}ethyl)-6-[4-(dimethylamino)phenyl]-5-(3-nitrophenyl)furo[2,3-*d*]pyrimidin-4-amine (66c).**

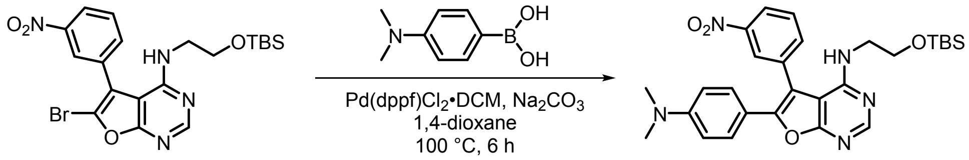

66b **66c**

To a solution of **66b** (200 mg, 0.41 mmol, 1.0 equiv.) in 1,4-dioxane (2.5 mL) was added 4-(*N*,*N*-dimethylamino)phenylboronic acid (99 mg, 0.60 mmol, 1.5 equiv.), $Pd(dppf)Cl_2 \cdot CH_2Cl_2$ (33 mg, 0.04 mmol, 10 mol%) and 2.0 M $Na_2CO_{3(aq)}$ (0.3 mL, 1.5 equiv.). The reaction mixture was degassed for 30 minutes, refilled with argon and stirred at 100 °C. After stirred for 6 hours, the reaction mixture was cooled down to room temperature, filtered through Celite, concentrated *in vacuo*, and purified by flash column chromatography (2% methanol in dichloromethane) to yield the title compound **66c** (130 mg, 0.24 mmol, 60%) as orange solid. LRMS (ESI) *m*/*z*: 534.1 $[M+H]^+$.

***N*-(2-{[*tert*-Butyl(dimethyl)silyl]oxy}ethyl)-6-[4-(morpholin-4-yl)phenyl]-5-(3-nitrophenyl)furo[2,3-*d*]pyrimidin-4-amine (66d).**

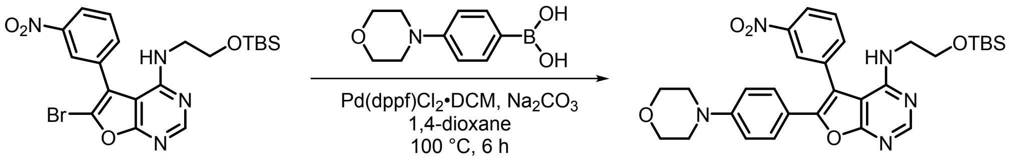

66b **66d**

To a solution of **66b** (200 mg, 0.41 mmol, 1.0 equiv.) in 1,4-dioxane (2.5 mL) was added 4-(morpholino)phenylboronic acid (124 mg, 0.60 mmol, 1.5 equiv.), $Pd(dppf)Cl_2 \cdot CH_2Cl_2$ (33 mg, 0.04 mmol, 10 mol%) and 2.0 M $Na_2CO_{3(aq)}$ (0.3 mL,

1.5 equiv.). The reaction mixture was degassed for 30 minutes, refilled with argon and stirred at 100 °C. After stirred for 6 hours, the reaction mixture was cooled down to room temperature, filtered through Celite, concentrated *in vacuo*, and purified by flash column chromatography (1% methanol in dichloromethane) to yield the title compound **66d** (187 mg, 0.32 mmol, 80%) as orange solid. LRMS (ESI) *m*/*z*: 576.2 $[M+H]^+$.

_N_-(2-{[_tert_-Butyl(dimethyl)silyl]oxy}ethyl)-6-[4-(4-methylpiperazin-1-yl)phenyl]-5-(3-nitrophenyl)furo[2,3-_d_]pyrimidin-4-amine (66e).

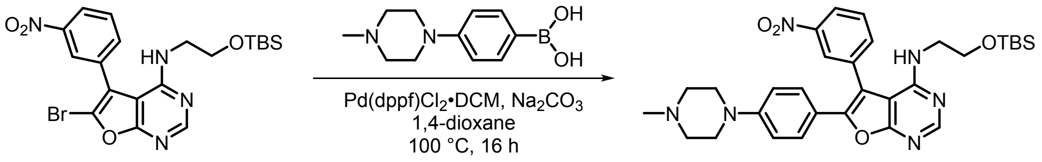

66b **66e**

To a solution of **66b** (247 mg, 0.50 mmol, 1.0 equiv.) in 1,4-dioxane (3.0 mL) was added 4-(4-methylpiperazin-1-yl)phenylboronic acid (302 mg, 1.00 mmol, 2.0 equiv.), $Pd(dppf)Cl_2 \cdot CH_2Cl_2$ (41 mg, 0.05 mmol, 10 mol%) and 2.0 M $Na_2CO_{3(aq)}$ (0.5 mL, 2.0 equiv.). The reaction mixture was degassed for 30 minutes, refilled with argon and stirred at 100 °C. After stirred for 16 hours, the reaction mixture was cooled down to room temperature, filtered through Celite, concentrated *in vacuo*, and purified by flash column chromatography (63% ethyl acetate in hexanes) to yield the title compound **66e** (200 mg, 0.34 mmol, 68%) as yellow solid. LRMS (ESI) *m*/*z*: 589.2 $[M+H]^+$.

2-{[5-(3-Aminophenyl)furo[2,3-_d_]pyrimidin-4-yl]amino}ethanol (67a).

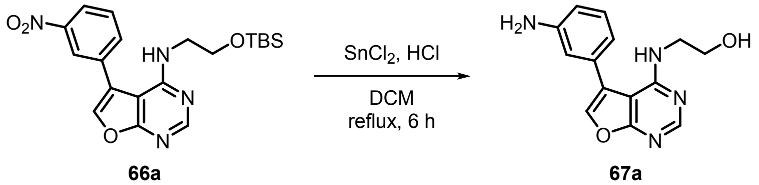

66a **67a**

To a solution of **66a** (40 mg, 0.10 mmol, 1.0 equiv.) in dichloromethane (2.0 mL) was added tin(II) chloride (70 mg, 0.37 mmol, 3.8 equiv.) and 12N HCl (catalytic amount) then the reaction mixture was stirred at reflux. After stirred for 6 hours, the reaction mixture was cooled down to room temperature, concentrated *in vacuo*, quenched with $NH_4OH_{(aq)}$ (10 mL) and purified by flash column chromatography (5% methanol in dichloromethane) to yield title compound **67a** (25 mg, 0.09 mmol, 96%) as white solid. LRMS (ESI) *m*/*z*: 271.1 $[M+H]^+$.

5-(3-Aminophenyl)-_N_-(2-{[_tert_-butyl(dimethyl)silyl]oxy}ethyl)-6-[4-(dimethylamino)phenyl]furo[2,3-_d_]pyrimidin-4-amine (67c).

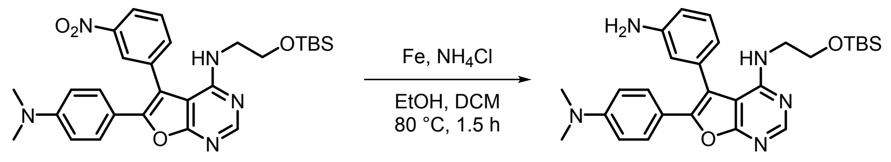

66c **67c**

To a solution of **66c** (130 mg, 0.24 mmol, 1.0 equiv.) in ethanol (2.0 mL) and dichloromethane (2.0 mL) was added saturated ammonium chloride(aq) (0.2 mL) and iron (28 mg, 0.50 mmol, 2.1 equiv.) then the reaction was stirred at 80 °C. After stirred for 1.5 hours, the reaction mixture was cooled down to room temperature, filtered through Celite, concentrated *in vacuo* and purified by flash column chromatography (2% methanol in dichloromethane) to yield the title compound **67c** (115 mg, 0.23 mmol, 94%) as yellow solid. LRMS (ESI) *m*/*z*: 504.1 $[M+H]^+$.

5-(3-Aminophenyl)-*N*-(2-{[*tert*-butyl(dimethyl)silyl]oxy}ethyl)-6-[4-(morpholin-4-yl)phenyl]furo[2,3-*d*]pyrimidin-4-amine (67d).

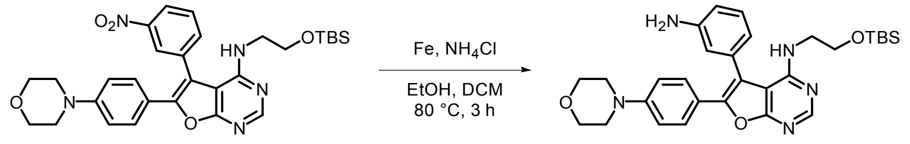

66d **67d**

To a solution of **66d** (187 mg, 0.32 mmol, 1.0 equiv.) in ethanol (4.0 mL) and dichloromethane (4.0 mL) was added saturated ammonium chloride$_{(aq)}$ (0.4 mL) and iron (56 mg, 1.00 mmol, 3.1 equiv.) then the reaction was stirred at 80 °C. After stirred for 3 hours, the reaction mixture was cooled down to room temperature, filtered through Celite, concentrated *in vacuo* and purified by flash column chromatography (0.5% methanol in dichloromethane) to yield the title compound **67d** (94 mg, 0.17 mmol, 53%) as yellow solid. LRMS (ESI) *m*/*z*: 546.1 $[M+H]^+$.

5-(3-Aminophenyl)-*N*-(2-{[*tert*-butyl(dimethyl)silyl]oxy}ethyl)-6-[4-(4-methylpiperazin-1-yl)phenyl]furo[2,3-*d*]pyrimidin-4-amine (67e).

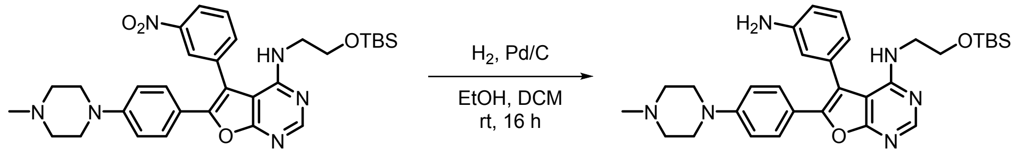

66e **67e**

To a solution of **66e** (200 mg, 0.34 mmol, 1.0 equiv.) in ethanol (4.0 mL) and dichloromethane (4.0 mL) was added 10% palladium on carbon (20 mg, 0.02 mmol, 6 mol%) then the reaction was stirred under an atmosphere of hydrogen at room temperature. After stirred for 16 hours, the reaction mixture was filtered through Celite, concentrated *in vacuo* and purified by flash column chromatography (63%

ethyl acetate in hexanes) to yield the title compound **67e** (114 mg, 0.20 mmol, 60%) as brown solid. LRMS (ESI) *m*/*z*: 554.1 $[M+H]^+$.

4-Chloro-5-(3-nitrophenyl)-6-phenylfuro[2,3-*d*]pyrimidine (69a).

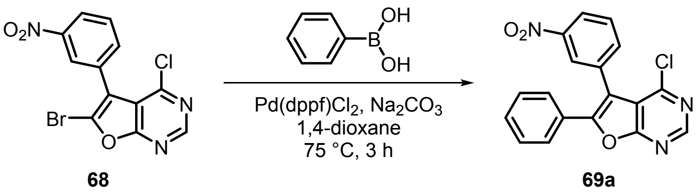

To a solution of 6-bromo-4-chloro-5-(3-nitrophenyl)furo[2,3-*d*]pyrimidine (**68**)[2] (100 mg, 0.28 mmol, 1.0 equiv.) in 1,4-dioxane (3.0 mL) was added phenylboronic acid (36 mg, 0.30 mmol, 1.0 equiv.), Pd(dppf)Cl_2 (36 mg, 0.05 mmol, 17 mol%) and 2.0 M $Na_2CO_{3(aq)}$ (212 μL, 0.42 mmol, 1.5 equiv.). The reaction mixture was degassed for 30 minutes, refilled with argon and stirred at 75 °C. After stirred for 3 hours, the reaction mixture was cooled down to room temperature, filtered through Celite, concentrated *in vacuo*, and purified by flash chromatography (30–80% dichloromethane in hexane) to yield the title compound **69a** (53 mg, 0.15 mmol, 53%) as white solid. LRMS (ESI) *m*/*z*: 352.1 $[M+H]^+$.

4-Chloro-6-[4-(4-methylpiperazin-1-yl)phenyl]-5-(3-nitrophenyl)furo[2,3-*d*]pyrimidine (69b).

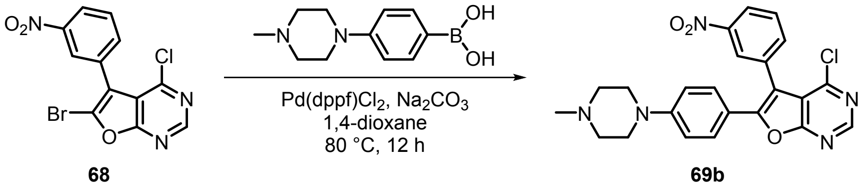

To a solution of 6-bromo-4-chloro-5-(3-nitrophenyl)furo[2,3-*d*]pyrimidine (**68**)[2] (300 mg, 0.53 mmol, 1.0 equiv.) in 1,4-dioxane (3.0 mL) was added 4-(4-methylpiperazin-1-yl)phenylboronic acid (384 mg, 1.27 mmol, 1.5 equiv.), Pd(dppf)Cl_2 (124 mg, 0.16 mmol, 20 mol%) and 2.0 M $Na_2CO_{3(aq)}$ (1.05 mL, 2.11 mmol, 2.5 equiv.). The reaction mixture was degassed for 30 minutes, refilled with argon and stirred at 80 °C. After stirred for 12 hours, the reaction mixture was cooled down to room temperature, filtered through Celite, concentrated *in vacuo*, and purified by flash chromatography (5–7% methanol in dichloromethane) to yield the title compound **69b** (210 mg, 0.47 mmol, 55%) as brown solid. LRMS (ESI) *m*/*z*: 450.0 $[M+H]^+$.

6-Bromo-*N*-cyclohexyl-5-(3-nitrophenyl)furo[2,3-*d*]pyrimidin-4-amine (70a).

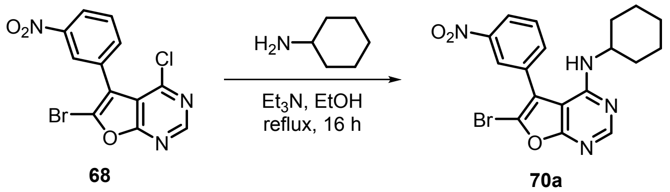

68 **70a**

To a solution of 6-bromo-4-chloro-5-(3-nitrophenyl)furo[2,3-*d*]pyrimidine (**68**) (300 mg, 0.85 mmol, 1.0 equiv.) in ethanol (2.8 mL) was added cyclohexylamine (130 μL, 1.14 mmol, 1.3 equiv.) and triethylamine (180 μL, 1.29 mmol, 1.5 equiv.) then the reaction mixture was stirred at reflux. After stirred for 16 hours, the reaction mixture was cooled down to room temperature, concentrated *in vacuo*, and purified by flash chromatography (2% methanol in dichloromethane) to yield the title compound **70a** (313 mg, 0.75 mmol, 89%) as yellow solid. LRMS (ESI) *m*/*z*: 417.0 $[M+H]^+$.

2-{[6-Bromo-5-(3-nitrophenyl)furo[2,3-*d*]pyrimidin-4-yl]amino}ethanol (70b).

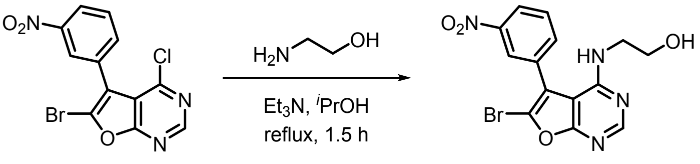

68 **70b**

To a solution of 6-bromo-4-chloro-5-(3-nitrophenyl)furo[2,3-*d*]pyrimidine (**68**) (300 mg, 0.85 mmol, 1.0 equiv.) in isopropyl alcohol (1.5 mL) was added ethanolamine (56 μL, 0.93 mmol, 1.1 equiv.) and triethylamine (180 μL, 1.30 mmol, 1.5 equiv.) then the reaction mixture was stirred at reflux. After stirred for 1.5 hours, the reaction mixture was cooled down to room temperature. The resulting precipitate was collected, washed with ethyl acetate (10 mL) to yield the title compound **70b** (271 mg, 0.71 mmol, 84%) as yellow solid. The crude product was used for next step without further purification. LRMS (ESI) *m*/*z*: 379.2 $[M+H]^+$.

6-Bromo-*N*-(2-{[*tert*-butyl(dimethyl)silyl]oxy}ethyl)-5-(3-nitrophenyl)furo[2,3-*d*]pyrimidin-4-amine (70b'). To a solution of **70b** (271 mg, 0.71 mmol, 1.0 equiv.) in *N*,*N*-dimethylformamide (5.3 mL) and dichloromethane (120 μL) was added triethylamine (410 μL, 2.96 mmol, 4.1 equiv.) and *tert*-butyldimethylsilyl chloride (216 mg, 1.43 mmol, 2.0 equiv.) then the reaction mixture was stirred at room temperature. After stirred for 16 hours, the reaction mixture was concentrated *in vacuo* and purified by CombiFlash automated flash chromatography (5% methanol in dichloromethane) to yield the title compound **70b'** (279 mg, 0.57 mmol, 79%) as yellow solid. LRMS (ESI) *m*/*z*: 493.2 $[M+H]^+$.

6-Bromo-*N*-cyclopentyl-5-(3-nitrophenyl)furo[2,3-*d*]pyrimidin-4-amine (70c).

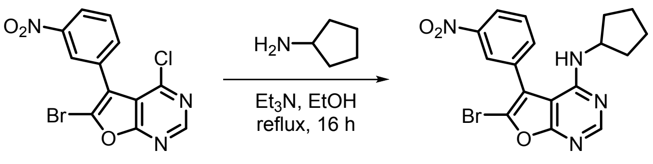

68 **70c**

To a solution of 6-bromo-4-chloro-5-(3-nitrophenyl)furo[2,3-*d*]pyrimidine (**68**) (500 mg, 1.41 mmol, 1.0 equiv.) in ethanol (2.0 mL) was added cyclopentylamine (181 μL, 1.83 mmol, 1.3 equiv.) and triethylamine (236 μL, 1.69 mmol, 1.2 equiv.) then the reaction mixture was stirred at reflux. After stirred for 16 hours, the reaction mixture was cooled down to room temperature. The resulting precipitate was collected and washed with cold ethanol (10 mL) to yield the title compound **70c** (480 mg, 1.19 mmol, 84%) as light brown solid. LRMS (ESI) *m*/*z*: 403.1 $[M+H]^+$.

(2*S*)-2-{[6-Bromo-5-(3-nitrophenyl)furo[2,3-*d*]pyrimidin-4-yl]amino}-2-phenylethanol (70d).

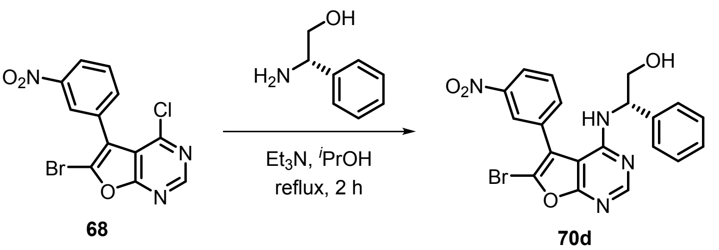

68 **70d**

To a solution of 6-bromo-4-chloro-5-(3-nitrophenyl)furo[2,3-*d*]pyrimidine (**68**) (300 mg, 0.85 mmol, 1.0 equiv.) in isopropyl alcohol (1.0 mL) was added (*S*)-(+)-2-phenylglycinol (128 mg, 0.93 mmol, 1.1 equiv.) and triethylamine (180 μL, 1.29 mmol, 1.5 equiv.) then the reaction mixture was stirred at reflux. After stirred for 2 hours, the reaction mixture was cooled down to room temperature, and the resulting precipitate was collected and washed with cold water (10 mL) to yield the title compound **70d** (366 mg, 0.80 mmol, 95%) as yellow solid. LRMS (ESI) *m*/*z*: 455.3 $[M+H]^+$.

***N*-Methyl-5-(3-nitrophenyl)-6-phenylfuro[2,3-*d*]pyrimidin-4-amine (71a).**

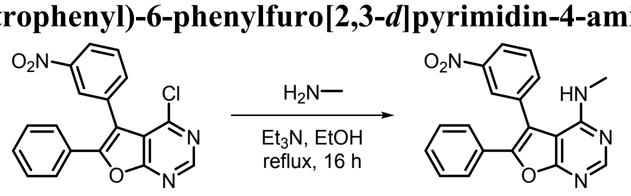

69a **71a**

To a solution of **69a** (50 mg, 0.14 mmol, 1.0 equiv.) in ethanol (2.0 mL) was added methylamine (92 μL, 0.19 mmol, 1.3 equiv., 2M solution in THF) and triethylamine (24 μL, 0.17 mmol, 1.2 equiv.) then the reaction mixture was stirred at reflux. After stirred for 16 hours, the reaction mixture was cooled down to room temperature and

concentrated *in vacuo*. The resulting precipitate was washed with cold ethanol (10 mL) and collected to yield the title compound **71a** (47 mg, 0.14 mmol, 95%) as yellow solid. LRMS (ESI) *m*/*z*: 347.1 [M+H]$^+$.

_N_,_N_-Dimethyl-5-(3-nitrophenyl)-6-phenylfuro[2,3-_d_]pyrimidin-4-amine (71b).

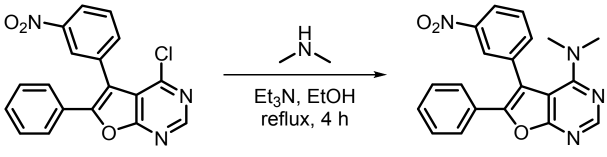

69a **71b**

To a solution of **69a** (50 mg, 0.14 mmol, 1.0 equiv.) in ethanol (2.0 mL) was added dimethylamine (92 μL, 0.19 mmol, 1.3 equiv., 2M solution in THF) and triethylamine (24 μL, 0.17 mmol, 1.2 equiv.) then the reaction mixture was stirred at reflux. After stirred for 4 hours, the reaction mixture was cooled down to room temperature and concentrated *in vacuo*. The resulting precipitate was washed with cold ethanol (10 mL) and collected to yield the title compound **71b** (46 mg, 0.13 mmol, 90%) as yellow solid. LRMS (ESI) *m*/*z*: 361.1 [M+H]$^+$.

5-(3-Nitrophenyl)-6-phenyl-_N_-(propan-2-yl)furo[2,3-_d_]pyrimidin-4-amine (71c).

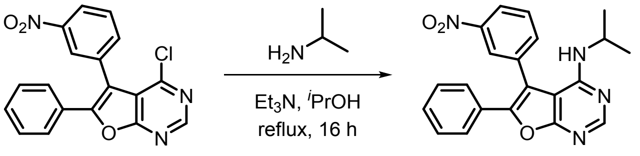

69a **71c**

To a solution of **69a** (50 mg, 0.14 mmol, 1.0 equiv.) in ethanol (2.0 mL) was added isopropylamine (16 μL, 0.19 mmol, 1.3 equiv.) and triethylamine (24 μL, 0.17 mmol, 1.2 equiv.) then the reaction mixture was stirred at reflux. After stirred for 16 hours, the reaction mixture was cooled down to room temperature and concentrated *in vacuo*. The resulting precipitate was washed with cold ethanol (10 mL) and collected to yield the title compound **71c** (32 mg, 0.09 mmol, 60%) as yellow solid. LRMS (ESI) *m*/*z*: 375.1 [M+H]$^+$.

_N_-Cyclopropyl-5-(3-nitrophenyl)-6-phenylfuro[2,3-_d_]pyrimidin-4-amine (71d).

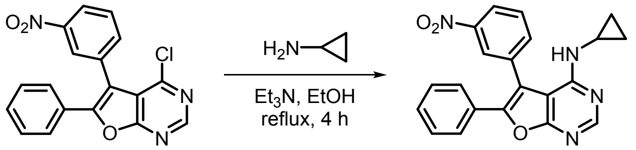

69a **71d**

To a solution of **69a** (50 mg, 0.14 mmol, 1.0 equiv.) in ethanol (2.0 mL) was added cyclopropylamine (13 μL, 0.19 mmol, 1.3 equiv.) and triethylamine (24 μL, 0.17 mmol, 1.2 equiv.) then the reaction mixture was stirred at reflux. After stirred for 4

hours, the reaction mixture was cooled down to room temperature and concentrated *in vacuo*. The resulting precipitate was washed with cold ethanol (10 mL) and collected to yield the title compound **71d** (42 mg, 0.11 mmol, 79%) as yellow solid. LRMS (ESI) *m*/*z*: 373.1 $[M+H]^+$.

N-Cyclobutyl-5-(3-nitrophenyl)-6-phenylfuro[2,3-*d*]pyrimidin-4-amine (71e).

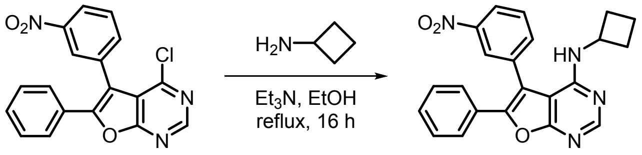

69a **71e**

To a solution of **69a** (50 mg, 0.14 mmol, 1.0 equiv.) in ethanol (2.0 mL) was added cyclobutylamine (16 μL, 0.19 mmol, 1.3 equiv.) and triethylamine (24 μL, 0.17 mmol, 1.2 equiv.) then the reaction mixture was stirred at reflux. After stirred for 16 hours, the reaction mixture was cooled down to room temperature and concentrated *in vacuo*. The resulting precipitate was washed with cold ethanol (10 mL) and collected to yield the title compound **71e** (44 mg, 0.11 mmol, 80%) as yellow solid. LRMS (ESI) *m*/*z*: 387.1 $[M+H]^+$.

N-Cyclopentyl-5-(3-nitrophenyl)-6-phenylfuro[2,3-*d*]pyrimidin-4-amine (71f).

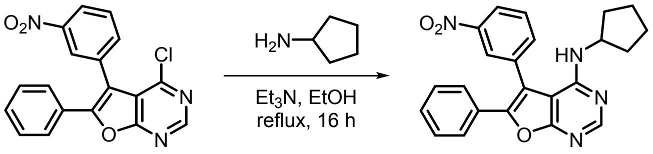

69a **71f**

To a solution of **69a** (50 mg, 0.14 mmol, 1.0 equiv.) in ethanol (2.0 mL) was added cyclopentylamine (18 μL, 0.18 mmol, 1.3 equiv.) and triethylamine (24 μL, 0.17 mmol, 1.2 equiv.) then the reaction mixture was stirred at reflux. After stirred for 16 hours, the reaction mixture was cooled down to room temperature and concentrated *in vacuo*. The resulting precipitate was washed with cold ethanol (10 mL) and collected to yield the title compound **71f** (43 mg, 0.11 mmol, 76%) as yellow solid. LRMS (ESI) *m*/*z*: 401.1 $[M+H]^+$.

N-Cyclohexyl-5-(3-nitrophenyl)-6-phenylfuro[2,3-*d*]pyrimidin-4-amine (71g).

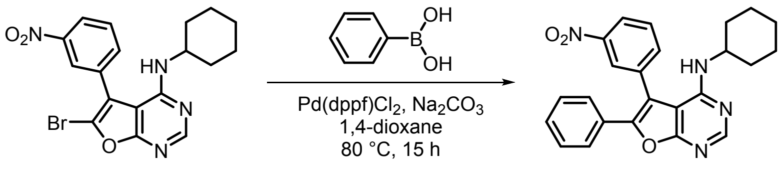

70a **71g**

To a solution of **70a** (80 mg, 0.19 mmol, 1.0 equiv.) in 1,4-dioxane (1.2 mL) was added phenylboronic acid (35 mg, 0.29 mmol, 1.5 equiv.), Pd(dppf)Cl_2 (14 mg, 0.02 mmol, 10 mol%) and 2.0 M $Na_2CO_{3(aq)}$ (190 μL, 0.42 mmol, 2.2 equiv.). The reaction mixture was degassed for 30 minutes, refilled with argon and stirred at 80 °C. After stirred for 15 hours, the reaction mixture was cooled down to room temperature, filtered through Celite, concentrated *in vacuo*, and purified by flash chromatography (25% ethyl acetate in hexane) to yield the title compound **71g** (76 mg, 0.18 mmol, 96%) as yellow solid. LRMS (ESI) *m/z*: 415.1 $[M+H]^+$.

***N*¹-(4-{4-[(2-{[*tert*-Butyl(dimethyl)silyl]oxy}ethyl)amino]-5-(3-nitrophenyl)furo[2,3-*d*]pyrimidin-6-yl}phenyl)-*N*¹,*N*²,*N*²-trimethylethane-1,2-diamine (71h).**

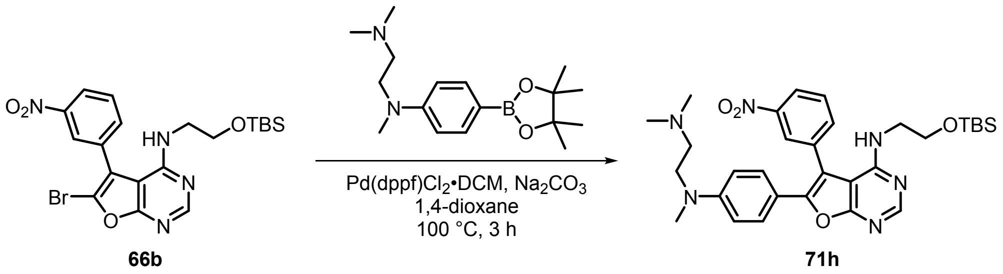

To a solution of **66b** (180 mg, 0.36 mmol, 1.0 equiv.) in 1,4-dioxane (3.6 mL) was added *N*,*N*,*N*'-trimethyl-*N*'-[4-(4,4,5,5-tetramethyl-1,3,2-dioxaborolan-2-yl)phenyl]ethane-1,2-diamine (133 mg, 0.44 mmol, 1.2 equiv.), Pd(dppf)$Cl_2 \cdot CH_2Cl_2$ (30 mg, 0.04 mmol, 10 mol%) and 2.0 M $Na_2CO_{3(aq)}$ (550 μL, 1.1 mmol, 3.0 equiv.). The reaction mixture was degassed for 30 minutes, refilled with argon and stirred at 100 °C. After stirred for 3 hours, the reaction mixture was cooled down to room temperature, filtered through Celite, concentrated *in vacuo*, and purified by CombiFlash automated flash chromatography (5% methanol in dichloromethane with 0.1% NH_4OH) to yield the title compound **71h** (161 mg, 0.27 mmol, 75%) as brown oil. LRMS (ESI) *m/z*: 591.2 $[M+H]^+$.

***N*-Cyclopentyl-6-[4-(morpholin-4-yl)phenyl]-5-(3-nitrophenyl)furo[2,3-*d*]pyrimidin-4-amine (71i).**

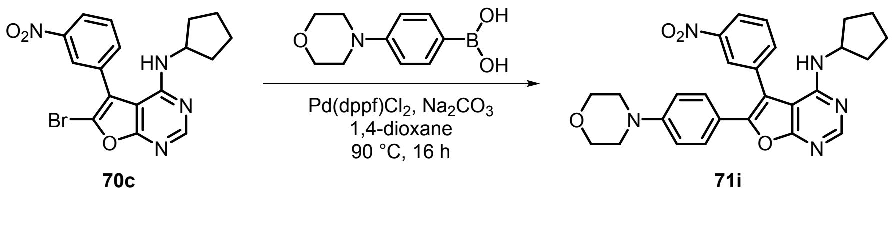

To a solution of **70c** (80 mg, 0.20 mmol, 1.0 equiv.) in 1,4-dioxane (5.5 mL) was added 4-(morpholino)phenylboronic acid (62 mg, 0.21 mmol, 1.1 equiv.), Pd(dppf)Cl_2

(29 mg, 0.04 mmol, 20 mol%) and 2.0 M $Na_2CO_{3(aq)}$ (149 µL, 0.30 mmol, 1.5 equiv.). The reaction mixture was degassed for 30 minutes, refilled with argon and stirred at 90 °C. After stirred for 16 hours, the reaction mixture was cooled down to room temperature, filtered through Celite, concentrated *in vacuo*, and purified by flash chromatography (17–50% ethyl acetate in hexane) to yield the title compound **71i** (90 mg, 0.19 mmol, 93%) as yellow solid. LRMS (ESI) *m/z*: 486.1 $[M+H]^+$.

_N_-Cyclopentyl-6-[4-(morpholin-4-yl)phenyl]-5-(3-nitrophenyl)furo[2,3-_d_]pyrimidin-4-amine (71j).

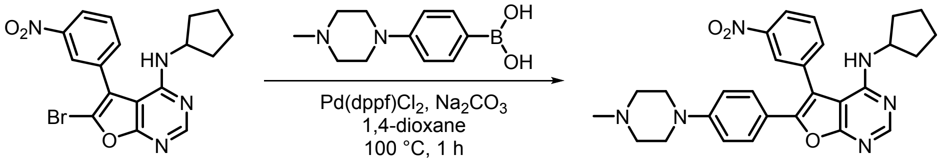

To a solution of **70c** (158 mg, 0.39 mmol, 1.0 equiv.) in 1,4-dioxane (1.0 mL) was added 4-(4-methylpiperazin-1-yl)phenylboronic acid (129 mg, 0.59 mmol, 1.5 equiv.), $Pd(dppf)Cl_2$ (29 mg, 0.04 mmol, 10 mol%) and 2.0 M Na_2CO_3(aq) (0.52 mL, 1.18 mmol, 3.0 equiv.). The reaction mixture was degassed for 30 minutes, refilled with argon and stirred at 100 °C. After stirred for 1 hour, the reaction mixture was cooled down to room temperature, filtered through Celite, concentrated *in vacuo*, and purified by flash chromatography (2% methanol in dichloromethane with 0.1% NH_4OH) to yield the title compound **71j** (180 mg, 0.36 mmol, 92%) as yellow solid. LRMS (ESI) *m/z*: 499.2 $[M+H]^+$.

(2_S_)-2-({6-[4-(4-Methylpiperazin-1-yl)phenyl]-5-(3-nitrophenyl)furo[2,3-_d_]pyrimidin-4-yl}amino)-2-phenylethanol (71k).

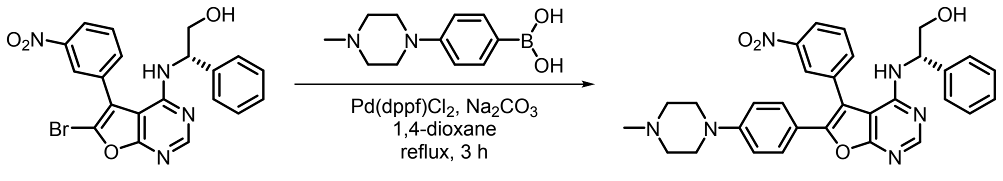

To a solution of **70d** (366 mg, 0.80 mmol, 1.0 equiv.) in 1,4-dioxane (5.6 mL) and H_2O (0.56 mL) was added (4-(4-methylpiperazin-1-yl)phenyl)boronic acid (363 mg, 1.65 mmol, 2.1 equiv.), $Pd(dppf)Cl_2$ (59 mg, 0.08 mmol, 10 mol%) and 2.0 M $Na_2CO_{3(aq)}$ (127 mg, 1.20 mmol, 1.5 equiv.). The reaction mixture was degassed for 30 minutes, refilled with argon and stirred at reflux. After stirred for 3 hours, the reaction mixture was cooled down to room temperature, filtered through Celite, concentrated *in vacuo*, and purified by Combiflash automated flash chromatography

(5–10% methanol in dichloromethane with 0.1% NH_4OH) to yield the title compound **71k** (333 mg, 0.60 mmol, 75%) as yellow solid. LRMS (ESI) *m/z*: 551.2 $[M+H]^+$.

***N*-Cyclohexyl-6-[4-(4-methylpiperazin-1-yl)phenyl]-5-(3-nitrophenyl)furo[2,3-*d*]pyrimidin-4-amine (71l).**

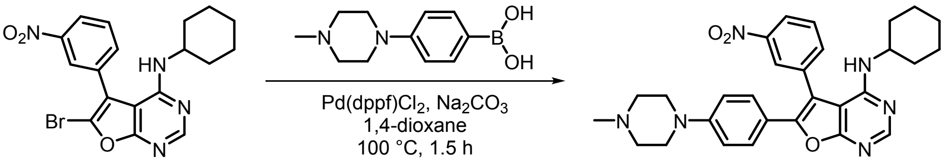

70a

71l

To a solution of **70a** (307 mg, 0.74 mmol, 1.0 equiv.) in 1,4-dioxane (3.0 mL) was added 4-(4-methylpiperazin-1-yl)phenylboronic acid (245 mg, 0.81 mmol, 1.1 equiv.), $Pd(dppf)Cl_2$ (54 mg, 0.07 mmol, 10 mol%) and 2.0 M $Na_2CO_{3(aq)}$ (1.10 mL, 2.21 mmol, 3.0 equiv.). The reaction mixture was degassed for 30 minutes, refilled with argon and stirred at 100 °C. After stirred for 1.5 hours, the reaction mixture was cooled down to room temperature, filtered through Celite, concentrated *in vacuo*, and purified by flash chromatography (5% methanol in dichloromethane with 0.1% NH_4OH) to yield the title compound **71l** (327 mg, 0.64 mmol, 87%) as yellow solid. LRMS (ESI) *m/z*: 513.2 $[M+H]^+$.

***N*-Methyl-6-[4-(4-methylpiperazin-1-yl)phenyl]-5-(3-nitrophenyl)furo[2,3-*d*]pyrimidin-4-amine (71m).**

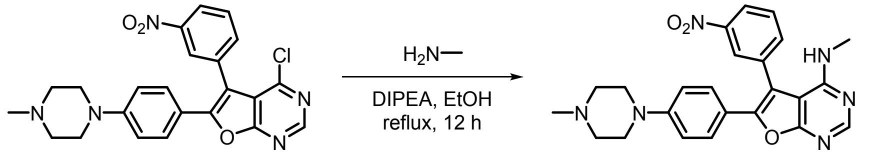

69b

71m

To a solution of **69b** (200 mg, 0.44 mmol, 1.0 equiv.) in ethanol (2.2 mL) was added methylamine (440 μL, 0.88 mmol, 2.0 equiv., 2M in methanol) and DIPEA (150 μL, 0.88 mmol, 2.0 equiv.) then the reaction mixture was stirred at reflux. After stirred for 12 hours, the reaction mixture was cooled down to room temperature, concentrated *in vacuo*, and purified by flash chromatography 30–40% ethyl acetate in hexanes to yield the title compound **71m** (100 mg, 0.22 mmol, 51%) as brown solid. LRMS (ESI) *m/z*: 445.2 $[M+H]^+$.

***N*-Cyclopentyl-5-(3-nitrophenyl)-6-(pyridin-4-yl)furo[2,3-*d*]pyrimidin-4-amine (71n).**

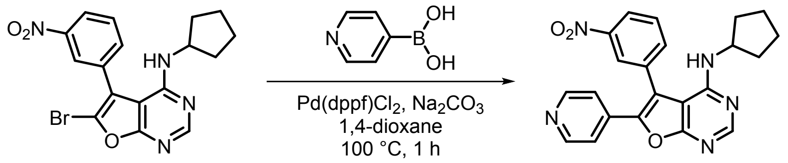

70c **71n**

To a solution of **70c** (100 mg, 0.25 mmol, 1.0 equiv.) in 1,4-dioxane (1.0 mL) was added 4-pyridylboronic acid (32 mg, 0.26 mmol, 1.0 equiv.), Pd(dppf)Cl_2 (18 mg, 0.02 mmol, 10 mol%) and 2.0 M $Na_2CO_{3(aq)}$ (0.37 mL, 0.75 mmol, 3.0 equiv.). The reaction mixture was degassed for 30 minutes, refilled with argon and stirred at 100 °C. After stirred for 1 hour, the reaction mixture was cooled down to room temperature, filtered through Celite, concentrated *in vacuo*, and purified by flash chromatography (5% methanol in dichloromethane with 0.1% NH_4OH) to yield the title compound **71n** (79 mg, 0.20 mmol, 79%) as yellow solid. LRMS (ESI) *m*/*z*: 402.1 $[M+H]^+$.

_N_-Cyclopentyl-6-[6-(morpholin-4-yl)pyridin-3-yl]-5-(3-nitrophenyl)furo[2,3-_d_]pyrimidin-4-amine (71o).

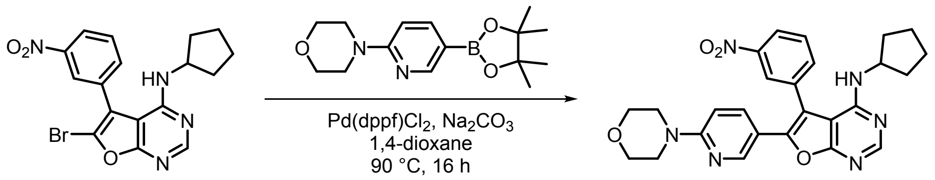

70c **71o**

To a solution of **70c** (80 mg, 0.20 mmol, 1.0 equiv.) in 1,4-dioxane (1.2 mL) was added 6-(morpholin-4-yl)pyridine-3-boronic acid pinacol ester (86 mg, 0.30 mmol, 1.5 equiv.), Pd(dppf)Cl_2 (15 mg, 0.02 mmol, 10 mol%) and 2.0 M $Na_2CO_{3(aq)}$ (0.20 mL, 0.44 mmol, 2.2 equiv.). The reaction mixture was degassed for 30 minutes, refilled with argon and stirred at 90 °C. After stirred for 16 hours, the reaction mixture was cooled down to room temperature, filtered through Celite, concentrated *in vacuo*, and purified by flash chromatography (33% ethyl acetate in hexane) to yield the title compound **71o** (71 mg, 0.15 mmol, 74%) as yellow solid. LRMS (ESI) *m*/*z*: 487.3 $[M+H]^+$.

_N_-Cyclopentyl-6-[6-(4-methylpiperazin-1-yl)pyridin-3-yl]-5-(3-nitrophenyl)furo[2,3-_d_]pyrimidin-4-amine (71p).

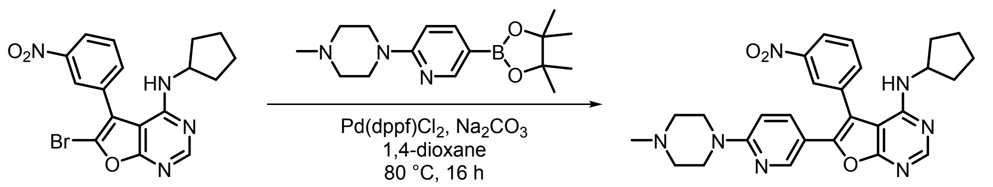

70c **71p**

To a solution of **70c** (74 mg, 0.18 mmol, 1.0 equiv.) in 1,4-dioxane (1.1 mL) was added 1-methyl-4-(5-(4,4,5,5-tetramethyl-1,3,2-dioxaborolan-2-yl)pyridin-2-yl)piperazine (83 mg, 0.24 mmol, 1.5 equiv.), Pd(dppf)Cl_2 (26 mg, 0.04 mmol, 20 mol%) and 2.0 M $Na_2CO_{3(aq)}$ (0.18 mL, 0.40 mmol, 2.2 equiv.). The reaction mixture was degassed for 30 minutes, refilled with argon and stirred at 80 °C. After stirred for 16 hours, the reaction mixture was cooled down to room temperature, filtered through Celite, concentrated *in vacuo*, and purified by flash column chromatography (5% methanol in dichloromethane) to yield the title compound **71p** (60 mg, 0.12 mmol, 65%) as yellow solid. LRMS (ESI) *m*/*z*: 500.3 $[M+H]^+$.

5-(3-Aminophenyl)-*N*-methyl-6-phenylfuro[2,3-*d*]pyrimidin-4-amine (72a).

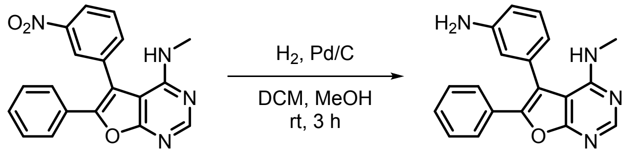

71a **72a**

To a solution of **71a** (47 mg, 0.14 mmol, 1.0 equiv.) in methanol (4.0 mL) and dichloromethane (1.0 mL) was added palladium on carbon (15 mg, 0.01 mmol, 10 mol%) then the reaction was stirred under an atmosphere of hydrogen at room temperature. After stirred for 3 hours, the reaction mixture was filtered through Celite, concentrated *in vacuo* to yield the title compound **72a** (40 mg, 0.13 mmol, 93%) as white solid. LRMS (ESI) *m*/*z*: 317.1 $[M+H]^+$.

5-(3-Aminophenyl)-*N*,*N*-dimethyl-6-phenylfuro[2,3-*d*]pyrimidin-4-amine (72b).

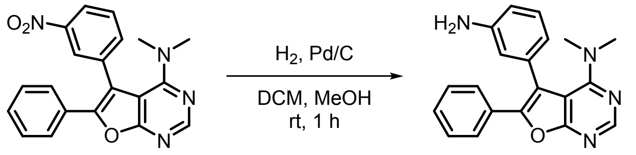

71b **72b**

To a solution of **71b** (45 mg, 0.12 mmol, 1.0 equiv.) in methanol (4.0 mL) and dichloromethane (1.0 mL) was added palladium on carbon (15 mg, 0.01 mmol, 10 mol%) then the reaction was stirred under an atmosphere of hydrogen at room temperature. After stirred for 1 hour, the reaction mixture was filtered through Celite, concentrated *in vacuo* to yield the title compound **72b** (40 mg, 0.12 mmol, 97%) as light yellow solid. LRMS (ESI) *m*/*z*: 331.1 $[M+H]^+$.

5-(3-Aminophenyl)-6-phenyl-*N*-(propan-2-yl)furo[2,3-*d*]pyrimidin-4-amine (72c).

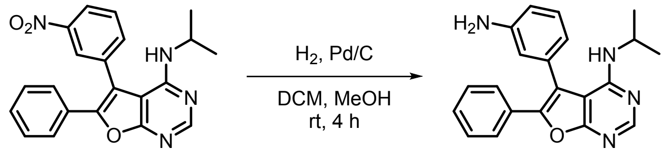

71c **72c**

To a solution of **71c** (32 mg, 0.09 mmol, 1.0 equiv.) in methanol (4.0 mL) and dichloromethane (1.0 mL) was added palladium on carbon (15 mg, 0.01 mmol, 10 mol%) then the reaction was stirred under an atmosphere of hydrogen at room temperature. After stirred for 4 hours, the reaction mixture was filtered through Celite, concentrated *in vacuo*, and purified by flash chromatography (25–50% ethyl acetate in hexane) to yield the title compound **72c** (27 mg, 0.08 mmol, 92%) as light yellow solid. LRMS (ESI) *m*/*z*: 345.1 $[M+H]^+$.

5-(3-Aminophenyl)-6-phenyl-*N*-(propan-2-yl)furo[2,3-*d*]pyrimidin-4-amine (72d).

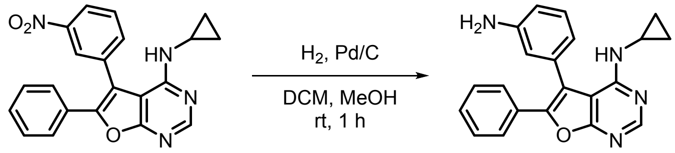

71d **72d**

To a solution of **71d** (42 mg, 0.11 mmol, 1.0 equiv.) in methanol (4.0 mL) and dichloromethane (1.0 mL) was added palladium on carbon (15 mg, 0.01 mmol, 10 mol%) then the reaction was stirred under an atmosphere of hydrogen at room temperature. After stirred for 1 hour, the reaction mixture was filtered through Celite, concentrated *in vacuo*, and purified by flash chromatography (33–75% ethyl acetate in hexane) to yield the title compound **72d** (32 mg, 0.09 mmol, 83%) as yellow solid. LRMS (ESI) *m*/*z*: 343.1 $[M+H]^+$.

5-(3-Aminophenyl)-6-phenyl-*N*-(propan-2-yl)furo[2,3-*d*]pyrimidin-4-amine (72e).

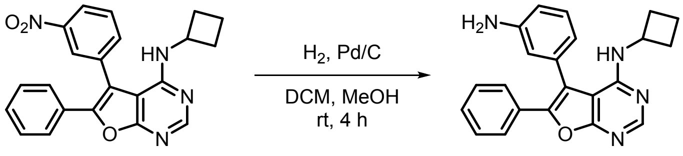

71e **72e**

To a solution of **71e** (44 mg, 0.11 mmol, 1.0 equiv.) in methanol (4.0 mL) and dichloromethane (1.0 mL) was added palladium on carbon (15 mg, 0.01 mmol, 10 mol%) then the reaction was stirred under an atmosphere of hydrogen at room temperature. After stirred for 4 hours, the reaction mixture was filtered through Celite

and concentrated *in vacuo* to yield the title compound **72e** (38 mg, 0.11 mmol, 94%) as white solid. LRMS (ESI) *m*/*z*: 357.1 $[M+H]^+$.

5-(3-Aminophenyl)-*N*-cyclopentyl-6-phenylfuro[2,3-*d*]pyrimidin-4-amine (72f).

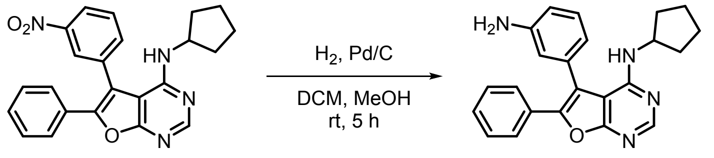

71f **72f**

To a solution of **71f** (42 mg, 0.10 mmol, 1.0 equiv.) in methanol (4.0 mL) and dichloromethane (1.0 mL) was added palladium on carbon (15 mg, 0.01 mmol, 10 mol%) then the reaction was stirred under an atmosphere of hydrogen at room temperature. After stirred for 5 hours, the reaction mixture was filtered through Celite and concentrated *in vacuo* to yield the title compound **72f** (37 mg, 0.10 mmol, 95%) as light yellow solid. LRMS (ESI) *m*/*z*: 371.1 $[M+H]^+$.

5-(3-Aminophenyl)-*N*-cyclohexyl-6-phenylfuro[2,3-*d*]pyrimidin-4-amine (72g).

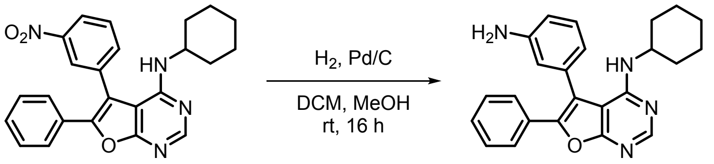

71g **72g**

To a solution of **71g** (74 mg, 0.18 mmol, 1.0 equiv.) in methanol (1.8 mL) and dichloromethane (1.8 mL) was added palladium on carbon (7 mg, 0.01 mmol, 4 mol%) then the reaction was stirred under an atmosphere of hydrogen at room temperature. After stirred for 16 hours, the reaction mixture was filtered through Celite, concentrated *in vacuo*, and purified by flash chromatography (50% ethyl acetate in hexane) to yield the title compound **72g** (67 mg, 0.17 mmol, 98%) as white solid. LRMS (ESI) *m*/*z*: 385.2 $[M+H]^+$.

2-{[5-(3-Aminophenyl)-6-(4-{[2-(dimethylamino)ethyl](methyl)amino}phenyl)furo[2,3-*d*]pyrimidin-4-yl]amino}ethanol (72h).

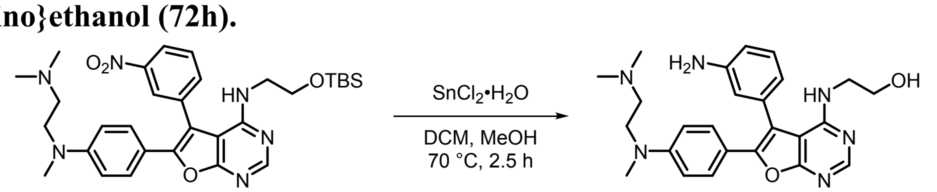

71h **72h**

To a solution of **71h** (212 mg, 0.36 mmol, 1.0 equiv.) in methanol (1.8 mL) and dichloromethane (1.8 mL) was added tin(II) chloride dihydrate (325 mg, 1.44 mmol,

4.0 equiv.) then the reaction mixture was stirred at 70 °C. After stirred for 2.5 hours, the reaction mixture was cooled down to room temperature, concentrated *in vacuo*, quenched with 50% $NaOH_{(aq)}$ until the pH value > 9, filtered through Celite, extracted into ethyl acetate (10 mL × 3). The organic layers were combined, washed with brine (10 mL), dried over $MgSO_4$, concentrated *in vacuo*, and purified by CombiFlash automated flash chromatography (5–10% methanol in dichloromethane with 0.1% NH_4OH) to yield the title compound **72h** (125 mg, 0.28 mmol, 78%) as yellow oil. LRMS (ESI) *m*/*z*: 447.1 $[M+H]^+$.

5-(3-Aminophenyl)-*N*-cyclopentyl-6-[4-(morpholin-4-yl)phenyl]furo[2,3-*d*]pyrimidin-4-amine (72i).

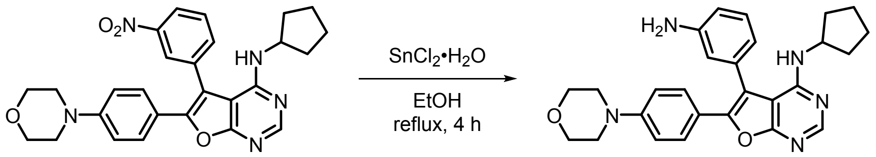

71i **72i**

To a solution of **71i** (90 mg, 0.19 mmol, 1.0 equiv.) in ethanol (5.0 mL) was added tin(II) chloride dihydrate (84 mg, 0.37 mmol, 2.0 equiv.) then the reaction mixture was stirred at reflux. After stirred for 4 hours, the reaction mixture was cooled down to room temperature and concentrated *in vacuo*. Then the reaction mixture was re-dissolved in ethyl acetate (5 mL), washed with $NaHCO_{3(aq)}$ (10 mL) and brine (10 mL). The combined organic layers were dried over $MgSO_4$, concentrated *in vacuo* to yield the title compound **72i** (84 mg, 0.18 mmol, 99%) as yellow solid. LRMS (ESI) *m*/*z*: 456.1 $[M+H]^+$.

5-(3-Aminophenyl)-*N*-cyclopentyl-6-[4-(4-methylpiperazin-1-yl)phenyl]furo[2,3-*d*]pyrimidin-4-amine (72j).

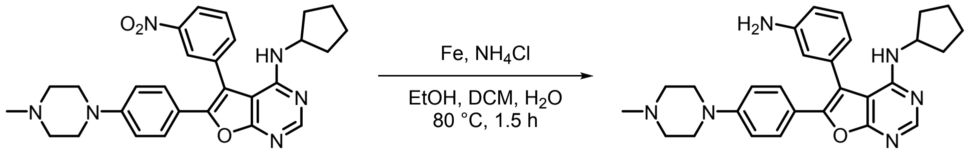

71j **72j**

To a solution of **71j** (67 mg, 0.13 mmol, 1.0 equiv.) in ethanol (3 mL), dichloromethane (3 mL) and water (0.6 mL) was added iron powder (22 mg, 0.40 mmol, 2.9 equiv.) and sat. $NH_4Cl_{(aq)}$ (0.3 mL) then the reaction mixture was stirred at 80 °C. After stirred for 1.5 hours, the reaction mixture was cooled down to room temperature, filtered through Celite, and concentrated *in vacuo*. Then the mixture was dissolved in dichloromethane (10 mL), washed with $NaHCO_3$(aq) (20 mL) and brine (10 mL), dried over $MgSO_4$, concentrated *in vacuo*, and purified by flash chromatography (5% methanol in dichloromethane with 0.1% NH_4OH) to yield the

title compound **72j** (40 mg, 0.09 mmol, 64%) as yellow solid. LRMS (ESI) *m*/*z*: 469.3 $[M+H]^+$.

(2*S*)-2-{[5-(3-Aminophenyl)-6-[4-(4-methylpiperazin-1-yl)phenyl]furo[2,3-*d*]pyrimidin-4-yl]amino}-2-phenylethanol (72k).

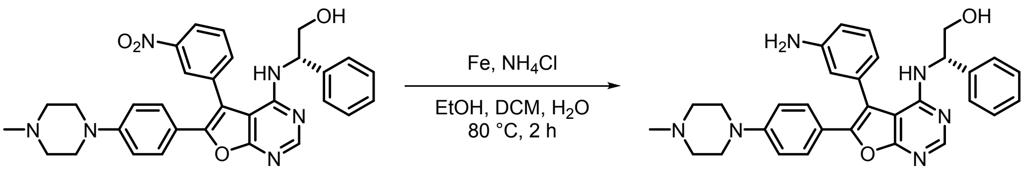

71k **72k**

To a solution of **71k** (333 mg, 0.60 mmol, 1.0 equiv.) in ethanol (6.3 mL), dichloromethane (6.3 mL) and water (1.36 mL) was added iron powder (135 mg, 2.42 mmol, 4.0 equiv.) and sat. $NH_4Cl_{(aq)}$ (0.63 mL) then the reaction mixture was stirred at 80 °C. After stirred for 2 hours, the reaction mixture was cooled down to room temperature, filtered through Celite, and concentrated *in vacuo*, and purified by Combiflash automated flash chromatography (5–15% methanol in dichloromethane with 0.1% NH_4OH) to yield the title compound **72k** (268 mg, 0.51 mmol, 85%) as yellow solid. LRMS (ESI) *m*/*z*: 521.3 $[M+H]^+$.

5-(3-Aminophenyl)-*N*-cyclohexyl-6-[4-(4-methylpiperazin-1-yl)phenyl]furo[2,3-*d*]pyrimidin-4-amine (72l).

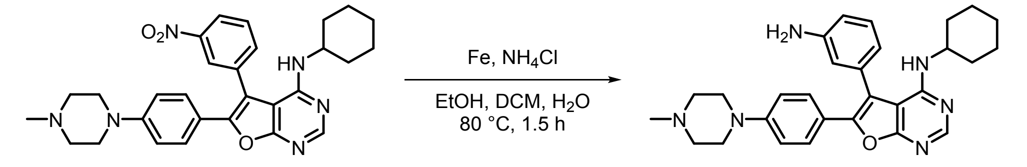

71l **72l**

To a solution of **71l** (330 mg, 0.64 mmol, 1.0 equiv.) in ethanol (5 mL), dichloromethane (5 mL) and water (1 mL) was added iron powder (107 mg, 1.93 mmol, 2.9 equiv.) and sat. $NH_4Cl_{(aq)}$ (0.5 mL) then the reaction mixture was stirred at 80 °C. After stirred for 1.5 hours, the reaction mixture was cooled down to room temperature, filtered through Celite, and concentrated *in vacuo*. Then the mixture was dissolved in dichloromethane (10 mL), washed with $NaHCO_{3(aq)}$ (20 mL) and brine (10 mL), dried over $MgSO_4$, concentrated *in vacuo*, and purified by flash chromatography (5% methanol in dichloromethane with 0.1% NH_4OH) to yield the title compound **72l** (130 mg, 0.27 mmol, 42%) as yellow solid. LRMS (ESI) *m*/*z*: 483.2 $[M+H]^+$.

5-(3-Aminophenyl)-*N*-cyclohexyl-6-[4-(4-methylpiperazin-1-yl)phenyl]furo[2,3-*d*]pyrimidin-4-amine (72m).

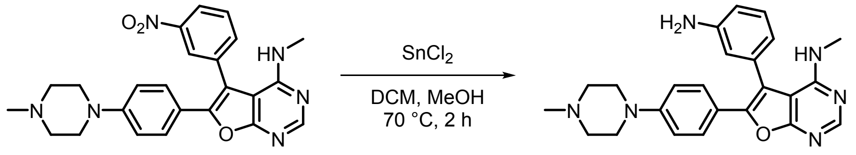

71m **72m**

To a solution of **71m** (100 mg, 0.22 mmol, 1.0 equiv.) in methanol (1.5 mL) and dichloromethane (1.5 mL) was added tin(II) chloride (202 mg, 1.07 mmol, 4.7 equiv.) then the reaction mixture was stirred at 70 °C. After stirred for 2 hours, the reaction mixture was cooled down to room temperature, filtered through Celite, and concentrated *in vacuo*. Then the mixture was dissolved in ethyl acetate (10 mL), washed with $NaHCO_{3(aq)}$ (20 mL) and brine (10 mL). The combined organic layers were dried over $MgSO_4$, concentrated *in vacuo*, and purified by flash chromatography (2–4% methanol in dichloromethane) to yield the title compound **72m** (60 mg, 0.14 mmol, 64%) as pale yellow solid. LRMS (ESI) *m*/*z*: 415.2 $[M+H]^+$.

5-(3-Aminophenyl)-*N*-cyclopentyl-6-(pyridin-4-yl)furo[2,3-*d*]pyrimidin-4-amine (72n).

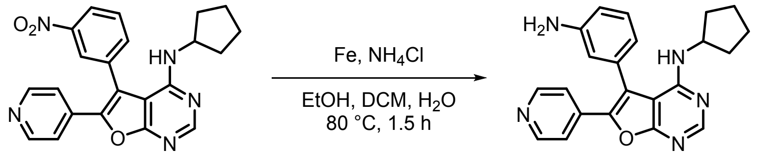

71n **72n**

To a solution of **71n** (79 mg, 0.20 mmol, 1.0 equiv.) in ethanol (3 mL), dichloromethane (3 mL) and water (0.6 mL) was added iron powder (33 mg, 0.59 mmol, 3.0 equiv.) and sat. $NH_4Cl_{(aq)}$ (0.3 mL) then the reaction mixture was stirred at 80 °C. After stirred for 1.5 hours, the reaction mixture was cooled down to room temperature, filtered through Celite, and concentrated *in vacuo*. Then the mixture was dissolved in dichloromethane (10 mL), washed with $NaHCO_{3(aq)}$ (20 mL) and brine (10 mL). The combined organic layers were dried over $MgSO_4$, concentrated *in vacuo*, and purified by flash chromatography (5% methanol in dichloromethane with 0.1% NH_4OH) to yield the title compound **72n** (28 mg, 0.08 mmol, 38%) as yellow solid. LRMS (ESI) *m*/*z*: 372.2 $[M+H]^+$.

5-(3-Aminophenyl)-*N*-cyclopentyl-6-[6-(morpholin-4-yl)pyridin-3-yl]furo[2,3-*d*]pyrimidin-4-amine (72o).

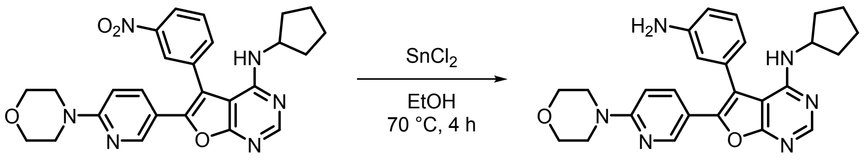

71o **72o**

To a solution of **71o** (69 mg, 0.14 mmol, 1.0 equiv.) in ethanol (3.5 mL) was added tin(II) chloride (64 mg, 0.28 mmol, 2.0 equiv.) then the reaction mixture was stirred at 70 °C. After stirred for 4 hours, the reaction mixture was cooled down to room temperature, filtered through Celite, and concentrated *in vacuo*. Then the mixture was dissolved in ethyl acetate (10 mL), washed with $NaHCO_{3(aq)}$ (20 mL) and brine (10 mL). The combined organic layers were dried over $MgSO_4$, concentrated *in vacuo*, and purified by flash chromatography (3% methanol in dichloromethane) to yield the title compound **72o** (62 mg, 0.14 mmol, 96%) as brown solid. LRMS (ESI) *m*/*z*: 457.3 $[M+H]^+$.

5-(3-Aminophenyl)-*N*-cyclopentyl-6-[6-(4-methylpiperazin-1-yl)pyridin-3-yl]furo[2,3-*d*]pyrimidin-4-amine (72p).

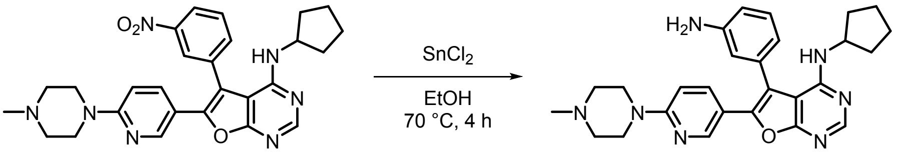

71p **72p**

To a solution of **71p** (87 mg, 0.18 mmol, 1.0 equiv.) in ethanol (3.5 mL) was added tin(II) chloride (79 mg, 0.35 mmol, 2.0 equiv.) then the reaction mixture was stirred at 70 °C. After stirred for 4 hours, the reaction mixture was cooled down to room temperature, filtered through Celite, and concentrated *in vacuo*. Then the mixture was dissolved in ethyl acetate (10 mL), washed with $NaHCO_{3(aq)}$ (20 mL) and brine (10 mL). The combined organic layers were dried over $MgSO_4$, concentrated *in vacuo*, and purified by flash chromatography (3% methanol in dichloromethane) to yield the title compound **72p** (77 mg, 0.16 mmol, 92%) as brown solid. LRMS (ESI) *m*/*z*: 470.3 $[M+H]^+$.

3. ^{1}H and ^{13}C spectra of compounds 13–58

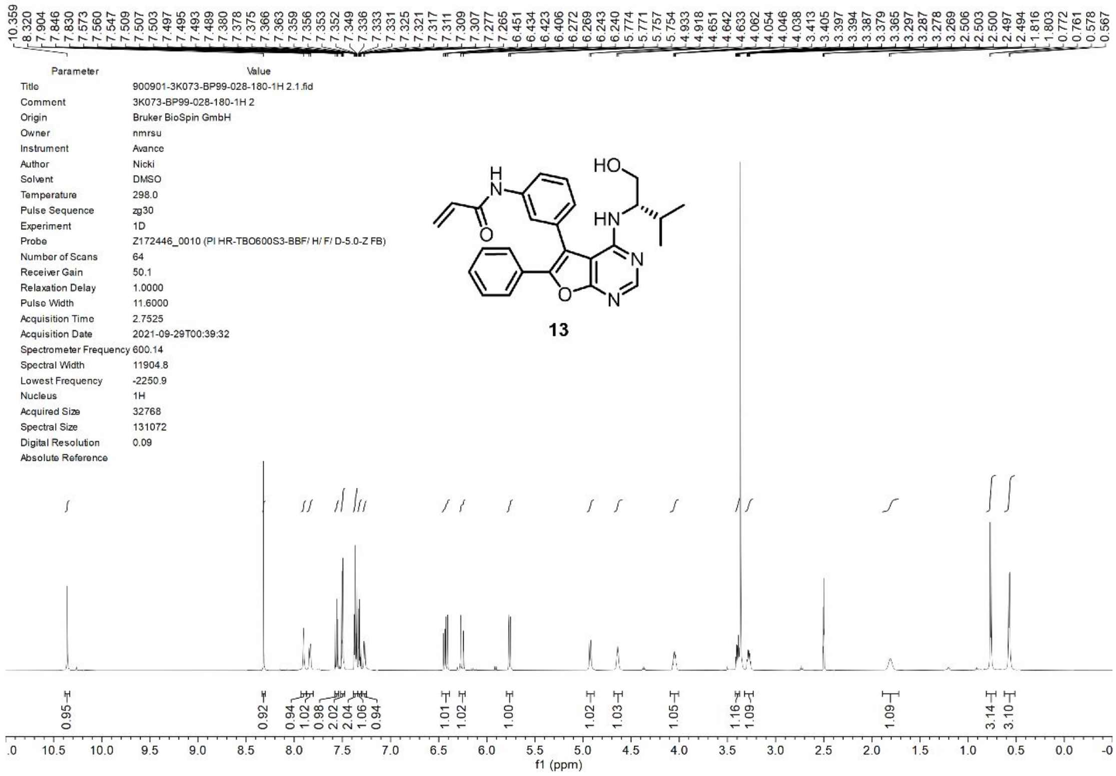

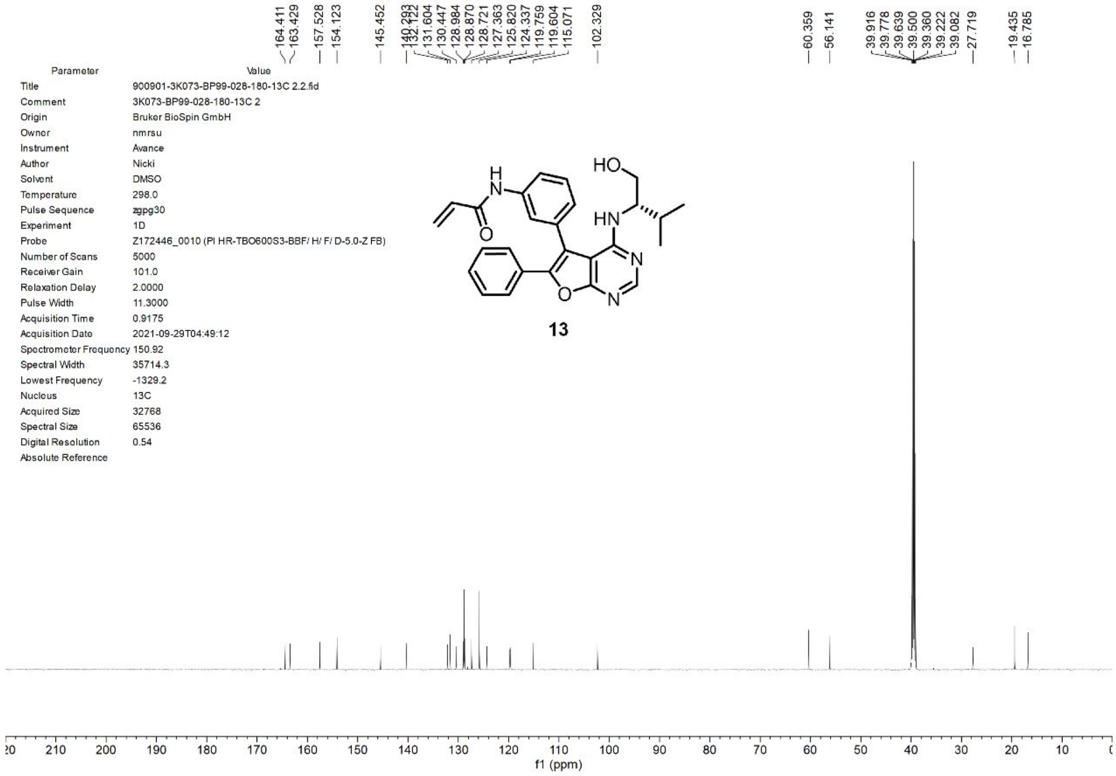

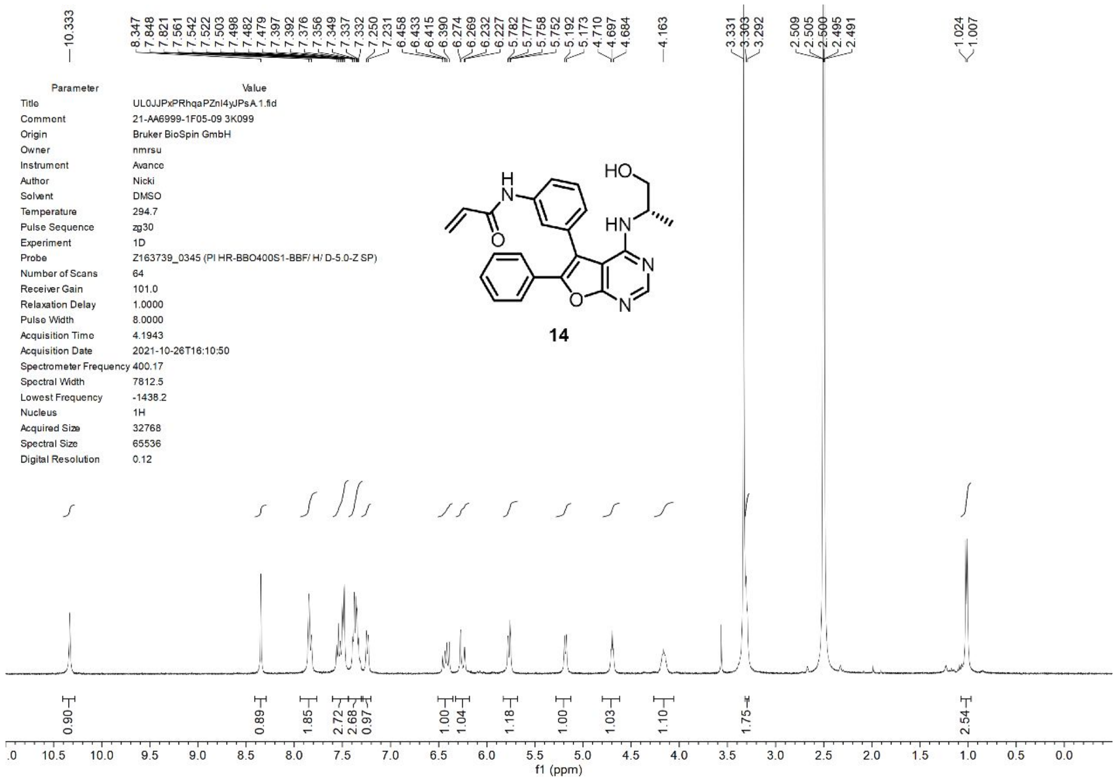
10.333
8.347 7.848 7.821 7.561 7.542 7.522 7.503 7.498 7.482 7.479 7.397 7.392 7.376 7.356 7.349 7.337 7.332 7.250 7.231 6.458 6.433 6.415 6.390 6.274 6.269 6.232 6.227 5.782 5.777 5.758 5.752 5.192 5.173 4.710 4.697 4.684
4.163
3.331 3.303 3.292
2.509 2.505 2.500 2.495 2.491
1.024 1.007
Parameter Value
Title UL0JJPxPRhqaPZnl4yJPsA.1.fid
Comment 21-AA6999-1F05-09 3K099
Origin Bruker BioSpin GmbH
Owner nmrsu
Instrument Avance
Author Nicki
Solvent DMSO
Temperature 294.7
Pulse Sequence zg30
Experiment 1D
Probe Z163739_0345 (PI HR-BBO400S1-BBF/ H/ D-5.0-Z SP)
Number of Scans 64
Receiver Gain 101.0
Relaxation Delay 1.0000
Pulse Width 8.0000
Acquisition Time 4.1943
Acquisition Date 2021-10-26T16:10:50
Spectrometer Frequency 400.17
Spectral Width 7812.5
Lowest Frequency -1438.2
Nucleus 1H
Acquired Size 32768
Spectral Size 65536
Digital Resolution 0.12
HO
HN
N
O
14
0.90 0.89 1.85 2.72 2.68 0.97 1.00 1.04 1.18 1.00 1.03 1.10 1.75 2.54
f1 (ppm)

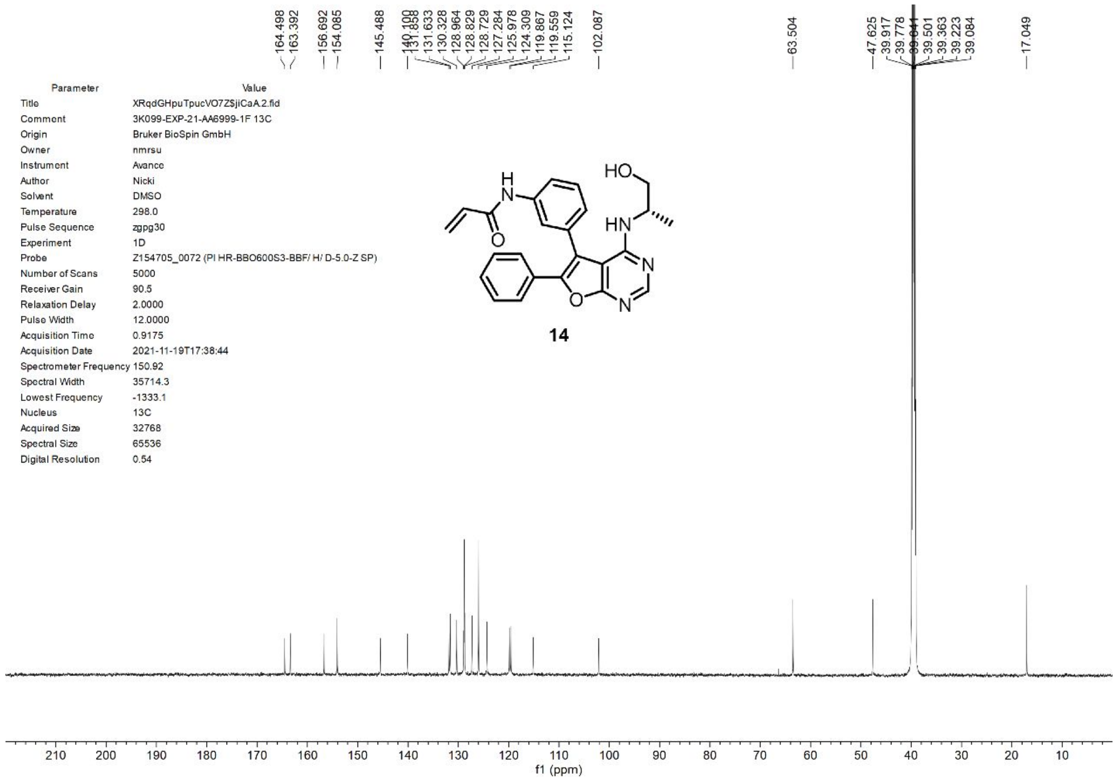
164.498 163.392 156.692 154.085 145.488 140.100 131.858 131.633 130.328 128.964 128.829 128.729 127.284 125.978 124.309 119.867 119.559 115.124 102.087
63.504
47.625 39.917 39.778 39.501 39.363 39.223 39.084
17.049
Parameter Value
Title XRqdGHpuTpucVO7ZSjiCaA.2.fid
Comment 3K099-EXP-21-AA6999-1F 13C
Origin Bruker BioSpin GmbH
Owner nmrsu
Instrument Avance
Author Nicki
Solvent DMSO
Temperature 298.0
Pulse Sequence zgpg30
Experiment 1D
Probe Z154705_0072 (PI HR-BBO600S3-BBF/ H/ D-5.0-Z SP)
Number of Scans 5000
Receiver Gain 90.5
Relaxation Delay 2.0000
Pulse Width 12.0000
Acquisition Time 0.9175
Acquisition Date 2021-11-19T17:38:44
Spectrometer Frequency 150.92
Spectral Width 35714.3
Lowest Frequency -1333.1
Nucleus 13C
Acquired Size 32768
Spectral Size 65536
Digital Resolution 0.54
HO
HN
N
O
14
f1 (ppm)

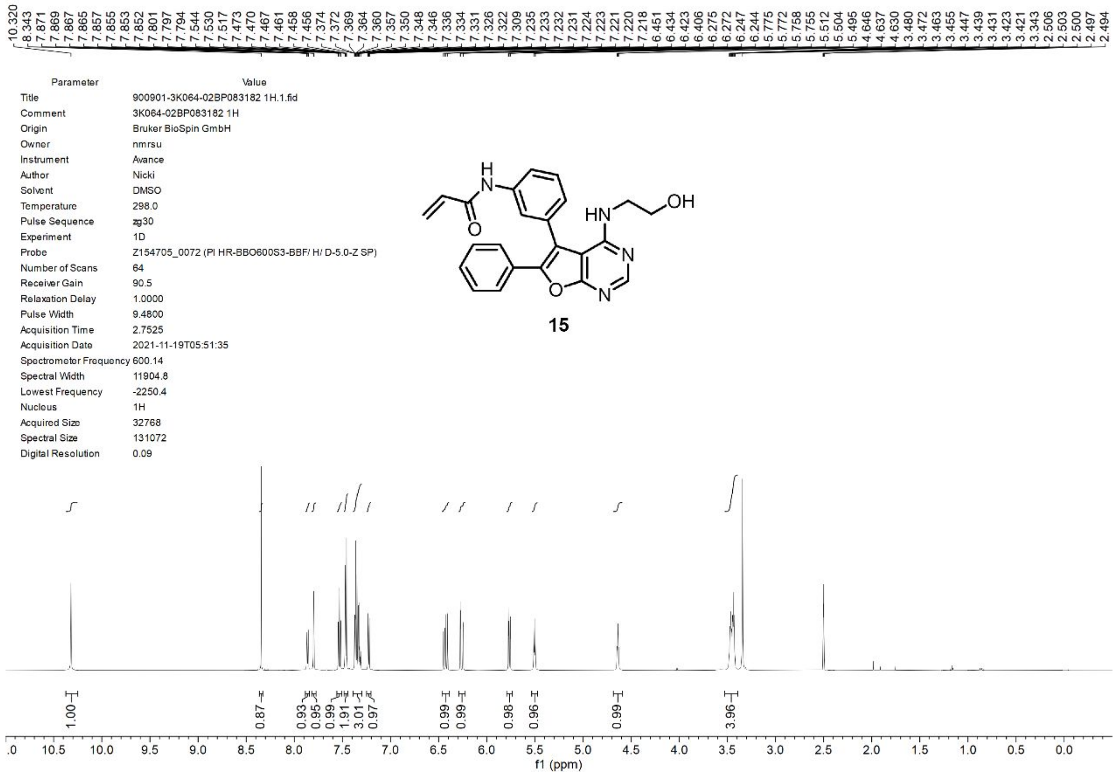

Parameter	Value
Title	900901-3K064-02BP083182 1H.1.fid
Comment	3K064-02BP083182 1H
Origin	Bruker BioSpin GmbH
Owner	nmrsu
Instrument	Avance
Author	Nicki
Solvent	DMSO
Temperature	298.0
Pulse Sequence	zg30
Experiment	1D
Probe	Z154705_0072 (PI HR-BBO600S3-BBF/ H/ D-5.0-Z SP)
Number of Scans	64
Receiver Gain	90.5
Relaxation Delay	1.0000
Pulse Width	9.4800
Acquisition Time	2.7525
Acquisition Date	2021-11-19T05:51:35
Spectrometer Frequency	600.14
Spectral Width	11904.8
Lowest Frequency	-2250.4
Nucleus	1H
Acquired Size	32768
Spectral Size	131072
Digital Resolution	0.09

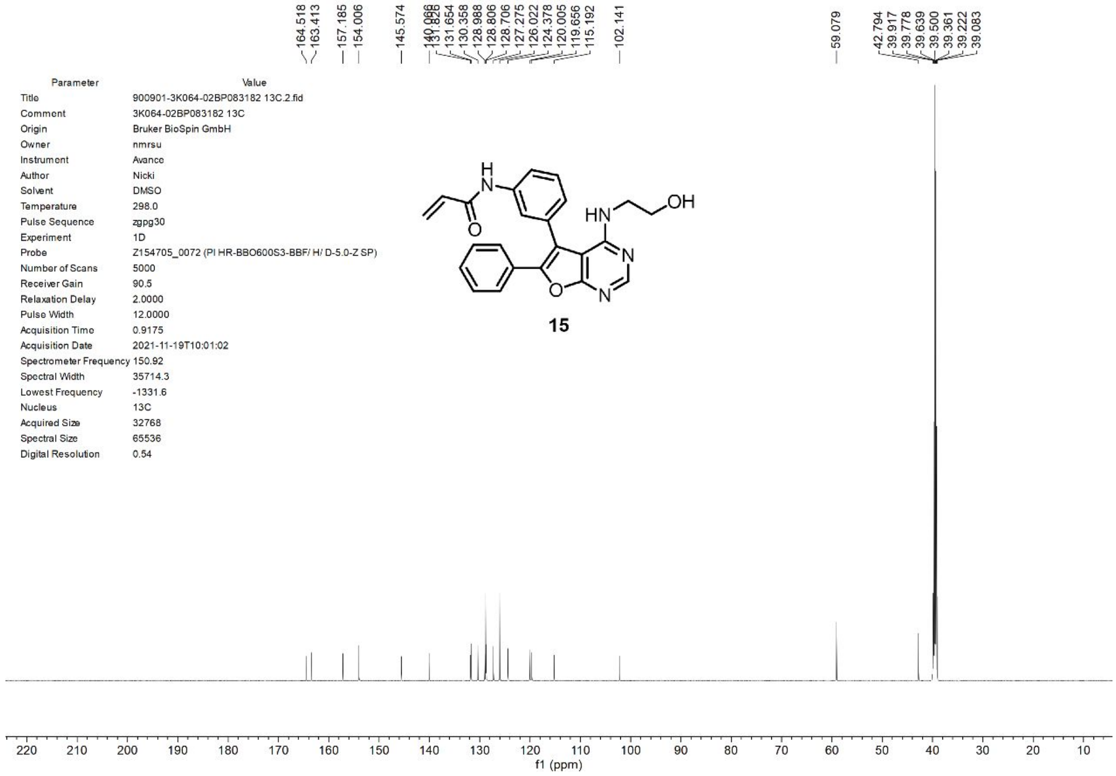

Parameter	Value
Title	900901-3K064-02BP083182 13C.2.fid
Comment	3K064-02BP083182 13C
Origin	Bruker BioSpin GmbH
Owner	nmrsu
Instrument	Avance
Author	Nicki
Solvent	DMSO
Temperature	298.0
Pulse Sequence	zgpg30
Experiment	1D
Probe	Z154705_0072 (PI HR-BBO600S3-BBF/ H/ D-5.0-Z SP)
Number of Scans	5000
Receiver Gain	90.5
Relaxation Delay	2.0000
Pulse Width	12.0000
Acquisition Time	0.9175
Acquisition Date	2021-11-19T10:01:02
Spectrometer Frequency	150.92
Spectral Width	35714.3
Lowest Frequency	-1331.6
Nucleus	13C
Acquired Size	32768
Spectral Size	65536
Digital Resolution	0.54

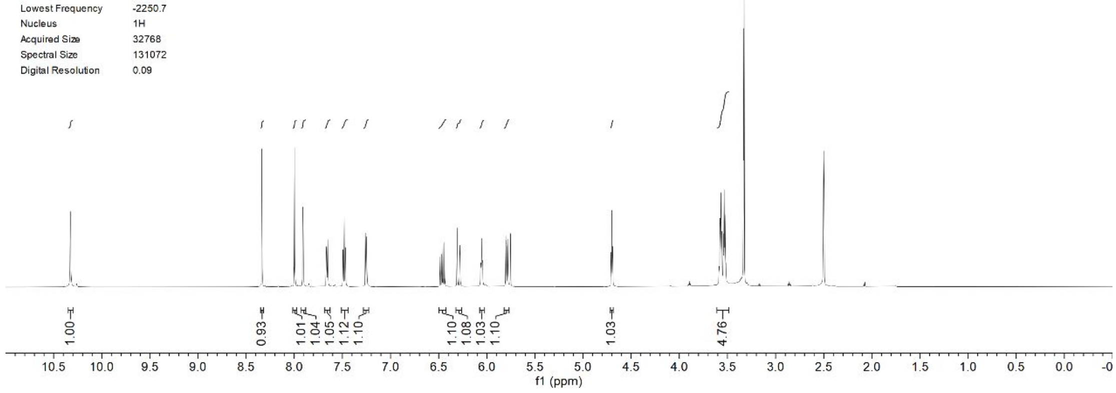

Parameter	Value
Title	rzovmktoTL6g2BV005sKLA.1.fid
Comment	900901-3K189-03BP-052-068-1H
Origin	Bruker BioSpin GmbH
Owner	nmrsu
Instrument	Avance
Author	Nicki
Solvent	DMSO
Temperature	298.0
Pulse Sequence	zg30
Experiment	1D
Probe	Z154705_0072 (PI HR-BBO600S3-BBF/ H/ D-5.0-Z SP)
Number of Scans	64
Receiver Gain	101.0
Relaxation Delay	1.0000
Pulse Width	9.4800
Acquisition Time	2.7525
Acquisition Date	2021-11-28T04:55:41
Spectrometer Frequency	600.14
Spectral Width	11904.8
Lowest Frequency	-2250.7
Nucleus	1H
Acquired Size	32768
Spectral Size	131072
Digital Resolution	0.09

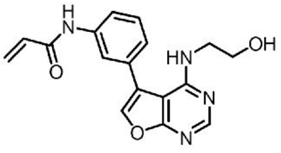

16

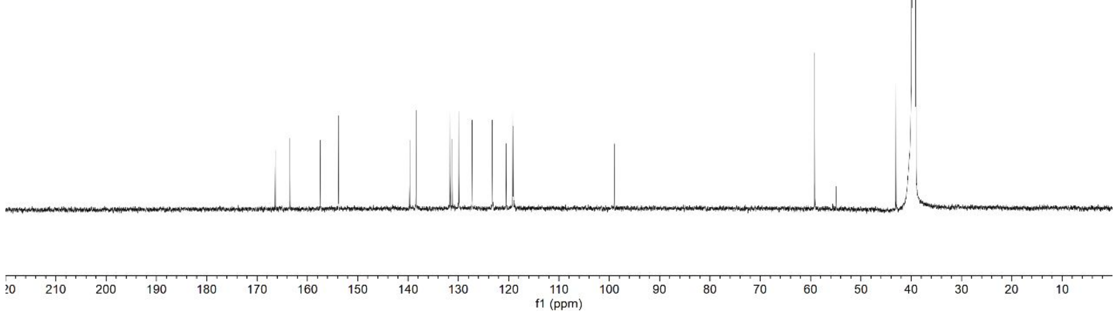

Parameter	Value
Title	tuAoum4FTp6E+vPnESmfqQ.2.fid
Comment	900901-3K189-03BP-052-068-13C
Origin	Bruker BioSpin GmbH
Owner	nmrsu
Instrument	Avance
Author	Nicki
Solvent	DMSO
Temperature	298.0
Pulse Sequence	zgpg30
Experiment	1D
Probe	Z154705_0072 (PI HR-BBO600S3-BBF/ H/ D-5.0-Z SP)
Number of Scans	5000
Receiver Gain	90.5
Relaxation Delay	2.0000
Pulse Width	12.0000
Acquisition Time	0.9175
Acquisition Date	2021-11-28T09:05:10
Spectrometer Frequency	150.92
Spectral Width	35714.3
Lowest Frequency	-1332.1
Nucleus	13C
Acquired Size	32768
Spectral Size	65536
Digital Resolution	0.54

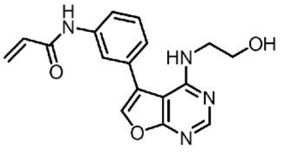

16

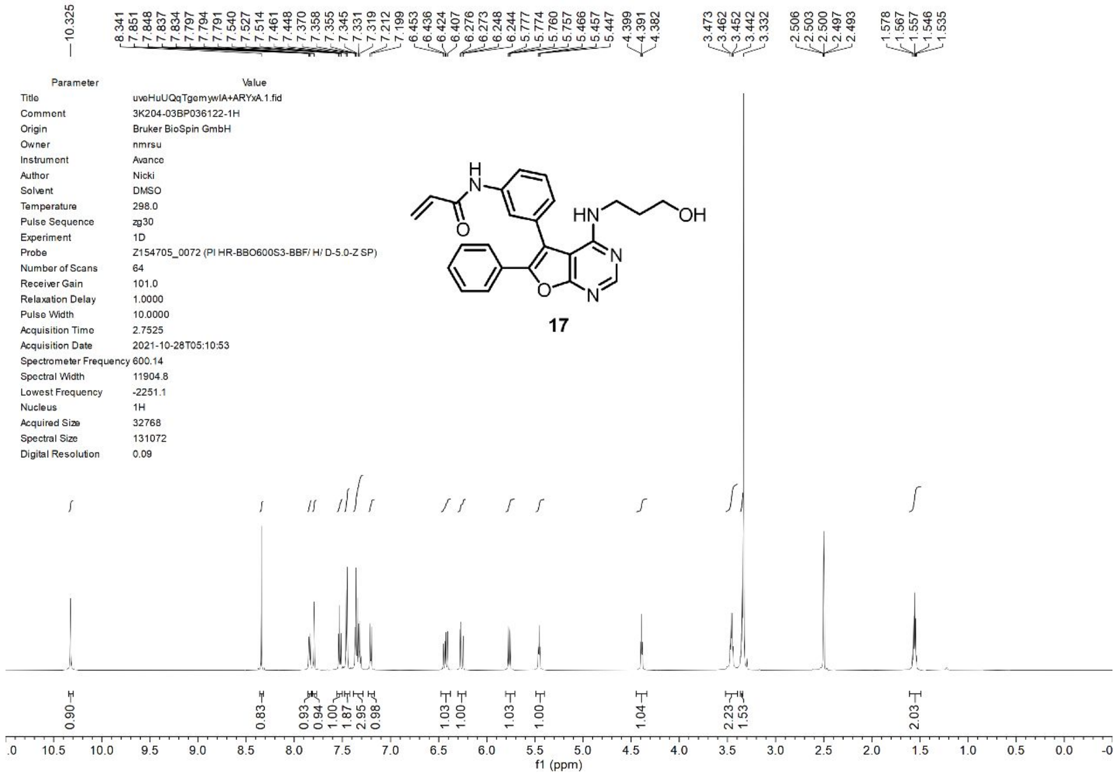
10.325
8.341 7.851 7.848 7.837 7.834 7.797 7.794 7.791 7.540 7.527 7.514 7.461 7.448 7.370 7.358 7.355 7.345 7.331 7.319 7.212 7.199 6.453 6.436 6.424 6.407 6.276 6.273 6.248 6.244 5.777 5.774 5.760 5.757 5.466 5.457 5.447 4.399 4.391 4.382
3.473 3.462 3.452 3.442 3.332 2.506 2.503 2.500 2.497 2.493 1.578 1.567 1.557 1.546 1.535
Parameter Value
Title uvoHuUQqTgomywIA+ARYxA.1.fid
Comment 3K204-03BP036122-1H
Origin Bruker BioSpin GmbH
Owner nmrsu
Instrument Avance
Author Nicki
Solvent DMSO
Temperature 298.0
Pulse Sequence zg30
Experiment 1D
Probe Z154705_0072 (PI HR-BBO600S3-BBF/ H/ D-5.0-Z SP)
Number of Scans 64
Receiver Gain 101.0
Relaxation Delay 1.0000
Pulse Width 10.0000
Acquisition Time 2.7525
Acquisition Date 2021-10-28T05:10:53
Spectrometer Frequency 600.14
Spectral Width 11904.8
Lowest Frequency -2251.1
Nucleus 1H
Acquired Size 32768
Spectral Size 131072
Digital Resolution 0.09
HN OH N O
17
0.90 0.83 0.93 0.94 1.00 1.87 2.95 0.98 1.03 1.00 1.03 1.00 1.04 2.23 1.53 2.03
f1 (ppm)

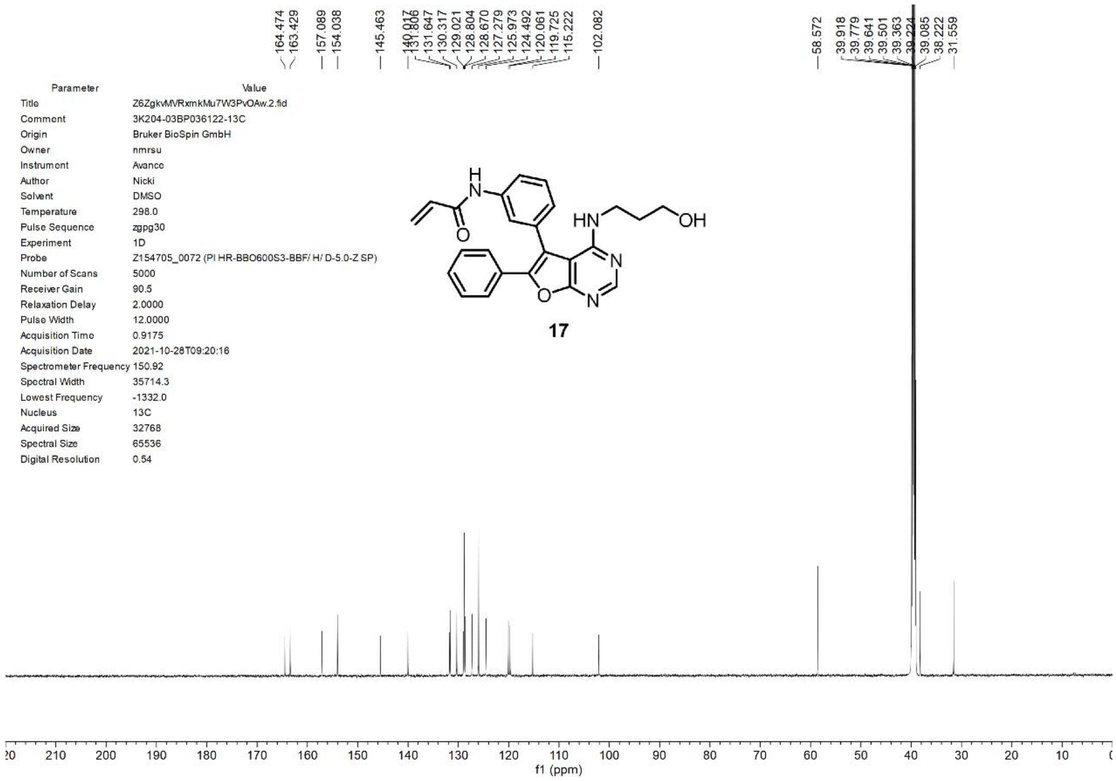
164.474 163.429 157.089 154.038 145.463 140.017 131.806 131.647 130.317 129.021 128.804 128.670 127.279 125.973 124.492 120.061 119.725 115.222 102.082
58.572 39.918 39.779 39.641 39.501 39.363 39.224 39.085 38.222 31.559
Parameter Value
Title Z6ZgkvMVRxmkMu7W3PvOAw.2.fid
Comment 3K204-03BP036122-13C
Origin Bruker BioSpin GmbH
Owner nmrsu
Instrument Avance
Author Nicki
Solvent DMSO
Temperature 298.0
Pulse Sequence zgpg30
Experiment 1D
Probe Z154705_0072 (PI HR-BBO600S3-BBF/ H/ D-5.0-Z SP)
Number of Scans 5000
Receiver Gain 90.5
Relaxation Delay 2.0000
Pulse Width 12.0000
Acquisition Time 0.9175
Acquisition Date 2021-10-28T09:20:16
Spectrometer Frequency 150.92
Spectral Width 35714.3
Lowest Frequency -1332.0
Nucleus 13C
Acquired Size 32768
Spectral Size 65536
Digital Resolution 0.54
HN OH N O
17
f1 (ppm)

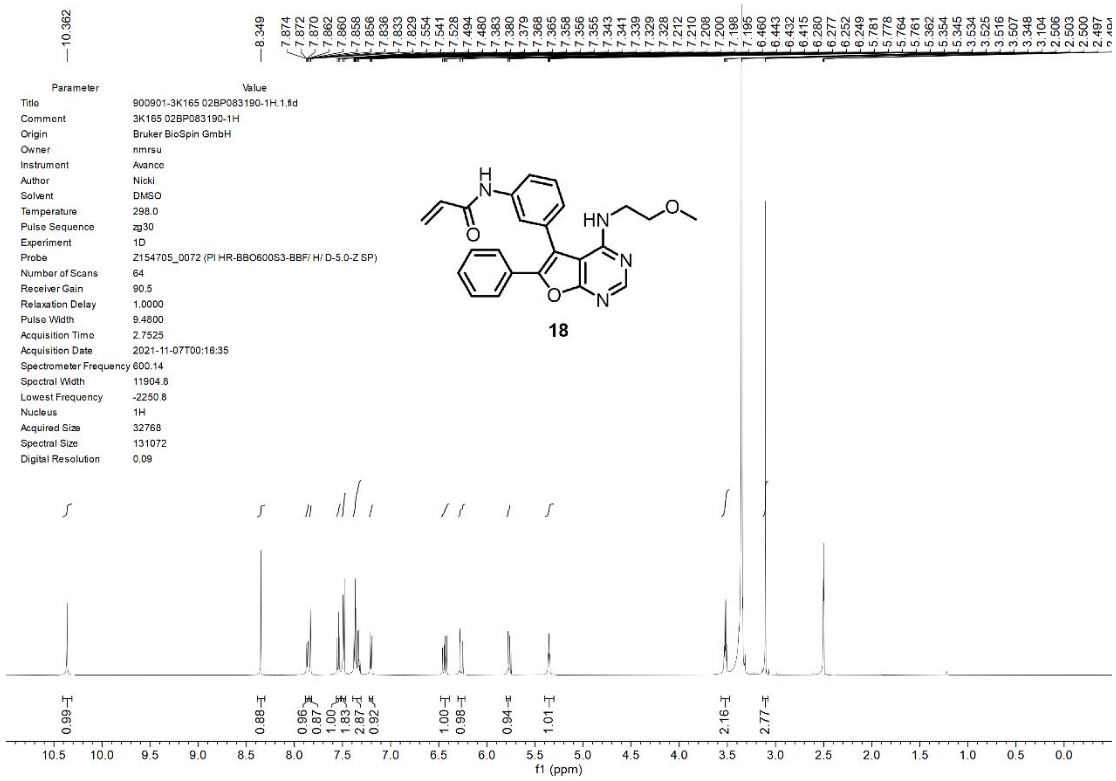
10.362
8.349
7.874 7.872 7.870 7.862 7.860 7.858 7.856 7.836 7.833 7.829 7.554 7.541 7.528 7.494 7.480 7.383 7.380 7.379 7.368 7.365 7.358 7.356 7.355 7.343 7.341 7.339 7.329 7.328 7.212 7.210 7.208 7.200 7.198 7.195 6.460 6.443 6.432 6.415 6.280 6.277 6.252 6.249 5.781 5.778 5.764 5.761 5.362 5.354 5.345 3.534 3.525 3.516 3.507 3.348 3.104 2.506 2.503 2.500 2.497
Parameter Value
Title 900901-3K165 02BP083190-1H.1.fid
Comment 3K165 02BP083190-1H
Origin Bruker BioSpin GmbH
Owner nmrsu
Instrument Avance
Author Nicki
Solvent DMSO
Temperature 298.0
Pulse Sequence zg30
Experiment 1D
Probe Z154705_0072 (PI HR-BBO600S3-BBF/ H/ D-5.0-Z SP)
Number of Scans 64
Receiver Gain 90.5
Relaxation Delay 1.0000
Pulse Width 9.4800
Acquisition Time 2.7525
Acquisition Date 2021-11-07T00:16:35
Spectrometer Frequency 600.14
Spectral Width 11904.8
Lowest Frequency -2250.8
Nucleus 1H
Acquired Size 32768
Spectral Size 131072
Digital Resolution 0.09
18
0.99 0.88 0.96 0.87 1.00 1.83 2.87 0.92 1.00 0.98 0.94 1.01 2.16 2.77
f1 (ppm)

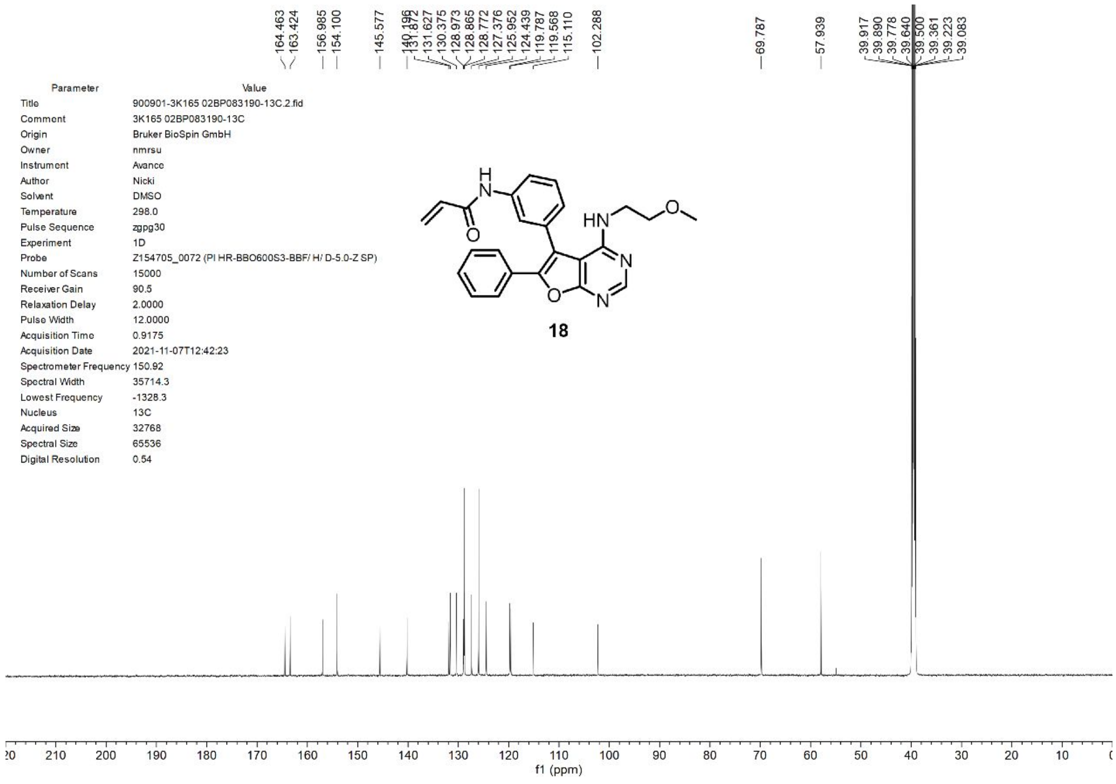
164.463 163.424 156.985 154.100 145.577 140.196 131.872 131.627 130.375 128.973 128.865 128.772 127.376 125.952 124.439 119.787 119.568 115.110 102.288 69.787 57.939 39.917 39.890 39.778 39.640 39.500 39.361 39.223 39.083
Parameter Value
Title 900901-3K165 02BP083190-13C.2.fid
Comment 3K165 02BP083190-13C
Origin Bruker BioSpin GmbH
Owner nmrsu
Instrument Avance
Author Nicki
Solvent DMSO
Temperature 298.0
Pulse Sequence zgpg30
Experiment 1D
Probe Z154705_0072 (PI HR-BBO600S3-BBF/ H/ D-5.0-Z SP)
Number of Scans 15000
Receiver Gain 90.5
Relaxation Delay 2.0000
Pulse Width 12.0000
Acquisition Time 0.9175
Acquisition Date 2021-11-07T12:42:23
Spectrometer Frequency 150.92
Spectral Width 35714.3
Lowest Frequency -1328.3
Nucleus 13C
Acquired Size 32768
Spectral Size 65536
Digital Resolution 0.54
18
f1 (ppm)

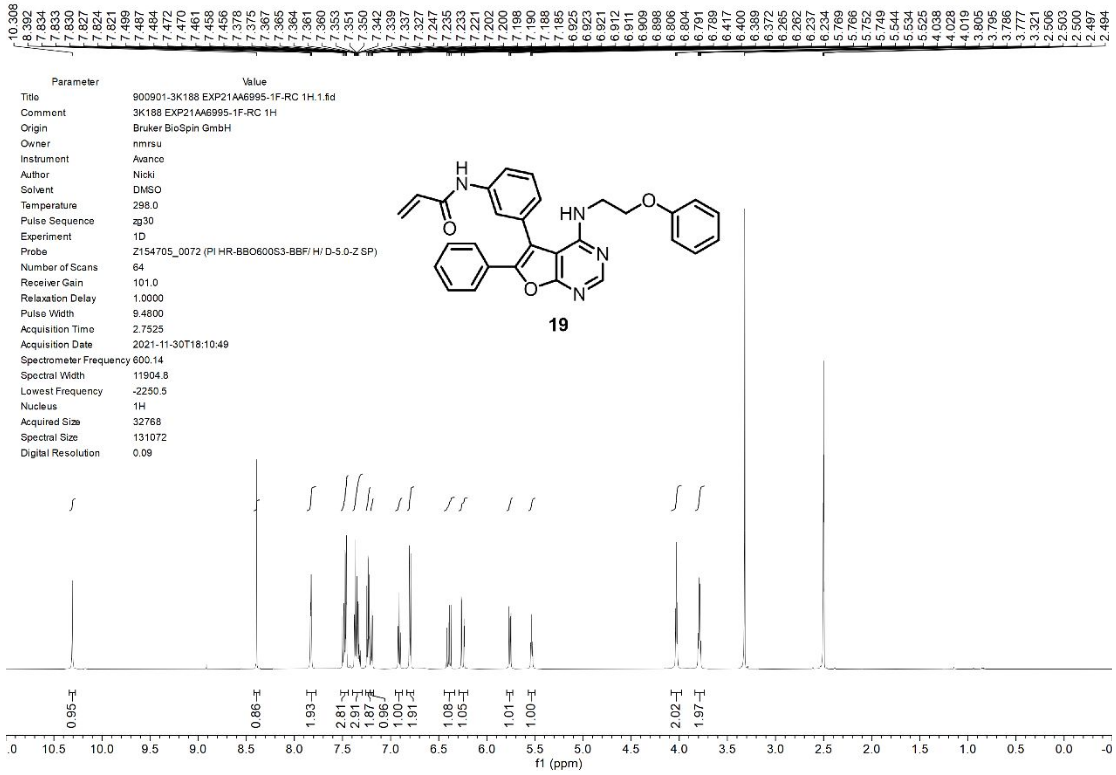

Parameter	Value
Title	900901-3K188 EXP21AA6995-1F-RC 1H.1.fid
Comment	3K188 EXP21AA6995-1F-RC 1H
Origin	Bruker BioSpin GmbH
Owner	nmrsu
Instrument	Avance
Author	Nicki
Solvent	DMSO
Temperature	298.0
Pulse Sequence	zg30
Experiment	1D
Probe	Z154705_0072 (PI HR-BBO600S3-BBF/ H/ D-5.0-Z SP)
Number of Scans	64
Receiver Gain	101.0
Relaxation Delay	1.0000
Pulse Width	9.4800
Acquisition Time	2.7525
Acquisition Date	2021-11-30T18:10:49
Spectrometer Frequency	600.14
Spectral Width	11904.8
Lowest Frequency	-2250.5
Nucleus	1H
Acquired Size	32768
Spectral Size	131072
Digital Resolution	0.09

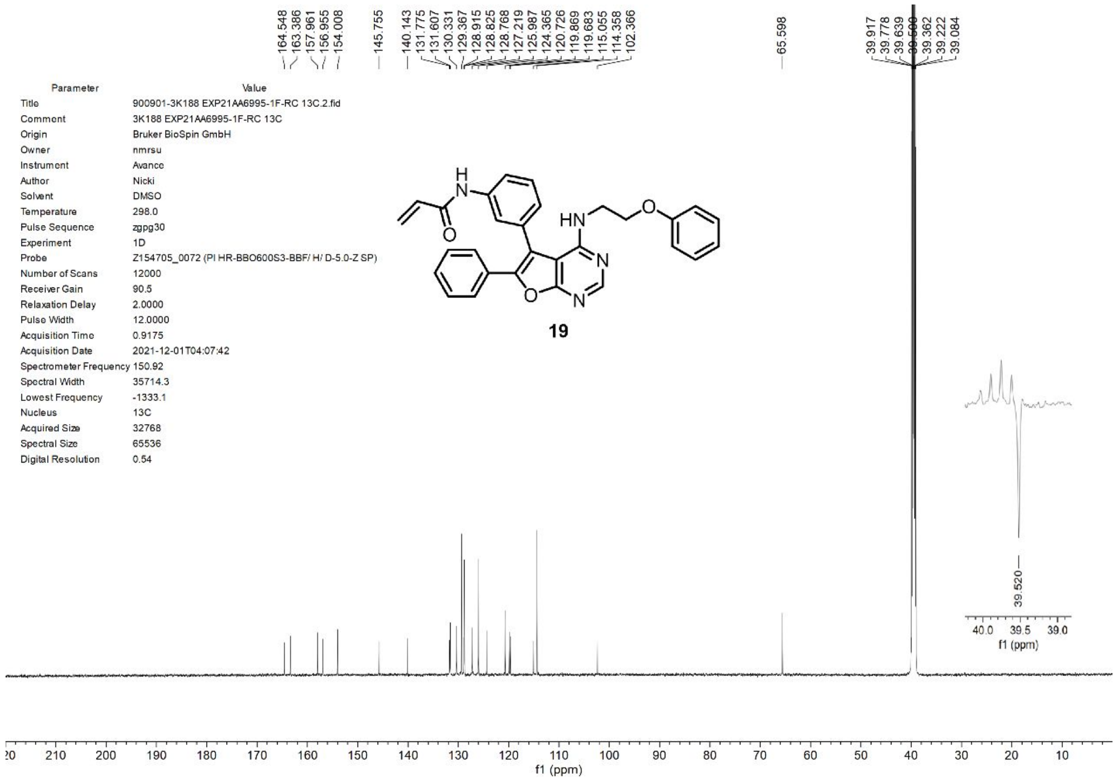

Parameter	Value
Title	900901-3K188 EXP21AA6995-1F-RC 13C.2.fid
Comment	3K188 EXP21AA6995-1F-RC 13C
Origin	Bruker BioSpin GmbH
Owner	nmrsu
Instrument	Avance
Author	Nicki
Solvent	DMSO
Temperature	298.0
Pulse Sequence	zgpg30
Experiment	1D
Probe	Z154705_0072 (PI HR-BBO600S3-BBF/ H/ D-5.0-Z SP)
Number of Scans	12000
Receiver Gain	90.5
Relaxation Delay	2.0000
Pulse Width	12.0000
Acquisition Time	0.9175
Acquisition Date	2021-12-01T04:07:42
Spectrometer Frequency	150.92
Spectral Width	35714.3
Lowest Frequency	-1333.1
Nucleus	13C
Acquired Size	32768
Spectral Size	65536
Digital Resolution	0.54

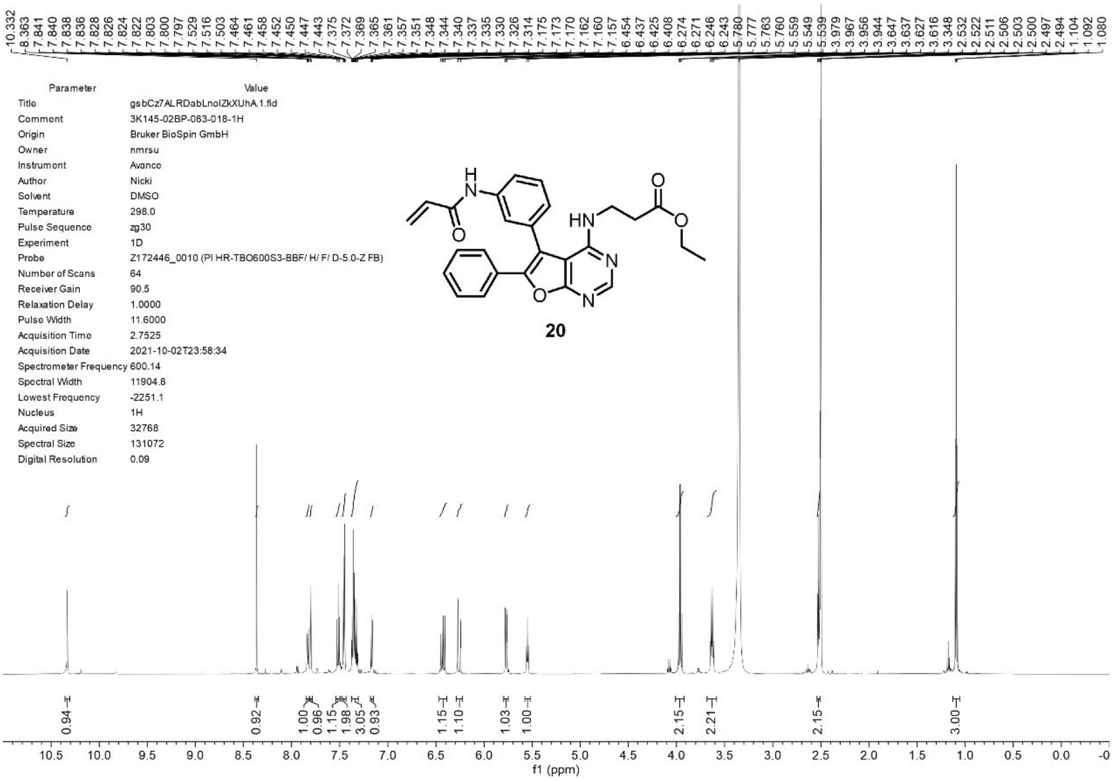
10.332, 8.363, 7.841, 7.840, 7.838, 7.836, 7.828, 7.826, 7.824, 7.822, 7.803, 7.800, 7.797, 7.529, 7.516, 7.503, 7.464, 7.461, 7.458, 7.452, 7.450, 7.447, 7.443, 7.375, 7.372, 7.369, 7.365, 7.361, 7.357, 7.351, 7.348, 7.344, 7.340, 7.337, 7.335, 7.330, 7.326, 7.314, 7.175, 7.173, 7.170, 7.162, 7.160, 7.157, 6.454, 6.437, 6.425, 6.408, 6.274, 6.271, 6.246, 6.243, 5.780, 5.777, 5.763, 5.760, 5.559, 5.549, 5.539, 3.979, 3.967, 3.956, 3.944, 3.647, 3.637, 3.627, 3.616, 3.348, 2.532, 2.522, 2.511, 2.506, 2.503, 2.500, 2.497, 2.494, 1.104, 1.092, 1.080
Parameter Value
Title gsbCz7ALRDabLnolZkXUhA.1.fid
Comment 3K145-02BP-083-018-1H
Origin Bruker BioSpin GmbH
Owner nmrsu
Instrument Avance
Author Nicki
Solvent DMSO
Temperature 298.0
Pulse Sequence zg30
Experiment 1D
Probe Z172446_0010 (PI HR-TBO600S3-BBF/ H/ F/ D-5.0-Z FB)
Number of Scans 64
Receiver Gain 90.5
Relaxation Delay 1.0000
Pulse Width 11.6000
Acquisition Time 2.7525
Acquisition Date 2021-10-02T23:58:34
Spectrometer Frequency 600.14
Spectral Width 11904.8
Lowest Frequency -2251.1
Nucleus 1H
Acquired Size 32768
Spectral Size 131072
Digital Resolution 0.09
20
0.94, 0.92, 1.00, 0.96, 1.15, 1.98, 3.05, 0.93, 1.15, 1.10, 1.03, 1.00, 2.15, 2.21, 2.15, 3.00
10.5 10.0 9.5 9.0 8.5 8.0 7.5 7.0 6.5 6.0 5.5 5.0 4.5 4.0 3.5 3.0 2.5 2.0 1.5 1.0 0.5 0.0
f1 (ppm)

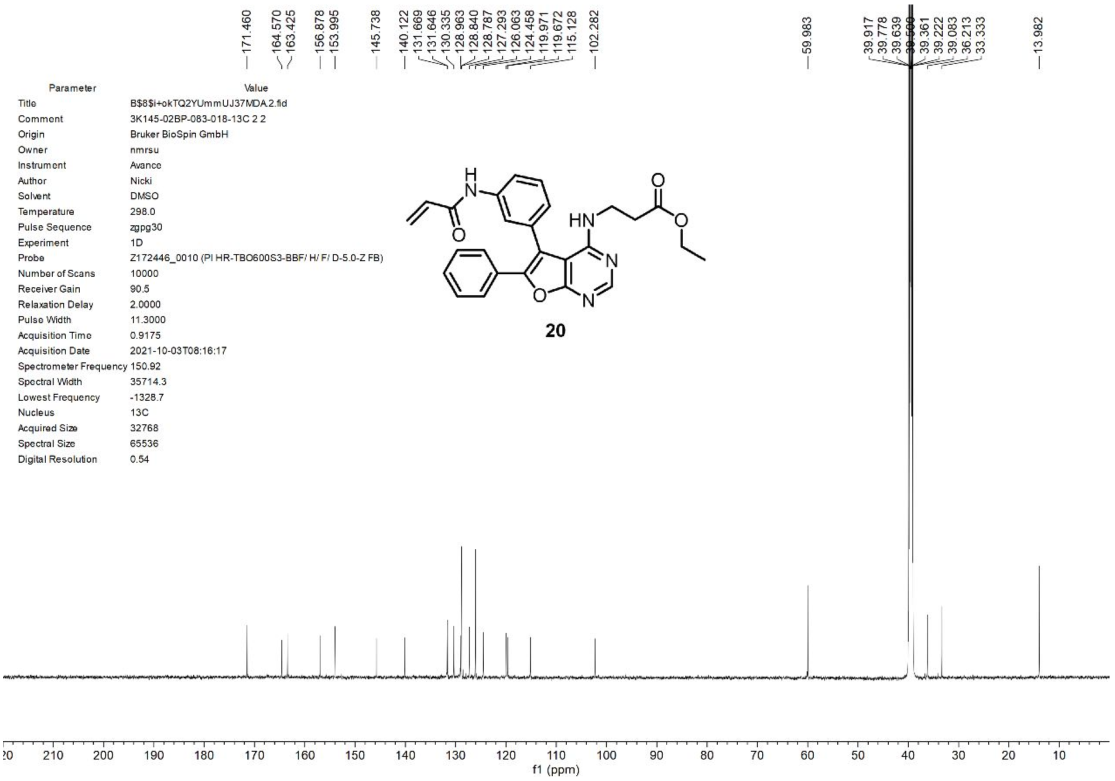
171.460, 164.570, 163.425, 156.878, 153.995, 145.738, 140.122, 131.669, 131.646, 130.335, 128.963, 128.840, 128.787, 127.293, 126.063, 124.458, 119.971, 119.672, 115.128, 102.282, 59.983, 39.917, 39.778, 39.639, 39.361, 39.222, 39.083, 36.213, 33.333, 13.982
Parameter Value
Title B8i+okTQ2YUmmUJ37MDA.2.fid
Comment 3K145-02BP-083-018-13C 2 2
Origin Bruker BioSpin GmbH
Owner nmrsu
Instrument Avance
Author Nicki
Solvent DMSO
Temperature 298.0
Pulse Sequence zgpg30
Experiment 1D
Probe Z172446_0010 (PI HR-TBO600S3-BBF/ H/ F/ D-5.0-Z FB)
Number of Scans 10000
Receiver Gain 90.5
Relaxation Delay 2.0000
Pulse Width 11.3000
Acquisition Time 0.9175
Acquisition Date 2021-10-03T08:16:17
Spectrometer Frequency 150.92
Spectral Width 35714.3
Lowest Frequency -1328.7
Nucleus 13C
Acquired Size 32768
Spectral Size 65536
Digital Resolution 0.54
20
210 200 190 180 170 160 150 140 130 120 110 100 90 80 70 60 50 40 30 20 10
f1 (ppm)

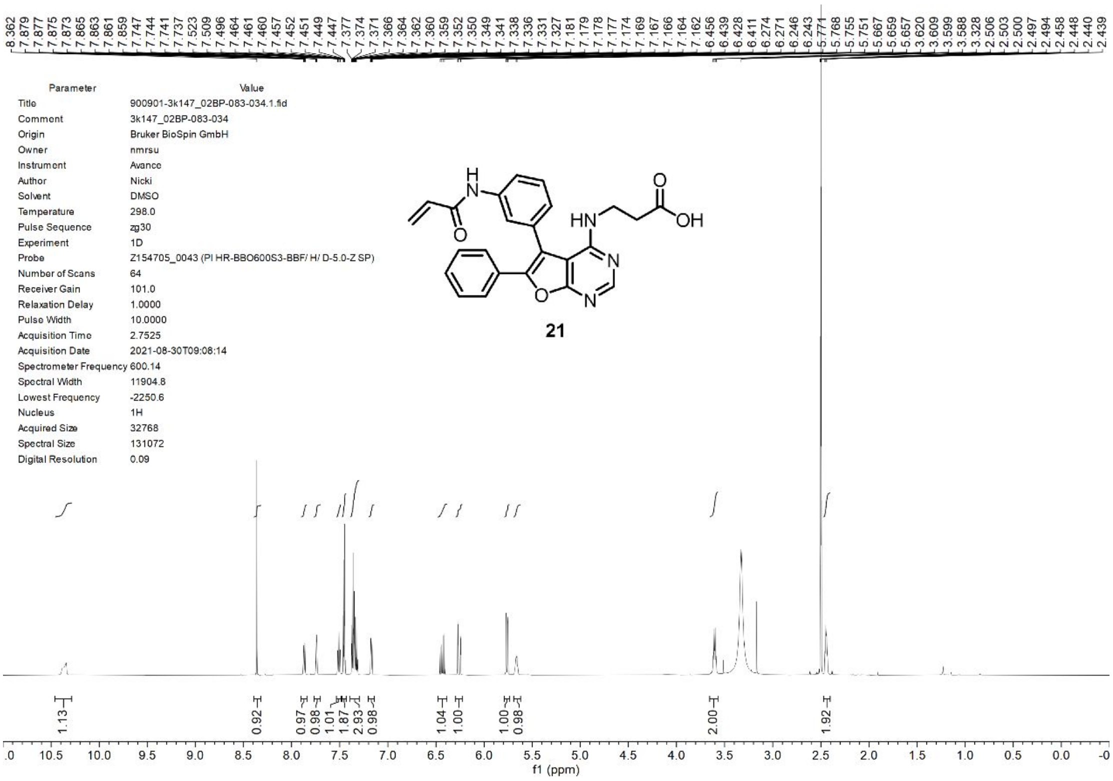
8.362 7.879 7.877 7.875 7.873 7.865 7.863 7.861 7.859 7.747 7.744 7.741 7.737 7.523 7.509 7.496 7.464 7.461 7.460 7.457 7.452 7.451 7.449 7.447 7.377 7.374 7.371 7.366 7.364 7.362 7.360 7.359 7.352 7.350 7.349 7.341 7.338 7.336 7.331 7.327 7.181 7.179 7.178 7.177 7.174 7.169 7.167 7.166 7.164 7.162 6.456 6.439 6.428 6.411 6.274 6.271 6.246 6.243 5.771 5.768 5.755 5.751 5.667 5.659 5.657 3.620 3.609 3.599 3.588 3.328 2.506 2.503 2.500 2.497 2.494 2.458 2.448 2.440 2.439
Parameter Value
Title 900901-3k147_02BP-083-034.1.fid
Comment 3k147_02BP-083-034
Origin Bruker BioSpin GmbH
Owner nmrsu
Instrument Avance
Author Nicki
Solvent DMSO
Temperature 298.0
Pulse Sequence zg30
Experiment 1D
Probe Z154705_0043 (PI HR-BBO600S3-BBF/ H/ D-5.0-Z SP)
Number of Scans 64
Receiver Gain 101.0
Relaxation Delay 1.0000
Pulse Width 10.0000
Acquisition Time 2.7525
Acquisition Date 2021-08-30T09:08:14
Spectrometer Frequency 600.14
Spectral Width 11904.8
Lowest Frequency -2250.6
Nucleus 1H
Acquired Size 32768
Spectral Size 131072
Digital Resolution 0.09
H N O HN O OH N N O
21
1.13 0.92 0.97 0.98 1.01 1.87 2.93 0.98 1.04 1.00 1.00 0.98 2.00 1.92
f1 (ppm)

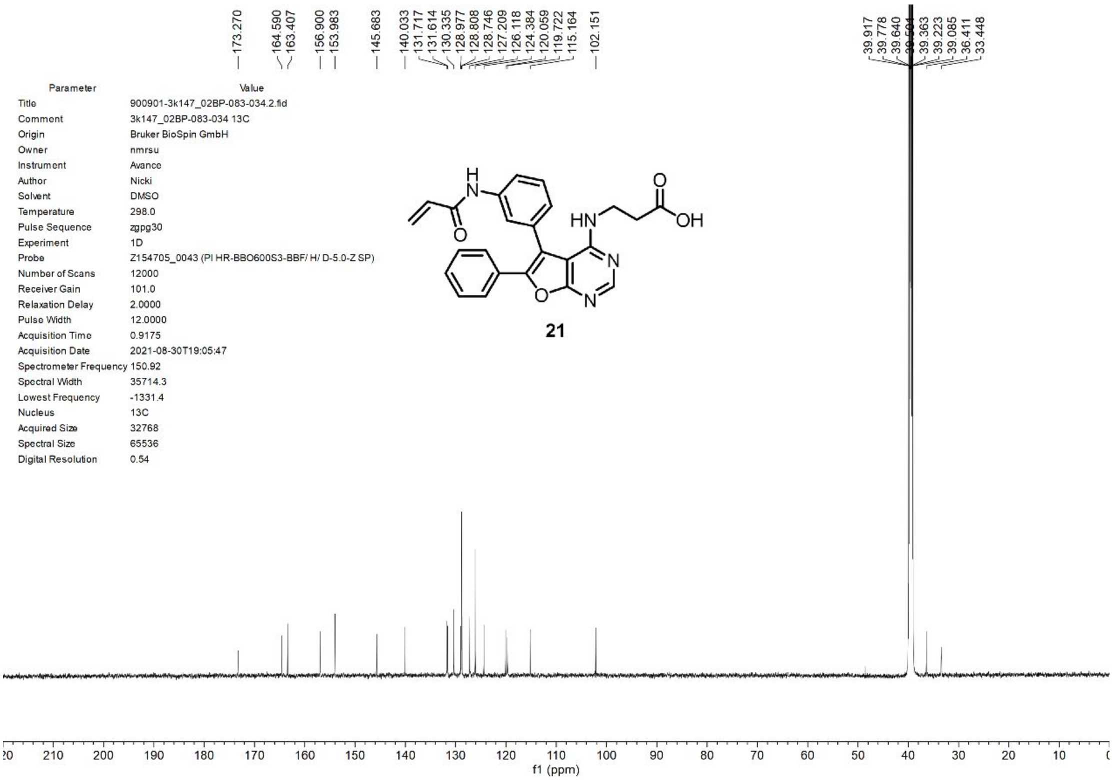
173.270 164.590 163.407 156.900 153.983 145.683 140.033 131.717 131.614 130.335 128.977 128.808 128.746 127.209 126.118 124.384 120.059 119.722 115.164 102.151 39.917 39.778 39.640 39.363 39.223 39.085 36.411 33.448
Parameter Value
Title 900901-3k147_02BP-083-034.2.fid
Comment 3k147_02BP-083-034 13C
Origin Bruker BioSpin GmbH
Owner nmrsu
Instrument Avance
Author Nicki
Solvent DMSO
Temperature 298.0
Pulse Sequence zgpg30
Experiment 1D
Probe Z154705_0043 (PI HR-BBO600S3-BBF/ H/ D-5.0-Z SP)
Number of Scans 12000
Receiver Gain 101.0
Relaxation Delay 2.0000
Pulse Width 12.0000
Acquisition Time 0.9175
Acquisition Date 2021-08-30T19:05:47
Spectrometer Frequency 150.92
Spectral Width 35714.3
Lowest Frequency -1331.4
Nucleus 13C
Acquired Size 32768
Spectral Size 65536
Digital Resolution 0.54
H N O HN O OH N N O
21
f1 (ppm)

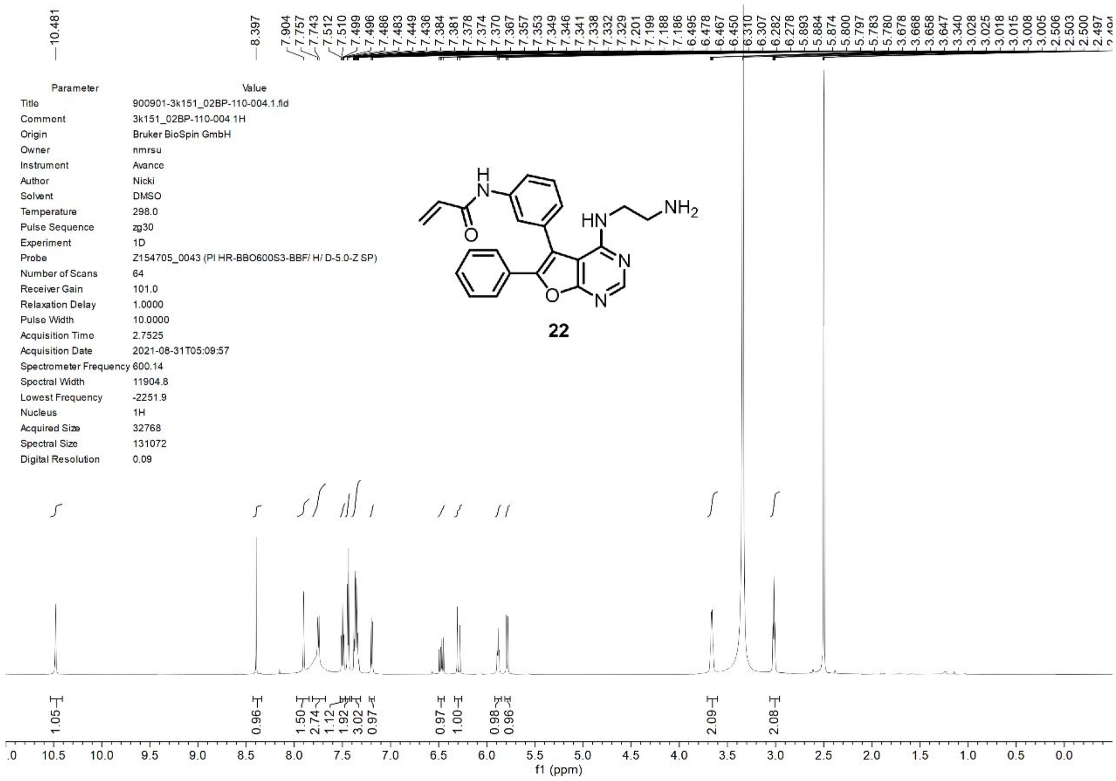
10.481
8.397
7.904 7.757 7.743 7.512 7.510 7.499 7.496 7.486 7.483 7.449 7.436 7.384 7.381 7.378 7.374 7.370 7.367 7.357 7.353 7.349 7.346 7.341 7.338 7.332 7.329 7.201 7.199 7.188 7.186 6.495 6.478 6.467 6.450 6.310 6.307 6.282 6.278 5.893 5.884 5.874 5.800 5.797 5.783 5.780 3.678 3.668 3.658 3.647 3.340 3.028 3.025 3.018 3.015 3.008 3.005 2.506 2.503 2.500 2.497
Parameter Value
Title 900901-3k151_02BP-110-004.1.fid
Comment 3k151_02BP-110-004 1H
Origin Bruker BioSpin GmbH
Owner nmrsu
Instrument Avance
Author Nicki
Solvent DMSO
Temperature 298.0
Pulse Sequence zg30
Experiment 1D
Probe Z154705_0043 (PI HR-BBO600S3-BBF/ H/ D-5.0-Z SP)
Number of Scans 64
Receiver Gain 101.0
Relaxation Delay 1.0000
Pulse Width 10.0000
Acquisition Time 2.7525
Acquisition Date 2021-08-31T05:09:57
Spectrometer Frequency 600.14
Spectral Width 11904.8
Lowest Frequency -2251.9
Nucleus 1H
Acquired Size 32768
Spectral Size 131072
Digital Resolution 0.09
H N NH2 HN N N O O
22
1.05 0.96 1.50 2.74 1.12 1.92 3.02 0.97 0.97 1.00 0.98 0.96 2.09 2.08
10.5 10.0 9.5 9.0 8.5 8.0 7.5 7.0 6.5 6.0 5.5 5.0 4.5 4.0 3.5 3.0 2.5 2.0 1.5 1.0 0.5 0.0
f1 (ppm)

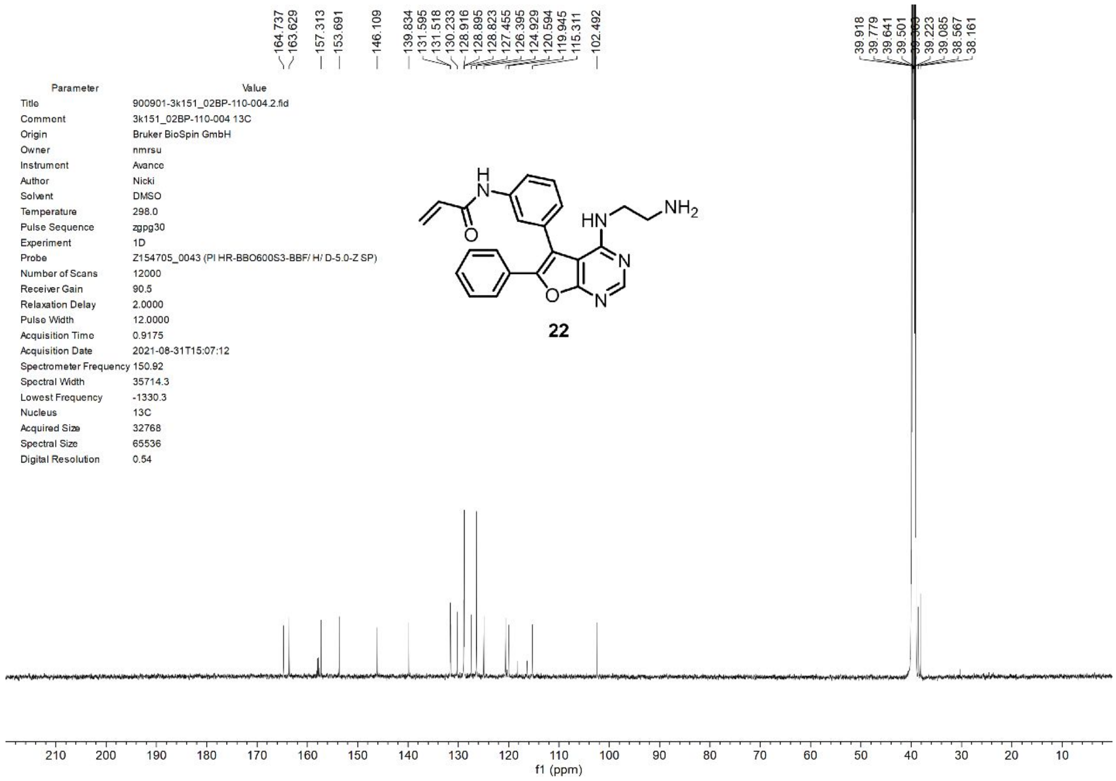
164.737 163.629 157.313 153.691 146.109 139.834 131.595 131.518 130.233 128.916 128.895 128.823 127.455 126.395 124.929 120.594 119.945 115.311 102.492
39.918 39.779 39.641 39.501 39.223 39.085 38.567 38.161
Parameter Value
Title 900901-3k151_02BP-110-004.2.fid
Comment 3k151_02BP-110-004 13C
Origin Bruker BioSpin GmbH
Owner nmrsu
Instrument Avance
Author Nicki
Solvent DMSO
Temperature 298.0
Pulse Sequence zgpg30
Experiment 1D
Probe Z154705_0043 (PI HR-BBO600S3-BBF/ H/ D-5.0-Z SP)
Number of Scans 12000
Receiver Gain 90.5
Relaxation Delay 2.0000
Pulse Width 12.0000
Acquisition Time 0.9175
Acquisition Date 2021-08-31T15:07:12
Spectrometer Frequency 150.92
Spectral Width 35714.3
Lowest Frequency -1330.3
Nucleus 13C
Acquired Size 32768
Spectral Size 65536
Digital Resolution 0.54
H N NH2 HN N N O O
22
210 200 190 180 170 160 150 140 130 120 110 100 90 80 70 60 50 40 30 20 10
f1 (ppm)

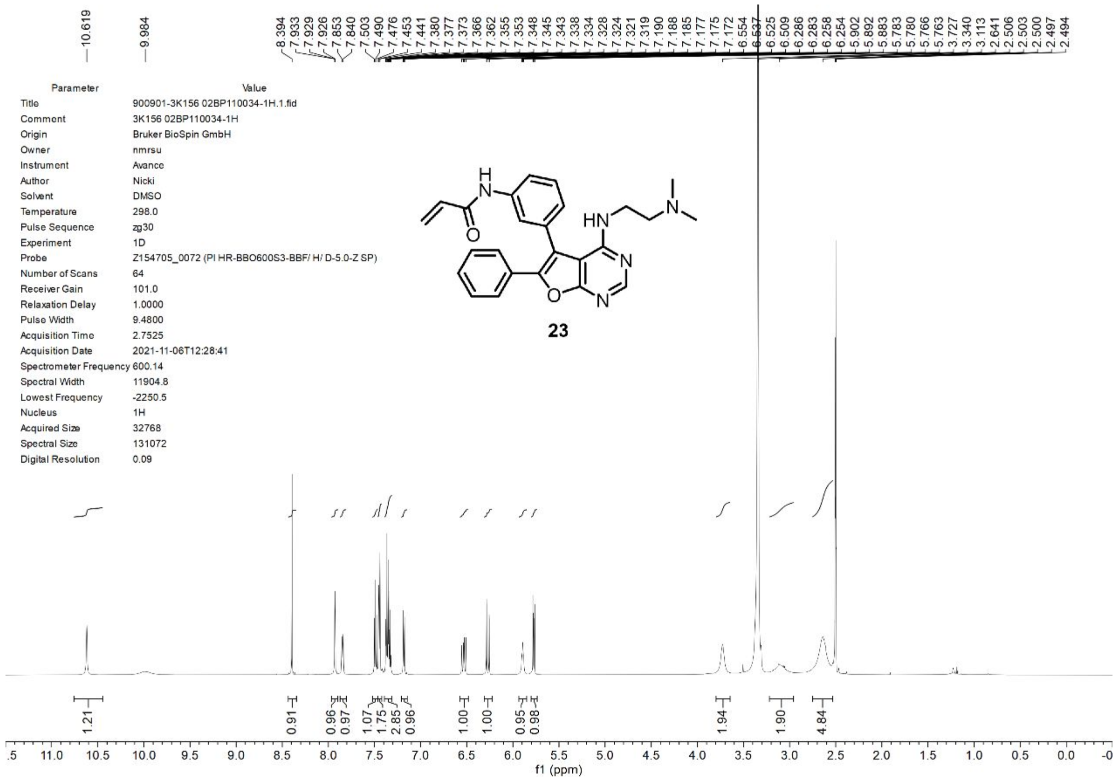
10.619
9.984
8.394
7.933
7.929
7.926
7.853
7.840
7.503
7.490
7.476
7.453
7.441
7.380
7.377
7.373
7.366
7.362
7.355
7.353
7.348
7.345
7.343
7.338
7.334
7.328
7.324
7.321
7.319
7.190
7.188
7.185
7.177
7.175
7.172
6.554
6.537
6.525
6.509
6.286
6.283
6.258
6.254
5.902
5.892
5.883
5.780
5.766
5.763
3.727
3.340
3.113
2.641
2.506
2.503
2.500
2.497
2.494
Parameter Value
Title 900901-3K156 02BP110034-1H.1.fid
Comment 3K156 02BP110034-1H
Origin Bruker BioSpin GmbH
Owner nmrsu
Instrument Avance
Author Nicki
Solvent DMSO
Temperature 298.0
Pulse Sequence zg30
Experiment 1D
Probe Z154705_0072 (PI HR-BBO600S3-BBF/ H/ D-5.0-Z SP)
Number of Scans 64
Receiver Gain 101.0
Relaxation Delay 1.0000
Pulse Width 9.4800
Acquisition Time 2.7525
Acquisition Date 2021-11-06T12:28:41
Spectrometer Frequency 600.14
Spectral Width 11904.8
Lowest Frequency -2250.5
Nucleus 1H
Acquired Size 32768
Spectral Size 131072
Digital Resolution 0.09
23
1.21
0.91
0.96
0.97
1.07
1.75
2.85
0.96
1.00
1.00
0.95
0.98
1.94
1.90
4.84
f1 (ppm)

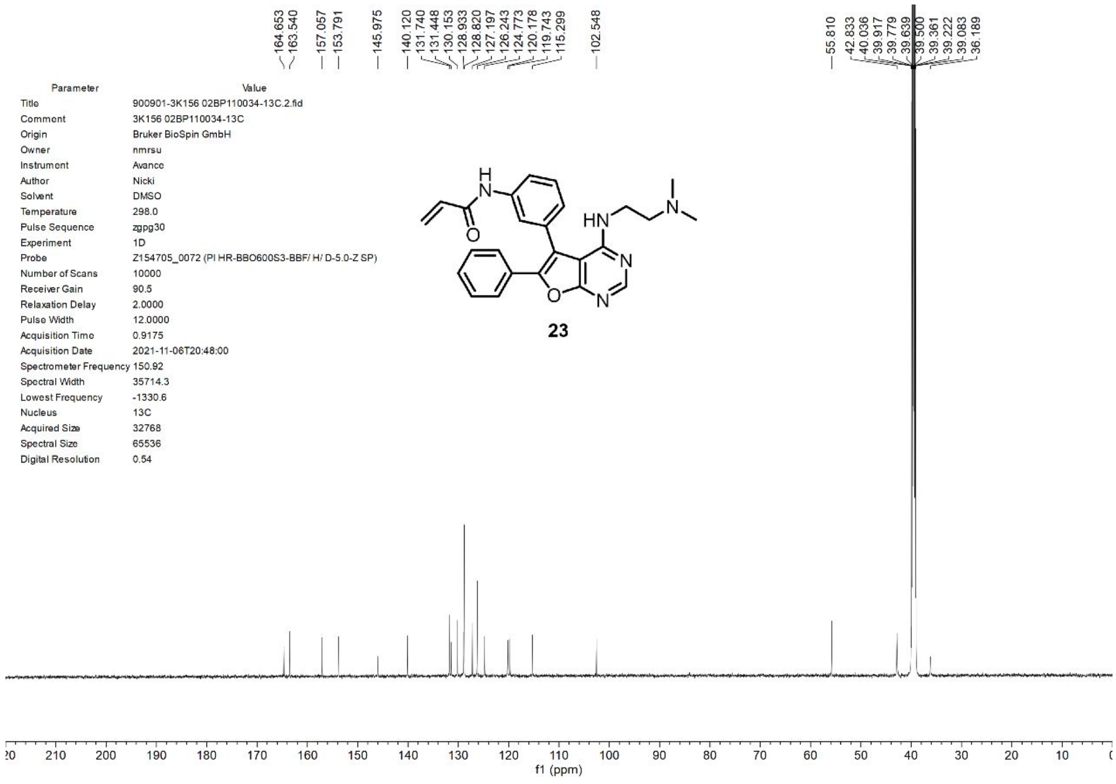
164.653
163.540
157.057
153.791
145.975
140.120
131.740
131.448
130.153
128.933
128.820
127.197
126.243
124.773
120.178
119.743
115.299
102.548
55.810
42.833
40.036
39.917
39.779
39.639
39.500
39.361
39.222
39.083
36.189
Parameter Value
Title 900901-3K156 02BP110034-13C.2.fid
Comment 3K156 02BP110034-13C
Origin Bruker BioSpin GmbH
Owner nmrsu
Instrument Avance
Author Nicki
Solvent DMSO
Temperature 298.0
Pulse Sequence zgpg30
Experiment 1D
Probe Z154705_0072 (PI HR-BBO600S3-BBF/ H/ D-5.0-Z SP)
Number of Scans 10000
Receiver Gain 90.5
Relaxation Delay 2.0000
Pulse Width 12.0000
Acquisition Time 0.9175
Acquisition Date 2021-11-06T20:48:00
Spectrometer Frequency 150.92
Spectral Width 35714.3
Lowest Frequency -1330.6
Nucleus 13C
Acquired Size 32768
Spectral Size 65536
Digital Resolution 0.54
23
f1 (ppm)

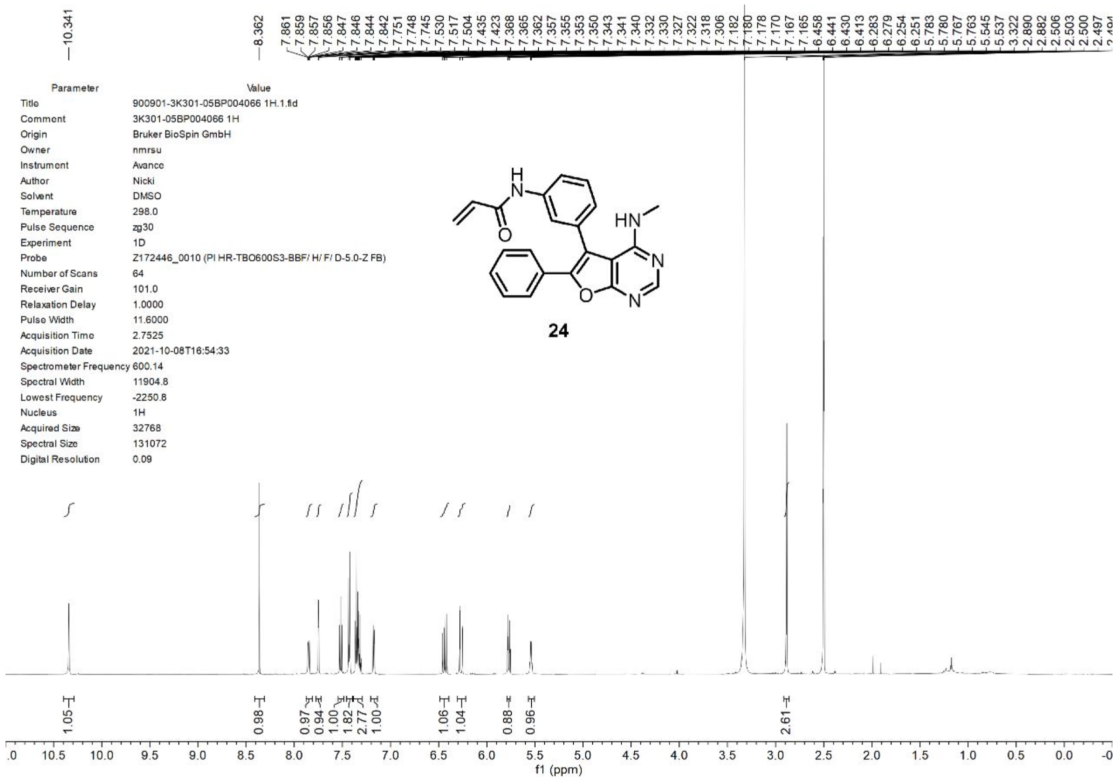
10.341
8.362
7.861 7.859 7.857 7.856 7.847 7.846 7.844 7.842 7.751 7.748 7.745 7.530 7.517 7.504 7.435 7.423 7.368 7.365 7.362 7.357 7.355 7.353 7.350 7.343 7.341 7.340 7.332 7.330 7.327 7.322 7.318 7.306 7.182 7.180 7.178 7.170 7.167 7.165 6.458 6.441 6.430 6.413 6.283 6.279 6.254 6.251 5.783 5.780 5.767 5.763 5.545 5.537 3.322 2.890 2.882 2.506 2.503 2.500 2.497
Parameter Value
Title 900901-3K301-05BP004066 1H.1.fid
Comment 3K301-05BP004066 1H
Origin Bruker BioSpin GmbH
Owner nmrsu
Instrument Avance
Author Nicki
Solvent DMSO
Temperature 298.0
Pulse Sequence zg30
Experiment 1D
Probe Z172446_0010 (PI HR-TBO600S3-BBF/ H/ F/ D-5.0-Z FB)
Number of Scans 64
Receiver Gain 101.0
Relaxation Delay 1.0000
Pulse Width 11.6000
Acquisition Time 2.7525
Acquisition Date 2021-10-08T16:54:33
Spectrometer Frequency 600.14
Spectral Width 11904.8
Lowest Frequency -2250.8
Nucleus 1H
Acquired Size 32768
Spectral Size 131072
Digital Resolution 0.09
HN
N
N
O
H
N
O
24
1.05 0.98 0.97 0.94 1.00 1.82 2.77 1.00 1.06 1.04 0.88 0.96 2.61
10.5 10.0 9.5 9.0 8.5 8.0 7.5 7.0 6.5 6.0 5.5 5.0 4.5 4.0 3.5 3.0 2.5 2.0 1.5 1.0 0.5 0.0
f1 (ppm)

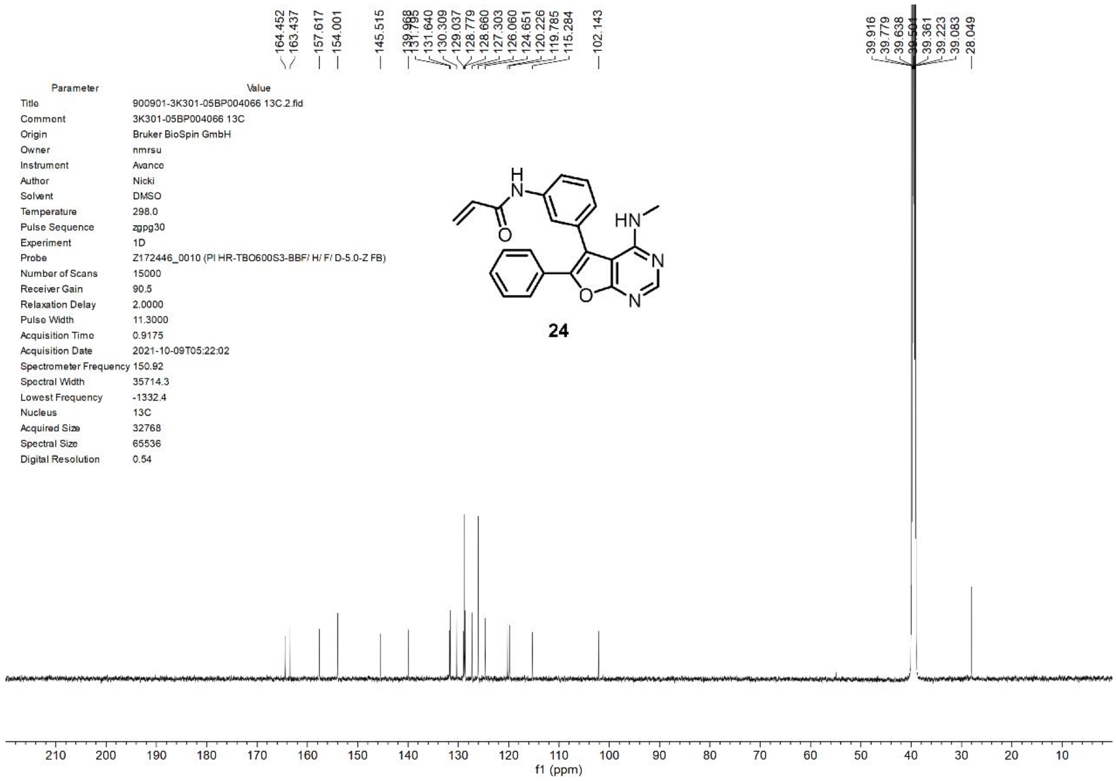
164.452 163.437 157.617 154.001 145.515 139.968 131.795 131.640 130.309 129.037 128.779 128.660 127.303 126.060 124.651 120.226 119.785 115.284 102.143
39.916 39.779 39.638 39.501 39.361 39.223 39.083 28.049
Parameter Value
Title 900901-3K301-05BP004066 13C.2.fid
Comment 3K301-05BP004066 13C
Origin Bruker BioSpin GmbH
Owner nmrsu
Instrument Avance
Author Nicki
Solvent DMSO
Temperature 298.0
Pulse Sequence zgpg30
Experiment 1D
Probe Z172446_0010 (PI HR-TBO600S3-BBF/ H/ F/ D-5.0-Z FB)
Number of Scans 15000
Receiver Gain 90.5
Relaxation Delay 2.0000
Pulse Width 11.3000
Acquisition Time 0.9175
Acquisition Date 2021-10-09T05:22:02
Spectrometer Frequency 150.92
Spectral Width 35714.3
Lowest Frequency -1332.4
Nucleus 13C
Acquired Size 32768
Spectral Size 65536
Digital Resolution 0.54
HN
N
N
O
H
N
O
24
210 200 190 180 170 160 150 140 130 120 110 100 90 80 70 60 50 40 30 20 10
f1 (ppm)

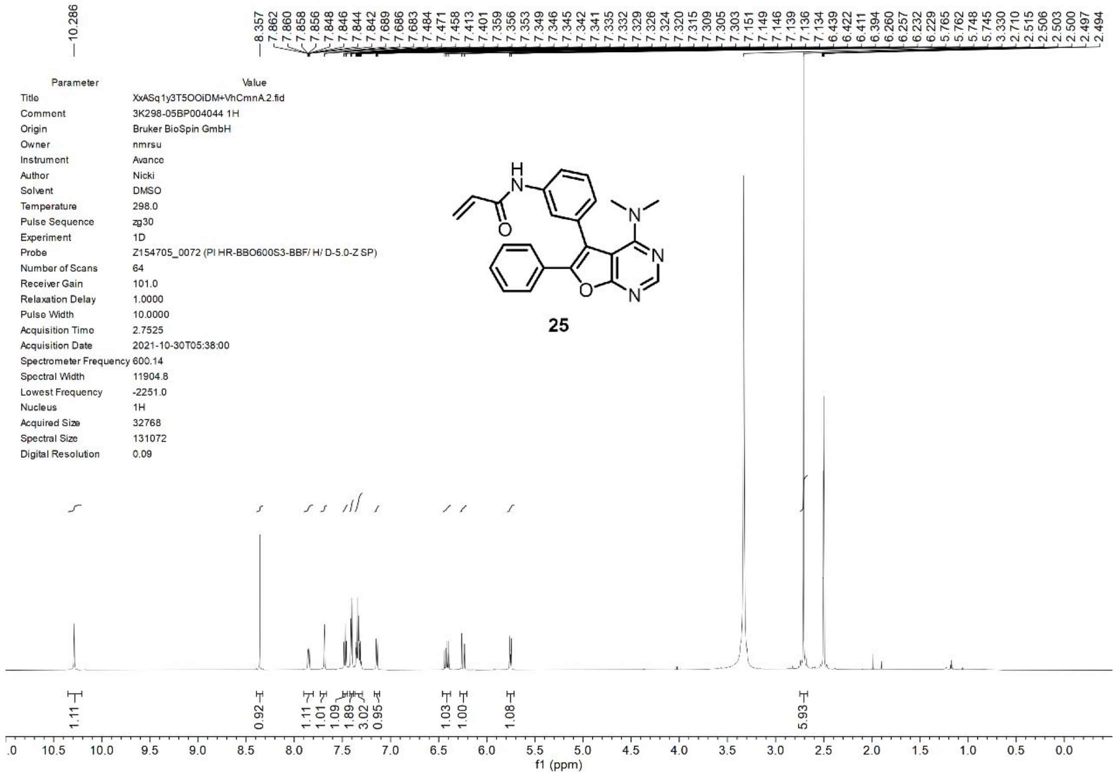
10.286
8.357 7.862 7.860 7.858 7.856 7.848 7.846 7.844 7.842 7.689 7.686 7.683 7.484 7.471 7.458 7.413 7.401 7.359 7.356 7.353 7.349 7.346 7.345 7.342 7.341 7.335 7.332 7.329 7.326 7.324 7.320 7.315 7.309 7.305 7.303 7.151 7.149 7.146 7.139 7.136 7.134 6.439 6.422 6.411 6.394 6.260 6.257 6.232 6.229 5.765 5.762 5.748 5.745 3.330 2.710 2.515 2.506 2.503 2.500 2.497 2.494
Parameter Value
Title XxASq1y3T5OOiDM+VhCmnA.2.fid
Comment 3K298-05BP004044 1H
Origin Bruker BioSpin GmbH
Owner nmrsu
Instrument Avance
Author Nicki
Solvent DMSO
Temperature 298.0
Pulse Sequence zg30
Experiment 1D
Probe Z154705_0072 (PI HR-BBO600S3-BBF/ H/ D-5.0-Z SP)
Number of Scans 64
Receiver Gain 101.0
Relaxation Delay 1.0000
Pulse Width 10.0000
Acquisition Time 2.7525
Acquisition Date 2021-10-30T05:38:00
Spectrometer Frequency 600.14
Spectral Width 11904.8
Lowest Frequency -2251.0
Nucleus 1H
Acquired Size 32768
Spectral Size 131072
Digital Resolution 0.09
25
1.11 0.92 1.11 1.01 1.09 1.89 3.02 0.95 1.03 1.00 1.08 5.93
f1 (ppm)

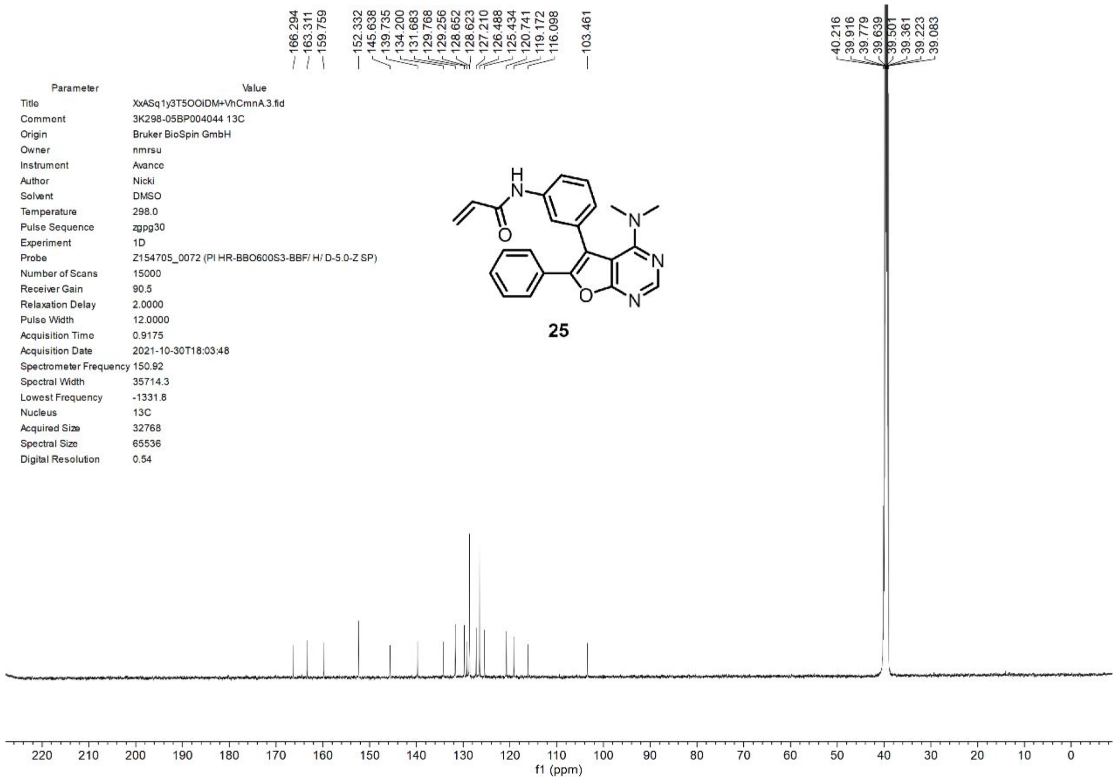
166.294 163.311 159.759 152.332 145.638 139.735 134.200 131.683 129.768 129.256 128.652 128.623 127.210 126.488 125.434 120.741 119.172 116.098 103.461
40.216 39.916 39.779 39.639 39.501 39.361 39.223 39.083
Parameter Value
Title XxASq1y3T5OOiDM+VhCmnA.3.fid
Comment 3K298-05BP004044 13C
Origin Bruker BioSpin GmbH
Owner nmrsu
Instrument Avance
Author Nicki
Solvent DMSO
Temperature 298.0
Pulse Sequence zgpg30
Experiment 1D
Probe Z154705_0072 (PI HR-BBO600S3-BBF/ H/ D-5.0-Z SP)
Number of Scans 15000
Receiver Gain 90.5
Relaxation Delay 2.0000
Pulse Width 12.0000
Acquisition Time 0.9175
Acquisition Date 2021-10-30T18:03:48
Spectrometer Frequency 150.92
Spectral Width 35714.3
Lowest Frequency -1331.8
Nucleus 13C
Acquired Size 32768
Spectral Size 65536
Digital Resolution 0.54
25
f1 (ppm)

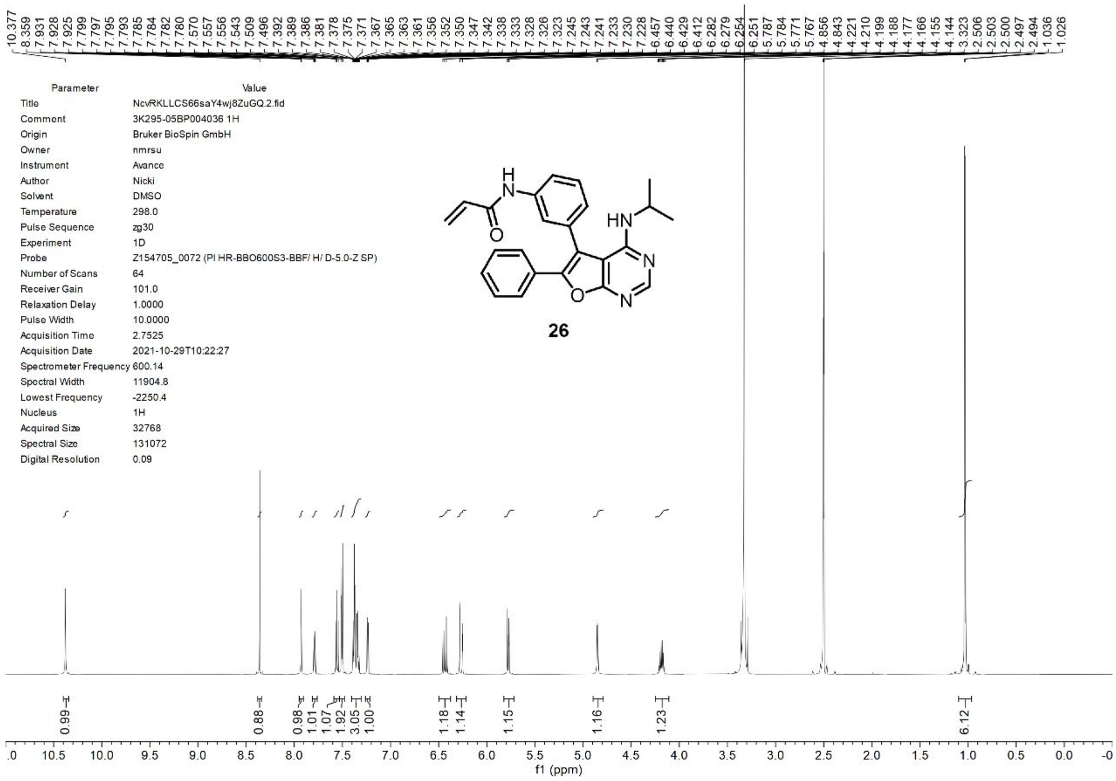
10.377
8.359
7.931
7.928
7.925
7.799
7.797
7.795
7.793
7.785
7.784
7.782
7.780
7.570
7.557
7.556
7.543
7.509
7.496
7.392
7.389
7.386
7.381
7.378
7.375
7.371
7.367
7.365
7.363
7.361
7.356
7.352
7.350
7.347
7.342
7.338
7.333
7.328
7.326
7.323
7.245
7.243
7.241
7.233
7.230
7.228
6.457
6.440
6.429
6.412
6.282
6.279
6.254
6.251
5.787
5.784
5.771
5.767
4.856
4.843
4.221
4.210
4.199
4.188
4.177
4.166
4.155
4.144
3.323
2.506
2.503
2.500
2.497
2.494
1.036
1.026
Parameter Value
Title NcvRKLLCS66saY4wj8ZuGQ.2.fid
Comment 3K295-05BP004036 1H
Origin Bruker BioSpin GmbH
Owner nmrsu
Instrument Avance
Author Nicki
Solvent DMSO
Temperature 298.0
Pulse Sequence zg30
Experiment 1D
Probe Z154705_0072 (PI HR-BBO600S3-BBF/ H/ D-5.0-Z SP)
Number of Scans 64
Receiver Gain 101.0
Relaxation Delay 1.0000
Pulse Width 10.0000
Acquisition Time 2.7525
Acquisition Date 2021-10-29T10:22:27
Spectrometer Frequency 600.14
Spectral Width 11904.8
Lowest Frequency -2250.4
Nucleus 1H
Acquired Size 32768
Spectral Size 131072
Digital Resolution 0.09
H
N
O
HN
N
N
O
26
0.99
0.88
0.98
1.01
1.07
1.92
3.05
1.00
1.18
1.14
1.15
1.16
1.23
6.12
10.5
10.0
9.5
9.0
8.5
8.0
7.5
7.0
6.5
6.0
5.5
5.0
4.5
4.0
3.5
3.0
2.5
2.0
1.5
1.0
0.5
0.0
f1 (ppm)

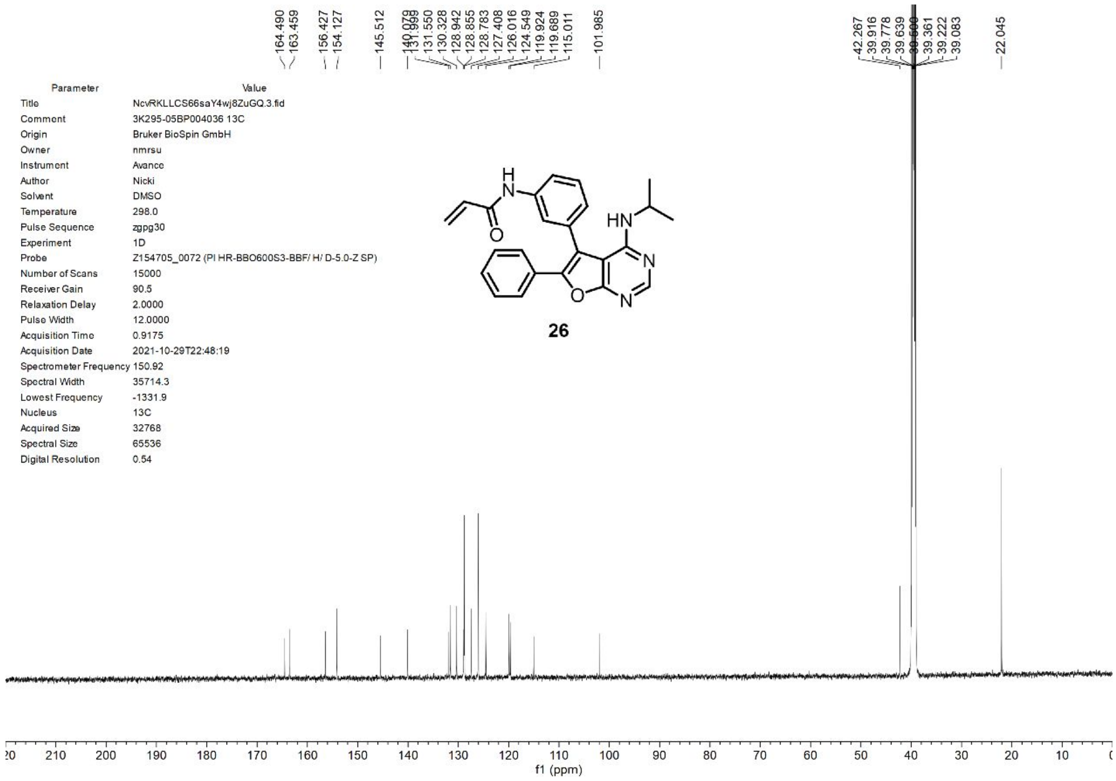
164.490
163.459
156.427
154.127
145.512
131.550
130.328
128.942
128.855
128.783
127.408
126.016
124.549
119.924
119.689
115.011
101.985
42.267
39.916
39.778
39.639
39.361
39.222
39.083
22.045
Parameter Value
Title NcvRKLLCS66saY4wj8ZuGQ.3.fid
Comment 3K295-05BP004036 13C
Origin Bruker BioSpin GmbH
Owner nmrsu
Instrument Avance
Author Nicki
Solvent DMSO
Temperature 298.0
Pulse Sequence zgpg30
Experiment 1D
Probe Z154705_0072 (PI HR-BBO600S3-BBF/ H/ D-5.0-Z SP)
Number of Scans 15000
Receiver Gain 90.5
Relaxation Delay 2.0000
Pulse Width 12.0000
Acquisition Time 0.9175
Acquisition Date 2021-10-29T22:48:19
Spectrometer Frequency 150.92
Spectral Width 35714.3
Lowest Frequency -1331.9
Nucleus 13C
Acquired Size 32768
Spectral Size 65536
Digital Resolution 0.54
H
N
O
HN
N
N
O
26
210
200
190
180
170
160
150
140
130
120
110
100
90
80
70
60
50
40
30
20
10
f1 (ppm)

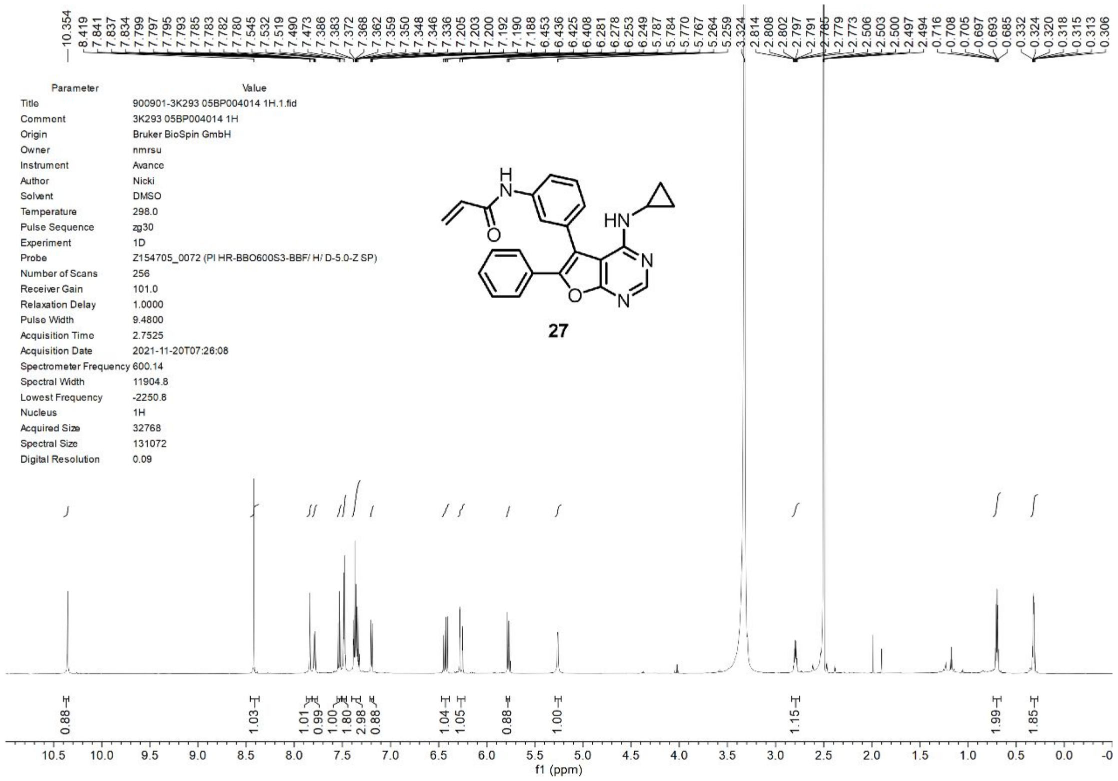
Parameter Value
Title 900901-3K293 05BP004014 1H.1.fid
Comment 3K293 05BP004014 1H
Origin Bruker BioSpin GmbH
Owner nmrsu
Instrument Avance
Author Nicki
Solvent DMSO
Temperature 298.0
Pulse Sequence zg30
Experiment 1D
Probe Z154705_0072 (PI HR-BBO600S3-BBF/ H/ D-5.0-Z SP)
Number of Scans 256
Receiver Gain 101.0
Relaxation Delay 1.0000
Pulse Width 9.4800
Acquisition Time 2.7525
Acquisition Date 2021-11-20T07:26:08
Spectrometer Frequency 600.14
Spectral Width 11904.8
Lowest Frequency -2250.8
Nucleus 1H
Acquired Size 32768
Spectral Size 131072
Digital Resolution 0.09
27
f1 (ppm)

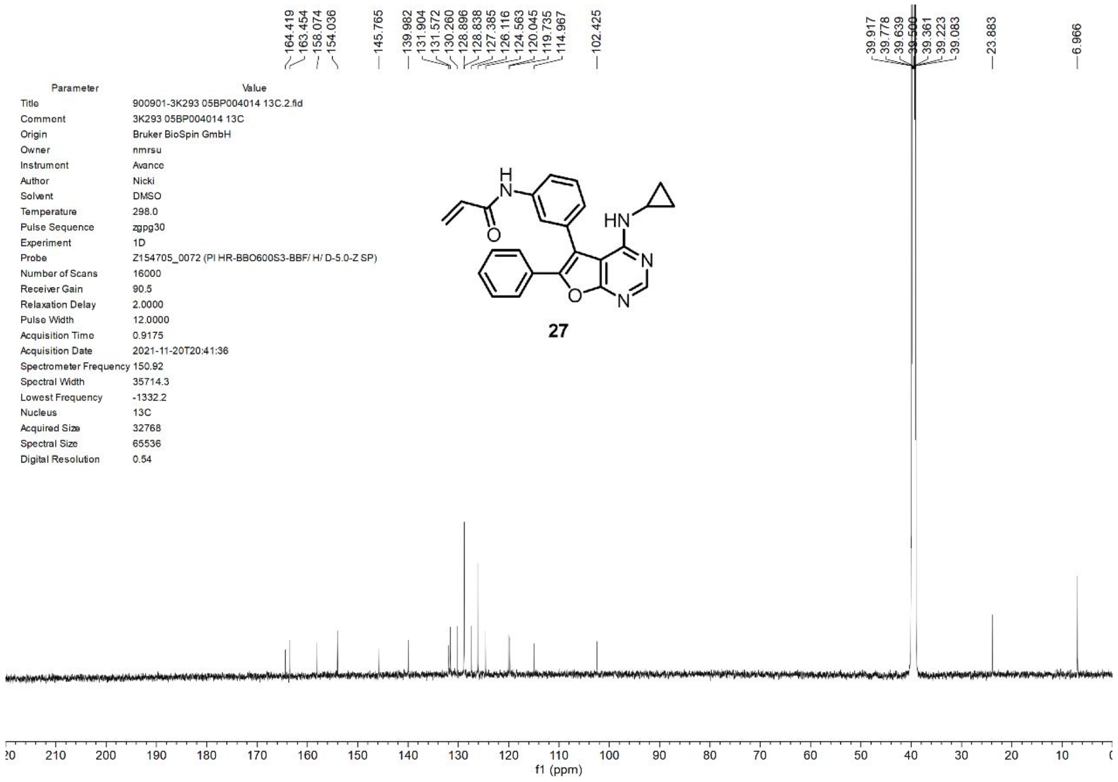
Parameter Value
Title 900901-3K293 05BP004014 13C.2.fid
Comment 3K293 05BP004014 13C
Origin Bruker BioSpin GmbH
Owner nmrsu
Instrument Avance
Author Nicki
Solvent DMSO
Temperature 298.0
Pulse Sequence zgpg30
Experiment 1D
Probe Z154705_0072 (PI HR-BBO600S3-BBF/ H/ D-5.0-Z SP)
Number of Scans 16000
Receiver Gain 90.5
Relaxation Delay 2.0000
Pulse Width 12.0000
Acquisition Time 0.9175
Acquisition Date 2021-11-20T20:41:36
Spectrometer Frequency 150.92
Spectral Width 35714.3
Lowest Frequency -1332.2
Nucleus 13C
Acquired Size 32768
Spectral Size 65536
Digital Resolution 0.54
27
f1 (ppm)

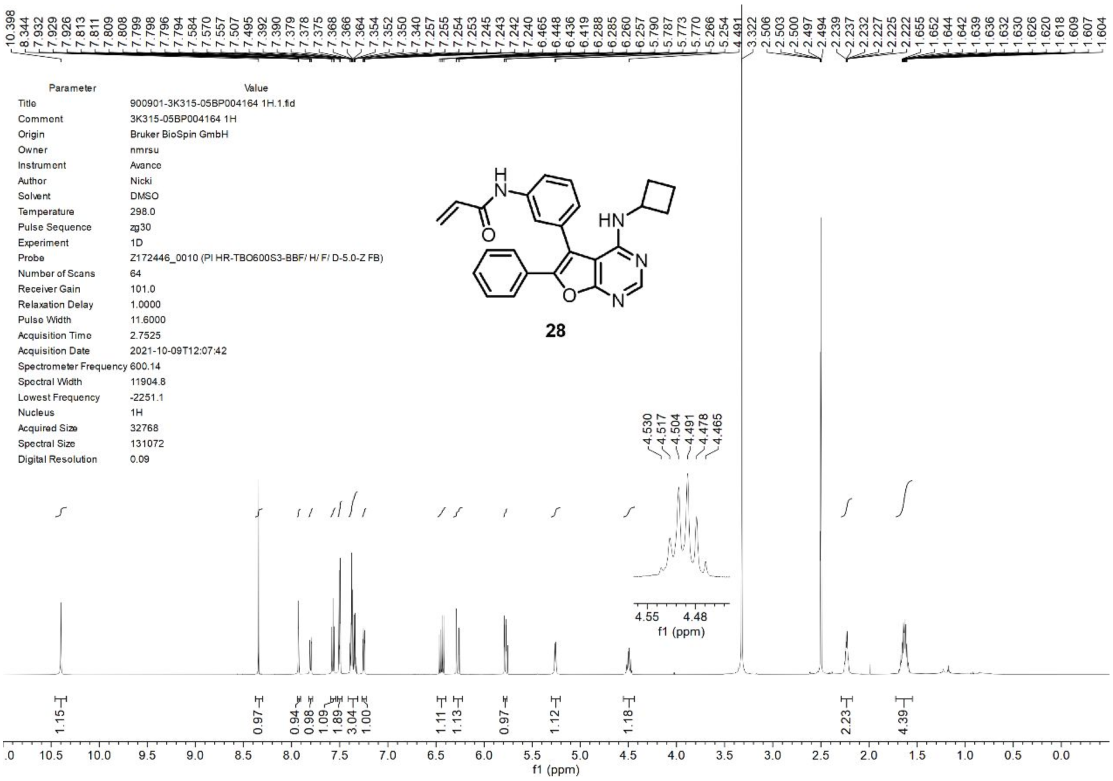

Parameter	Value
Title	900901-3K315-05BP004164 1H.1.fid
Comment	3K315-05BP004164 1H
Origin	Bruker BioSpin GmbH
Owner	nmrsu
Instrument	Avance
Author	Nicki
Solvent	DMSO
Temperature	298.0
Pulse Sequence	zg30
Experiment	1D
Probe	Z172446_0010 (PI HR-TBO600S3-BBF/ H/ F/ D-5.0-Z FB)
Number of Scans	64
Receiver Gain	101.0
Relaxation Delay	1.0000
Pulse Width	11.6000
Acquisition Time	2.7525
Acquisition Date	2021-10-09T12:07:42
Spectrometer Frequency	600.14
Spectral Width	11904.8
Lowest Frequency	-2251.1
Nucleus	1H
Acquired Size	32768
Spectral Size	131072
Digital Resolution	0.09

28

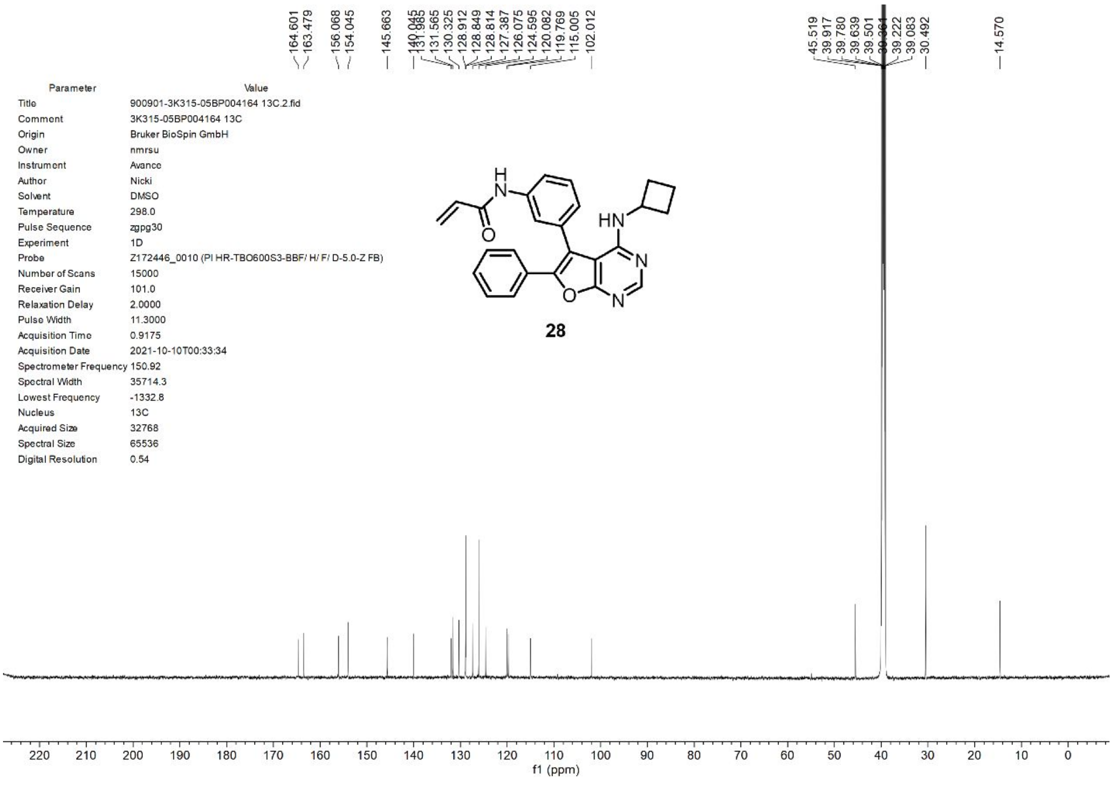

Parameter	Value
Title	900901-3K315-05BP004164 13C.2.fid
Comment	3K315-05BP004164 13C
Origin	Bruker BioSpin GmbH
Owner	nmrsu
Instrument	Avance
Author	Nicki
Solvent	DMSO
Temperature	298.0
Pulse Sequence	zgpg30
Experiment	1D
Probe	Z172446_0010 (PI HR-TBO600S3-BBF/ H/ F/ D-5.0-Z FB)
Number of Scans	15000
Receiver Gain	101.0
Relaxation Delay	2.0000
Pulse Width	11.3000
Acquisition Time	0.9175
Acquisition Date	2021-10-10T00:33:34
Spectrometer Frequency	150.92
Spectral Width	35714.3
Lowest Frequency	-1332.8
Nucleus	13C
Acquired Size	32768
Spectral Size	65536
Digital Resolution	0.54

28

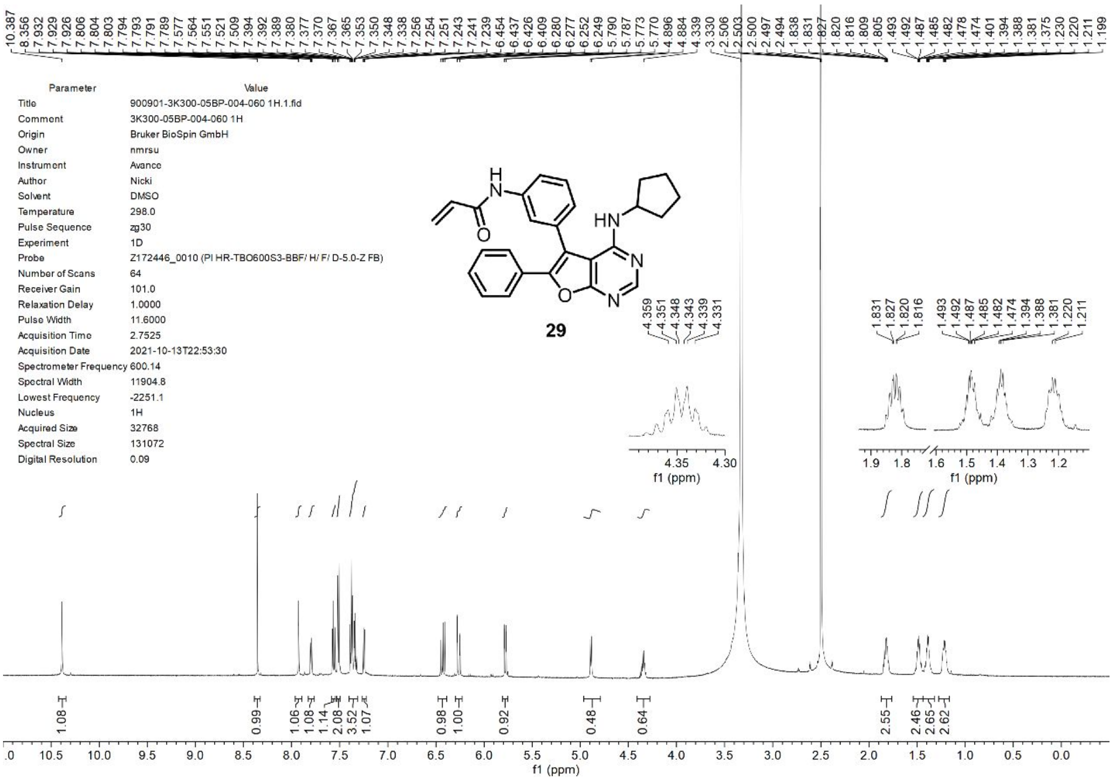
10.387
8.356
7.932
7.929
7.926
7.806
7.804
7.803
7.794
7.793
7.791
7.789
7.577
7.564
7.551
7.521
7.509
7.394
7.392
7.389
7.380
7.377
7.370
7.367
7.365
7.353
7.350
7.348
7.338
7.256
7.254
7.251
7.243
7.241
7.239
6.454
6.437
6.426
6.409
6.280
6.277
6.252
6.249
5.790
5.787
5.773
5.770
4.896
4.884
4.339
3.330
2.506
2.503
2.500
2.497
2.494
1.838
1.831
1.827
1.820
1.816
1.809
1.805
1.493
1.492
1.487
1.485
1.482
1.478
1.474
1.401
1.394
1.388
1.381
1.375
1.230
1.220
1.211
1.199
Parameter Value
Title 900901-3K300-05BP-004-060 1H.1.fid
Comment 3K300-05BP-004-060 1H
Origin Bruker BioSpin GmbH
Owner nmrsu
Instrument Avance
Author Nicki
Solvent DMSO
Temperature 298.0
Pulse Sequence zg30
Experiment 1D
Probe Z172446_0010 (PI HR-TBO600S3-BBF/ H/ F/ D-5.0-Z FB)
Number of Scans 64
Receiver Gain 101.0
Relaxation Delay 1.0000
Pulse Width 11.6000
Acquisition Time 2.7525
Acquisition Date 2021-10-13T22:53:30
Spectrometer Frequency 600.14
Spectral Width 11904.8
Lowest Frequency -2251.1
Nucleus 1H
Acquired Size 32768
Spectral Size 131072
Digital Resolution 0.09
29
4.359
4.351
4.348
4.343
4.339
4.331
1.831
1.827
1.820
1.816
1.493
1.492
1.487
1.485
1.482
1.474
1.394
1.388
1.381
1.220
1.211
f1 (ppm)
1.08
0.99
1.06
1.08
1.14
2.08
3.52
1.07
0.98
1.00
0.92
0.48
0.64
2.55
2.46
2.65
2.62
f1 (ppm)

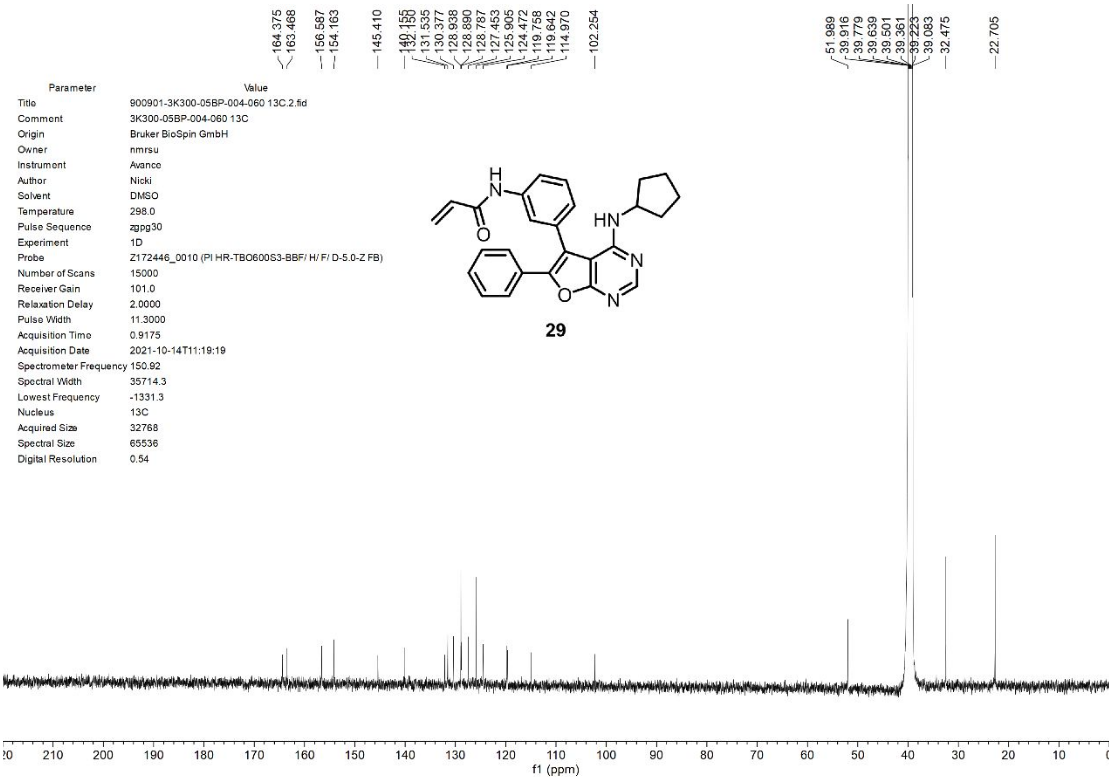
164.375
163.468
156.587
154.163
145.410
140.155
132.150
131.535
130.377
128.938
128.890
128.787
127.453
125.905
124.472
119.758
119.642
114.970
102.254
51.989
39.916
39.779
39.639
39.501
39.361
39.223
39.083
32.475
22.705
Parameter Value
Title 900901-3K300-05BP-004-060 13C.2.fid
Comment 3K300-05BP-004-060 13C
Origin Bruker BioSpin GmbH
Owner nmrsu
Instrument Avance
Author Nicki
Solvent DMSO
Temperature 298.0
Pulse Sequence zgpg30
Experiment 1D
Probe Z172446_0010 (PI HR-TBO600S3-BBF/ H/ F/ D-5.0-Z FB)
Number of Scans 15000
Receiver Gain 101.0
Relaxation Delay 2.0000
Pulse Width 11.3000
Acquisition Time 0.9175
Acquisition Date 2021-10-14T11:19:19
Spectrometer Frequency 150.92
Spectral Width 35714.3
Lowest Frequency -1331.3
Nucleus 13C
Acquired Size 32768
Spectral Size 65536
Digital Resolution 0.54
29
f1 (ppm)

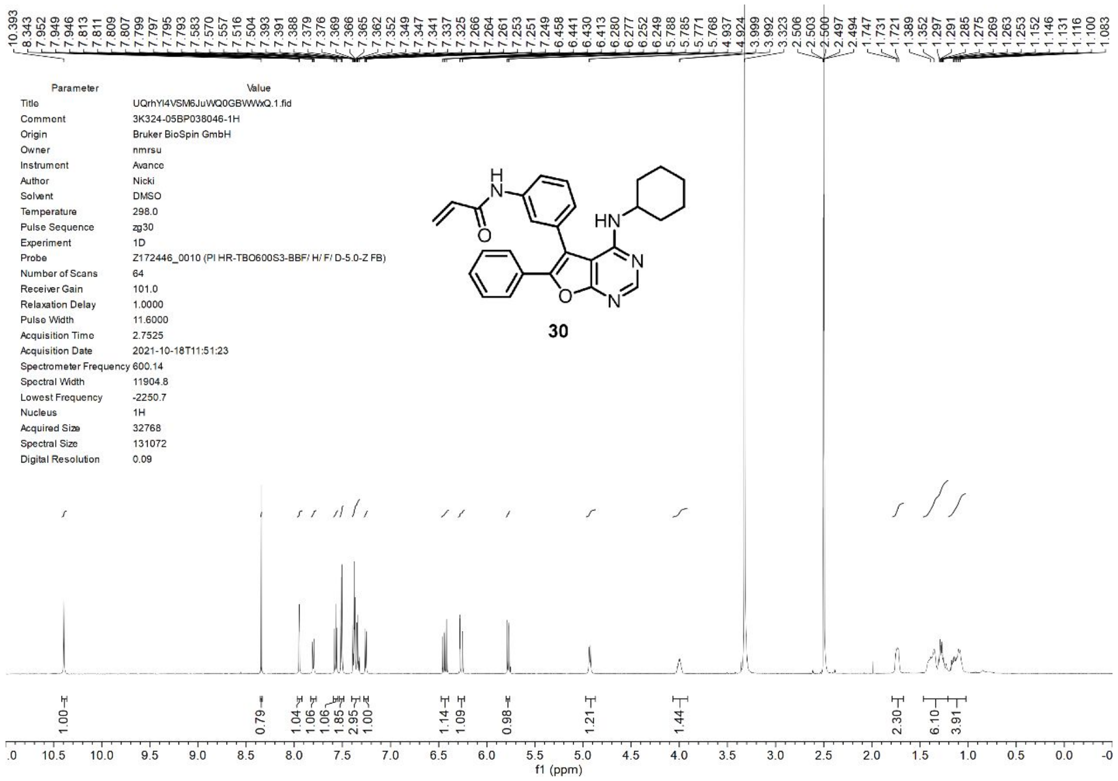
10.393
8.343
7.952
7.949
7.946
7.813
7.811
7.809
7.807
7.799
7.797
7.795
7.793
7.583
7.570
7.557
7.516
7.504
7.393
7.391
7.388
7.379
7.376
7.369
7.366
7.365
7.362
7.352
7.349
7.347
7.341
7.337
7.325
7.266
7.264
7.261
7.253
7.251
7.249
6.458
6.441
6.430
6.413
6.280
6.277
6.252
6.249
5.788
5.785
5.771
5.768
4.937
4.924
3.999
3.992
3.323
2.506
2.503
2.500
2.497
2.494
1.747
1.731
1.721
1.389
1.352
1.297
1.291
1.285
1.275
1.269
1.263
1.253
1.152
1.146
1.131
1.116
1.100
1.083
Parameter Value
Title UQrhYl4VSM6JuWQ0GBWWxQ.1.fid
Comment 3K324-05BP038046-1H
Origin Bruker BioSpin GmbH
Owner nmrsu
Instrument Avance
Author Nicki
Solvent DMSO
Temperature 298.0
Pulse Sequence zg30
Experiment 1D
Probe Z172446_0010 (PI HR-TBO600S3-BBF/ H/ F/ D-5.0-Z FB)
Number of Scans 64
Receiver Gain 101.0
Relaxation Delay 1.0000
Pulse Width 11.6000
Acquisition Time 2.7525
Acquisition Date 2021-10-18T11:51:23
Spectrometer Frequency 600.14
Spectral Width 11904.8
Lowest Frequency -2250.7
Nucleus 1H
Acquired Size 32768
Spectral Size 131072
Digital Resolution 0.09
HN
N
O
N
N
O
30
1.00
0.79
1.04
1.06
1.06
1.85
2.95
1.00
1.14
1.09
0.98
1.21
1.44
2.30
6.10
3.91
f1 (ppm)

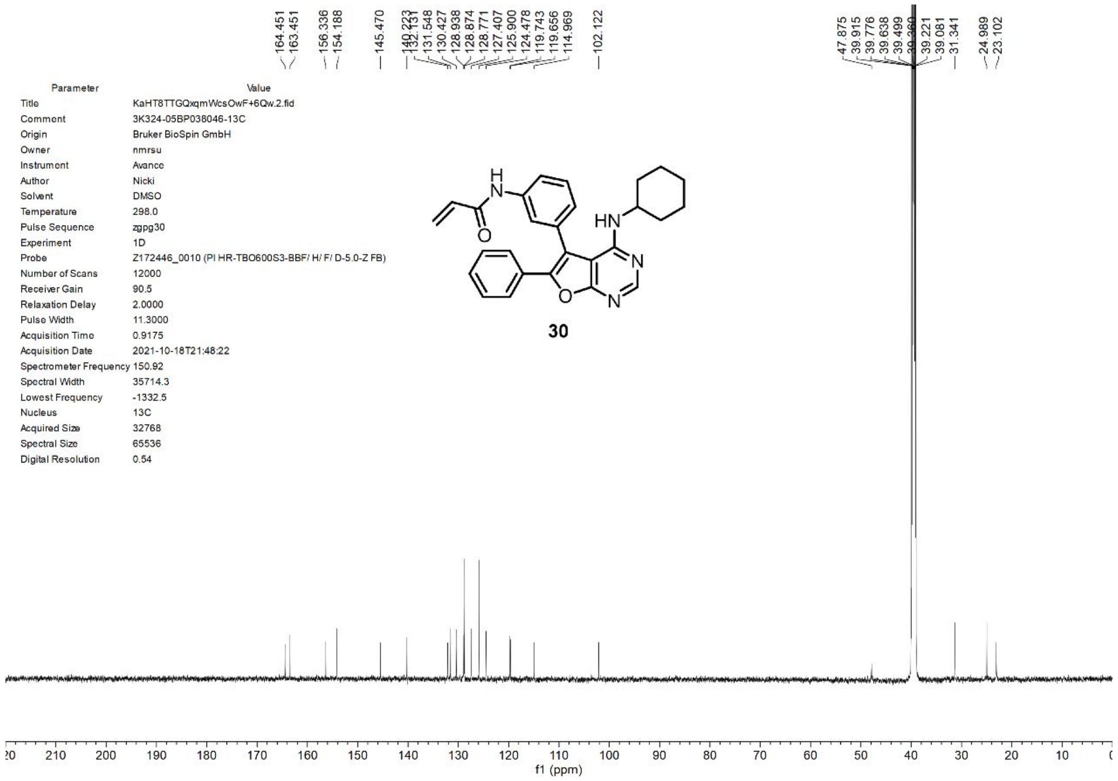
164.451
163.451
156.336
154.188
145.470
140.223
132.131
131.548
130.427
128.938
128.874
128.771
127.407
125.900
124.478
119.743
119.656
114.969
102.122
47.875
39.915
39.776
39.638
39.499
39.221
39.081
31.341
24.989
23.102
Parameter Value
Title KaHT8TTGQxqmWcsOwF+6Qw.2.fid
Comment 3K324-05BP038046-13C
Origin Bruker BioSpin GmbH
Owner nmrsu
Instrument Avance
Author Nicki
Solvent DMSO
Temperature 298.0
Pulse Sequence zgpg30
Experiment 1D
Probe Z172446_0010 (PI HR-TBO600S3-BBF/ H/ F/ D-5.0-Z FB)
Number of Scans 12000
Receiver Gain 90.5
Relaxation Delay 2.0000
Pulse Width 11.3000
Acquisition Time 0.9175
Acquisition Date 2021-10-18T21:48:22
Spectrometer Frequency 150.92
Spectral Width 35714.3
Lowest Frequency -1332.5
Nucleus 13C
Acquired Size 32768
Spectral Size 65536
Digital Resolution 0.54
HN
N
O
N
N
O
30
f1 (ppm)

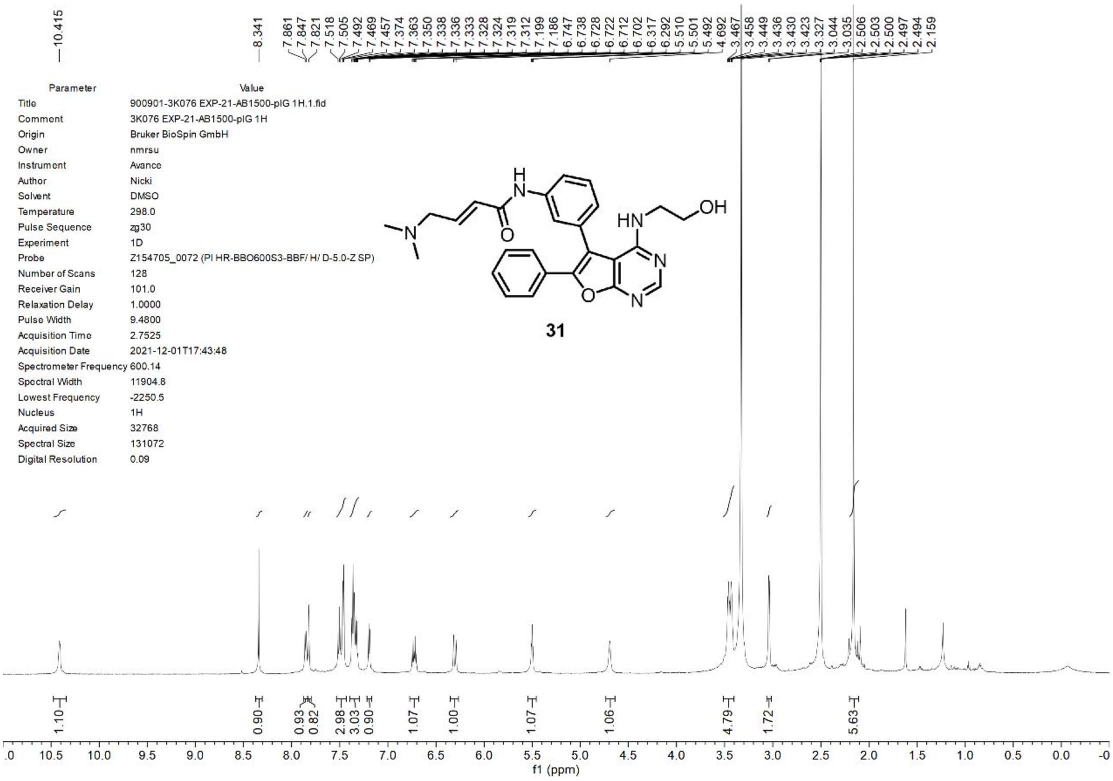
Parameter Value
Title 900901-3K076 EXP-21-AB1500-pIG 1H.1.fid
Comment 3K076 EXP-21-AB1500-pIG 1H
Origin Bruker BioSpin GmbH
Owner nmrsu
Instrument Avance
Author Nicki
Solvent DMSO
Temperature 298.0
Pulse Sequence zg30
Experiment 1D
Probe Z154705_0072 (PI HR-BBO600S3-BBF/ H/ D-5.0-Z SP)
Number of Scans 128
Receiver Gain 101.0
Relaxation Delay 1.0000
Pulse Width 9.4800
Acquisition Time 2.7525
Acquisition Date 2021-12-01T17:43:48
Spectrometer Frequency 600.14
Spectral Width 11904.8
Lowest Frequency -2250.5
Nucleus 1H
Acquired Size 32768
Spectral Size 131072
Digital Resolution 0.09
10.415
8.341
7.861 7.847 7.821 7.518 7.505 7.492 7.469 7.457 7.374 7.363 7.350 7.338 7.336 7.333 7.328 7.324 7.319 7.312 7.199 7.186 6.747 6.738 6.728 6.722 6.712 6.702 6.317 6.292 5.510 5.501 5.492 4.692 3.467 3.458 3.449 3.436 3.430 3.423 3.327 3.044 3.035 2.506 2.503 2.500 2.497 2.494 2.159
1.10 0.90 0.93 0.82 2.98 3.03 0.90 1.07 1.00 1.07 1.06 4.79 1.72 5.63
31
f1 (ppm)

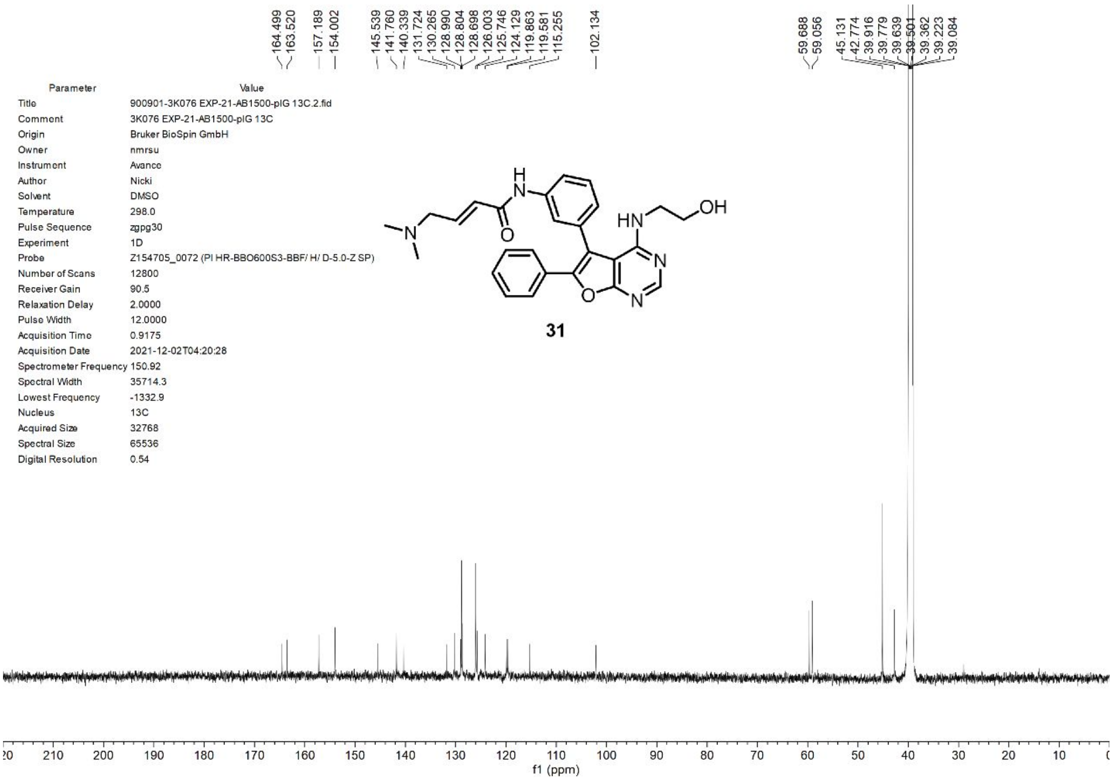
Parameter Value
Title 900901-3K076 EXP-21-AB1500-pIG 13C.2.fid
Comment 3K076 EXP-21-AB1500-pIG 13C
Origin Bruker BioSpin GmbH
Owner nmrsu
Instrument Avance
Author Nicki
Solvent DMSO
Temperature 298.0
Pulse Sequence zgpg30
Experiment 1D
Probe Z154705_0072 (PI HR-BBO600S3-BBF/ H/ D-5.0-Z SP)
Number of Scans 12800
Receiver Gain 90.5
Relaxation Delay 2.0000
Pulse Width 12.0000
Acquisition Time 0.9175
Acquisition Date 2021-12-02T04:20:28
Spectrometer Frequency 150.92
Spectral Width 35714.3
Lowest Frequency -1332.9
Nucleus 13C
Acquired Size 32768
Spectral Size 65536
Digital Resolution 0.54
164.499 163.520 157.189 154.002 145.539 141.760 140.339 131.724 130.265 128.990 128.804 128.698 126.003 125.746 124.129 119.863 119.581 115.255 102.134 59.688 59.056 45.131 42.774 39.916 39.779 39.639 39.501 39.362 39.223 39.084
31
f1 (ppm)

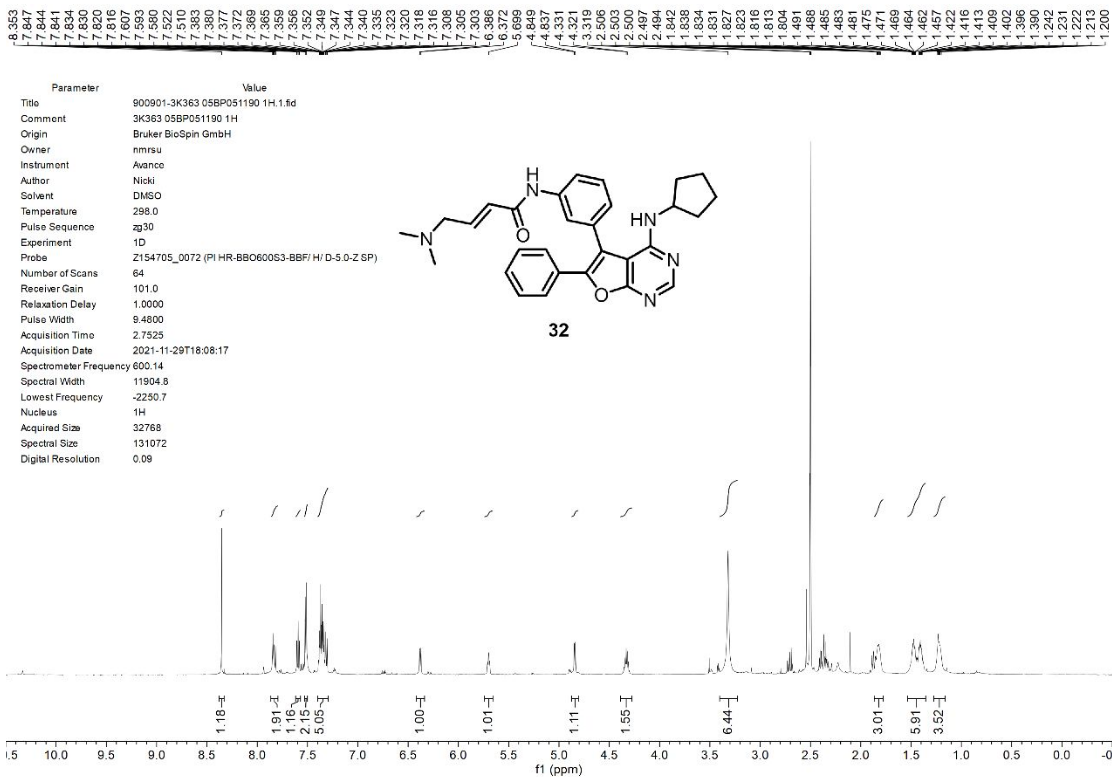
8.353 7.847 7.844 7.841 7.834 7.830 7.820 7.816 7.607 7.593 7.580 7.522 7.510 7.383 7.380 7.377 7.372 7.369 7.365 7.359 7.356 7.352 7.349 7.347 7.344 7.340 7.335 7.323 7.320 7.318 7.316 7.308 7.305 7.303 6.386 6.372 5.699 4.849 4.837 4.331 4.321 3.319 2.506 2.503 2.500 2.497 2.494 1.842 1.838 1.834 1.831 1.827 1.823 1.816 1.813 1.804 1.491 1.488 1.485 1.483 1.481 1.475 1.471 1.469 1.464 1.462 1.457 1.422 1.416 1.413 1.409 1.402 1.396 1.390 1.242 1.231 1.222 1.213 1.200
Parameter Value
Title 900901-3K363 05BP051190 1H.1.fid
Comment 3K363 05BP051190 1H
Origin Bruker BioSpin GmbH
Owner nmrsu
Instrument Avance
Author Nicki
Solvent DMSO
Temperature 298.0
Pulse Sequence zg30
Experiment 1D
Probe Z154705_0072 (PI HR-BBO600S3-BBF/ H/ D-5.0-Z SP)
Number of Scans 64
Receiver Gain 101.0
Relaxation Delay 1.0000
Pulse Width 9.4800
Acquisition Time 2.7525
Acquisition Date 2021-11-29T18:08:17
Spectrometer Frequency 600.14
Spectral Width 11904.8
Lowest Frequency -2250.7
Nucleus 1H
Acquired Size 32768
Spectral Size 131072
Digital Resolution 0.09
32
1.18 1.91 1.16 2.15 5.05 1.00 1.01 1.11 1.55 6.44 3.01 5.91 3.52
f1 (ppm)

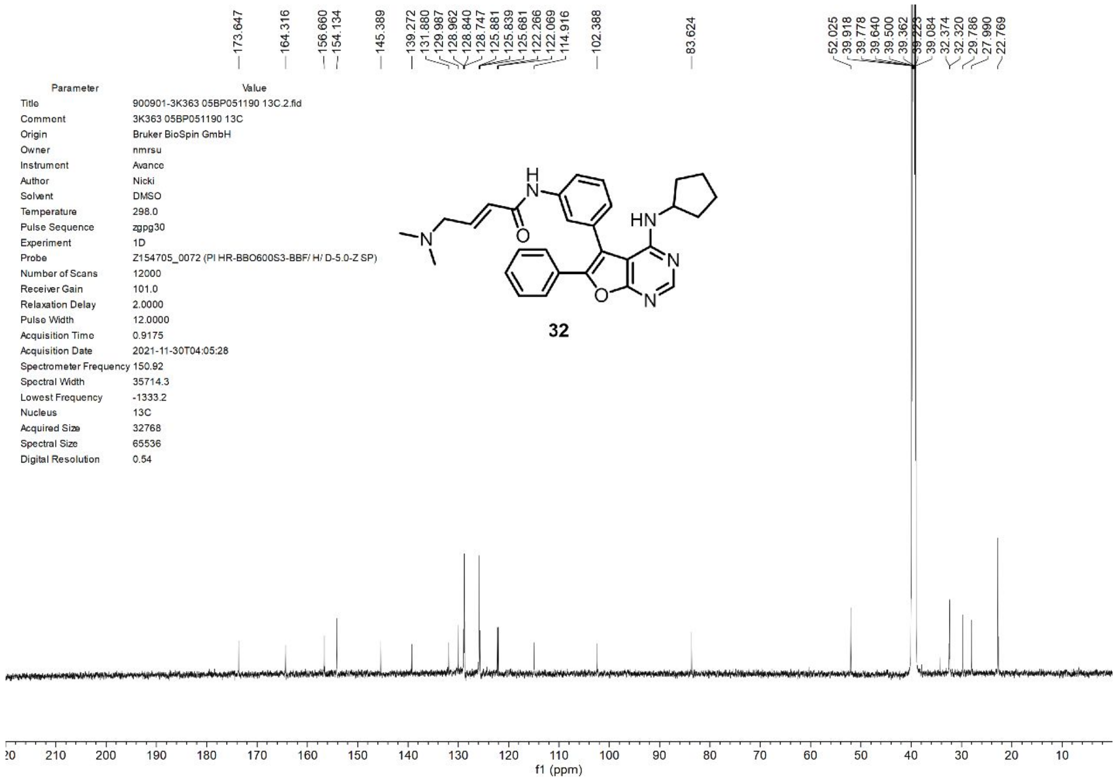
173.647 164.316 156.660 154.134 145.389 139.272 131.880 129.987 128.962 128.840 128.747 125.881 125.839 125.681 122.266 122.069 114.916 102.388 83.624 52.025 39.918 39.778 39.640 39.500 39.362 39.223 39.084 32.374 32.320 29.786 27.990 22.769
Parameter Value
Title 900901-3K363 05BP051190 13C.2.fid
Comment 3K363 05BP051190 13C
Origin Bruker BioSpin GmbH
Owner nmrsu
Instrument Avance
Author Nicki
Solvent DMSO
Temperature 298.0
Pulse Sequence zgpg30
Experiment 1D
Probe Z154705_0072 (PI HR-BBO600S3-BBF/ H/ D-5.0-Z SP)
Number of Scans 12000
Receiver Gain 101.0
Relaxation Delay 2.0000
Pulse Width 12.0000
Acquisition Time 0.9175
Acquisition Date 2021-11-30T04:05:28
Spectrometer Frequency 150.92
Spectral Width 35714.3
Lowest Frequency -1333.2
Nucleus 13C
Acquired Size 32768
Spectral Size 65536
Digital Resolution 0.54
32
f1 (ppm)

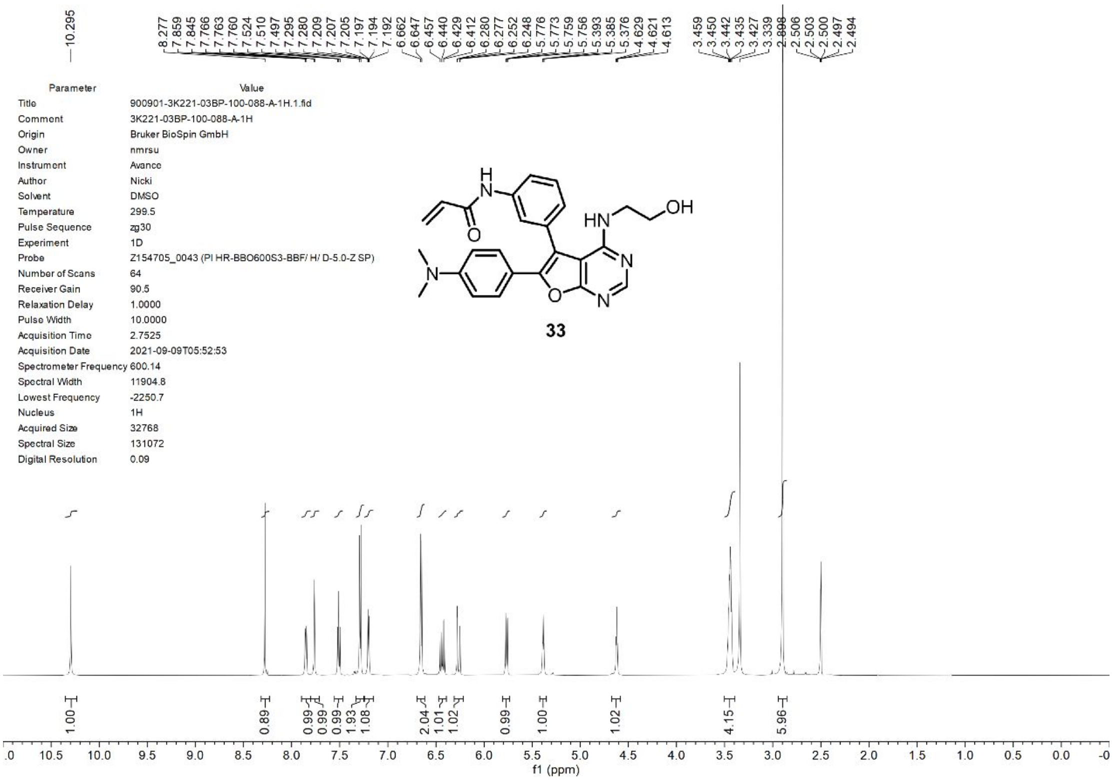

Parameter	Value
Title	900901-3K221-03BP-100-088-A-1H.1.fid
Comment	3K221-03BP-100-088-A-1H
Origin	Bruker BioSpin GmbH
Owner	nmrsu
Instrument	Avance
Author	Nicki
Solvent	DMSO
Temperature	299.5
Pulse Sequence	zg30
Experiment	1D
Probe	Z154705_0043 (PI HR-BBO600S3-BBF/ H/ D-5.0-Z SP)
Number of Scans	64
Receiver Gain	90.5
Relaxation Delay	1.0000
Pulse Width	10.0000
Acquisition Time	2.7525
Acquisition Date	2021-09-09T05:52:53
Spectrometer Frequency	600.14
Spectral Width	11904.8
Lowest Frequency	-2250.7
Nucleus	1H
Acquired Size	32768
Spectral Size	131072
Digital Resolution	0.09

33

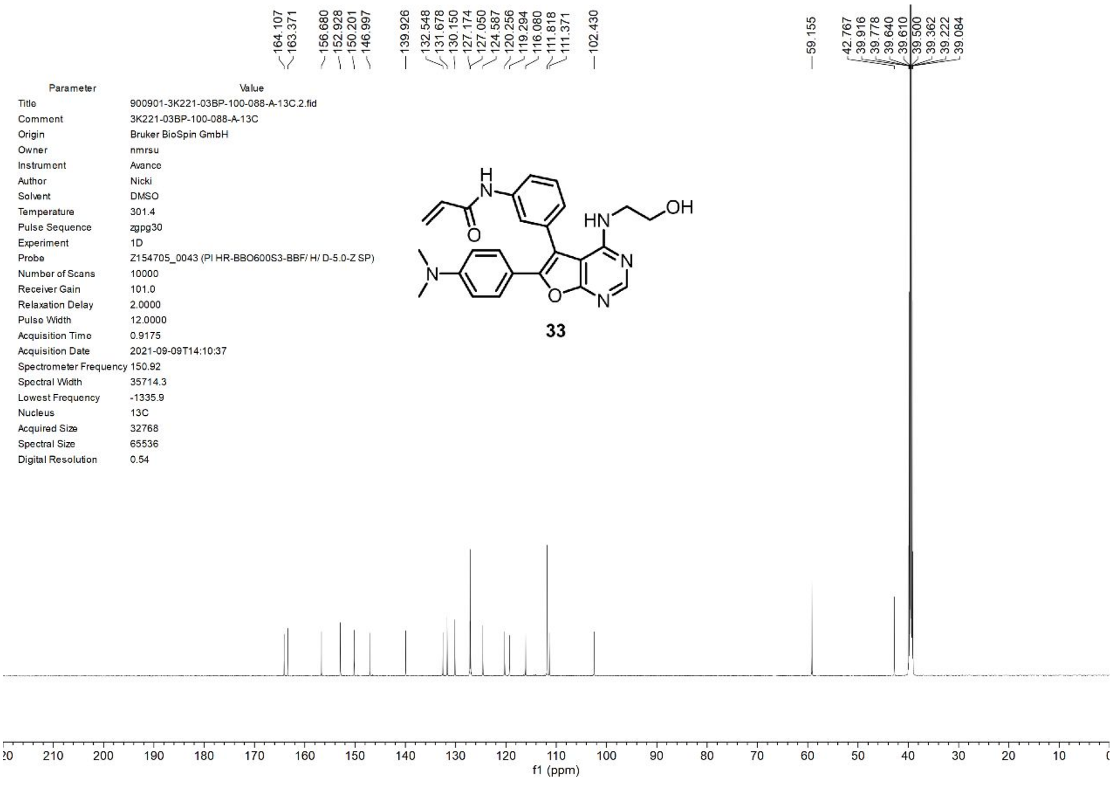

Parameter	Value
Title	900901-3K221-03BP-100-088-A-13C.2.fid
Comment	3K221-03BP-100-088-A-13C
Origin	Bruker BioSpin GmbH
Owner	nmrsu
Instrument	Avance
Author	Nicki
Solvent	DMSO
Temperature	301.4
Pulse Sequence	zgpg30
Experiment	1D
Probe	Z154705_0043 (PI HR-BBO600S3-BBF/ H/ D-5.0-Z SP)
Number of Scans	10000
Receiver Gain	101.0
Relaxation Delay	2.0000
Pulse Width	12.0000
Acquisition Time	0.9175
Acquisition Date	2021-09-09T14:10:37
Spectrometer Frequency	150.92
Spectral Width	35714.3
Lowest Frequency	-1335.9
Nucleus	13C
Acquired Size	32768
Spectral Size	65536
Digital Resolution	0.54

33

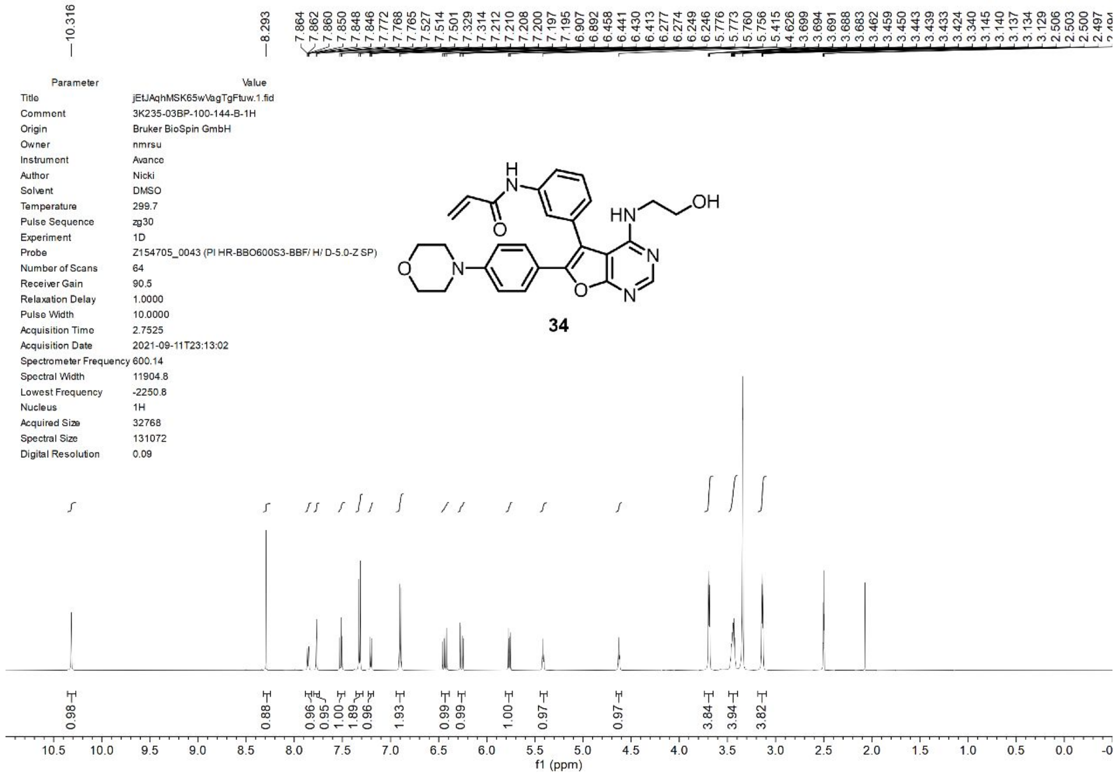

Parameter	Value
Title	jEtJAqhMSK65wVagTgFtuw.1.fid
Comment	3K235-03BP-100-144-B-1H
Origin	Bruker BioSpin GmbH
Owner	nmrsu
Instrument	Avance
Author	Nicki
Solvent	DMSO
Temperature	299.7
Pulse Sequence	zg30
Experiment	1D
Probe	Z154705_0043 (PI HR-BBO600S3-BBF/ H/ D-5.0-Z SP)
Number of Scans	64
Receiver Gain	90.5
Relaxation Delay	1.0000
Pulse Width	10.0000
Acquisition Time	2.7525
Acquisition Date	2021-09-11T23:13:02
Spectrometer Frequency	600.14
Spectral Width	11904.8
Lowest Frequency	-2250.8
Nucleus	1H
Acquired Size	32768
Spectral Size	131072
Digital Resolution	0.09

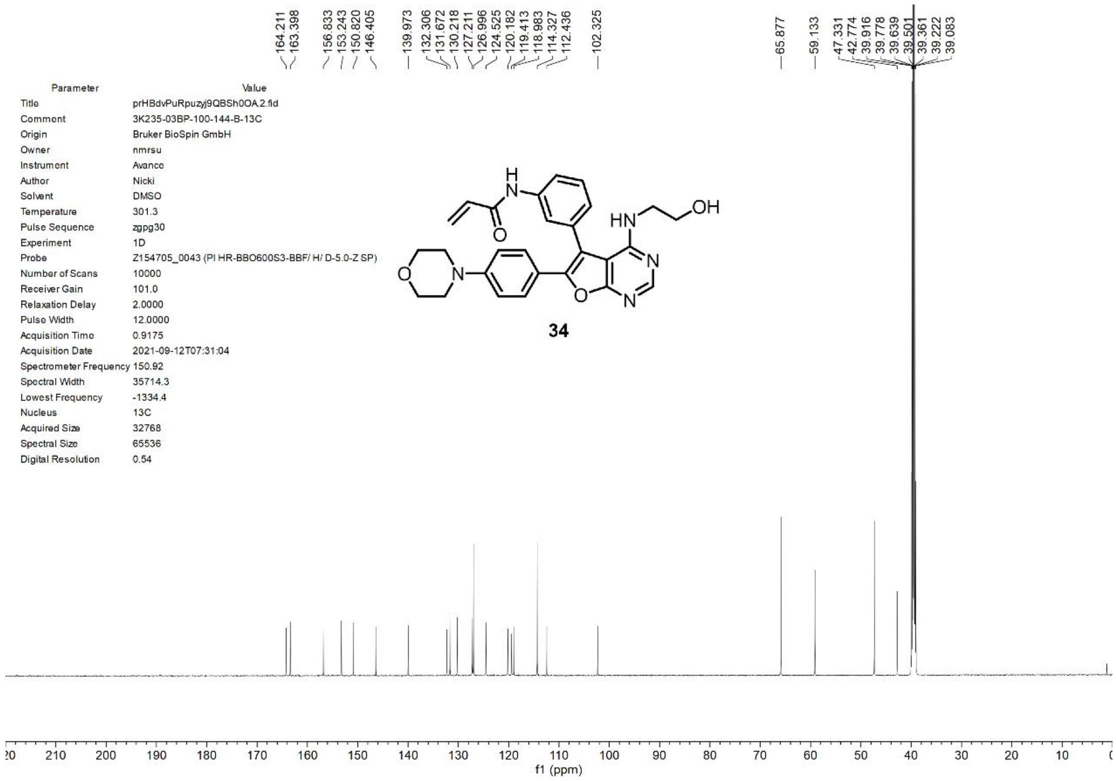

Parameter	Value
Title	prHBdvPuRpuzyj9QBSh0OA.2.fid
Comment	3K235-03BP-100-144-B-13C
Origin	Bruker BioSpin GmbH
Owner	nmrsu
Instrument	Avance
Author	Nicki
Solvent	DMSO
Temperature	301.3
Pulse Sequence	zgpg30
Experiment	1D
Probe	Z154705_0043 (PI HR-BBO600S3-BBF/ H/ D-5.0-Z SP)
Number of Scans	10000
Receiver Gain	101.0
Relaxation Delay	2.0000
Pulse Width	12.0000
Acquisition Time	0.9175
Acquisition Date	2021-09-12T07:31:04
Spectrometer Frequency	150.92
Spectral Width	35714.3
Lowest Frequency	-1334.4
Nucleus	13C
Acquired Size	32768
Spectral Size	65536
Digital Resolution	0.54

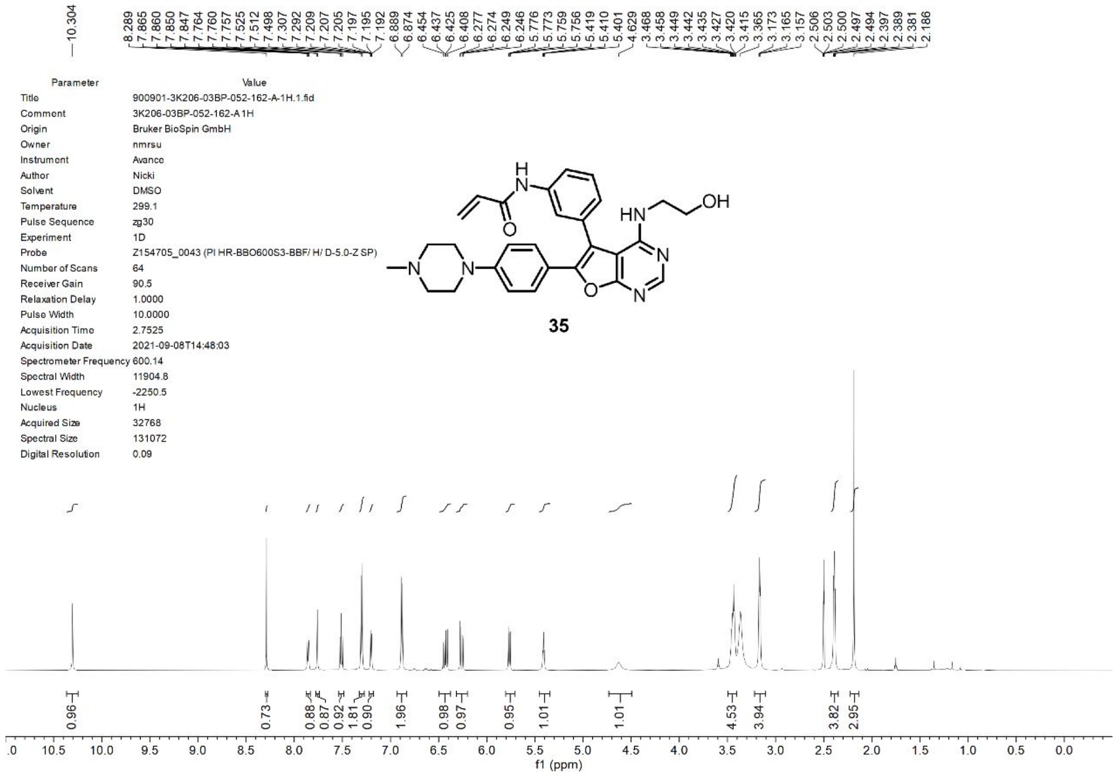

Parameter	Value
Title	900901-3K206-03BP-052-162-A-1H.1.fid
Comment	3K206-03BP-052-162-A1H
Origin	Bruker BioSpin GmbH
Owner	nmrsu
Instrument	Avance
Author	Nicki
Solvent	DMSO
Temperature	299.1
Pulse Sequence	zg30
Experiment	1D
Probe	Z154705_0043 (PI HR-BBO600S3-BBF/ H/ D-5.0-Z SP)
Number of Scans	64
Receiver Gain	90.5
Relaxation Delay	1.0000
Pulse Width	10.0000
Acquisition Time	2.7525
Acquisition Date	2021-09-08T14:48:03
Spectrometer Frequency	600.14
Spectral Width	11904.8
Lowest Frequency	-2250.5
Nucleus	1H
Acquired Size	32768
Spectral Size	131072
Digital Resolution	0.09

35

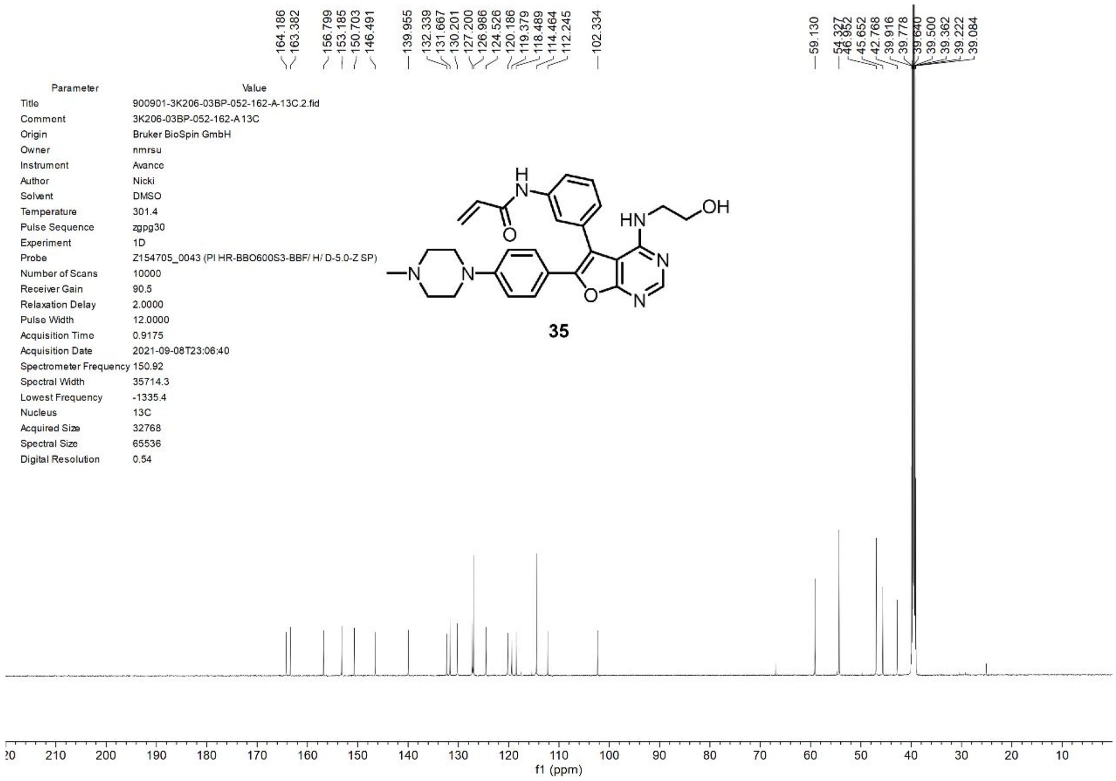

Parameter	Value
Title	900901-3K206-03BP-052-162-A-13C.2.fid
Comment	3K206-03BP-052-162-A13C
Origin	Bruker BioSpin GmbH
Owner	nmrsu
Instrument	Avance
Author	Nicki
Solvent	DMSO
Temperature	301.4
Pulse Sequence	zgpg30
Experiment	1D
Probe	Z154705_0043 (PI HR-BBO600S3-BBF/ H/ D-5.0-Z SP)
Number of Scans	10000
Receiver Gain	90.5
Relaxation Delay	2.0000
Pulse Width	12.0000
Acquisition Time	0.9175
Acquisition Date	2021-09-08T23:06:40
Spectrometer Frequency	150.92
Spectral Width	35714.3
Lowest Frequency	-1335.4
Nucleus	13C
Acquired Size	32768
Spectral Size	65536
Digital Resolution	0.54

35

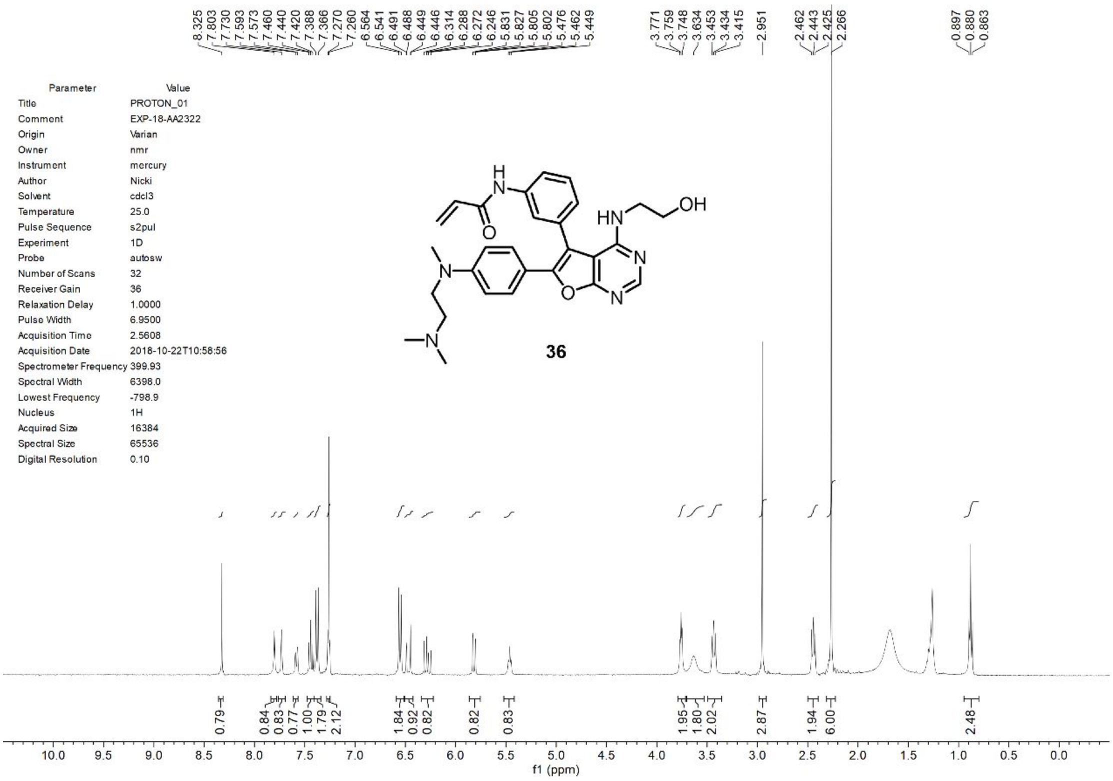

Parameter	Value
Title	PROTON_01
Comment	EXP-18-AA2322
Origin	Varian
Owner	nmr
Instrument	mercury
Author	Nicki
Solvent	cdcl3
Temperature	25.0
Pulse Sequence	s2pul
Experiment	1D
Probe	autosw
Number of Scans	32
Receiver Gain	36
Relaxation Delay	1.0000
Pulse Width	6.9500
Acquisition Time	2.5608
Acquisition Date	2018-10-22T10:58:56
Spectrometer Frequency	399.93
Spectral Width	6398.0
Lowest Frequency	-798.9
Nucleus	1H
Acquired Size	16384
Spectral Size	65536
Digital Resolution	0.10

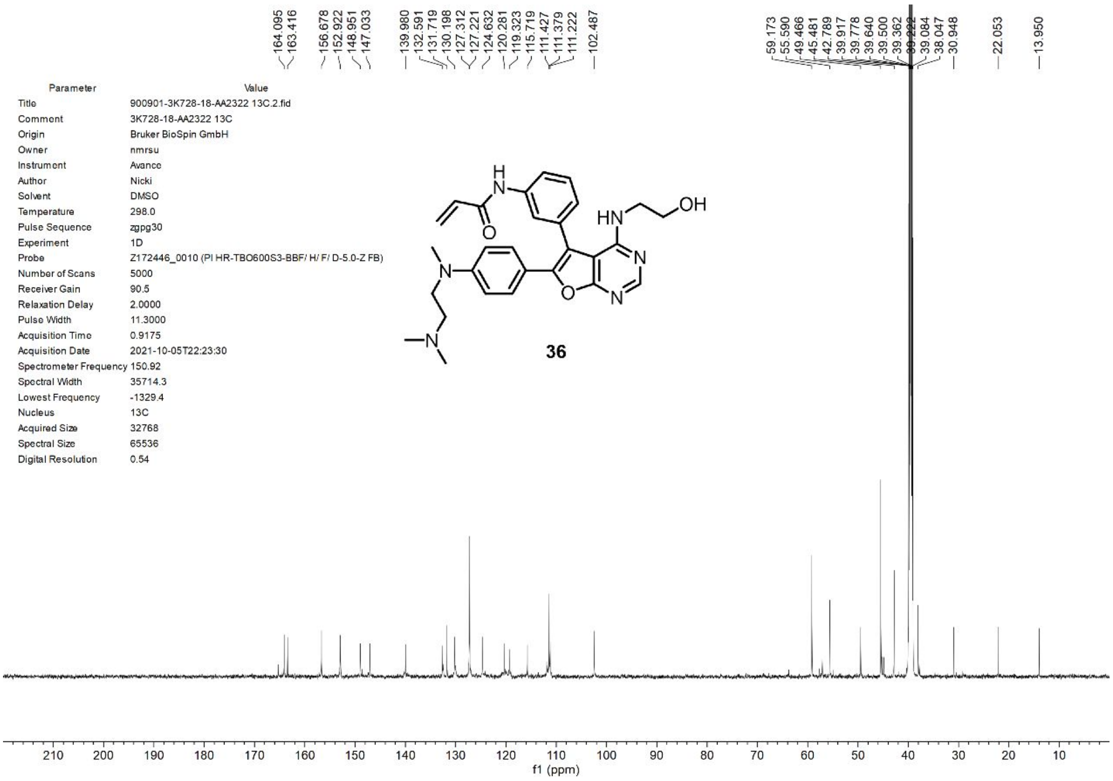

Parameter	Value
Title	900901-3K728-18-AA2322 13C.2.fid
Comment	3K728-18-AA2322 13C
Origin	Bruker BioSpin GmbH
Owner	nmrsu
Instrument	Avance
Author	Nicki
Solvent	DMSO
Temperature	298.0
Pulse Sequence	zgpg30
Experiment	1D
Probe	Z172446_0010 (PI HR-TBO600S3-BBF/ H/ F/ D-5.0-Z FB)
Number of Scans	5000
Receiver Gain	90.5
Relaxation Delay	2.0000
Pulse Width	11.3000
Acquisition Time	0.9175
Acquisition Date	2021-10-05T22:23:30
Spectrometer Frequency	150.92
Spectral Width	35714.3
Lowest Frequency	-1329.4
Nucleus	13C
Acquired Size	32768
Spectral Size	65536
Digital Resolution	0.54

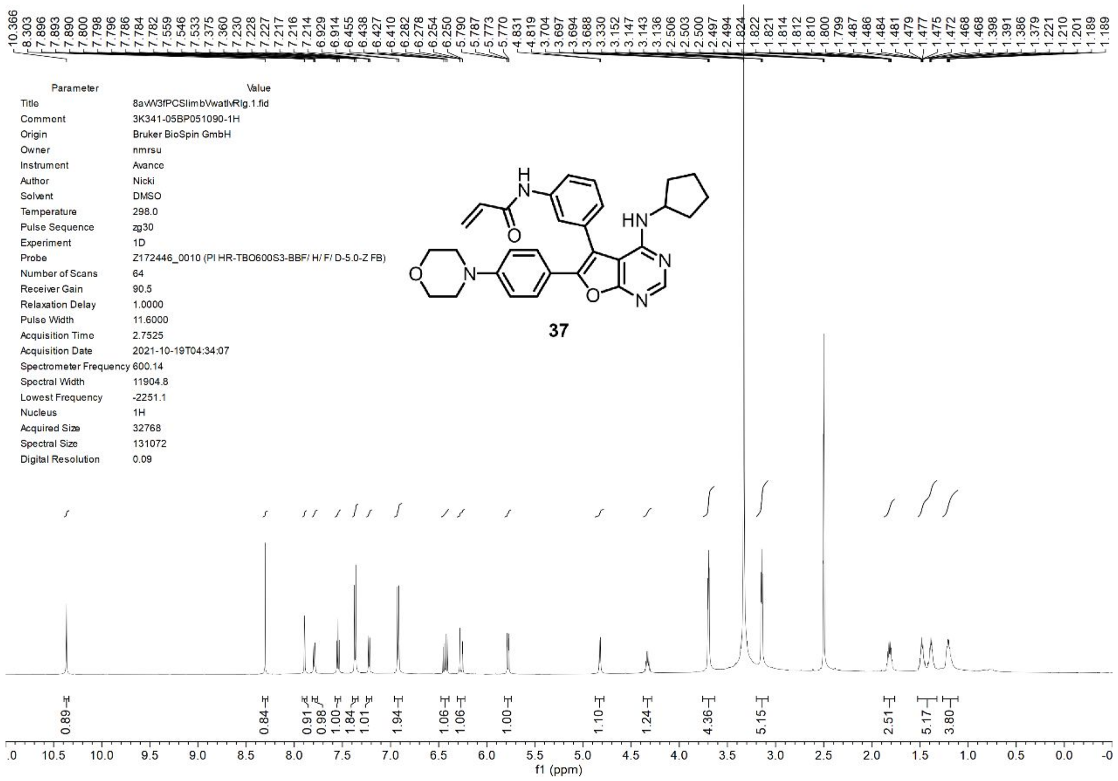
10.366
8.303
7.896
7.893
7.890
7.800
7.798
7.796
7.786
7.784
7.782
7.559
7.546
7.533
7.375
7.360
7.230
7.228
7.227
7.217
7.216
7.214
6.929
6.914
6.455
6.438
6.427
6.410
6.282
6.278
6.254
6.250
5.790
5.787
5.773
5.770
4.831
4.819
3.704
3.697
3.694
3.688
3.330
3.152
3.147
3.143
3.136
2.506
2.503
2.500
2.497
2.494
1.824
1.822
1.821
1.814
1.812
1.810
1.800
1.799
1.487
1.486
1.484
1.481
1.479
1.477
1.475
1.472
1.468
1.398
1.391
1.386
1.379
1.221
1.210
1.201
1.189
Parameter Value
Title 8avW3fPCSlimbVwatlvRlg.1.fid
Comment 3K341-05BP051090-1H
Origin Bruker BioSpin GmbH
Owner nmrsu
Instrument Avance
Author Nicki
Solvent DMSO
Temperature 298.0
Pulse Sequence zg30
Experiment 1D
Probe Z172446_0010 (PI HR-TBO600S3-BBF/ H/ F/ D-5.0-Z FB)
Number of Scans 64
Receiver Gain 90.5
Relaxation Delay 1.0000
Pulse Width 11.6000
Acquisition Time 2.7525
Acquisition Date 2021-10-19T04:34:07
Spectrometer Frequency 600.14
Spectral Width 11904.8
Lowest Frequency -2251.1
Nucleus 1H
Acquired Size 32768
Spectral Size 131072
Digital Resolution 0.09
37
0.89
0.84
0.91
0.98
1.00
1.84
1.01
1.94
1.06
1.06
1.00
1.10
1.24
4.36
5.15
2.51
5.17
3.80
f1 (ppm)

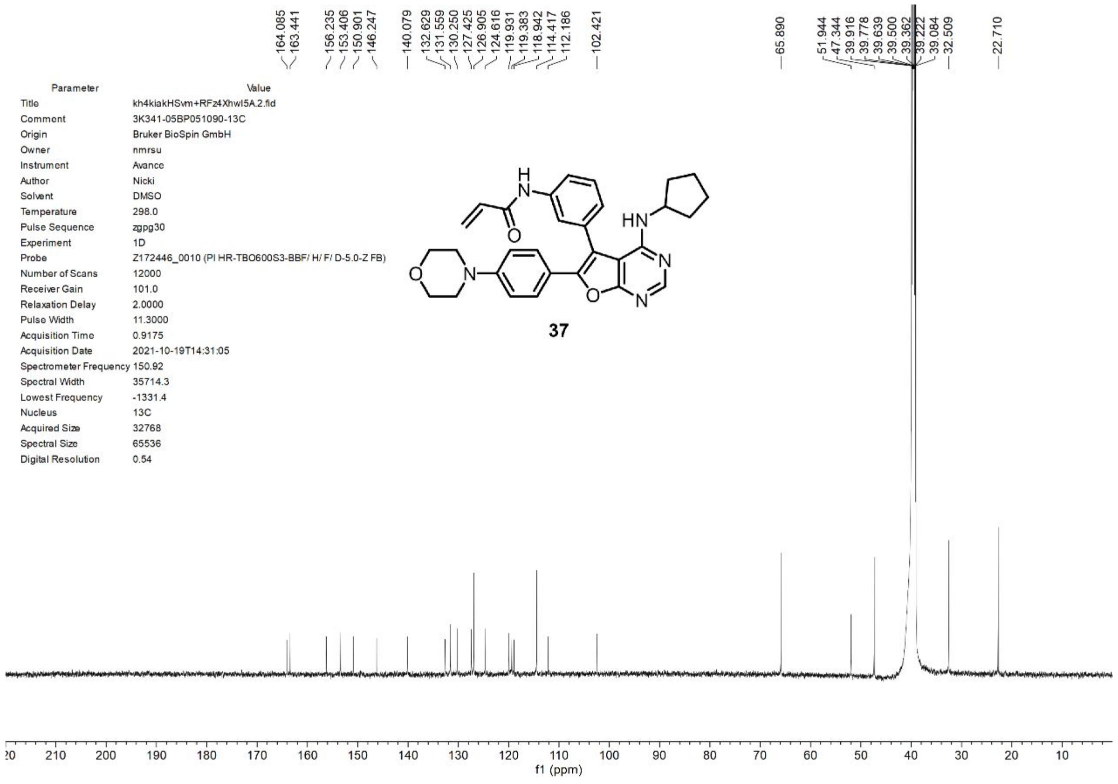
164.085
163.441
156.235
153.406
150.901
146.247
140.079
132.629
131.559
130.250
127.425
126.905
124.616
119.931
119.383
118.942
114.417
112.186
102.421
65.890
51.944
47.344
39.916
39.778
39.639
39.500
39.362
39.222
39.084
32.509
22.710
Parameter Value
Title kh4kiakHSvm+RFz4XhwI5A.2.fid
Comment 3K341-05BP051090-13C
Origin Bruker BioSpin GmbH
Owner nmrsu
Instrument Avance
Author Nicki
Solvent DMSO
Temperature 298.0
Pulse Sequence zgpg30
Experiment 1D
Probe Z172446_0010 (PI HR-TBO600S3-BBF/ H/ F/ D-5.0-Z FB)
Number of Scans 12000
Receiver Gain 101.0
Relaxation Delay 2.0000
Pulse Width 11.3000
Acquisition Time 0.9175
Acquisition Date 2021-10-19T14:31:05
Spectrometer Frequency 150.92
Spectral Width 35714.3
Lowest Frequency -1331.4
Nucleus 13C
Acquired Size 32768
Spectral Size 65536
Digital Resolution 0.54
37
f1 (ppm)

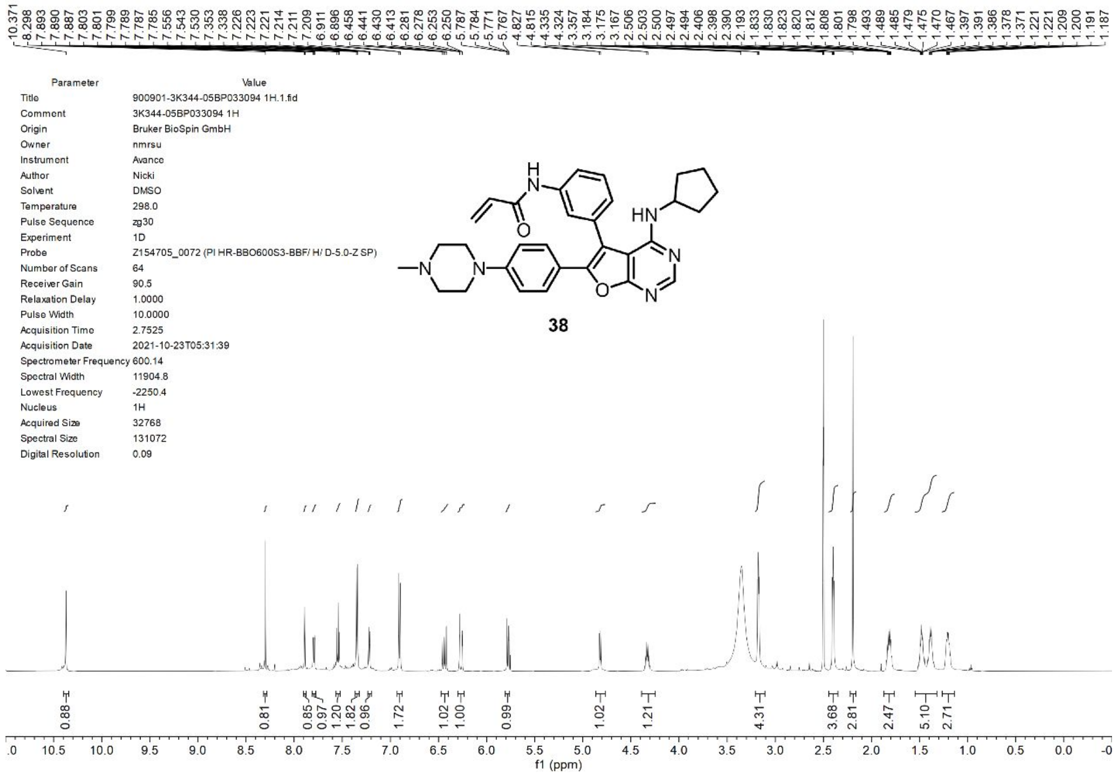
10.371 8.298 7.893 7.890 7.887 7.803 7.801 7.799 7.789 7.787 7.785 7.556 7.543 7.530 7.353 7.338 7.226 7.223 7.221 7.214 7.211 7.209 6.911 6.896 6.458 6.441 6.430 6.413 6.281 6.278 6.253 6.250 5.787 5.784 5.771 5.767 4.827 4.815 4.335 4.324 3.357 3.184 3.175 3.167 2.506 2.503 2.500 2.497 2.494 2.406 2.398 2.390 2.193 1.833 1.830 1.823 1.820 1.812 1.808 1.801 1.798 1.493 1.489 1.485 1.479 1.475 1.470 1.467 1.397 1.391 1.386 1.378 1.371 1.221 1.209 1.200 1.191 1.187
Parameter Value
Title 900901-3K344-05BP033094 1H.1.fid
Comment 3K344-05BP033094 1H
Origin Bruker BioSpin GmbH
Owner nmrsu
Instrument Avance
Author Nicki
Solvent DMSO
Temperature 298.0
Pulse Sequence zg30
Experiment 1D
Probe Z154705_0072 (PI HR-BBO600S3-BBF/ H/ D-5.0-Z SP)
Number of Scans 64
Receiver Gain 90.5
Relaxation Delay 1.0000
Pulse Width 10.0000
Acquisition Time 2.7525
Acquisition Date 2021-10-23T05:31:39
Spectrometer Frequency 600.14
Spectral Width 11904.8
Lowest Frequency -2250.4
Nucleus 1H
Acquired Size 32768
Spectral Size 131072
Digital Resolution 0.09
38
0.88 0.81 0.85 0.97 1.20 1.82 0.96 1.72 1.02 1.00 0.99 1.02 1.21 4.31 3.68 2.81 2.47 5.10 2.71
f1 (ppm)

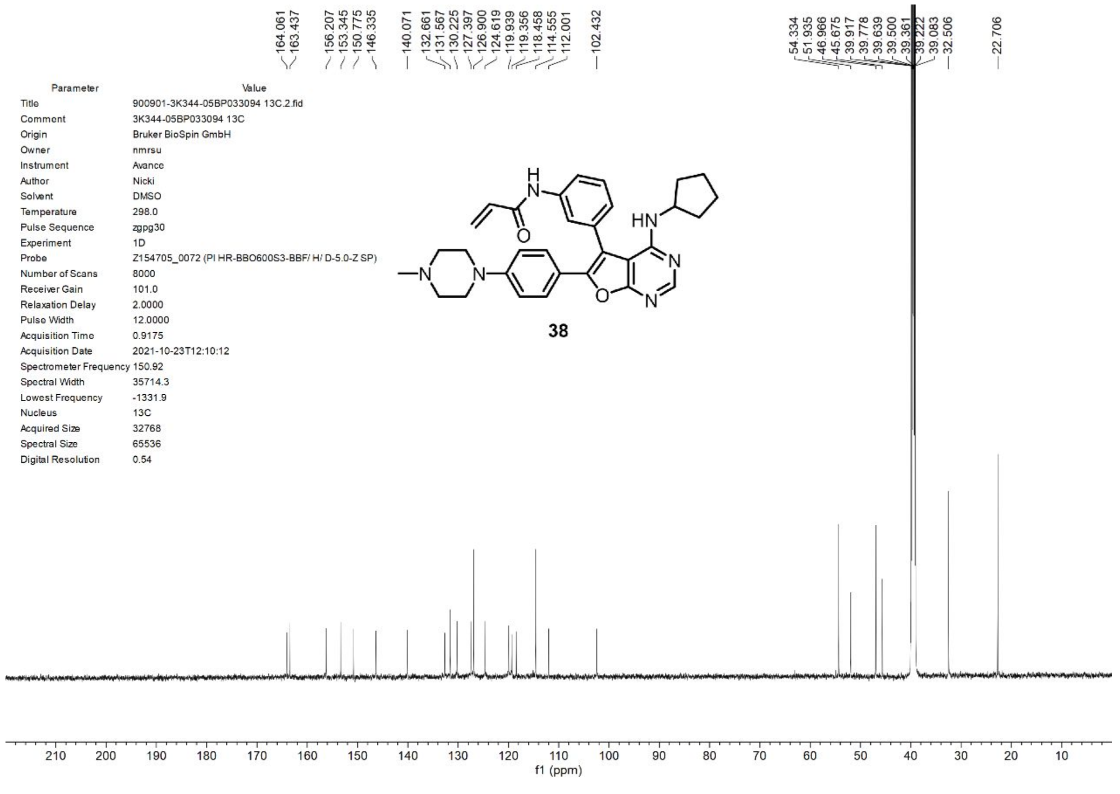
164.061 163.437 156.207 153.345 150.775 146.335 140.071 132.661 131.567 130.225 127.397 126.900 124.619 119.939 119.356 118.458 114.555 112.001 102.432 54.334 51.935 46.966 45.675 39.917 39.778 39.639 39.500 39.361 39.222 39.083 32.506 22.706
Parameter Value
Title 900901-3K344-05BP033094 13C.2.fid
Comment 3K344-05BP033094 13C
Origin Bruker BioSpin GmbH
Owner nmrsu
Instrument Avance
Author Nicki
Solvent DMSO
Temperature 298.0
Pulse Sequence zgpg30
Experiment 1D
Probe Z154705_0072 (PI HR-BBO600S3-BBF/ H/ D-5.0-Z SP)
Number of Scans 8000
Receiver Gain 101.0
Relaxation Delay 2.0000
Pulse Width 12.0000
Acquisition Time 0.9175
Acquisition Date 2021-10-23T12:10:12
Spectrometer Frequency 150.92
Spectral Width 35714.3
Lowest Frequency -1331.9
Nucleus 13C
Acquired Size 32768
Spectral Size 65536
Digital Resolution 0.54
38
f1 (ppm)

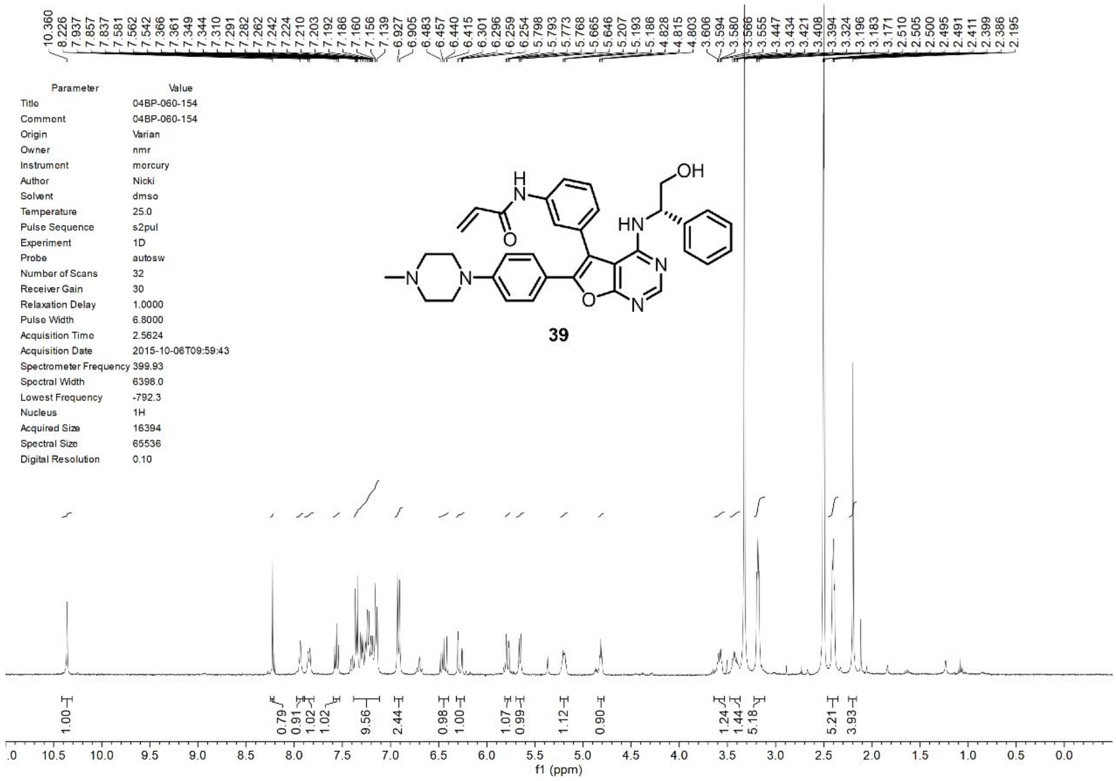
10.360
8.226
7.937
7.857
7.837
7.581
7.562
7.542
7.366
7.361
7.349
7.344
7.310
7.291
7.282
7.262
7.242
7.224
7.210
7.203
7.192
7.186
7.160
7.156
7.139
6.927
6.905
6.483
6.457
6.440
6.415
6.301
6.296
6.259
6.254
5.798
5.793
5.773
5.768
5.665
5.646
5.207
5.193
5.186
4.828
4.815
4.803
3.606
3.594
3.580
3.566
3.555
3.447
3.434
3.421
3.408
3.394
3.324
3.196
3.183
3.171
2.510
2.505
2.500
2.495
2.491
2.411
2.399
2.386
2.195
Parameter Value
Title 04BP-060-154
Comment 04BP-060-154
Origin Varian
Owner nmr
Instrument mercury
Author Nicki
Solvent dmso
Temperature 25.0
Pulse Sequence s2pul
Experiment 1D
Probe autosw
Number of Scans 32
Receiver Gain 30
Relaxation Delay 1.0000
Pulse Width 6.8000
Acquisition Time 2.5624
Acquisition Date 2015-10-06T09:59:43
Spectrometer Frequency 399.93
Spectral Width 6398.0
Lowest Frequency -792.3
Nucleus 1H
Acquired Size 16394
Spectral Size 65536
Digital Resolution 0.10
OH
N
H
O
HN
N
N
O
N
N
39
1.00
0.79
0.91
1.02
1.02
9.56
2.44
0.98
1.00
1.07
0.99
1.12
0.90
1.24
1.44
5.18
5.21
3.93
10.5 10.0 9.5 9.0 8.5 8.0 7.5 7.0 6.5 6.0 5.5 5.0 4.5 4.0 3.5 3.0 2.5 2.0 1.5 1.0 0.5 0.0
f1 (ppm)

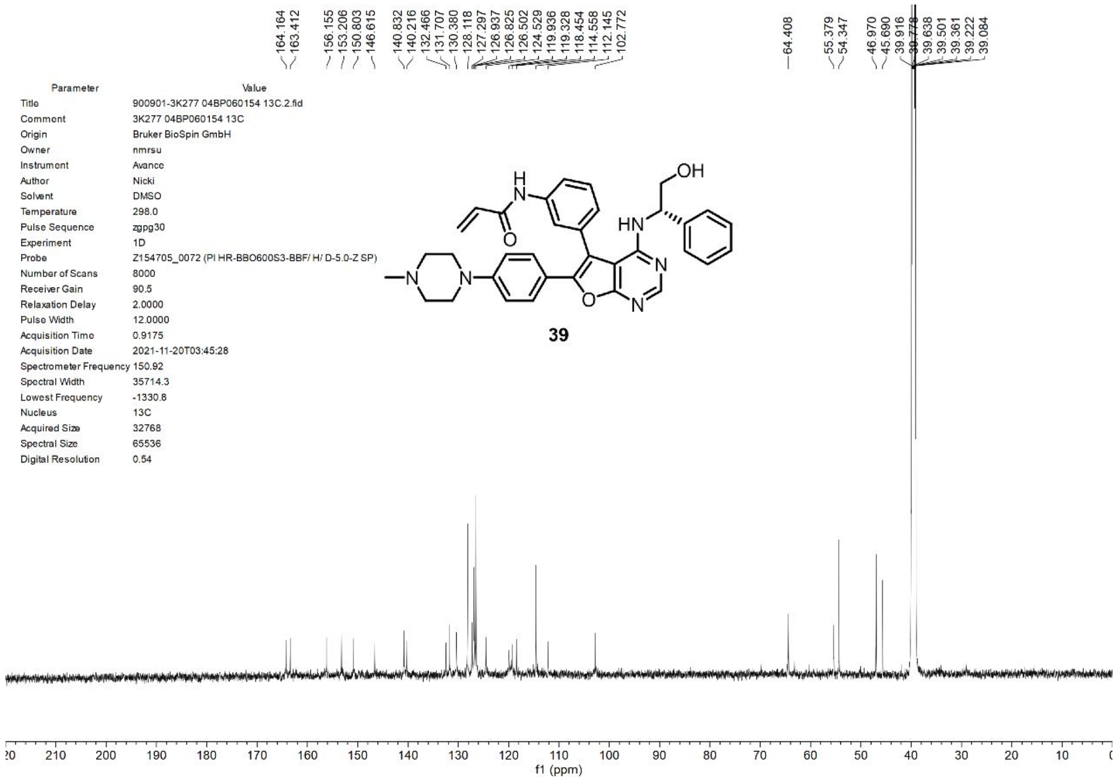
164.164
163.412
156.155
153.206
150.803
146.615
140.832
140.216
132.466
131.707
130.380
128.118
127.297
126.937
126.825
126.502
124.529
119.936
119.328
118.454
114.558
112.145
102.772
64.408
55.379
54.347
46.970
45.690
39.916
39.778
39.638
39.501
39.361
39.222
39.084
Parameter Value
Title 900901-3K277 04BP060154 13C.2.fid
Comment 3K277 04BP060154 13C
Origin Bruker BioSpin GmbH
Owner nmrsu
Instrument Avance
Author Nicki
Solvent DMSO
Temperature 298.0
Pulse Sequence zgpg30
Experiment 1D
Probe Z154705_0072 (PI HR-BBO600S3-BBF/ H/ D-5.0-Z SP)
Number of Scans 8000
Receiver Gain 90.5
Relaxation Delay 2.0000
Pulse Width 12.0000
Acquisition Time 0.9175
Acquisition Date 2021-11-20T03:45:28
Spectrometer Frequency 150.92
Spectral Width 35714.3
Lowest Frequency -1330.8
Nucleus 13C
Acquired Size 32768
Spectral Size 65536
Digital Resolution 0.54
OH
N
H
O
HN
N
N
O
N
N
39
210 200 190 180 170 160 150 140 130 120 110 100 90 80 70 60 50 40 30 20 10
f1 (ppm)

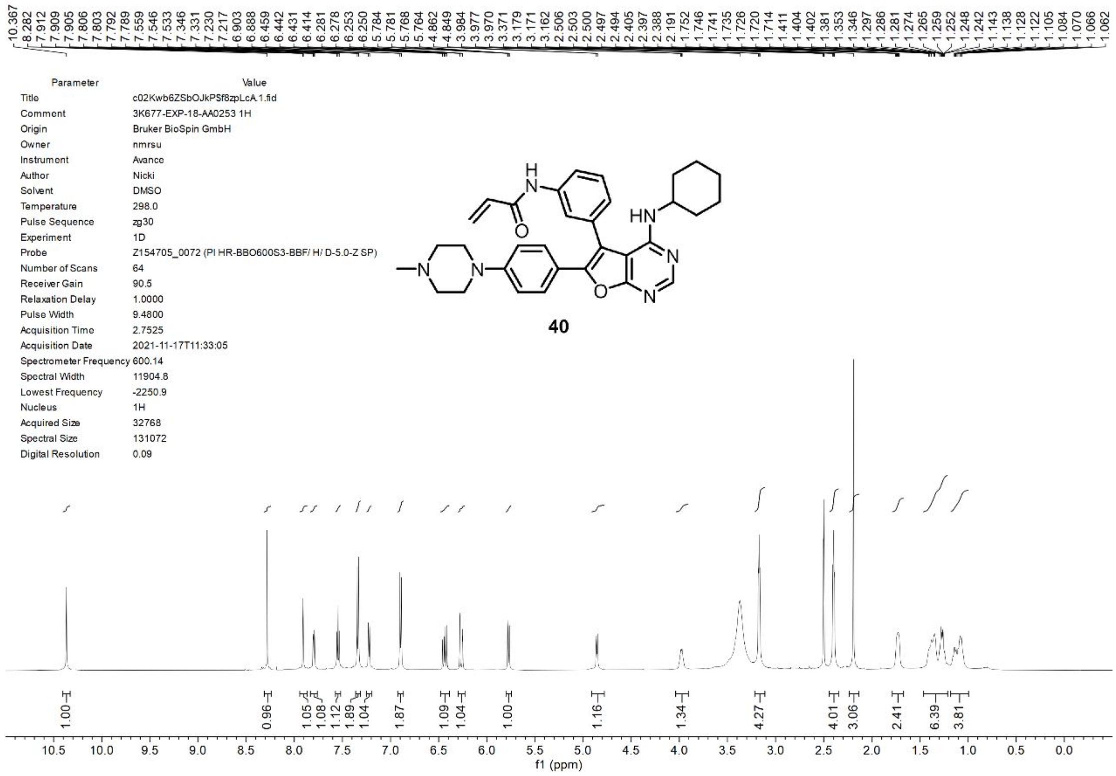
10.367 8.282 7.912 7.909 7.905 7.806 7.803 7.792 7.789 7.559 7.546 7.533 7.346 7.331 7.230 7.217 6.903 6.888 6.459 6.442 6.431 6.414 6.281 6.278 6.253 6.250 5.784 5.781 5.768 5.764 4.862 4.849 3.984 3.977 3.970 3.371 3.179 3.171 3.162 2.506 2.503 2.500 2.497 2.494 2.405 2.397 2.388 2.191 1.752 1.746 1.741 1.735 1.726 1.720 1.714 1.411 1.404 1.402 1.381 1.353 1.346 1.297 1.286 1.281 1.274 1.265 1.259 1.252 1.248 1.242 1.143 1.138 1.122 1.105 1.084 1.070 1.066 1.062
Parameter Value
Title c02Kwb6ZSbOJkPSf8zpLcA.1.fid
Comment 3K677-EXP-18-AA0253 1H
Origin Bruker BioSpin GmbH
Owner nmrsu
Instrument Avance
Author Nicki
Solvent DMSO
Temperature 298.0
Pulse Sequence zg30
Experiment 1D
Probe Z154705_0072 (PI HR-BBO600S3-BBF/ H/ D-5.0-Z SP)
Number of Scans 64
Receiver Gain 90.5
Relaxation Delay 1.0000
Pulse Width 9.4800
Acquisition Time 2.7525
Acquisition Date 2021-11-17T11:33:05
Spectrometer Frequency 600.14
Spectral Width 11904.8
Lowest Frequency -2250.9
Nucleus 1H
Acquired Size 32768
Spectral Size 131072
Digital Resolution 0.09
40
1.00 0.96 1.05 1.08 1.12 1.89 1.04 1.87 1.09 1.04 1.00 1.16 1.34 4.27 4.01 3.06 2.41 6.39 3.81
f1 (ppm)

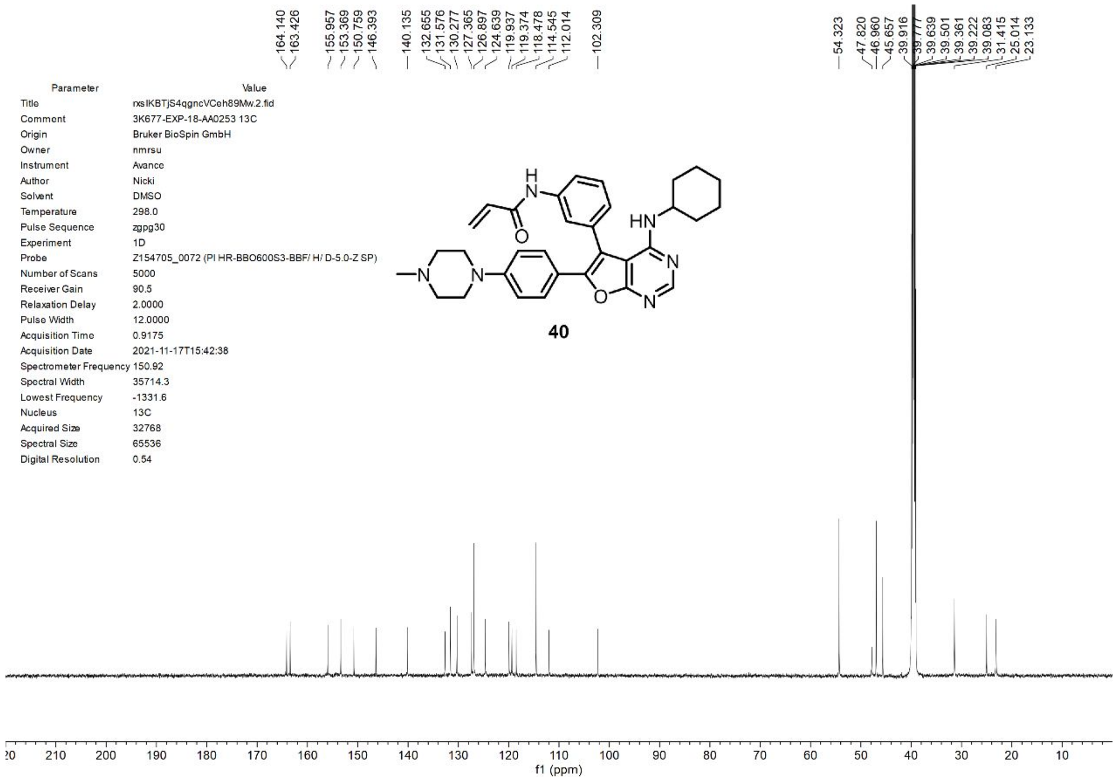
164.140 163.426 155.957 153.369 150.759 146.393 140.135 132.655 131.576 130.277 127.365 126.897 124.639 119.937 119.374 118.478 114.545 112.014 102.309 54.323 47.820 46.960 45.657 39.916 39.777 39.639 39.501 39.361 39.222 39.083 31.415 25.014 23.133
Parameter Value
Title rxsIKBTjS4qgncVCoh89Mw.2.fid
Comment 3K677-EXP-18-AA0253 13C
Origin Bruker BioSpin GmbH
Owner nmrsu
Instrument Avance
Author Nicki
Solvent DMSO
Temperature 298.0
Pulse Sequence zgpg30
Experiment 1D
Probe Z154705_0072 (PI HR-BBO600S3-BBF/ H/ D-5.0-Z SP)
Number of Scans 5000
Receiver Gain 90.5
Relaxation Delay 2.0000
Pulse Width 12.0000
Acquisition Time 0.9175
Acquisition Date 2021-11-17T15:42:38
Spectrometer Frequency 150.92
Spectral Width 35714.3
Lowest Frequency -1331.6
Nucleus 13C
Acquired Size 32768
Spectral Size 65536
Digital Resolution 0.54
40
f1 (ppm)

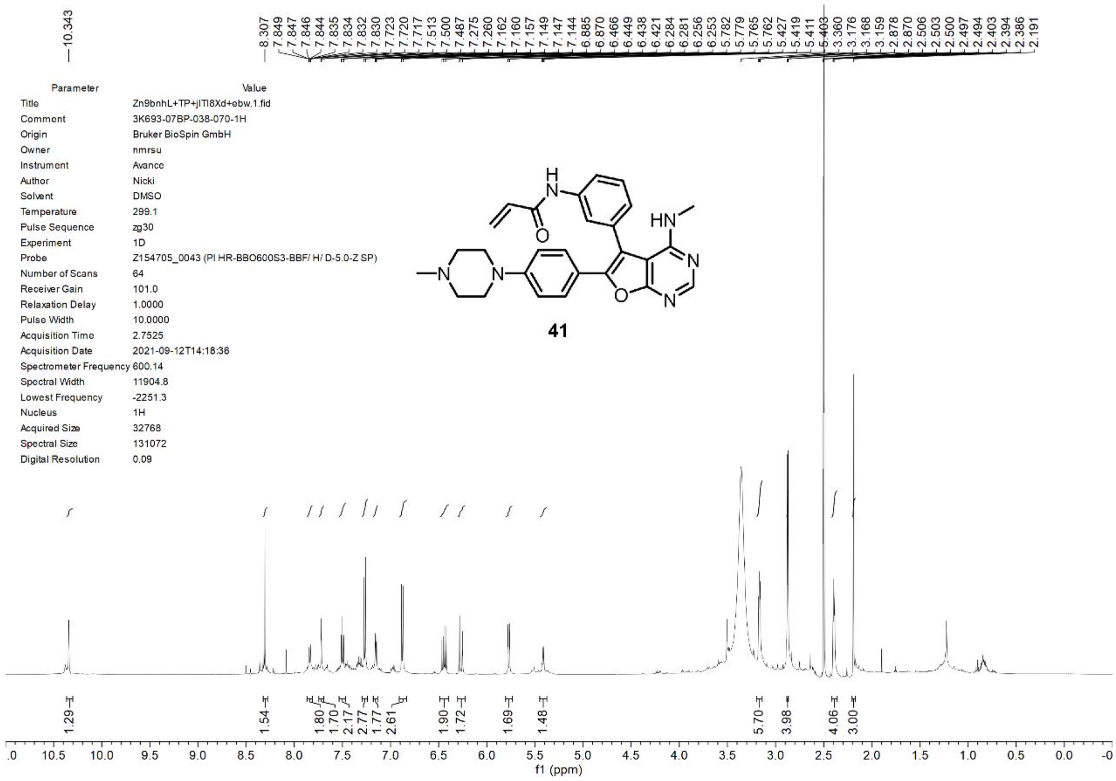

41

Parameter	Value
Title	Zn9bnhL+TP+jlTl8Xd+obw.1.fid
Comment	3K693-07BP-038-070-1H
Origin	Bruker BioSpin GmbH
Owner	nmrsu
Instrument	Avance
Author	Nicki
Solvent	DMSO
Temperature	299.1
Pulse Sequence	zg30
Experiment	1D
Probe	Z154705_0043 (PI HR-BBO600S3-BBF/ H/ D-5.0-Z SP)
Number of Scans	64
Receiver Gain	101.0
Relaxation Delay	1.0000
Pulse Width	10.0000
Acquisition Time	2.7525
Acquisition Date	2021-09-12T14:18:36
Spectrometer Frequency	600.14
Spectral Width	11904.8
Lowest Frequency	-2251.3
Nucleus	1H
Acquired Size	32768
Spectral Size	131072
Digital Resolution	0.09

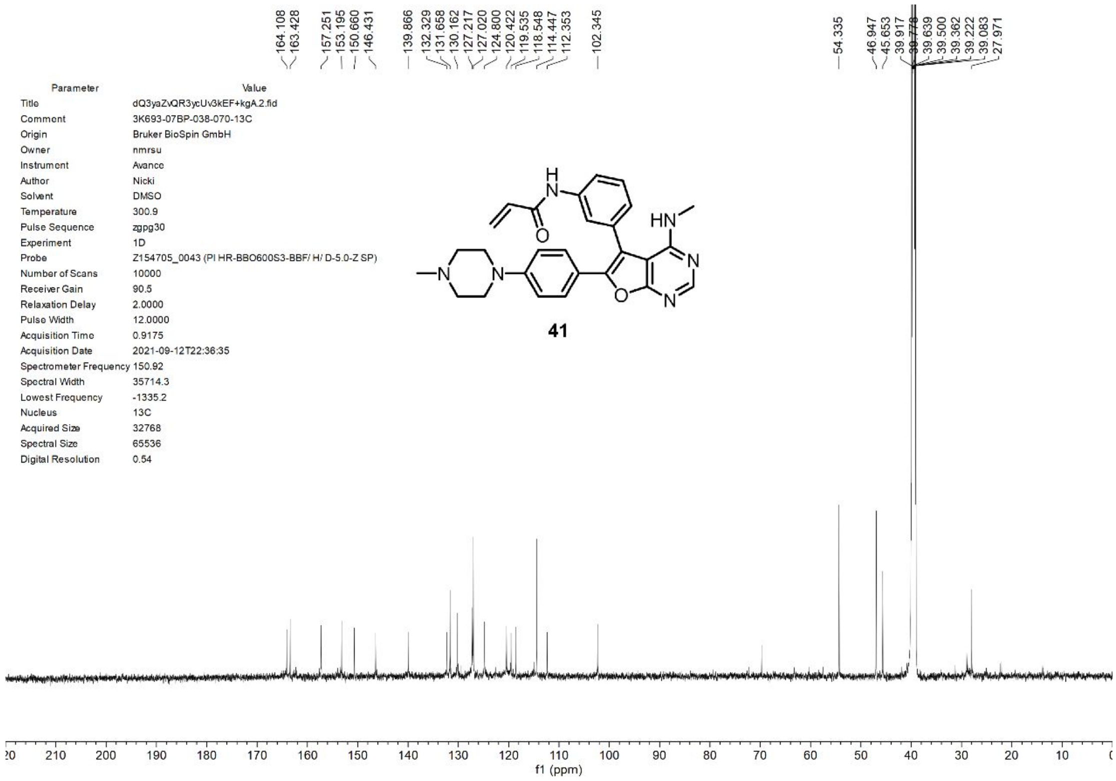

41

Parameter	Value
Title	dQ3yaZvQR3ycUv3kEF+kgA.2.fid
Comment	3K693-07BP-038-070-13C
Origin	Bruker BioSpin GmbH
Owner	nmrsu
Instrument	Avance
Author	Nicki
Solvent	DMSO
Temperature	300.9
Pulse Sequence	zgpg30
Experiment	1D
Probe	Z154705_0043 (PI HR-BBO600S3-BBF/ H/ D-5.0-Z SP)
Number of Scans	10000
Receiver Gain	90.5
Relaxation Delay	2.0000
Pulse Width	12.0000
Acquisition Time	0.9175
Acquisition Date	2021-09-12T22:36:35
Spectrometer Frequency	150.92
Spectral Width	35714.3
Lowest Frequency	-1335.2
Nucleus	13C
Acquired Size	32768
Spectral Size	65536
Digital Resolution	0.54

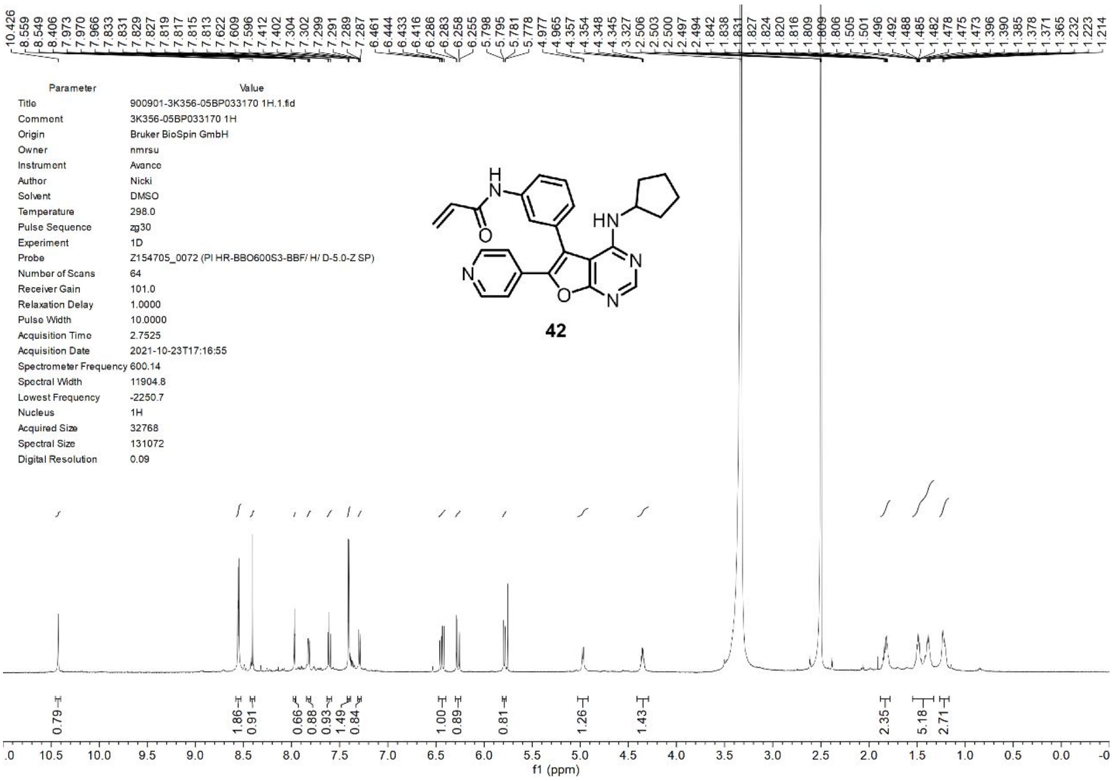
10.426 8.559 8.549 8.406 7.973 7.970 7.966 7.833 7.831 7.829 7.827 7.819 7.817 7.815 7.813 7.622 7.609 7.596 7.412 7.402 7.304 7.302 7.299 7.291 7.289 7.287 6.461 6.444 6.433 6.416 6.286 6.283 6.258 6.255 5.798 5.795 5.781 5.778 4.977 4.965 4.357 4.354 4.348 4.345 3.327 2.506 2.503 2.500 2.497 2.494 1.842 1.838 1.831 1.827 1.824 1.820 1.816 1.809 1.806 1.505 1.501 1.496 1.492 1.488 1.485 1.482 1.478 1.475 1.473 1.396 1.390 1.385 1.378 1.371 1.365 1.232 1.223 1.214
Parameter Value
Title 900901-3K356-05BP033170 1H.1.fid
Comment 3K356-05BP033170 1H
Origin Bruker BioSpin GmbH
Owner nmrsu
Instrument Avance
Author Nicki
Solvent DMSO
Temperature 298.0
Pulse Sequence zg30
Experiment 1D
Probe Z154705_0072 (PI HR-BBO600S3-BBF/ H/ D-5.0-Z SP)
Number of Scans 64
Receiver Gain 101.0
Relaxation Delay 1.0000
Pulse Width 10.0000
Acquisition Time 2.7525
Acquisition Date 2021-10-23T17:16:55
Spectrometer Frequency 600.14
Spectral Width 11904.8
Lowest Frequency -2250.7
Nucleus 1H
Acquired Size 32768
Spectral Size 131072
Digital Resolution 0.09
42
0.79 1.86 0.91 0.66 0.88 0.93 1.49 0.84 1.00 0.89 0.81 1.26 1.43 2.35 5.18 2.71
f1 (ppm)

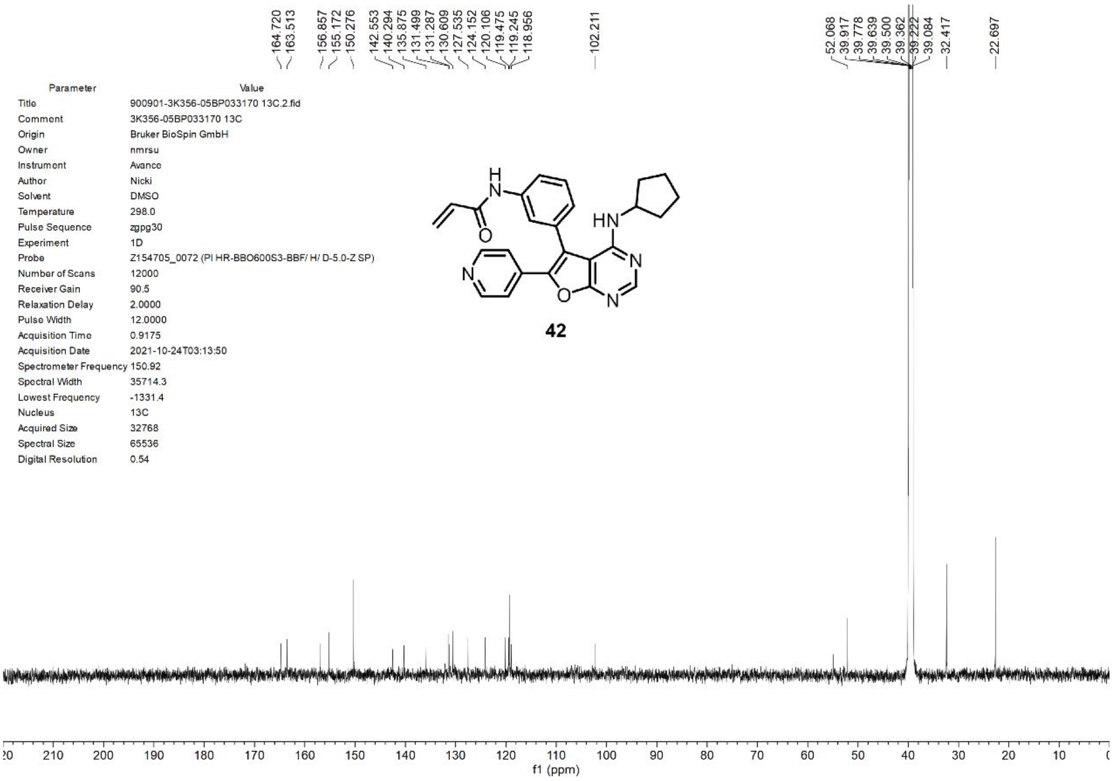
164.720 163.513 156.857 155.172 150.276 142.553 140.294 135.875 131.499 131.287 130.609 127.535 124.152 120.106 119.475 119.245 118.956 102.211 52.068 39.917 39.778 39.639 39.500 39.362 39.222 39.084 32.417 22.697
Parameter Value
Title 900901-3K356-05BP033170 13C.2.fid
Comment 3K356-05BP033170 13C
Origin Bruker BioSpin GmbH
Owner nmrsu
Instrument Avance
Author Nicki
Solvent DMSO
Temperature 298.0
Pulse Sequence zgpg30
Experiment 1D
Probe Z154705_0072 (PI HR-BBO600S3-BBF/ H/ D-5.0-Z SP)
Number of Scans 12000
Receiver Gain 90.5
Relaxation Delay 2.0000
Pulse Width 12.0000
Acquisition Time 0.9175
Acquisition Date 2021-10-24T03:13:50
Spectrometer Frequency 150.92
Spectral Width 35714.3
Lowest Frequency -1331.4
Nucleus 13C
Acquired Size 32768
Spectral Size 65536
Digital Resolution 0.54
42
f1 (ppm)

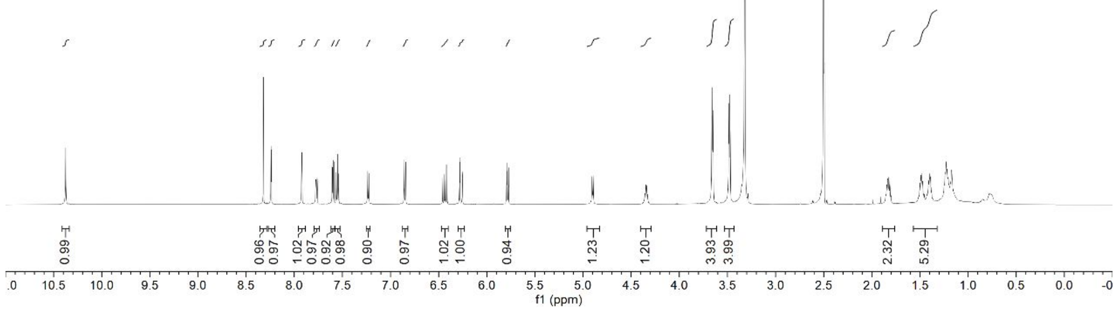

Parameter	Value
Title	900901-3K680 05BP086036 1H.1.fid
Comment	3K680 05BP086036 1H
Origin	Bruker BioSpin GmbH
Owner	nmrsu
Instrument	Avance
Author	Nicki
Solvent	DMSO
Temperature	298.0
Pulse Sequence	zg30
Experiment	1D
Probe	Z154705_0072 (PI HR-BBO600S3-BBF/ H/ D-5.0-Z SP)
Number of Scans	128
Receiver Gain	101.0
Relaxation Delay	1.0000
Pulse Width	9.4800
Acquisition Time	2.7525
Acquisition Date	2021-11-22T11:24:23
Spectrometer Frequency	600.14
Spectral Width	11904.8
Lowest Frequency	-2250.6
Nucleus	1H
Acquired Size	32768
Spectral Size	131072
Digital Resolution	0.09

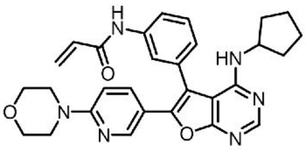

43

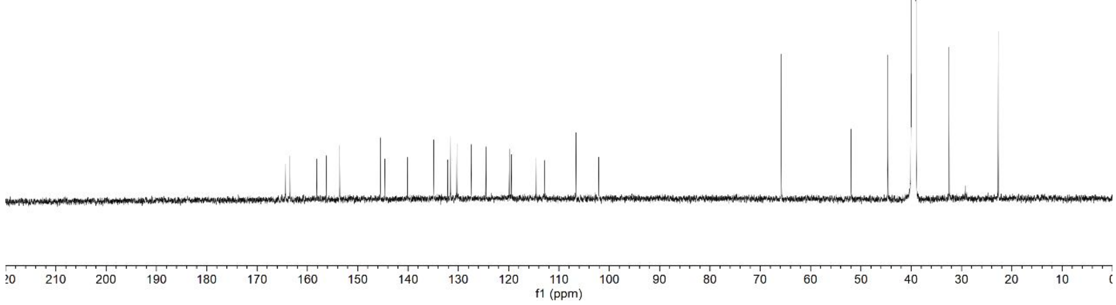

Parameter	Value
Title	900901-3K680 05BP086036 13C.2.fid
Comment	3K680 05BP086036 13C
Origin	Bruker BioSpin GmbH
Owner	nmrsu
Instrument	Avance
Author	Nicki
Solvent	DMSO
Temperature	298.0
Pulse Sequence	zgpg30
Experiment	1D
Probe	Z154705_0072 (PI HR-BBO600S3-BBF/ H/ D-5.0-Z SP)
Number of Scans	16000
Receiver Gain	90.5
Relaxation Delay	2.0000
Pulse Width	12.0000
Acquisition Time	0.9175
Acquisition Date	2021-11-23T00:39:48
Spectrometer Frequency	150.92
Spectral Width	35714.3
Lowest Frequency	-1332.5
Nucleus	13C
Acquired Size	32768
Spectral Size	65536
Digital Resolution	0.54

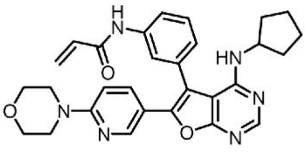

43

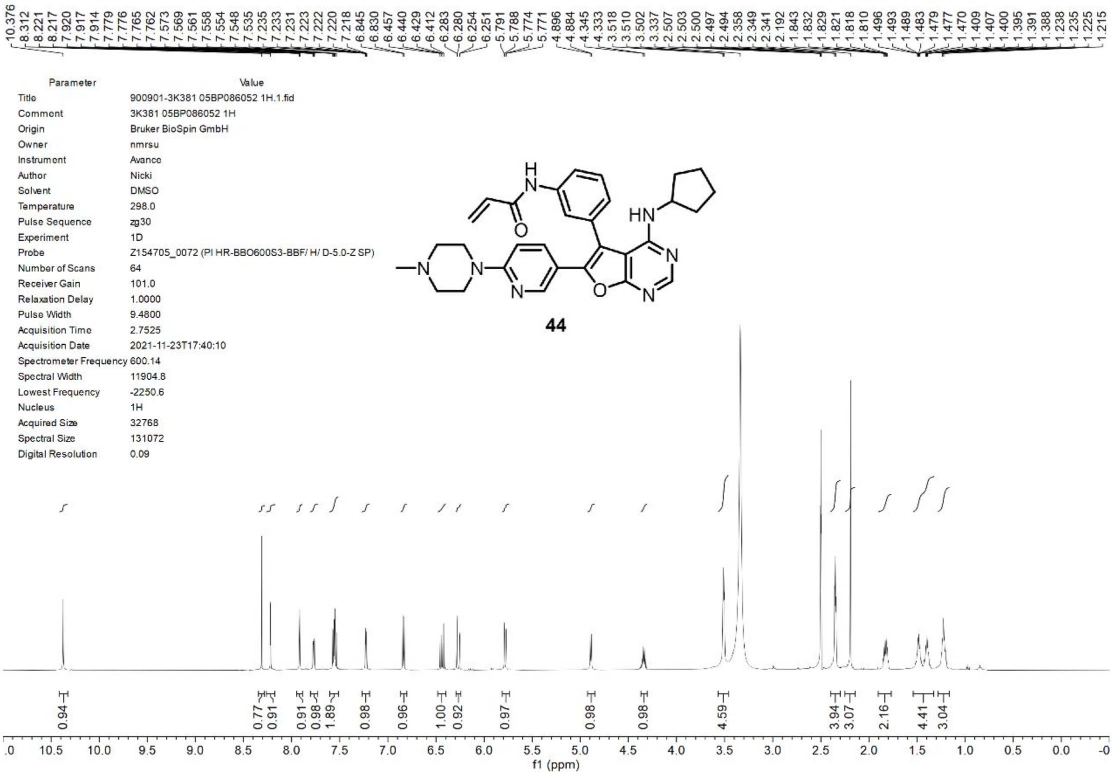

Parameter	Value
Title	900901-3K381 05BP086052 1H.1.fid
Comment	3K381 05BP086052 1H
Origin	Bruker BioSpin GmbH
Owner	nmrsu
Instrument	Avance
Author	Nicki
Solvent	DMSO
Temperature	298.0
Pulse Sequence	zg30
Experiment	1D
Probe	Z154705_0072 (PI HR-BBO600S3-BBF/ H/ D-5.0-Z SP)
Number of Scans	64
Receiver Gain	101.0
Relaxation Delay	1.0000
Pulse Width	9.4800
Acquisition Time	2.7525
Acquisition Date	2021-11-23T17:40:10
Spectrometer Frequency	600.14
Spectral Width	11904.8
Lowest Frequency	-2250.6
Nucleus	1H
Acquired Size	32768
Spectral Size	131072
Digital Resolution	0.09

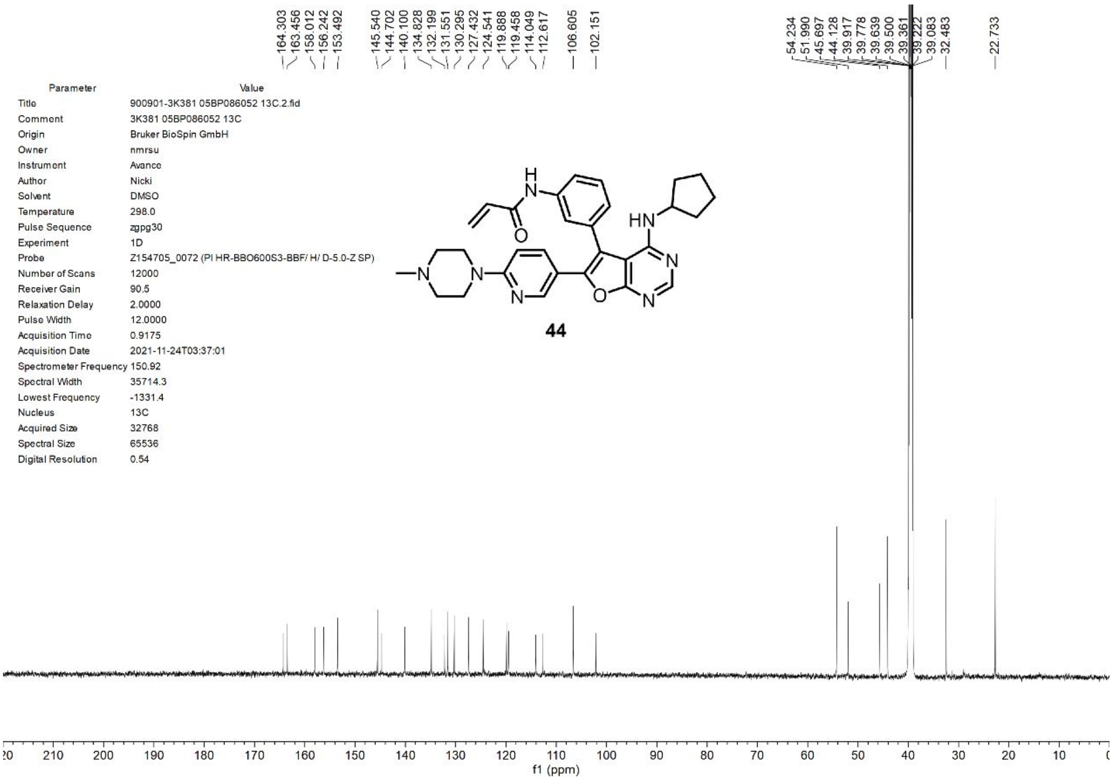

Parameter	Value
Title	900901-3K381 05BP086052 13C.2.fid
Comment	3K381 05BP086052 13C
Origin	Bruker BioSpin GmbH
Owner	nmrsu
Instrument	Avance
Author	Nicki
Solvent	DMSO
Temperature	298.0
Pulse Sequence	zgpg30
Experiment	1D
Probe	Z154705_0072 (PI HR-BBO600S3-BBF/ H/ D-5.0-Z SP)
Number of Scans	12000
Receiver Gain	90.5
Relaxation Delay	2.0000
Pulse Width	12.0000
Acquisition Time	0.9175
Acquisition Date	2021-11-24T03:37:01
Spectrometer Frequency	150.92
Spectral Width	35714.3
Lowest Frequency	-1331.4
Nucleus	13C
Acquired Size	32768
Spectral Size	65536
Digital Resolution	0.54

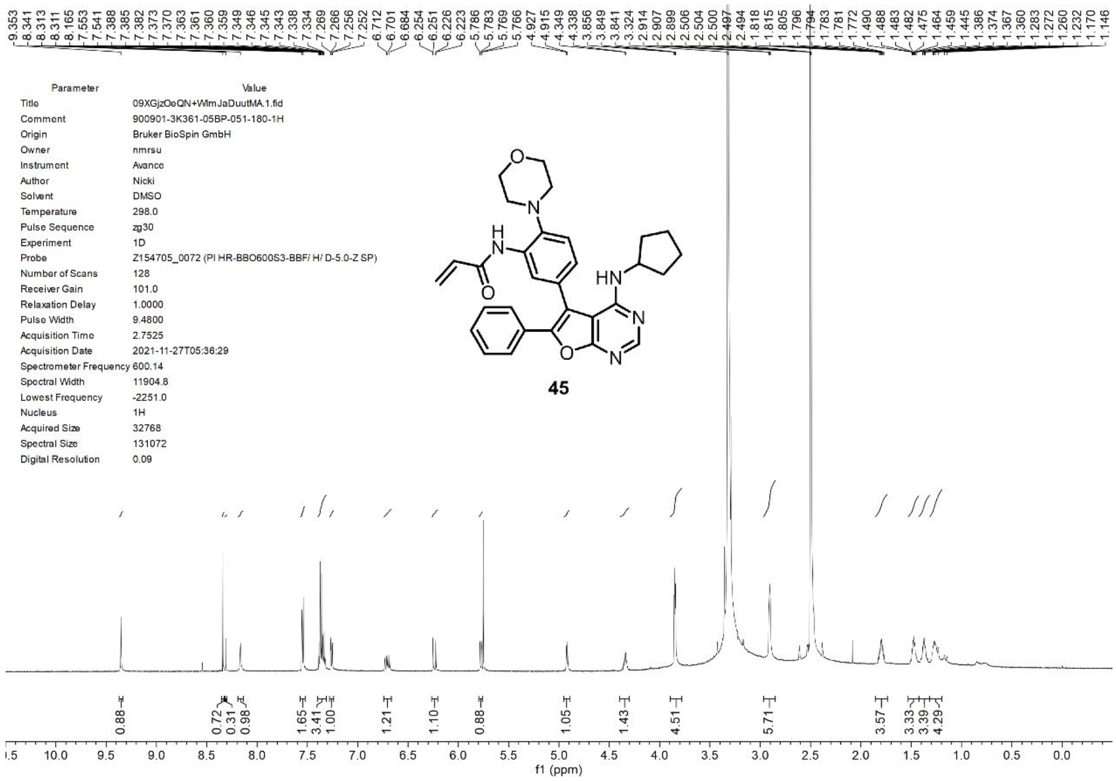
9.353 8.341 8.313 8.311 8.165 7.553 7.541 7.388 7.385 7.382 7.373 7.370 7.363 7.361 7.360 7.359 7.349 7.346 7.345 7.343 7.338 7.334 7.269 7.266 7.256 7.252 6.712 6.701 6.684 6.254 6.251 6.226 6.223 5.786 5.783 5.769 5.766 4.927 4.915 4.349 4.338 3.856 3.849 3.841 3.324 2.914 2.907 2.899 2.506 2.504 2.500 2.497 2.494 1.818 1.815 1.805 1.796 1.794 1.783 1.781 1.772 1.490 1.488 1.483 1.482 1.475 1.464 1.459 1.445 1.386 1.374 1.367 1.360 1.283 1.272 1.260 1.232 1.170 1.146
Parameter Value
Title 09XGjzOeQN+WImJaDuutMA.1.fid
Comment 900901-3K361-05BP-051-180-1H
Origin Bruker BioSpin GmbH
Owner nmrsu
Instrument Avance
Author Nicki
Solvent DMSO
Temperature 298.0
Pulse Sequence zg30
Experiment 1D
Probe Z154705_0072 (PI HR-BBO600S3-BBF/ H/ D-5.0-Z SP)
Number of Scans 128
Receiver Gain 101.0
Relaxation Delay 1.0000
Pulse Width 9.4800
Acquisition Time 2.7525
Acquisition Date 2021-11-27T05:36:29
Spectrometer Frequency 600.14
Spectral Width 11904.8
Lowest Frequency -2251.0
Nucleus 1H
Acquired Size 32768
Spectral Size 131072
Digital Resolution 0.09
45
0.88 0.72 0.31 0.98 1.65 3.41 1.00 1.21 1.10 0.88 1.05 1.43 4.51 5.71 3.57 3.33 3.39 4.29
10.0 9.5 9.0 8.5 8.0 7.5 7.0 6.5 6.0 5.5 5.0 4.5 4.0 3.5 3.0 2.5 2.0 1.5 1.0 0.5 0.0
f1 (ppm)

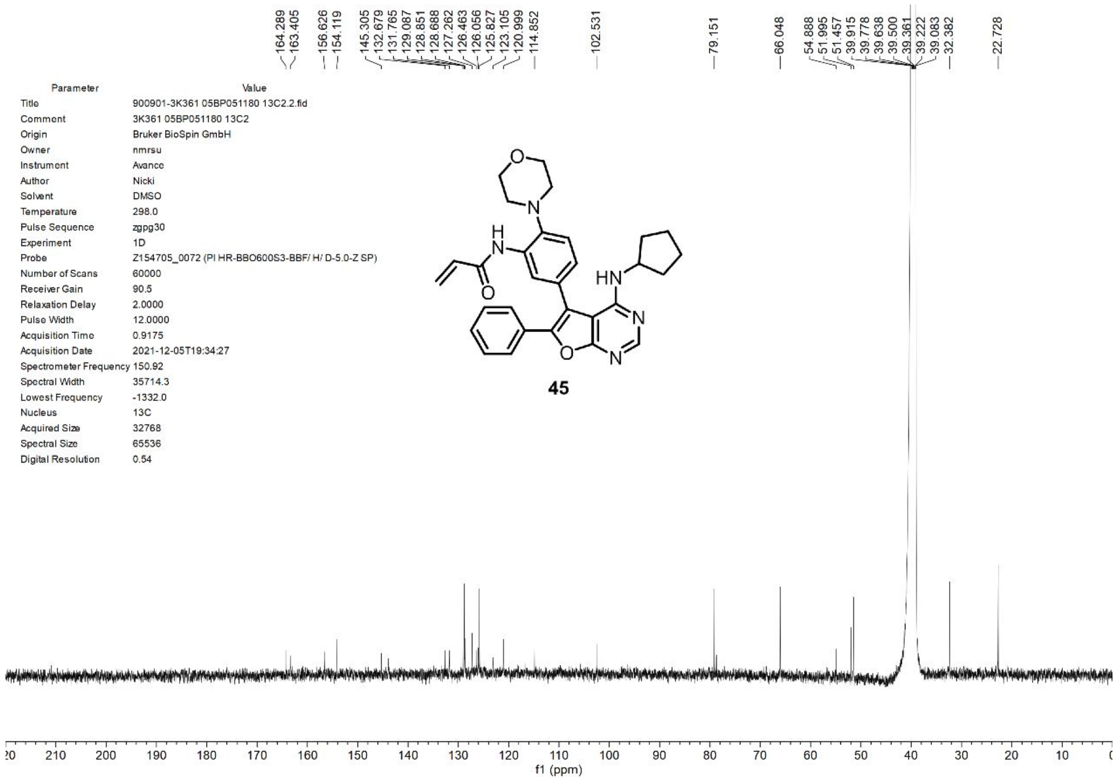
164.289 163.405 156.626 154.119 145.305 132.679 131.765 129.087 128.851 128.688 127.262 126.463 126.056 125.827 123.105 120.999 114.852 102.531 79.151 66.048 54.888 51.995 51.457 39.915 39.778 39.638 39.500 39.361 39.222 39.083 32.382 22.728
Parameter Value
Title 900901-3K361 05BP051180 13C2.2.fid
Comment 3K361 05BP051180 13C2
Origin Bruker BioSpin GmbH
Owner nmrsu
Instrument Avance
Author Nicki
Solvent DMSO
Temperature 298.0
Pulse Sequence zgpg30
Experiment 1D
Probe Z154705_0072 (PI HR-BBO600S3-BBF/ H/ D-5.0-Z SP)
Number of Scans 60000
Receiver Gain 90.5
Relaxation Delay 2.0000
Pulse Width 12.0000
Acquisition Time 0.9175
Acquisition Date 2021-12-05T19:34:27
Spectrometer Frequency 150.92
Spectral Width 35714.3
Lowest Frequency -1332.0
Nucleus 13C
Acquired Size 32768
Spectral Size 65536
Digital Resolution 0.54
45
210 200 190 180 170 160 150 140 130 120 110 100 90 80 70 60 50 40 30 20 10
f1 (ppm)

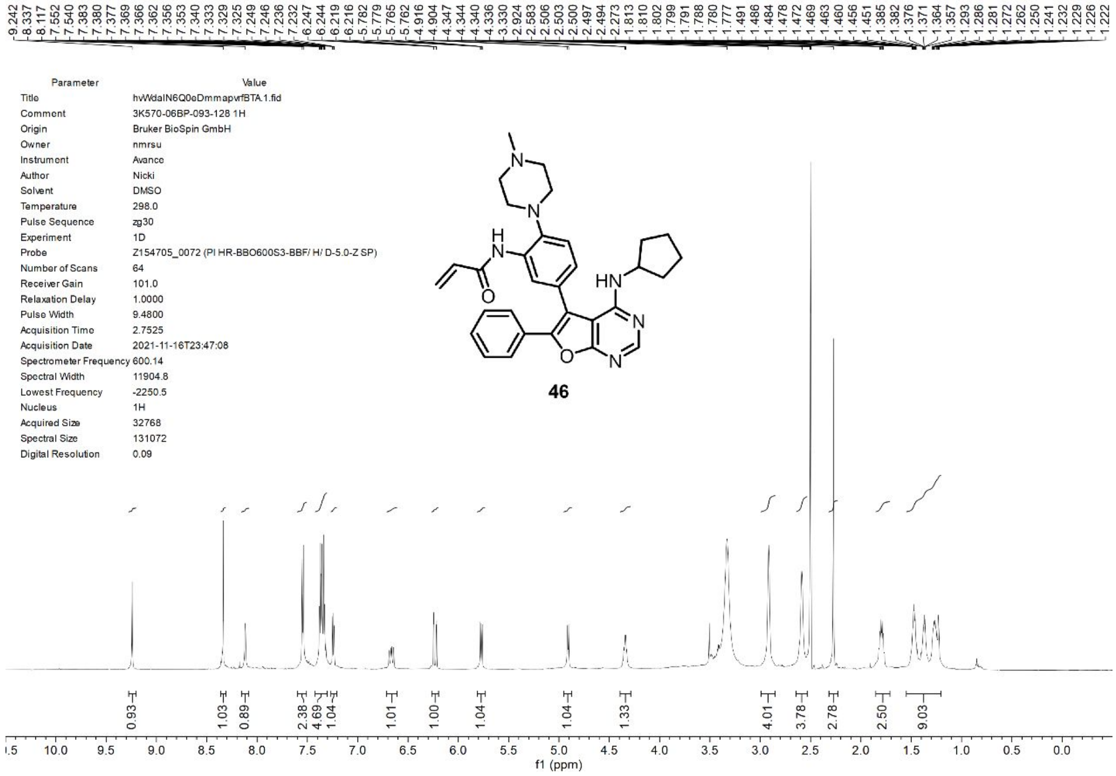
9.242 8.337 8.117 7.552 7.540 7.383 7.380 7.377 7.369 7.366 7.362 7.356 7.353 7.340 7.333 7.329 7.325 7.249 7.246 7.236 7.232 6.247 6.244 6.219 6.216 5.782 5.779 5.765 5.762 4.916 4.904 4.347 4.344 4.340 4.336 3.330 2.924 2.583 2.506 2.503 2.500 2.497 2.494 2.273 1.813 1.810 1.802 1.799 1.791 1.788 1.780 1.777 1.491 1.486 1.484 1.478 1.472 1.469 1.463 1.460 1.456 1.451 1.385 1.382 1.376 1.371 1.364 1.357 1.293 1.286 1.281 1.272 1.262 1.250 1.241 1.232 1.229 1.226 1.222
Parameter Value
Title hvWdalN6Q0oDmmapvrfBTA.1.fid
Comment 3K570-06BP-093-128 1H
Origin Bruker BioSpin GmbH
Owner nmrsu
Instrument Avance
Author Nicki
Solvent DMSO
Temperature 298.0
Pulse Sequence zg30
Experiment 1D
Probe Z154705_0072 (PI HR-BBO600S3-BBF/ H/ D-5.0-Z SP)
Number of Scans 64
Receiver Gain 101.0
Relaxation Delay 1.0000
Pulse Width 9.4800
Acquisition Time 2.7525
Acquisition Date 2021-11-16T23:47:08
Spectrometer Frequency 600.14
Spectral Width 11904.8
Lowest Frequency -2250.5
Nucleus 1H
Acquired Size 32768
Spectral Size 131072
Digital Resolution 0.09
46
0.93 1.03 0.89 2.38 4.69 1.04 1.01 1.00 1.04 1.04 1.33 4.01 3.78 2.78 2.50 9.03
f1 (ppm)

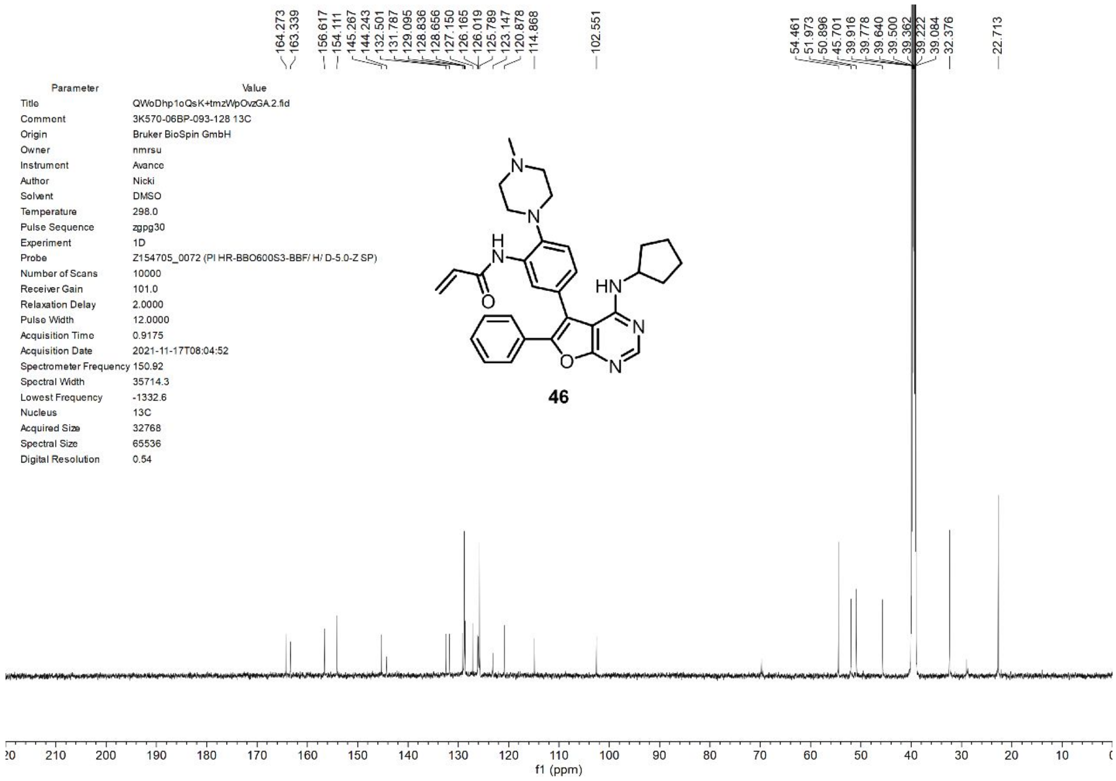
164.273 163.339 156.617 154.111 145.267 144.243 132.501 131.787 129.095 128.836 128.656 127.150 126.165 126.019 125.789 123.147 120.878 114.868 102.551 54.461 51.973 50.896 45.701 39.916 39.778 39.640 39.500 39.362 39.222 39.084 32.376 22.713
Parameter Value
Title QWoDhp1oQsK+tmzWpOvzGA.2.fid
Comment 3K570-06BP-093-128 13C
Origin Bruker BioSpin GmbH
Owner nmrsu
Instrument Avance
Author Nicki
Solvent DMSO
Temperature 298.0
Pulse Sequence zgpg30
Experiment 1D
Probe Z154705_0072 (PI HR-BBO600S3-BBF/ H/ D-5.0-Z SP)
Number of Scans 10000
Receiver Gain 101.0
Relaxation Delay 2.0000
Pulse Width 12.0000
Acquisition Time 0.9175
Acquisition Date 2021-11-17T08:04:52
Spectrometer Frequency 150.92
Spectral Width 35714.3
Lowest Frequency -1332.6
Nucleus 13C
Acquired Size 32768
Spectral Size 65536
Digital Resolution 0.54
46
f1 (ppm)

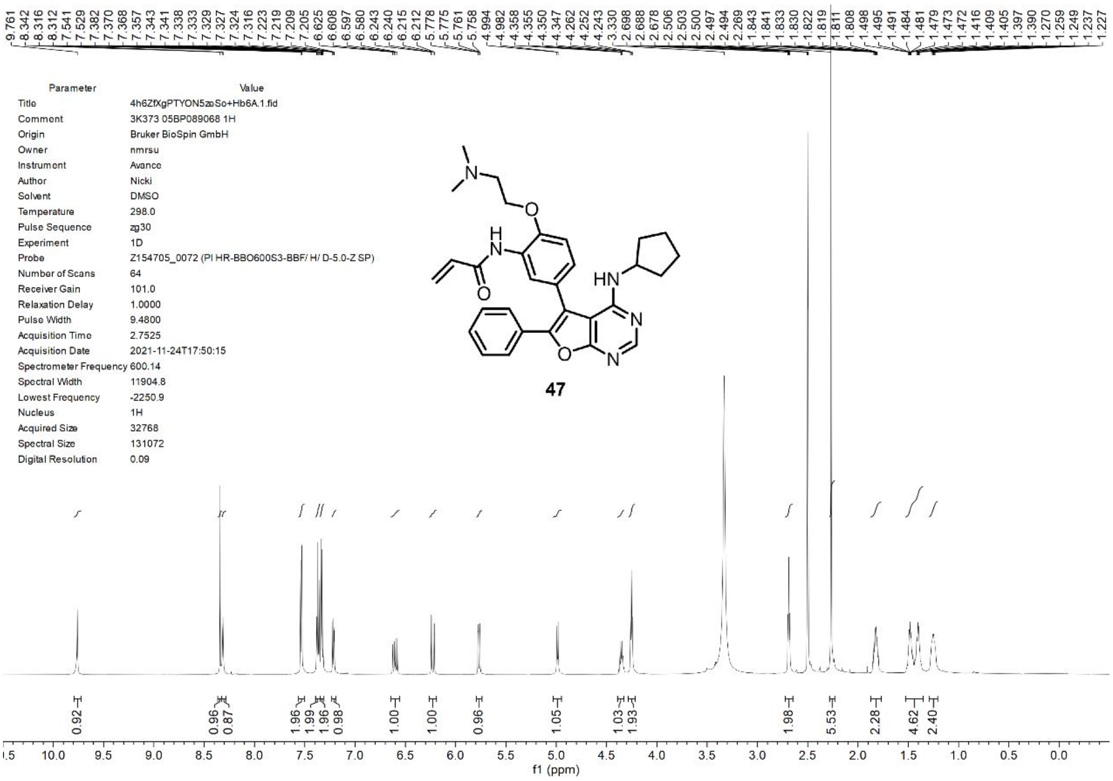
Parameter Value
Title 4h6ZfXgPTYON5zoSo+Hb6A.1.fid
Comment 3K373 05BP089068 1H
Origin Bruker BioSpin GmbH
Owner nmrsu
Instrument Avance
Author Nicki
Solvent DMSO
Temperature 298.0
Pulse Sequence zg30
Experiment 1D
Probe Z154705_0072 (PI HR-BBO600S3-BBF/ H/ D-5.0-Z SP)
Number of Scans 64
Receiver Gain 101.0
Relaxation Delay 1.0000
Pulse Width 9.4800
Acquisition Time 2.7525
Acquisition Date 2021-11-24T17:50:15
Spectrometer Frequency 600.14
Spectral Width 11904.8
Lowest Frequency -2250.9
Nucleus 1H
Acquired Size 32768
Spectral Size 131072
Digital Resolution 0.09
47
f1 (ppm)

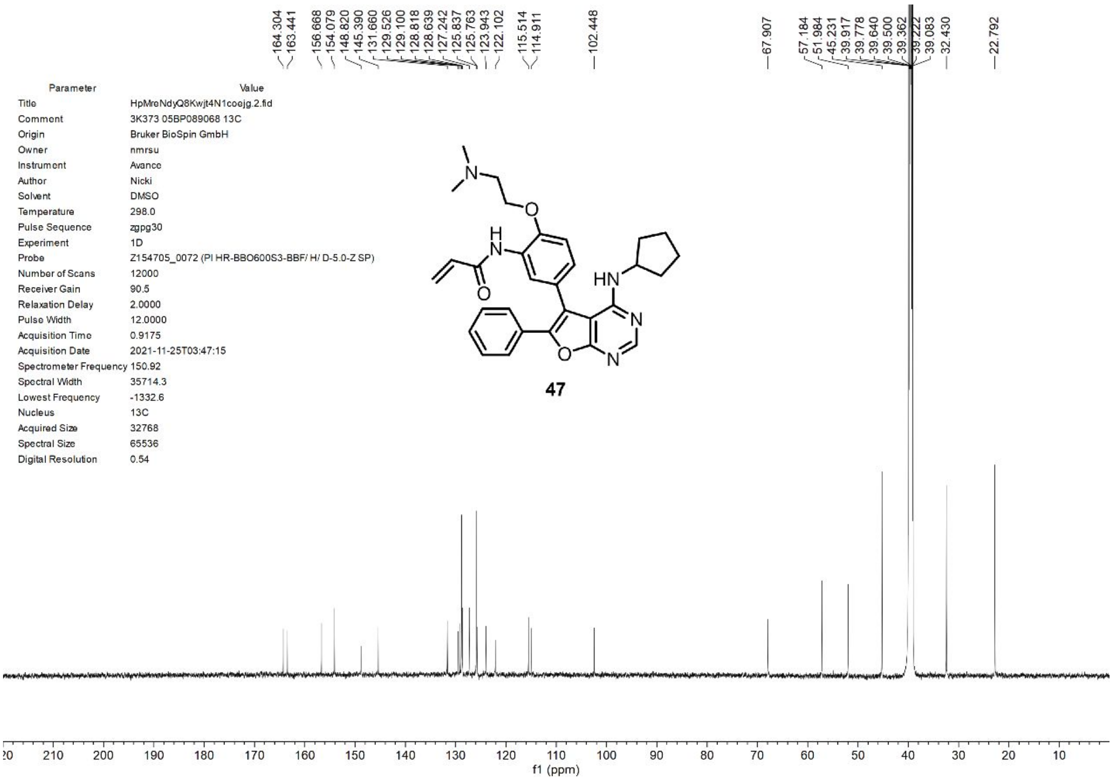
Parameter Value
Title HpMroNdyQ8Kwjt4N1coojg.2.fid
Comment 3K373 05BP089068 13C
Origin Bruker BioSpin GmbH
Owner nmrsu
Instrument Avance
Author Nicki
Solvent DMSO
Temperature 298.0
Pulse Sequence zgpg30
Experiment 1D
Probe Z154705_0072 (PI HR-BBO600S3-BBF/ H/ D-5.0-Z SP)
Number of Scans 12000
Receiver Gain 90.5
Relaxation Delay 2.0000
Pulse Width 12.0000
Acquisition Time 0.9175
Acquisition Date 2021-11-25T03:47:15
Spectrometer Frequency 150.92
Spectral Width 35714.3
Lowest Frequency -1332.6
Nucleus 13C
Acquired Size 32768
Spectral Size 65536
Digital Resolution 0.54
47
f1 (ppm)

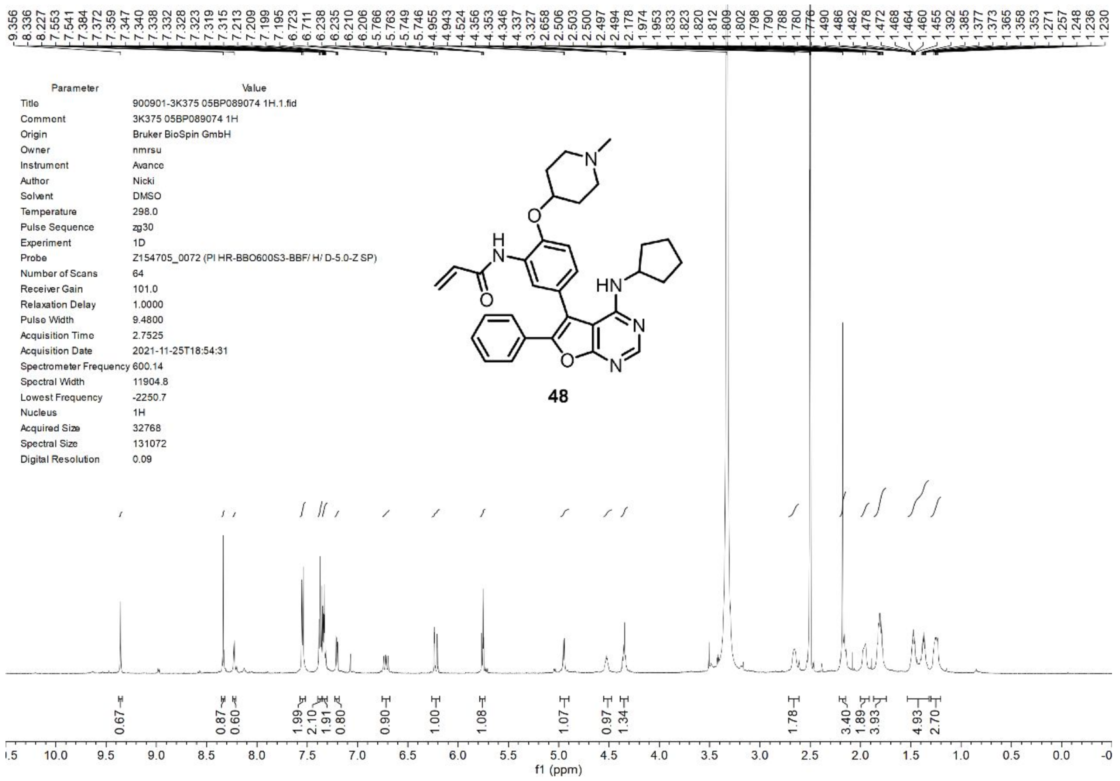
Parameter Value
Title 900901-3K375 05BP089074 1H.1.fid
Comment 3K375 05BP089074 1H
Origin Bruker BioSpin GmbH
Owner nmrsu
Instrument Avance
Author Nicki
Solvent DMSO
Temperature 298.0
Pulse Sequence zg30
Experiment 1D
Probe Z154705_0072 (PI HR-BBO600S3-BBF/ H/ D-5.0-Z SP)
Number of Scans 64
Receiver Gain 101.0
Relaxation Delay 1.0000
Pulse Width 9.4800
Acquisition Time 2.7525
Acquisition Date 2021-11-25T18:54:31
Spectrometer Frequency 600.14
Spectral Width 11904.8
Lowest Frequency -2250.7
Nucleus 1H
Acquired Size 32768
Spectral Size 131072
Digital Resolution 0.09
48
f1 (ppm)

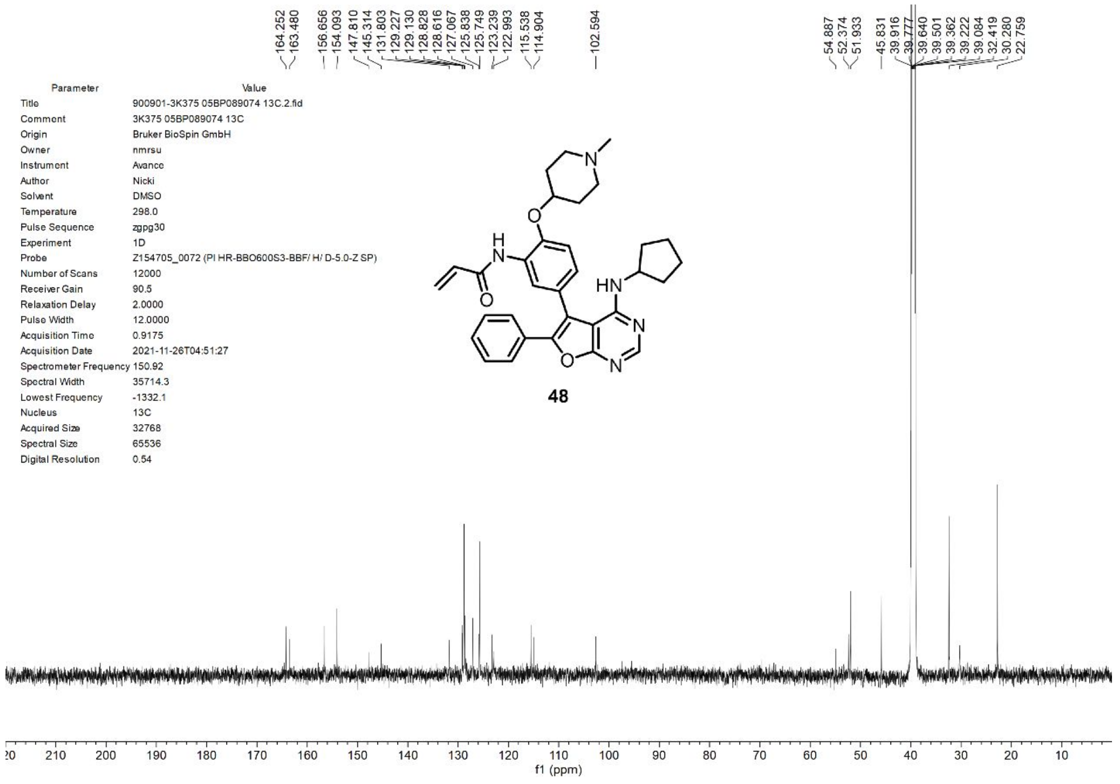
Parameter Value
Title 900901-3K375 05BP089074 13C.2.fid
Comment 3K375 05BP089074 13C
Origin Bruker BioSpin GmbH
Owner nmrsu
Instrument Avance
Author Nicki
Solvent DMSO
Temperature 298.0
Pulse Sequence zgpg30
Experiment 1D
Probe Z154705_0072 (PI HR-BBO600S3-BBF/ H/ D-5.0-Z SP)
Number of Scans 12000
Receiver Gain 90.5
Relaxation Delay 2.0000
Pulse Width 12.0000
Acquisition Time 0.9175
Acquisition Date 2021-11-26T04:51:27
Spectrometer Frequency 150.92
Spectral Width 35714.3
Lowest Frequency -1332.1
Nucleus 13C
Acquired Size 32768
Spectral Size 65536
Digital Resolution 0.54
48
f1 (ppm)

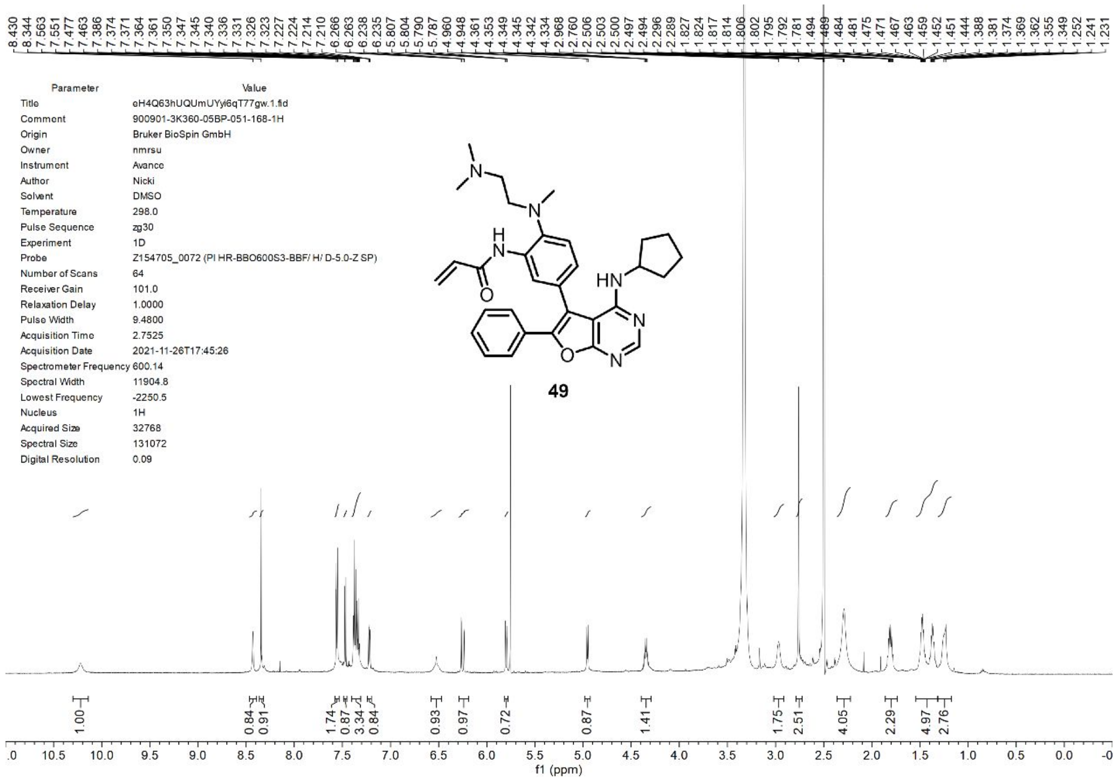
8.430 8.344 7.563 7.551 7.477 7.463 7.386 7.374 7.371 7.364 7.361 7.350 7.347 7.345 7.340 7.336 7.331 7.326 7.323 7.227 7.224 7.214 7.210 6.266 6.263 6.238 6.235 5.807 5.804 5.790 5.787 4.960 4.948 4.361 4.353 4.349 4.345 4.342 4.334 2.968 2.760 2.506 2.503 2.500 2.497 2.494 2.296 2.289 1.827 1.824 1.817 1.814 1.806 1.802 1.795 1.792 1.781 1.494 1.489 1.484 1.481 1.475 1.471 1.467 1.463 1.459 1.452 1.451 1.444 1.388 1.381 1.374 1.369 1.362 1.355 1.349 1.252 1.241 1.231
Parameter Value
Title eH4Q63hUQUmUYyi6qT77gw.1.fid
Comment 900901-3K360-05BP-051-168-1H
Origin Bruker BioSpin GmbH
Owner nmrsu
Instrument Avance
Author Nicki
Solvent DMSO
Temperature 298.0
Pulse Sequence zg30
Experiment 1D
Probe Z154705_0072 (PI HR-BBO600S3-BBF/ H/ D-5.0-Z SP)
Number of Scans 64
Receiver Gain 101.0
Relaxation Delay 1.0000
Pulse Width 9.4800
Acquisition Time 2.7525
Acquisition Date 2021-11-26T17:45:26
Spectrometer Frequency 600.14
Spectral Width 11904.8
Lowest Frequency -2250.5
Nucleus 1H
Acquired Size 32768
Spectral Size 131072
Digital Resolution 0.09
49
1.00 0.84 0.91 1.74 0.87 3.34 0.84 0.93 0.97 0.72 0.87 1.41 1.75 2.51 4.05 2.29 4.97 2.76
f1 (ppm)

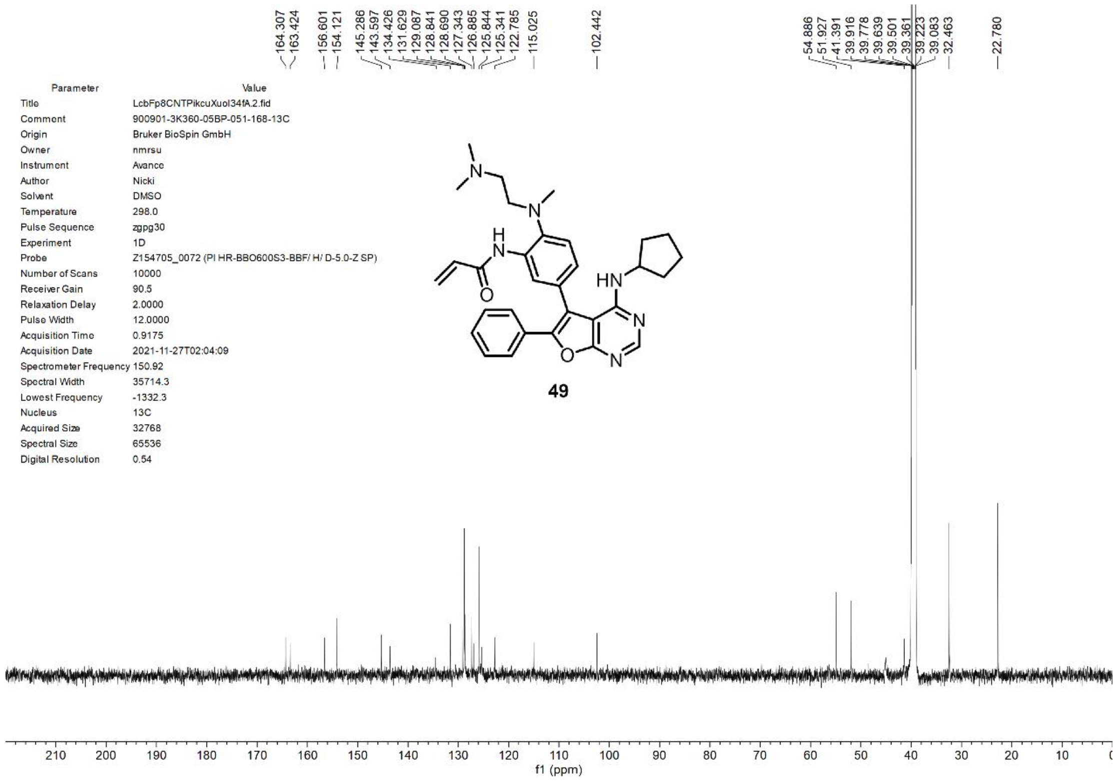
164.307 163.424 156.601 154.121 145.286 143.597 134.426 131.629 129.087 128.841 128.690 127.343 126.885 125.844 125.341 122.785 115.025 102.442 54.886 51.927 41.391 39.916 39.778 39.639 39.501 39.361 39.223 39.083 32.463 22.780
Parameter Value
Title LcbFp8CNTPikcuXuol34fA.2.fid
Comment 900901-3K360-05BP-051-168-13C
Origin Bruker BioSpin GmbH
Owner nmrsu
Instrument Avance
Author Nicki
Solvent DMSO
Temperature 298.0
Pulse Sequence zgpg30
Experiment 1D
Probe Z154705_0072 (PI HR-BBO600S3-BBF/ H/ D-5.0-Z SP)
Number of Scans 10000
Receiver Gain 90.5
Relaxation Delay 2.0000
Pulse Width 12.0000
Acquisition Time 0.9175
Acquisition Date 2021-11-27T02:04:09
Spectrometer Frequency 150.92
Spectral Width 35714.3
Lowest Frequency -1332.3
Nucleus 13C
Acquired Size 32768
Spectral Size 65536
Digital Resolution 0.54
49
f1 (ppm)

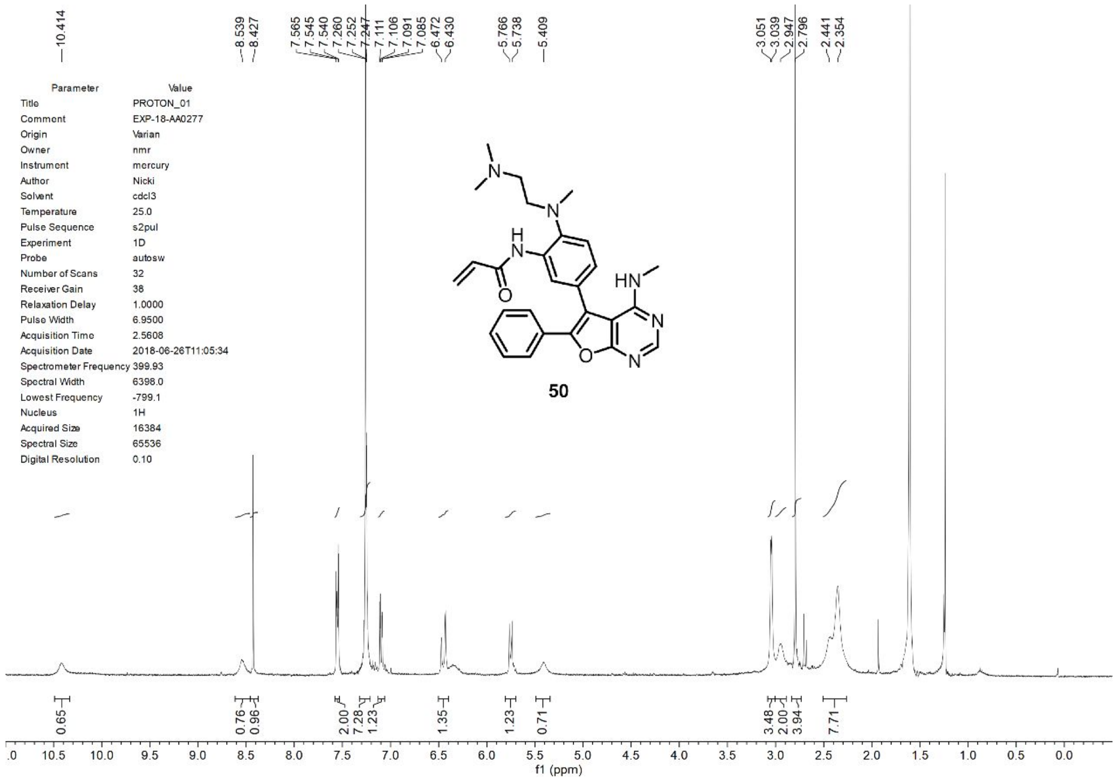

10.414
8.539
8.427
7.565
7.545
7.540
7.260
7.252
7.247
7.111
7.106
7.091
7.085
6.472
6.430
5.766
5.738
5.409
3.051
3.039
2.947
2.796
2.441
2.354
Parameter Value
Title PROTON_01
Comment EXP-18-AA0277
Origin Varian
Owner nmr
Instrument mercury
Author Nicki
Solvent cdcl3
Temperature 25.0
Pulse Sequence s2pul
Experiment 1D
Probe autosw
Number of Scans 32
Receiver Gain 38
Relaxation Delay 1.0000
Pulse Width 6.9500
Acquisition Time 2.5608
Acquisition Date 2018-06-26T11:05:34
Spectrometer Frequency 399.93
Spectral Width 6398.0
Lowest Frequency -799.1
Nucleus 1H
Acquired Size 16384
Spectral Size 65536
Digital Resolution 0.10
HN
N
O
50
0.65
0.76
0.96
2.00
7.28
1.23
1.35
1.23
0.71
3.48
2.00
3.94
7.71
f1 (ppm)

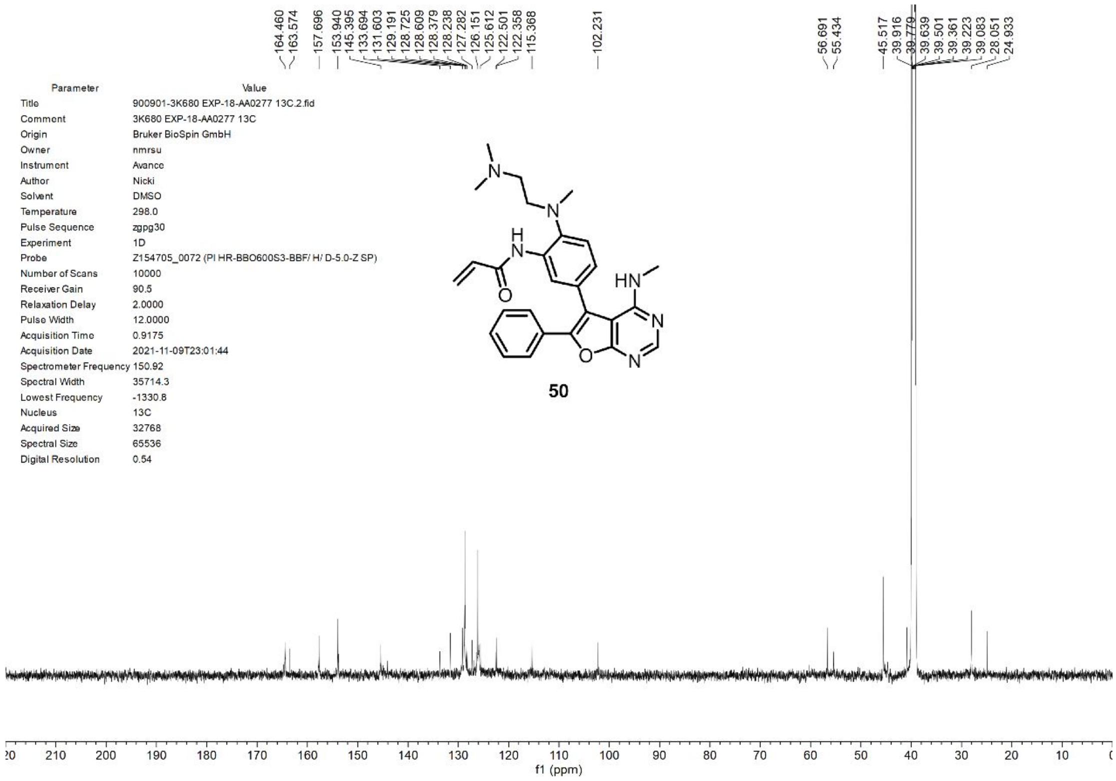

164.460
163.574
157.696
153.940
145.395
133.694
131.603
129.191
128.725
128.609
128.379
128.238
127.282
126.151
125.612
122.501
122.358
115.368
102.231
56.691
55.434
45.517
39.916
39.779
39.639
39.501
39.361
39.223
39.083
28.051
24.933
Parameter Value
Title 900901-3K680 EXP-18-AA0277 13C.2.fid
Comment 3K680 EXP-18-AA0277 13C
Origin Bruker BioSpin GmbH
Owner nmrsu
Instrument Avance
Author Nicki
Solvent DMSO
Temperature 298.0
Pulse Sequence zgpg30
Experiment 1D
Probe Z154705_0072 (PI HR-BBO600S3-BBF/ H/ D-5.0-Z SP)
Number of Scans 10000
Receiver Gain 90.5
Relaxation Delay 2.0000
Pulse Width 12.0000
Acquisition Time 0.9175
Acquisition Date 2021-11-09T23:01:44
Spectrometer Frequency 150.92
Spectral Width 35714.3
Lowest Frequency -1330.8
Nucleus 13C
Acquired Size 32768
Spectral Size 65536
Digital Resolution 0.54
HN
N
O
50
f1 (ppm)

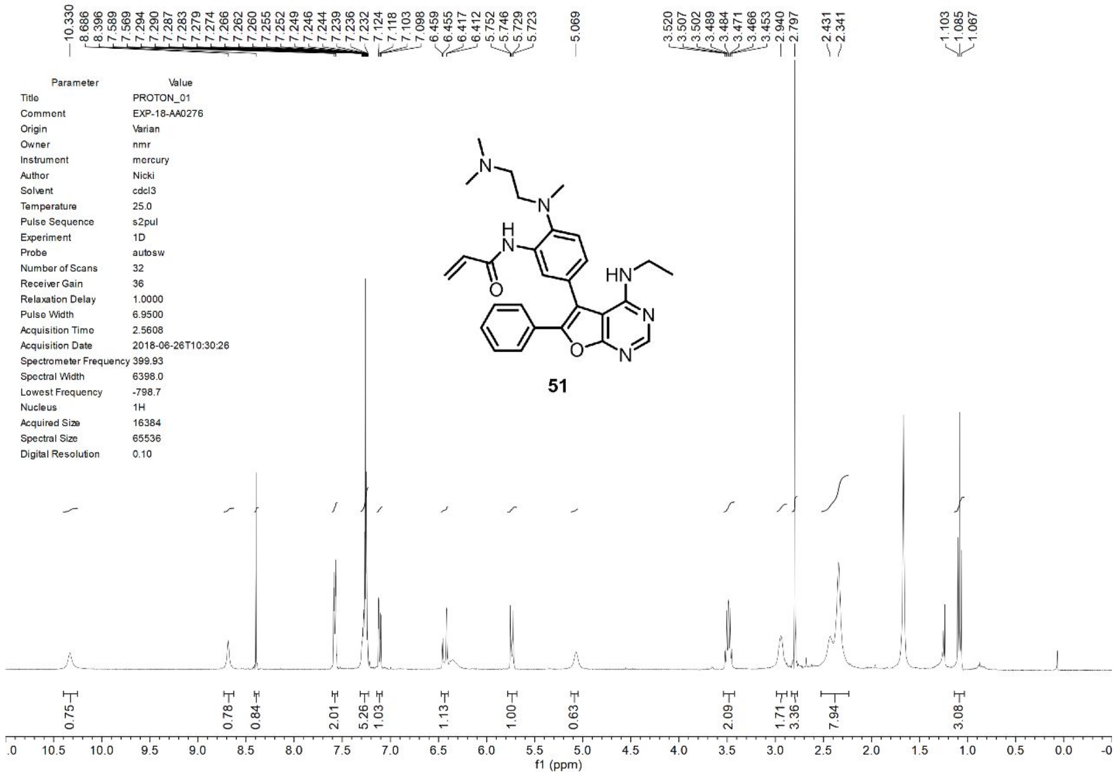
10.330
8.686
8.396
7.589
7.569
7.294
7.290
7.287
7.283
7.279
7.274
7.266
7.262
7.260
7.255
7.252
7.249
7.246
7.244
7.239
7.236
7.232
7.124
7.118
7.103
7.098
6.459
6.455
6.417
6.412
5.752
5.746
5.729
5.723
5.069
3.520
3.507
3.502
3.489
3.484
3.471
3.466
3.453
2.940
2.797
2.431
2.341
1.103
1.085
1.067
Parameter Value
Title PROTON_01
Comment EXP-18-AA0276
Origin Varian
Owner nmr
Instrument mercury
Author Nicki
Solvent cdcl3
Temperature 25.0
Pulse Sequence s2pul
Experiment 1D
Probe autosw
Number of Scans 32
Receiver Gain 36
Relaxation Delay 1.0000
Pulse Width 6.9500
Acquisition Time 2.5608
Acquisition Date 2018-06-26T10:30:26
Spectrometer Frequency 399.93
Spectral Width 6398.0
Lowest Frequency -798.7
Nucleus 1H
Acquired Size 16384
Spectral Size 65536
Digital Resolution 0.10
HN
N
O
N
N
H
N
O
51
0.75
0.78
0.84
2.01
5.26
1.03
1.13
1.00
0.63
2.09
1.71
3.36
7.94
3.08
f1 (ppm)

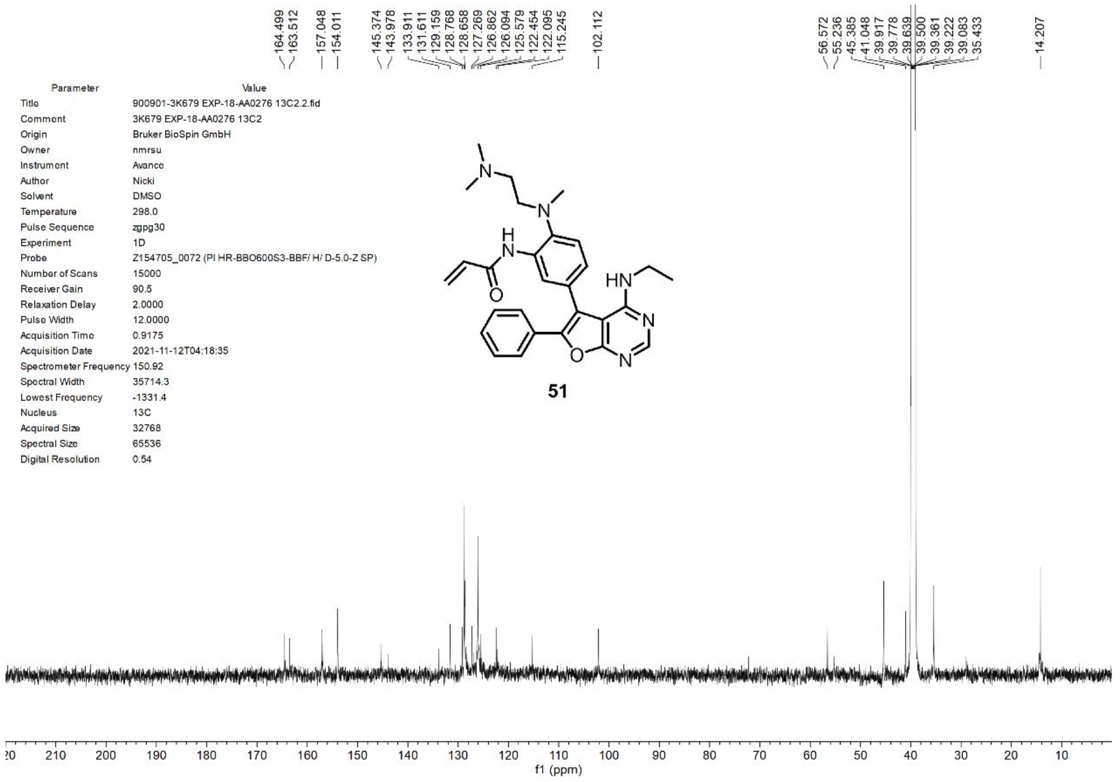
164.499
163.512
157.048
154.011
145.374
143.978
133.911
131.611
129.159
128.768
128.658
127.269
126.862
126.094
125.579
122.454
122.095
115.245
102.112
56.572
55.236
45.385
41.048
39.917
39.778
39.639
39.500
39.361
39.222
39.083
35.433
14.207
Parameter Value
Title 900901-3K679 EXP-18-AA0276 13C2.2.fid
Comment 3K679 EXP-18-AA0276 13C2
Origin Bruker BioSpin GmbH
Owner nmrsu
Instrument Avance
Author Nicki
Solvent DMSO
Temperature 298.0
Pulse Sequence zgpg30
Experiment 1D
Probe Z154705_0072 (PI HR-BBO600S3-BBF/ H/ D-5.0-Z SP)
Number of Scans 15000
Receiver Gain 90.5
Relaxation Delay 2.0000
Pulse Width 12.0000
Acquisition Time 0.9175
Acquisition Date 2021-11-12T04:18:35
Spectrometer Frequency 150.92
Spectral Width 35714.3
Lowest Frequency -1331.4
Nucleus 13C
Acquired Size 32768
Spectral Size 65536
Digital Resolution 0.54
HN
N
O
N
N
H
N
O
51
f1 (ppm)

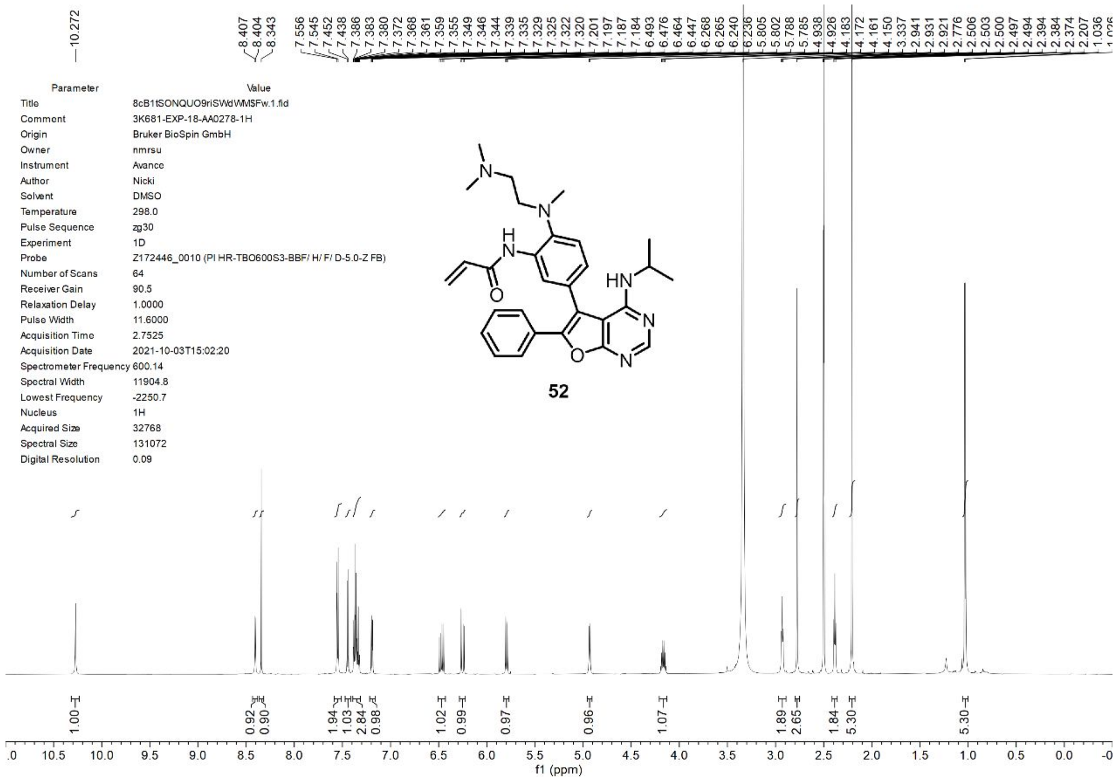

Parameter	Value
Title	8cB1tSONQUO9riSWdWMSFw.1.fid
Comment	3K681-EXP-18-AA0278-1H
Origin	Bruker BioSpin GmbH
Owner	nmrsu
Instrument	Avance
Author	Nicki
Solvent	DMSO
Temperature	298.0
Pulse Sequence	zg30
Experiment	1D
Probe	Z172446_0010 (PI HR-TBO600S3-BBF/ H/ F/ D-5.0-Z FB)
Number of Scans	64
Receiver Gain	90.5
Relaxation Delay	1.0000
Pulse Width	11.6000
Acquisition Time	2.7525
Acquisition Date	2021-10-03T15:02:20
Spectrometer Frequency	600.14
Spectral Width	11904.8
Lowest Frequency	-2250.7
Nucleus	1H
Acquired Size	32768
Spectral Size	131072
Digital Resolution	0.09

52

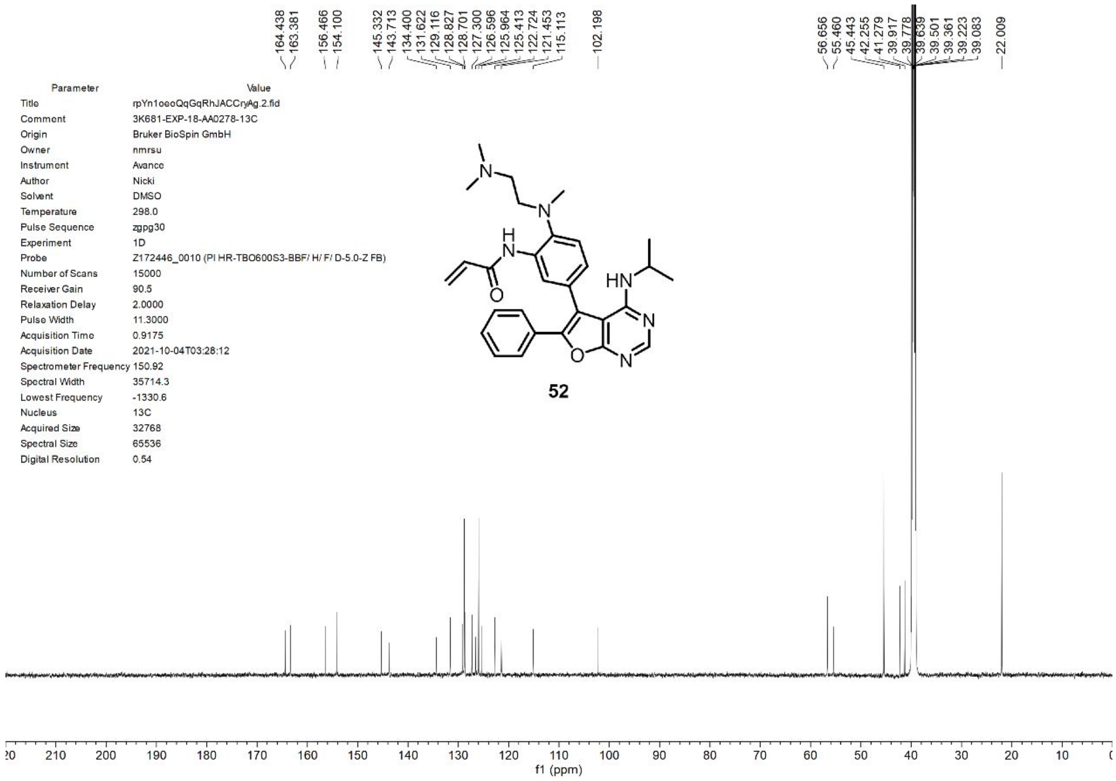

Parameter	Value
Title	rpYn1oeoQqGqRhJACCryAg.2.fid
Comment	3K681-EXP-18-AA0278-13C
Origin	Bruker BioSpin GmbH
Owner	nmrsu
Instrument	Avance
Author	Nicki
Solvent	DMSO
Temperature	298.0
Pulse Sequence	zgpg30
Experiment	1D
Probe	Z172446_0010 (PI HR-TBO600S3-BBF/ H/ F/ D-5.0-Z FB)
Number of Scans	15000
Receiver Gain	90.5
Relaxation Delay	2.0000
Pulse Width	11.3000
Acquisition Time	0.9175
Acquisition Date	2021-10-04T03:28:12
Spectrometer Frequency	150.92
Spectral Width	35714.3
Lowest Frequency	-1330.6
Nucleus	13C
Acquired Size	32768
Spectral Size	65536
Digital Resolution	0.54

52

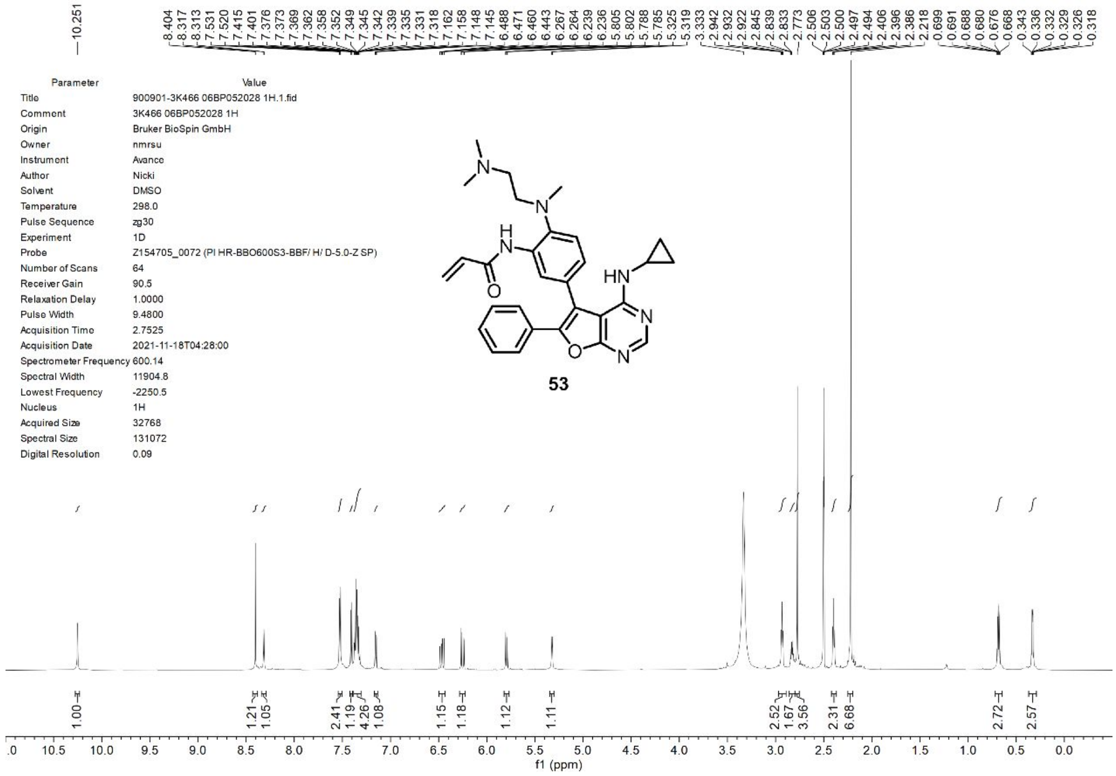
10.251
8.404 8.317 8.313 7.531 7.520 7.415 7.401 7.376 7.373 7.369 7.362 7.358 7.352 7.349 7.345 7.342 7.339 7.335 7.331 7.318 7.162 7.158 7.148 7.145 6.488 6.471 6.460 6.443 6.267 6.264 6.239 6.236 5.805 5.802 5.788 5.785 5.325 5.319 3.333 2.942 2.932 2.922 2.845 2.839 2.833 2.773 2.506 2.503 2.500 2.497 2.494 2.406 2.396 2.386 2.218 0.699 0.691 0.688 0.680 0.676 0.668 0.343 0.336 0.332 0.329 0.326 0.318
Parameter | Value
Title | 900901-3K466 06BP052028 1H.1.fid
Comment | 3K466 06BP052028 1H
Origin | Bruker BioSpin GmbH
Owner | nmrsu
Instrument | Avance
Author | Nicki
Solvent | DMSO
Temperature | 298.0
Pulse Sequence | zg30
Experiment | 1D
Probe | Z154705_0072 (PI HR-BBO600S3-BBF/ H/ D-5.0-Z SP)
Number of Scans | 64
Receiver Gain | 90.5
Relaxation Delay | 1.0000
Pulse Width | 9.4800
Acquisition Time | 2.7525
Acquisition Date | 2021-11-18T04:28:00
Spectrometer Frequency | 600.14
Spectral Width | 11904.8
Lowest Frequency | -2250.5
Nucleus | 1H
Acquired Size | 32768
Spectral Size | 131072
Digital Resolution | 0.09
HN
N
N
O
H
N
O
N
N
53
1.00 1.21 1.05 2.41 1.19 4.26 1.08 1.15 1.18 1.12 1.11 2.52 1.67 3.56 2.31 6.68 2.72 2.57
f1 (ppm)

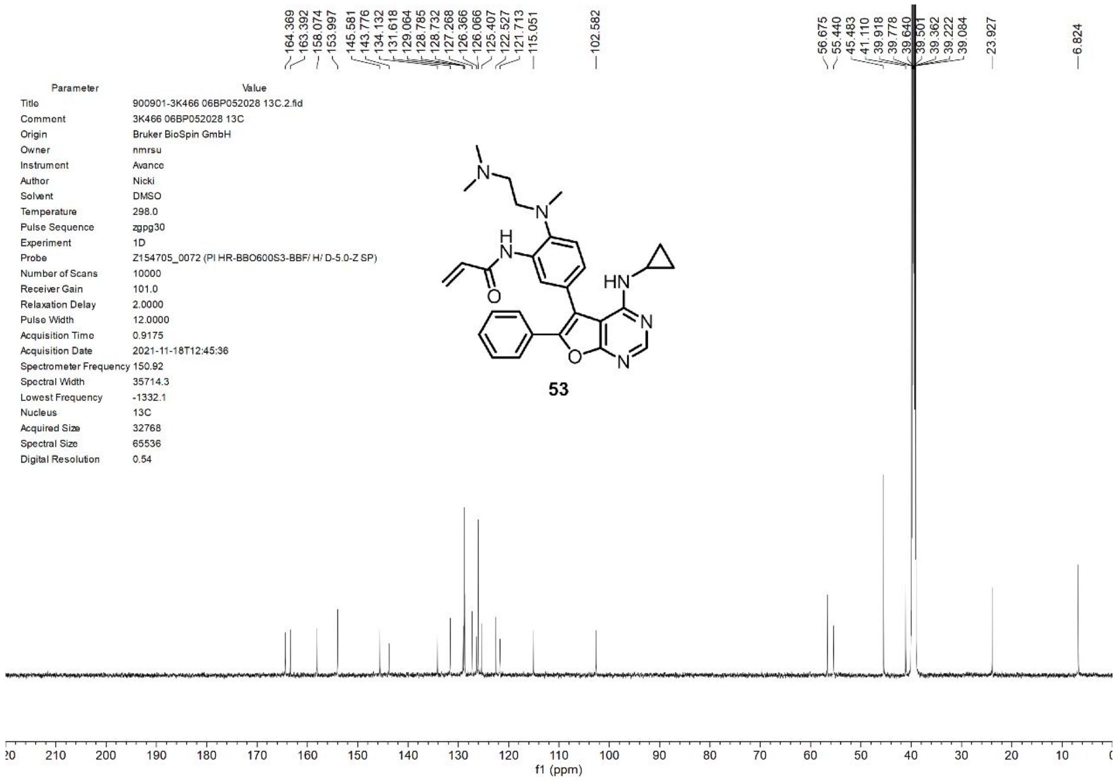
164.369 163.392 158.074 153.997 145.581 143.776 134.132 131.618 129.064 128.785 128.732 127.268 126.366 126.066 125.407 122.527 121.713 115.051
102.582
56.675 55.440 45.483 41.110 39.918 39.778 39.640 39.501 39.362 39.222 39.084
23.927
6.824
Parameter | Value
Title | 900901-3K466 06BP052028 13C.2.fid
Comment | 3K466 06BP052028 13C
Origin | Bruker BioSpin GmbH
Owner | nmrsu
Instrument | Avance
Author | Nicki
Solvent | DMSO
Temperature | 298.0
Pulse Sequence | zgpg30
Experiment | 1D
Probe | Z154705_0072 (PI HR-BBO600S3-BBF/ H/ D-5.0-Z SP)
Number of Scans | 10000
Receiver Gain | 101.0
Relaxation Delay | 2.0000
Pulse Width | 12.0000
Acquisition Time | 0.9175
Acquisition Date | 2021-11-18T12:45:36
Spectrometer Frequency | 150.92
Spectral Width | 35714.3
Lowest Frequency | -1332.1
Nucleus | 13C
Acquired Size | 32768
Spectral Size | 65536
Digital Resolution | 0.54
HN
N
N
O
H
N
O
N
N
53
f1 (ppm)

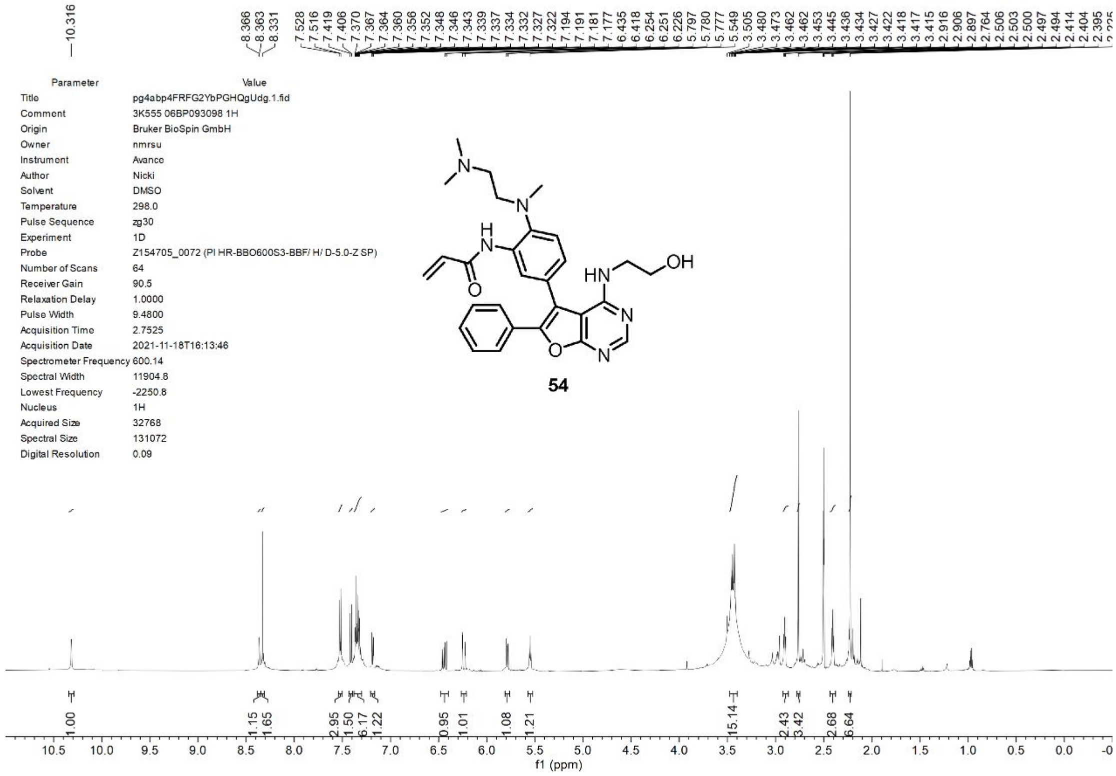
10.316
8.366 8.363 8.331 7.528 7.516 7.419 7.406 7.370 7.367 7.364 7.360 7.356 7.352 7.348 7.346 7.343 7.339 7.337 7.334 7.332 7.327 7.322 7.194 7.191 7.181 7.177 6.435 6.418 6.254 6.251 6.226 5.797 5.780 5.777 5.549 3.505 3.480 3.473 3.462 3.462 3.453 3.445 3.436 3.434 3.427 3.422 3.418 3.417 3.415 2.916 2.906 2.897 2.764 2.506 2.503 2.500 2.497 2.494 2.414 2.404 2.395
Parameter Value
Title pg4abp4FRFG2YbPGHQgUdg.1.fid
Comment 3K555 06BP093098 1H
Origin Bruker BioSpin GmbH
Owner nmrsu
Instrument Avance
Author Nicki
Solvent DMSO
Temperature 298.0
Pulse Sequence zg30
Experiment 1D
Probe Z154705_0072 (PI HR-BBO600S3-BBF/ H/ D-5.0-Z SP)
Number of Scans 64
Receiver Gain 90.5
Relaxation Delay 1.0000
Pulse Width 9.4800
Acquisition Time 2.7525
Acquisition Date 2021-11-18T16:13:46
Spectrometer Frequency 600.14
Spectral Width 11904.8
Lowest Frequency -2250.8
Nucleus 1H
Acquired Size 32768
Spectral Size 131072
Digital Resolution 0.09
HN OH
N
O
54
1.00 1.15 1.65 2.95 1.50 6.17 1.22 0.95 1.01 1.08 1.21 15.14 2.43 3.42 2.68 6.64
f1 (ppm)

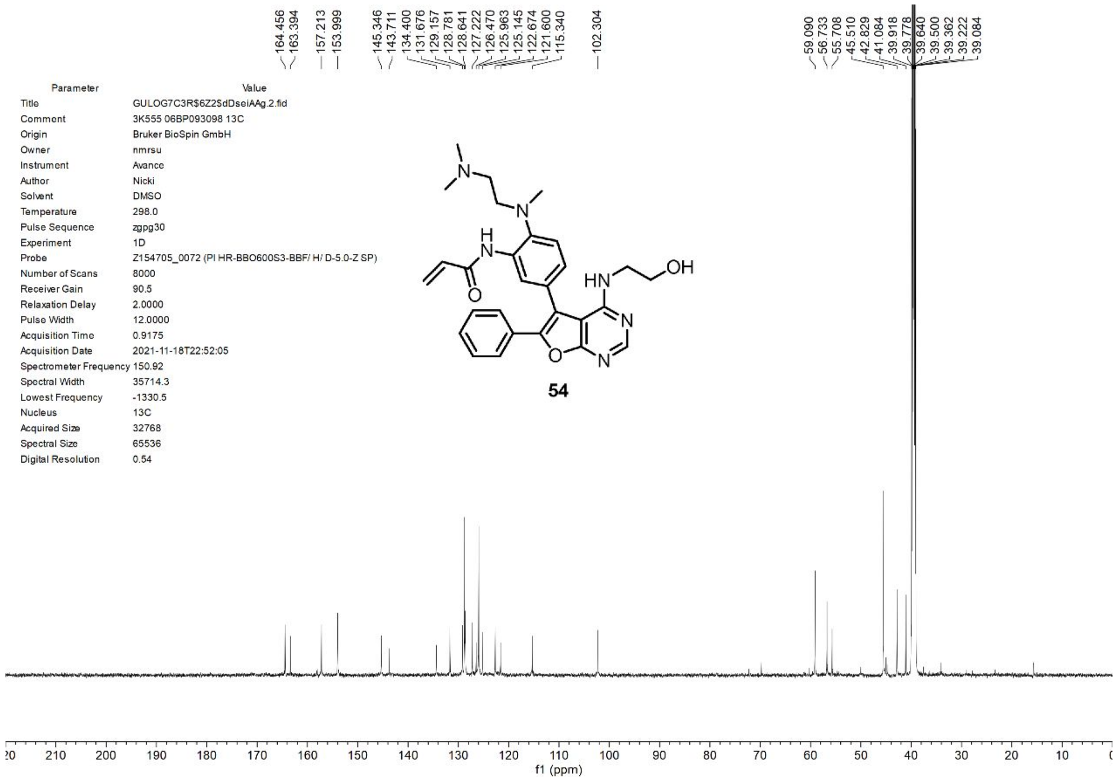
164.456 163.394 157.213 153.999 145.346 143.711 134.400 131.676 129.157 128.781 128.641 127.222 126.470 125.963 125.145 122.674 121.800 115.340 102.304
59.090 56.733 55.708 45.510 42.829 41.084 39.918 39.778 39.640 39.500 39.362 39.222 39.084
Parameter Value
Title GULOG7C3R$6Z2SdDsoiAAg.2.fid
Comment 3K555 06BP093098 13C
Origin Bruker BioSpin GmbH
Owner nmrsu
Instrument Avance
Author Nicki
Solvent DMSO
Temperature 298.0
Pulse Sequence zgpg30
Experiment 1D
Probe Z154705_0072 (PI HR-BBO600S3-BBF/ H/ D-5.0-Z SP)
Number of Scans 8000
Receiver Gain 90.5
Relaxation Delay 2.0000
Pulse Width 12.0000
Acquisition Time 0.9175
Acquisition Date 2021-11-18T22:52:05
Spectrometer Frequency 150.92
Spectral Width 35714.3
Lowest Frequency -1330.5
Nucleus 13C
Acquired Size 32768
Spectral Size 65536
Digital Resolution 0.54
HN OH
54
f1 (ppm)

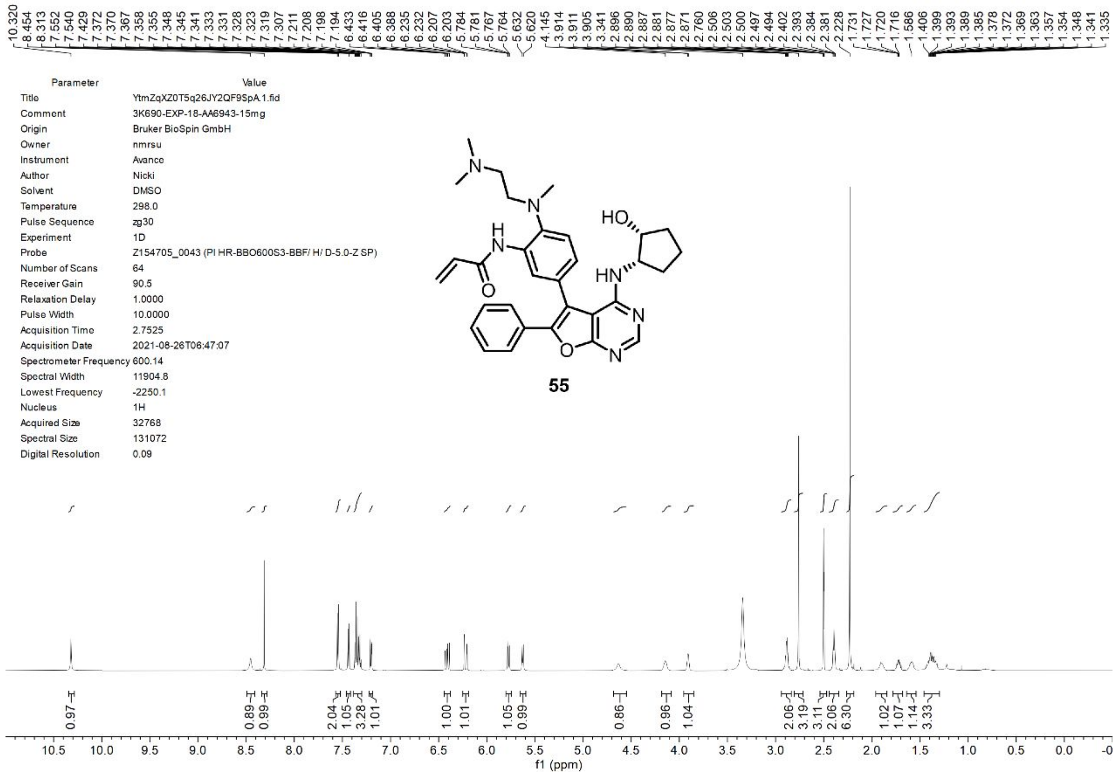
10.320 8.454 8.313 7.552 7.540 7.429 7.372 7.370 7.367 7.358 7.355 7.348 7.345 7.341 7.333 7.331 7.328 7.323 7.319 7.307 7.211 7.208 7.198 7.194 6.433 6.416 6.405 6.388 6.235 6.232 6.207 6.203 5.784 5.781 5.767 5.764 5.632 5.620 4.145 3.914 3.911 3.905 3.341 2.896 2.890 2.887 2.881 2.877 2.871 2.760 2.506 2.503 2.500 2.497 2.494 2.402 2.393 2.384 2.381 2.228 1.731 1.727 1.720 1.716 1.586 1.406 1.399 1.393 1.389 1.385 1.378 1.372 1.369 1.363 1.357 1.354 1.348 1.341 1.335
Parameter Value
Title YtmZqXZ0T5q26JY2QF9SpA.1.fid
Comment 3K690-EXP-18-AA6943-15mg
Origin Bruker BioSpin GmbH
Owner nmrsu
Instrument Avance
Author Nicki
Solvent DMSO
Temperature 298.0
Pulse Sequence zg30
Experiment 1D
Probe Z154705_0043 (PI HR-BBO600S3-BBF/ H/ D-5.0-Z SP)
Number of Scans 64
Receiver Gain 90.5
Relaxation Delay 1.0000
Pulse Width 10.0000
Acquisition Time 2.7525
Acquisition Date 2021-08-26T06:47:07
Spectrometer Frequency 600.14
Spectral Width 11904.8
Lowest Frequency -2250.1
Nucleus 1H
Acquired Size 32768
Spectral Size 131072
Digital Resolution 0.09
HO
HN
N
N
O
55
0.97 0.89 0.99 2.04 1.05 3.28 1.01 1.00 1.01 1.05 0.99 0.86 0.96 1.04 2.06 3.19 3.11 2.06 6.30 1.02 1.07 1.14 3.33
10.5 10.0 9.5 9.0 8.5 8.0 7.5 7.0 6.5 6.0 5.5 5.0 4.5 4.0 3.5 3.0 2.5 2.0 1.5 1.0 0.5 0.0
f1 (ppm)

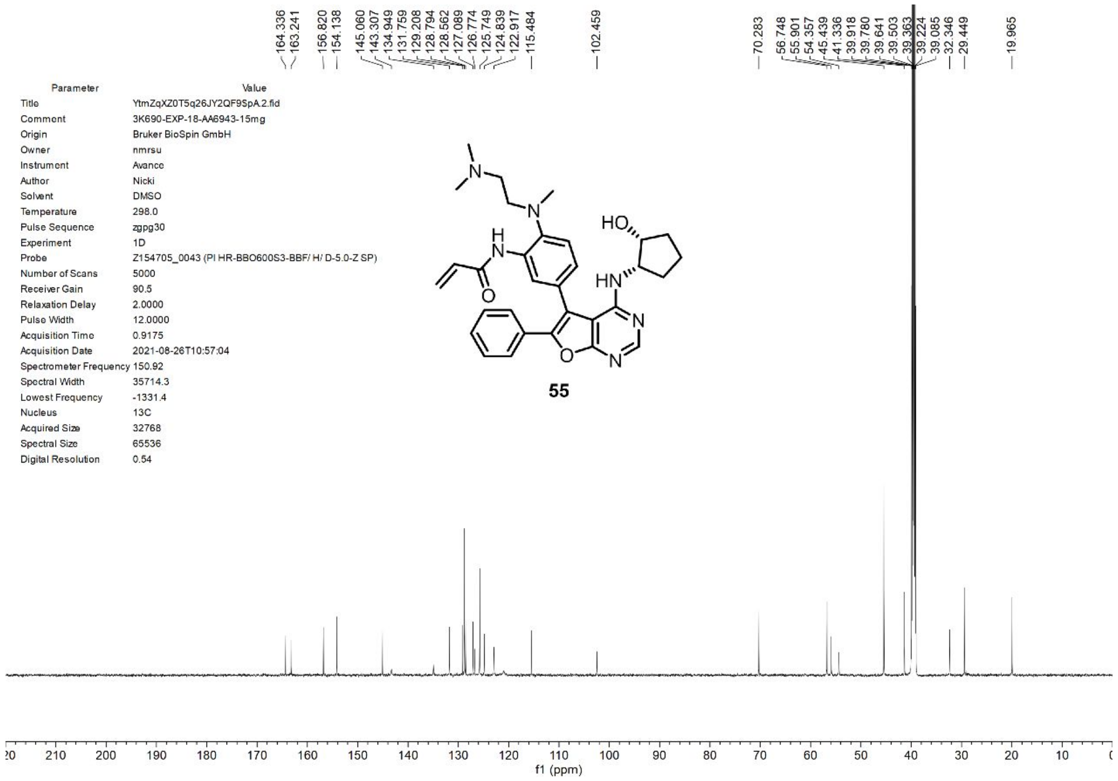
164.336 163.241 156.820 154.138 145.060 143.307 134.949 131.759 129.208 128.794 128.562 127.089 126.774 125.749 124.839 122.917 115.484 102.459 70.283 56.748 55.901 54.357 45.439 41.336 39.918 39.780 39.641 39.503 39.363 39.224 39.085 32.346 29.449 19.965
Parameter Value
Title YtmZqXZ0T5q26JY2QF9SpA.2.fid
Comment 3K690-EXP-18-AA6943-15mg
Origin Bruker BioSpin GmbH
Owner nmrsu
Instrument Avance
Author Nicki
Solvent DMSO
Temperature 298.0
Pulse Sequence zgpg30
Experiment 1D
Probe Z154705_0043 (PI HR-BBO600S3-BBF/ H/ D-5.0-Z SP)
Number of Scans 5000
Receiver Gain 90.5
Relaxation Delay 2.0000
Pulse Width 12.0000
Acquisition Time 0.9175
Acquisition Date 2021-08-26T10:57:04
Spectrometer Frequency 150.92
Spectral Width 35714.3
Lowest Frequency -1331.4
Nucleus 13C
Acquired Size 32768
Spectral Size 65536
Digital Resolution 0.54
HO
HN
N
N
O
55
210 200 190 180 170 160 150 140 130 120 110 100 90 80 70 60 50 40 30 20 10
f1 (ppm)

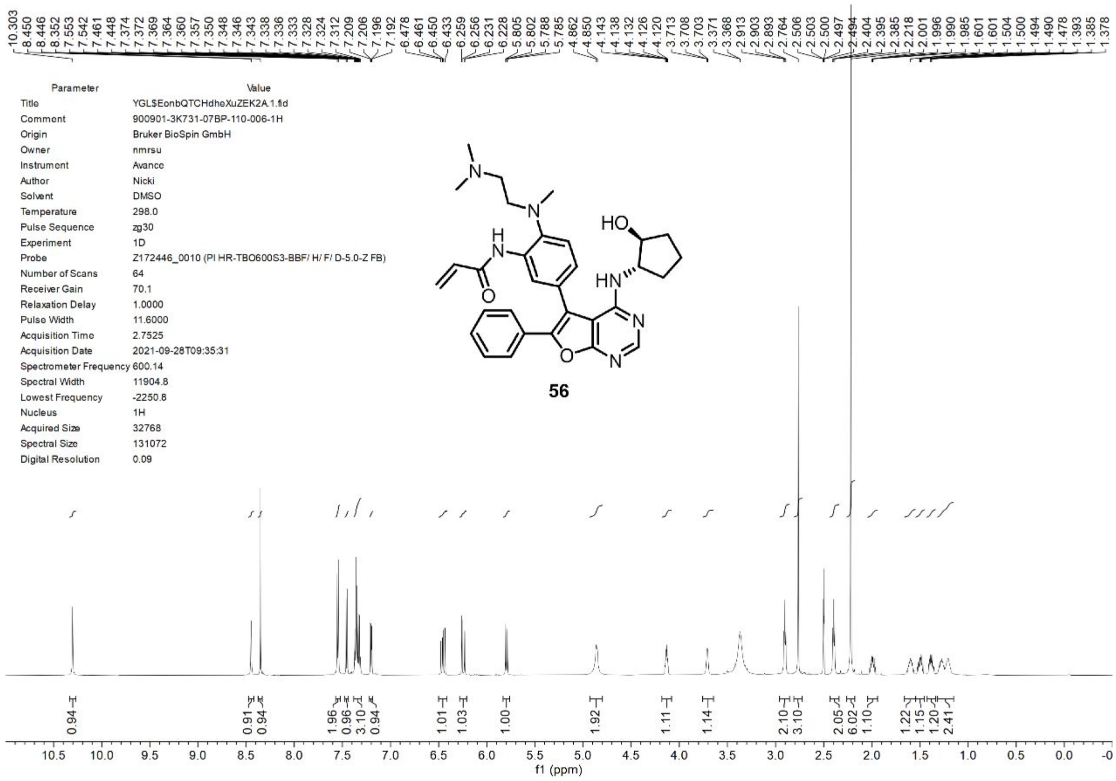
10.303 8.450 8.446 8.352 7.553 7.542 7.461 7.448 7.374 7.372 7.369 7.364 7.360 7.357 7.350 7.348 7.346 7.343 7.338 7.336 7.333 7.328 7.324 7.312 7.209 7.206 7.196 7.192 6.478 6.461 6.450 6.433 6.259 6.256 6.231 6.228 5.805 5.802 5.788 5.785 4.862 4.850 4.143 4.138 4.132 4.126 4.120 3.713 3.708 3.703 3.371 3.368 2.913 2.903 2.893 2.764 2.506 2.503 2.500 2.497 2.494 2.404 2.395 2.385 2.218 2.001 1.996 1.990 1.985 1.601 1.504 1.500 1.494 1.490 1.478 1.393 1.385 1.378
Parameter Value
Title YGL$EonbQTCHdhoXuZEK2A.1.fid
Comment 900901-3K731-07BP-110-006-1H
Origin Bruker BioSpin GmbH
Owner nmrsu
Instrument Avance
Author Nicki
Solvent DMSO
Temperature 298.0
Pulse Sequence zg30
Experiment 1D
Probe Z172446_0010 (PI HR-TBO600S3-BBF/ H/ F/ D-5.0-Z FB)
Number of Scans 64
Receiver Gain 70.1
Relaxation Delay 1.0000
Pulse Width 11.6000
Acquisition Time 2.7525
Acquisition Date 2021-09-28T09:35:31
Spectrometer Frequency 600.14
Spectral Width 11904.8
Lowest Frequency -2250.8
Nucleus 1H
Acquired Size 32768
Spectral Size 131072
Digital Resolution 0.09
HO
HN
N
N
O
N
N
H
O
56
0.94 0.91 0.94 1.96 0.96 3.10 0.94 1.01 1.03 1.00 1.92 1.11 1.14 2.10 3.10 2.05 6.02 1.10 1.22 1.15 1.20 2.41
10.5 10.0 9.5 9.0 8.5 8.0 7.5 7.0 6.5 6.0 5.5 5.0 4.5 4.0 3.5 3.0 2.5 2.0 1.5 1.0 0.5 0.0 -0
f1 (ppm)

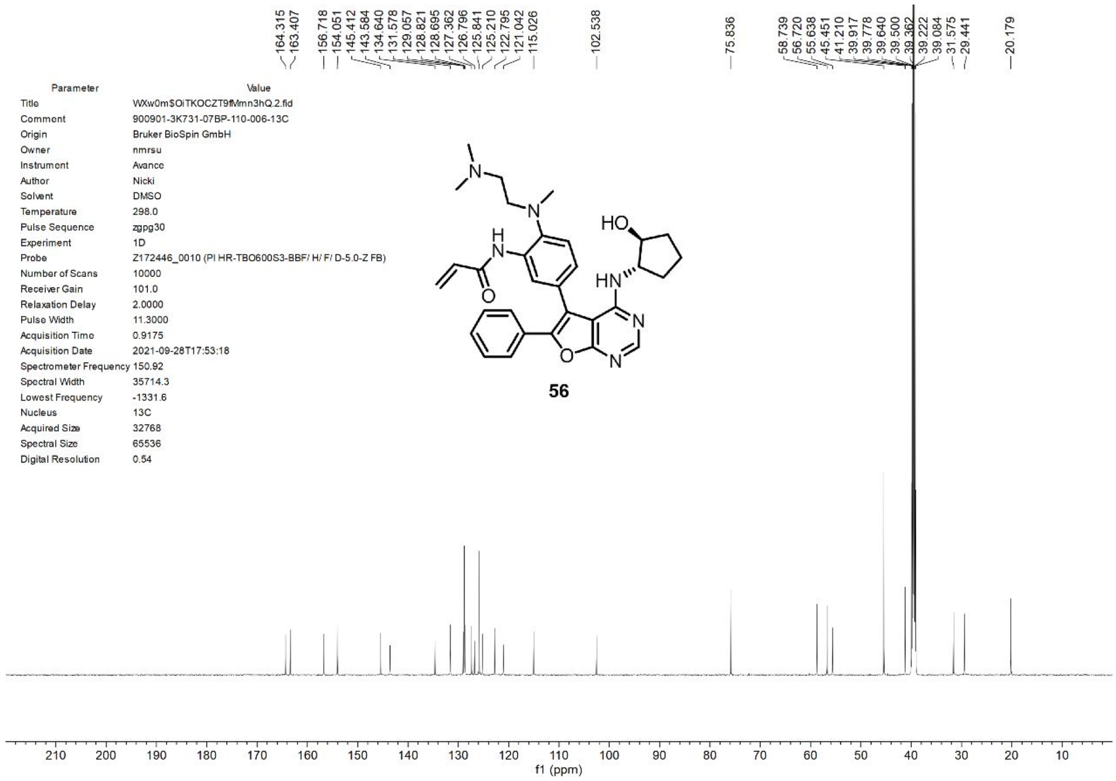
164.315 163.407 156.718 154.051 145.412 143.584 134.640 131.578 129.057 128.821 128.695 127.362 126.796 125.841 125.210 122.795 121.042 115.026 102.538 75.836 58.739 56.720 55.638 45.451 41.210 39.917 39.778 39.640 39.500 39.362 39.222 39.084 31.575 29.441 20.179
Parameter Value
Title WXw0m$OiTKOCZT9fMmn3hQ.2.fid
Comment 900901-3K731-07BP-110-006-13C
Origin Bruker BioSpin GmbH
Owner nmrsu
Instrument Avance
Author Nicki
Solvent DMSO
Temperature 298.0
Pulse Sequence zgpg30
Experiment 1D
Probe Z172446_0010 (PI HR-TBO600S3-BBF/ H/ F/ D-5.0-Z FB)
Number of Scans 10000
Receiver Gain 101.0
Relaxation Delay 2.0000
Pulse Width 11.3000
Acquisition Time 0.9175
Acquisition Date 2021-09-28T17:53:18
Spectrometer Frequency 150.92
Spectral Width 35714.3
Lowest Frequency -1331.6
Nucleus 13C
Acquired Size 32768
Spectral Size 65536
Digital Resolution 0.54
HO
HN
N
N
O
N
N
H
O
56
210 200 190 180 170 160 150 140 130 120 110 100 90 80 70 60 50 40 30 20 10
f1 (ppm)

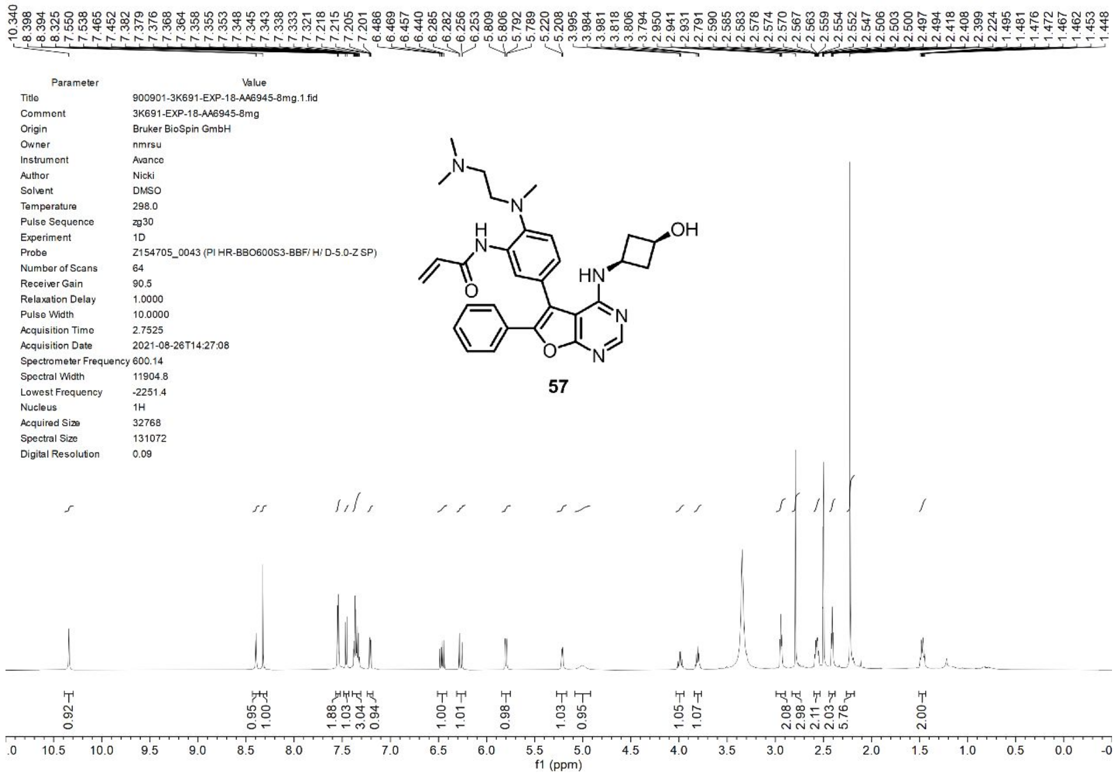

10.340 8.398 8.394 8.325 7.550 7.538 7.465 7.452 7.382 7.379 7.376 7.368 7.364 7.358 7.355 7.353 7.348 7.345 7.343 7.338 7.333 7.321 7.218 7.215 7.205 7.201 6.486 6.469 6.457 6.440 6.285 6.282 6.256 6.253 5.809 5.806 5.792 5.789 5.220 5.208 3.995 3.984 3.981 3.818 3.806 3.794 2.950 2.941 2.931 2.791 2.590 2.585 2.583 2.578 2.574 2.570 2.567 2.563 2.559 2.554 2.552 2.547 2.506 2.503 2.500 2.497 2.494 2.418 2.408 2.399 2.224 1.495 1.481 1.476 1.472 1.467 1.462 1.453 1.448
Parameter Value
Title 900901-3K691-EXP-18-AA6945-8mg.1.fid
Comment 3K691-EXP-18-AA6945-8mg
Origin Bruker BioSpin GmbH
Owner nmrsu
Instrument Avance
Author Nicki
Solvent DMSO
Temperature 298.0
Pulse Sequence zg30
Experiment 1D
Probe Z154705_0043 (PI HR-BBO600S3-BBF/ H/ D-5.0-Z SP)
Number of Scans 64
Receiver Gain 90.5
Relaxation Delay 1.0000
Pulse Width 10.0000
Acquisition Time 2.7525
Acquisition Date 2021-08-26T14:27:08
Spectrometer Frequency 600.14
Spectral Width 11904.8
Lowest Frequency -2251.4
Nucleus 1H
Acquired Size 32768
Spectral Size 131072
Digital Resolution 0.09
57
0.92 0.95 1.00 1.88 1.03 3.04 0.94 1.00 1.01 0.98 1.03 0.95 1.05 1.07 2.08 2.98 2.11 2.03 5.76 2.00
f1 (ppm)

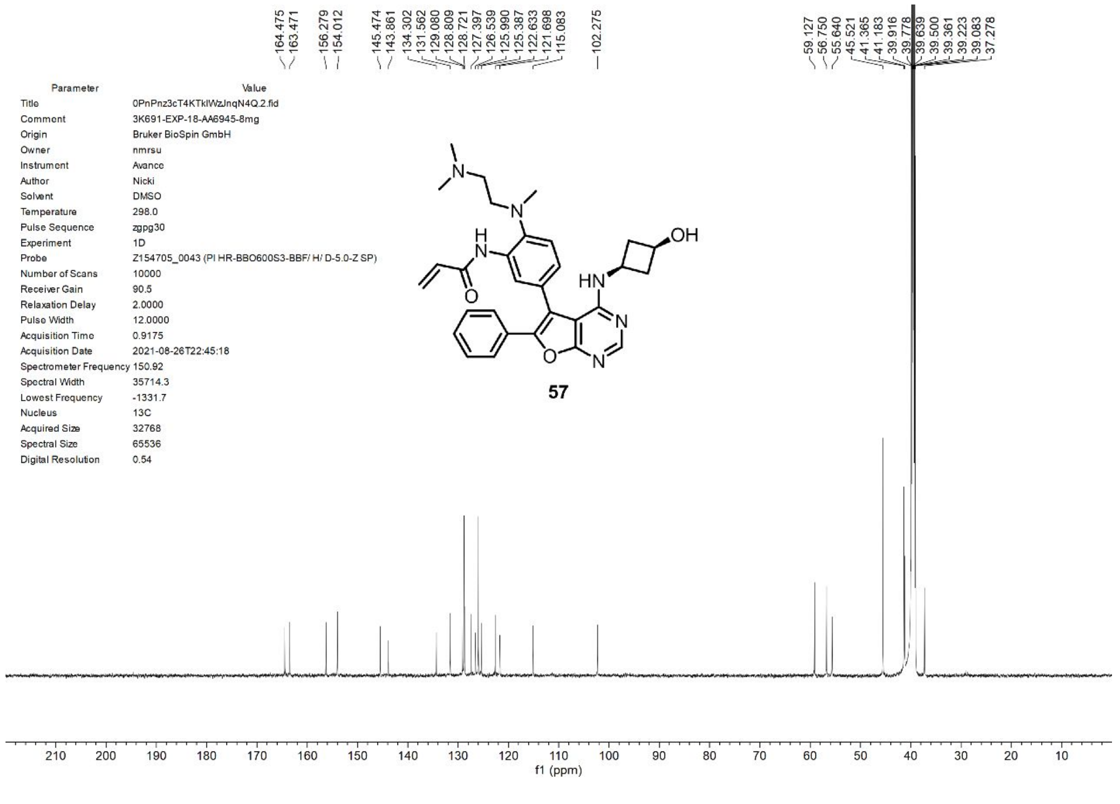

164.475 163.471 156.279 154.012 145.474 143.861 134.302 131.562 129.080 128.809 128.721 127.397 126.539 125.990 125.387 122.633 121.698 115.083 102.275
59.127 56.750 55.640 45.521 41.365 41.183 39.916 39.778 39.639 39.500 39.361 39.223 39.083 37.278
Parameter Value
Title 0PnPnz3cT4KTklWzJnqN4Q.2.fid
Comment 3K691-EXP-18-AA6945-8mg
Origin Bruker BioSpin GmbH
Owner nmrsu
Instrument Avance
Author Nicki
Solvent DMSO
Temperature 298.0
Pulse Sequence zgpg30
Experiment 1D
Probe Z154705_0043 (PI HR-BBO600S3-BBF/ H/ D-5.0-Z SP)
Number of Scans 10000
Receiver Gain 90.5
Relaxation Delay 2.0000
Pulse Width 12.0000
Acquisition Time 0.9175
Acquisition Date 2021-08-26T22:45:18
Spectrometer Frequency 150.92
Spectral Width 35714.3
Lowest Frequency -1331.7
Nucleus 13C
Acquired Size 32768
Spectral Size 65536
Digital Resolution 0.54
57
f1 (ppm)

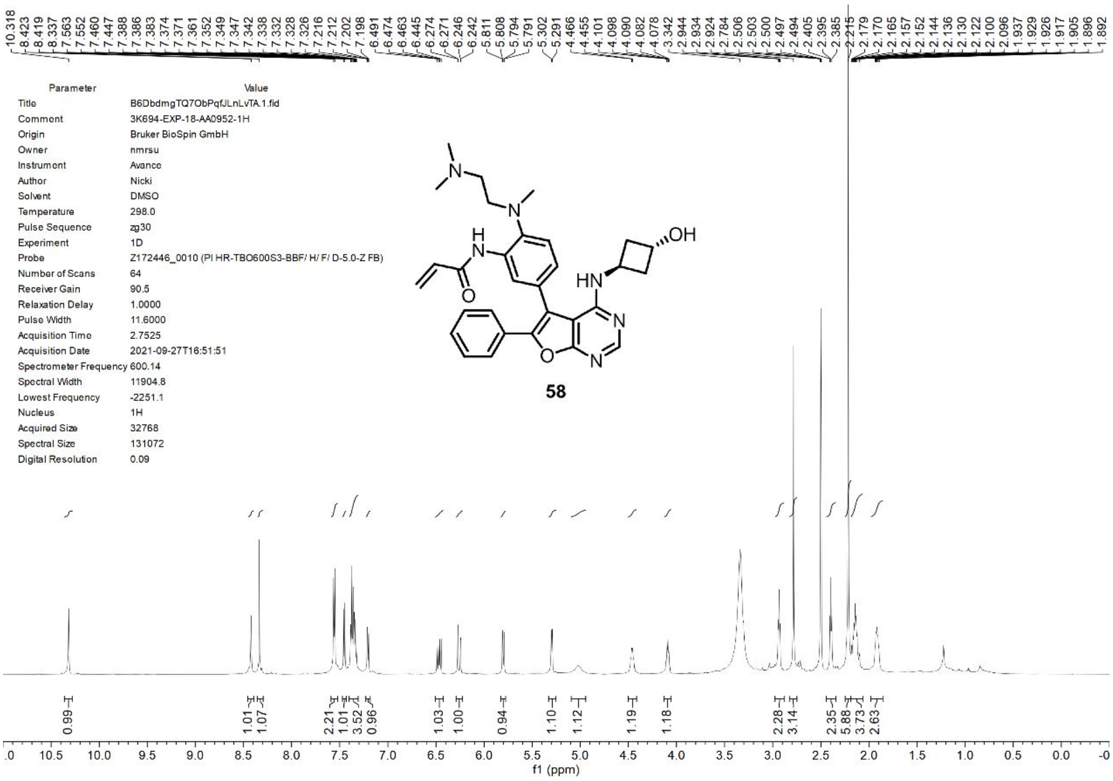
10.318 8.423 8.419 8.337 7.563 7.552 7.460 7.447 7.388 7.386 7.383 7.374 7.371 7.361 7.352 7.349 7.347 7.342 7.338 7.332 7.328 7.326 7.216 7.212 7.202 7.198 6.491 6.474 6.463 6.445 6.274 6.271 6.246 6.242 5.811 5.808 5.794 5.791 5.302 5.291 4.466 4.455 4.101 4.098 4.090 4.082 4.078 3.342 2.944 2.934 2.924 2.784 2.506 2.503 2.500 2.497 2.494 2.405 2.395 2.385 2.215 2.179 2.170 2.165 2.157 2.152 2.144 2.136 2.122 2.100 2.096 1.937 1.929 1.926 1.917 1.905 1.896 1.892
Parameter Value
Title B6DbdmgTQ7ObPqfJLnLvTA.1.fid
Comment 3K694-EXP-18-AA0952-1H
Origin Bruker BioSpin GmbH
Owner nmrsu
Instrument Avance
Author Nicki
Solvent DMSO
Temperature 298.0
Pulse Sequence zg30
Experiment 1D
Probe Z172446_0010 (PI HR-TBO600S3-BBF/ H/ F/ D-5.0-Z FB)
Number of Scans 64
Receiver Gain 90.5
Relaxation Delay 1.0000
Pulse Width 11.6000
Acquisition Time 2.7525
Acquisition Date 2021-09-27T16:51:51
Spectrometer Frequency 600.14
Spectral Width 11904.8
Lowest Frequency -2251.1
Nucleus 1H
Acquired Size 32768
Spectral Size 131072
Digital Resolution 0.09
N
N
H
N
O
HN
OH
N
N
O
58
0.99 1.01 1.07 2.21 1.01 3.52 0.96 1.03 1.00 0.94 1.10 1.12 1.19 1.18 2.28 3.14 2.35 5.88 3.73 2.63
10.5 10.0 9.5 9.0 8.5 8.0 7.5 7.0 6.5 6.0 5.5 5.0 4.5 4.0 3.5 3.0 2.5 2.0 1.5 1.0 0.5 0.0
f1 (ppm)

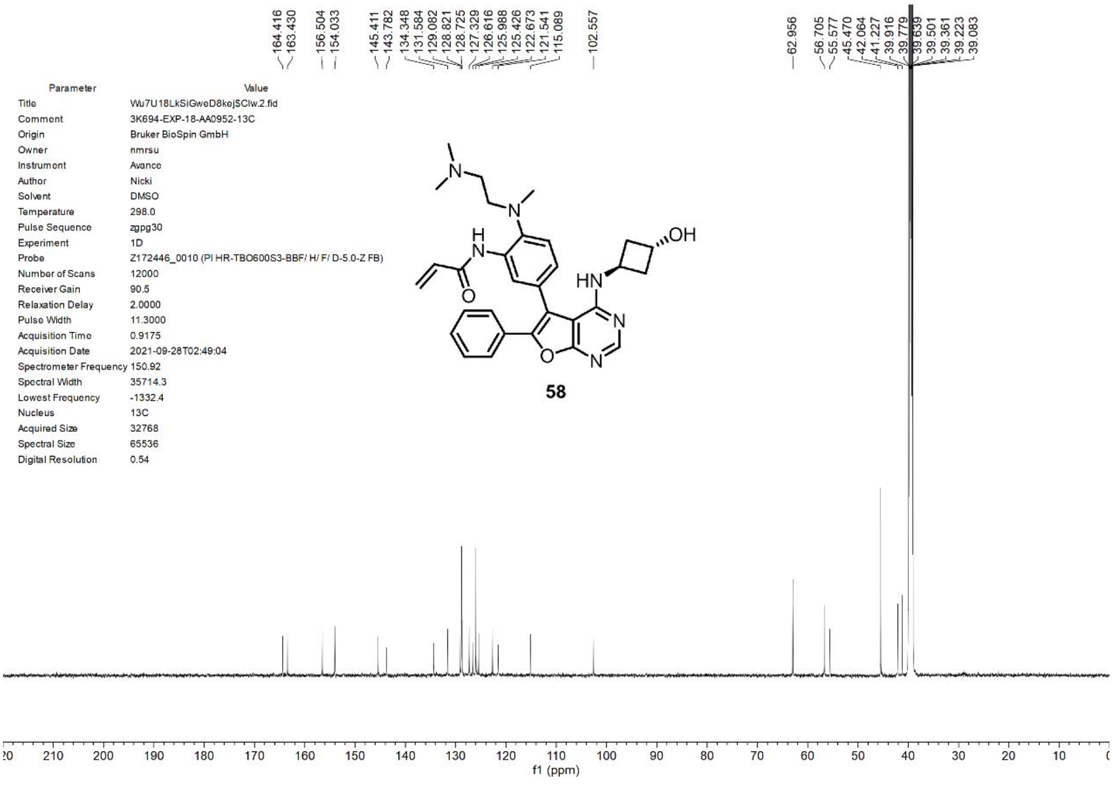
164.416 163.430 156.504 154.033 145.411 143.782 134.348 131.584 129.082 128.821 128.725 127.329 126.616 125.988 125.426 122.673 121.541 115.089 102.557 62.956 56.705 55.577 45.470 42.064 41.227 39.916 39.779 39.639 39.501 39.361 39.223 39.083
Parameter Value
Title Wu7U18LkSiGweD8kejSClw.2.fid
Comment 3K694-EXP-18-AA0952-13C
Origin Bruker BioSpin GmbH
Owner nmrsu
Instrument Avance
Author Nicki
Solvent DMSO
Temperature 298.0
Pulse Sequence zgpg30
Experiment 1D
Probe Z172446_0010 (PI HR-TBO600S3-BBF/ H/ F/ D-5.0-Z FB)
Number of Scans 12000
Receiver Gain 90.5
Relaxation Delay 2.0000
Pulse Width 11.3000
Acquisition Time 0.9175
Acquisition Date 2021-09-28T02:49:04
Spectrometer Frequency 150.92
Spectral Width 35714.3
Lowest Frequency -1332.4
Nucleus 13C
Acquired Size 32768
Spectral Size 65536
Digital Resolution 0.54
N
N
H
N
O
HN
OH
N
N
O
58
210 200 190 180 170 160 150 140 130 120 110 100 90 80 70 60 50 40 30 20 10
f1 (ppm)

4. Kinase profiling data for 52

Table S1. Kinase profiling for **52** against a 468-kinase panel (containing 403 non-mutant kinases) at a concentration of 1 μM using the KINOMEscan^{TM} technology.

DiscoveRx Gene Symbol	% Control
AAK1	100
ABL1(E255K)-phosphorylated	100
ABL1(F317I)-nonphosphorylated	84
ABL1(F317I)-phosphorylated	76
ABL1(F317L)-nonphosphorylated	100
ABL1(F317L)-phosphorylated	67
ABL1(H396P)-nonphosphorylated	79
ABL1(H396P)-phosphorylated	100
ABL1(M351T)-phosphorylated	100
ABL1(Q252H)-nonphosphorylated	86
ABL1(Q252H)-phosphorylated	100
ABL1(T315I)-nonphosphorylated	96
ABL1(T315I)-phosphorylated	100
ABL1(Y253F)-phosphorylated	100
ABL1-nonphosphorylated	85
ABL1-phosphorylated	92
ABL2	100
ACVR1	100
ACVR1B	100
ACVR2A	100
ACVR2B	100
ACVRL1	72
ADCK3	100
ADCK4	100
AKT1	100
AKT2	100
AKT3	100
ALK	51
ALK(C1156Y)	47
ALK(L1196M)	76
AMPK-alpha1	91
AMPK-alpha2	98
ANKK1	100
ARK5	100
ASK1	100
ASK2	84
AURKA	91

AURKB	81
DiscoveRx Gene Symbol	% Control
AURKC	100
AXL	91
BIKE	100
BLK	0.9
BMPR1A	100
BMPR1B	93
BMPR2	96
BMX	65
BRAF	94
BRAF(V600E)	100
BRK	100
BRSK1	100
BRSK2	96
BTK	0
BUB1	57
CAMK1	97
CAMK1B	100
CAMK1D	100
CAMK1G	100
CAMK2A	100
CAMK2B	100
CAMK2D	100
CAMK2G	100
CAMK4	99
CAMKK1	100
CAMKK2	100
CASK	100
CDC2L1	100
CDC2L2	100
CDC2L5	100
CDK11	100
CDK2	100
CDK3	100
CDK4	99
CDK4-cyclinD1	66
CDK4-cyclinD3	100

CDK5	100
CDK7	80
DiscoveRx Gene Symbol	% Control
CDK8	99
CDK9	100
CDKL1	97
CDKL2	100
CDKL3	100
CDKL5	52
CHEK1	100
CHEK2	98
CIT	100
CLK1	93
CLK2	100
CLK3	100
CLK4	100
CSF1R	100
CSF1R-autoinhibited	87
CSK	100
CSNK1A1	100
CSNK1A1L	98
CSNK1D	100
CSNK1E	100
CSNK1G1	100
CSNK1G2	100
CSNK1G3	100
CSNK2A1	75
CSNK2A2	92
CTK	94
DAPK1	100
DAPK2	100
DAPK3	100
DCAMKL1	77
DCAMKL2	100
DCAMKL3	100
DDR1	97
DDR2	100
DLK	64
DMPK	100
DMPK2	99
DRAK1	100

DRAK2	95
DYRK1A	93
DiscoveRx Gene Symbol	% Control
DYRK1B	94
DYRK2	73
EGFR	1.7
EGFR(E746-A750del)	1.1
EGFR(G719C)	8.6
EGFR(G719S)	5.3
EGFR(L747-E749del, A750P)	6.4
EGFR(L747-S752del, P753S)	0
EGFR(L747-T751del,Sins)	0
EGFR(L858R)	0.55
EGFR(L858R,T790M)	1.9
EGFR(L861Q)	9.3
EGFR(S752-I759del)	94
EGFR(T790M)	0.45
EIF2AK1	100
EPHA1	99
EPHA2	100
EPHA3	100
EPHA4	98
EPHA5	100
EPHA6	98
EPHA7	100
EPHA8	100
EPHB1	100
EPHB2	100
EPHB3	98
EPHB4	100
EPHB6	100
ERBB2	0
ERBB3	48
ERBB4	0
ERK1	100
ERK2	100
ERK3	100
ERK4	100
ERK5	100
ERK8	100
ERN1	93

FAK	100
FER	100
DiscoveRx Gene Symbol	% Control
FES	100
FGFR1	100
FGFR2	100
FGFR3	100
FGFR3(G697C)	100
FGFR4	100
FGR	93
FLT1	100
FLT3	65
FLT3(D835H)	71
FLT3(D835V)	69
FLT3(D835Y)	91
FLT3(ITD)	100
FLT3(ITD,D835V)	60
FLT3(ITD,F691L)	93
FLT3(K663Q)	83
FLT3(N841I)	94
FLT3(R834Q)	72
FLT3-autoinhibited	93
FLT4	100
FRK	100
FYN	100
GAK	99
GCN2(Kin.Dom.2,S808G)	99
GRK1	90
GRK2	100
GRK3	98
GRK4	100
GRK7	81
GSK3A	98
GSK3B	91
HASPIN	84
HCK	90
HIPK1	100
HIPK2	81
HIPK3	89
HIPK4	100
HPK1	100

HUNK	100
ICK	100
DiscoveRx Gene Symbol	% Control
IGF1R	100
IKK-alpha	99
IKK-beta	100
IKK-epsilon	97
INSR	89
INSRR	100
IRAK1	100
IRAK3	57
IRAK4	95
ITK	57
JAK1(JH1domain-catalytic)	100
JAK1(JH2domain-pseudokinase)	100
JAK2(JH1domain-catalytic)	100
JAK3(JH1domain-catalytic)	0.4
JNK1	100
JNK2	100
JNK3	100
KIT	100
KIT(A829P)	94
KIT(D816H)	82
KIT(D816V)	100
KIT(L576P)	87
KIT(V559D)	99
KIT(V559D,T670I)	100
KIT(V559D,V654A)	100
KIT-autoinhibited	95
LATS1	99
LATS2	100
LCK	92
LIMK1	100
LIMK2	100
LKB1	100
LOK	83
LRRK2	90
LRRK2(G2019S)	80
LTK	82
LYN	88
LZK	100

MAK	96
MAP3K1	48
DiscoveRx Gene Symbol	% Control
MAP3K15	100
MAP3K2	100
MAP3K3	96
MAP3K4	100
MAP4K2	97
MAP4K3	100
MAP4K4	99
MAP4K5	100
MAPKAPK2	80
MAPKAPK5	92
MARK1	87
MARK2	100
MARK3	78
MARK4	100
MAST1	91
MEK1	97
MEK2	100
MEK3	87
MEK4	99
MEK5	100
MEK6	92
MELK	84
MERTK	100
MET	100
MET(M1250T)	97
MET(Y1235D)	100
MINK	96
MKK7	99
MKNK1	85
MKNK2	100
MLCK	100
MLK1	100
MLK2	100
MLK3	100
MRCKA	100
MRCKB	100
MST1	99
MST1R	100

MST2	100
MST3	100
DiscoveRx Gene Symbol	% Control
MST4	93
MTOR	97
MUSK	100
MYLK	100
MYLK2	100
MYLK4	100
MYO3A	91
MYO3B	95
NDR1	100
NDR2	100
NEK1	90
NEK10	100
NEK11	100
NEK2	98
NEK3	100
NEK4	100
NEK5	100
NEK6	100
NEK7	98
NEK9	97
NIK	97
NIM1	100
NLK	100
OSR1	100
p38-alpha	100
p38-beta	100
p38-delta	100
p38-gamma	100
PAK1	100
PAK2	83
PAK3	84
PAK4	100
PAK6	98
PAK7	100
PCTK1	100
PCTK2	100
PCTK3	100
PDGFRA	81

PDGFRB	82
PDPK1	97
DiscoveRx Gene Symbol	% Control
PFCDPK1(P.falciparum)	82
PFPK5(P.falciparum)	100
PFTAIRE2	98
PFTK1	100
PHKG1	97
PHKG2	100
PIK3C2B	100
PIK3C2G	86
PIK3CA	100
PIK3CA(C420R)	97
PIK3CA(E542K)	98
PIK3CA(E545A)	100
PIK3CA(E545K)	95
PIK3CA(H1047L)	100
PIK3CA(H1047Y)	89
PIK3CA(I800L)	89
PIK3CA(M1043I)	84
PIK3CA(Q546K)	98
PIK3CB	100
PIK3CD	78
PIK3CG	100
PIK4CB	100
PIKFYVE	92
PIM1	99
PIM2	100
PIM3	94
PIP5K1A	100
PIP5K1C	84
PIP5K2B	100
PIP5K2C	88
PKAC-alpha	100
PKAC-beta	100
PKMYT1	100
PKN1	100
PKN2	97
PKNB(M.tuberculosis)	100
PLK1	80
PLK2	84

PLK3	88
PLK4	74
DiscoveRx Gene Symbol	% Control
PRKCD	100
PRKCE	94
PRKCH	100
PRKCI	100
PRKCQ	100
PRKD1	99
PRKD2	100
PRKD3	83
PRKG1	99
PRKG2	100
PRKR	79
PRKX	100
PRP4	100
PYK2	100
QSK	100
RAF1	100
RET	100
RET(M918T)	100
RET(V804L)	100
RET(V804M)	97
RIOK1	100
RIOK2	100
RIOK3	100
RIPK1	100
RIPK2	100
RIPK4	92
RIPK5	94
ROCK1	92
ROCK2	83
ROS1	51
RPS6KA4(Kin.Dom.1-N-terminal)	100
RPS6KA4(Kin.Dom.2-C-terminal)	97
RPS6KA5(Kin.Dom.1-N-terminal)	100
RPS6KA5(Kin.Dom.2-C-terminal)	100
RSK1(Kin.Dom.1-N-terminal)	100
RSK1(Kin.Dom.2-C-terminal)	93
RSK2(Kin.Dom.1-N-terminal)	98
RSK2(Kin.Dom.2-C-terminal)	89

RSK3(Kin.Dom.1-N-terminal)	100
RSK3(Kin.Dom.2-C-terminal)	100
DiscoveRx Gene Symbol	% Control
RSK4(Kin.Dom.1-N-terminal)	85
RSK4(Kin.Dom.2-C-terminal)	100
S6K1	96
SBK1	100
SGK	100
SgK110	100
SGK2	100
SGK3	99
SIK	100
SIK2	84
SLK	96
SNARK	100
SNRK	100
SRC	100
SRMS	100
SRPK1	87
SRPK2	93
SRPK3	82
STK16	88
STK33	93
STK35	100
STK36	100
STK39	100
SYK	98
TAK1	97
TAOK1	100
TAOK2	100
TAOK3	100
TBK1	100
TEC	14
TESK1	100
TGFBR1	100
TGFBR2	100
TIE1	100
TIE2	100

TLK1	96
TLK2	88
DiscoveRx Gene Symbol	% Control
TNIK	100
TNK1	100
TNK2	100
TNNI3K	93
TRKA	38
TRKB	89
TRKC	89
TRPM6	86
TSSK1B	100
TSSK3	100
TTK	100
TXK	9.9
TYK2(JH1domain-catalytic)	100
TYK2(JH2domain-pseudokinase)	100
TYRO3	100
ULK1	75
ULK2	94
ULK3	98
VEGFR2	100
VPS34	100
VRK2	93
WEE1	99
WEE2	96
WNK1	82
WNK2	100
WNK3	98
WNK4	100
YANK1	100
YANK2	100
YANK3	100
YES	100
YSK1	90
YSK4	95
ZAK	100
ZAP70	82

Table S2. *S*-score results for **52**.

Compound	Selectivity Score Type	Number of Hits	Number of Non-Mutant Kinases	Screening Concentration (nM)	Selectivity Score
**52**	*S*(35)	8	403	1000	0.020
**52**	*S*(10)	7	403	1000	0.017
**52**	*S*(1)	5	403	1000	0.012

5. HPLC trace of 49 and 52

Injection Summary Report

SAMPLE INFORMATION

Sample Name:	05BP-051-158-50	Acquired By:	System
Sample Type:	Unknown	Sample Set Name:	20160715 S
Vial:	1:F,8	Acq. Method Set:	Purity 20071224 37min
Injection #:	1	Processing Method:	C18
Injection Volume:	10.00 ul	Channel Name:	PDA Ch1 254nm@4.8nm
Run Time:	37.0 Minutes	Proc. Chnl. Descr.:	PDA Ch1 254nm@4.8nm
Date Acquired:	2016/7/15 AM 05:55:47 CST		
Date Processed:	2016/7/21 PM 02:43:15 CST		

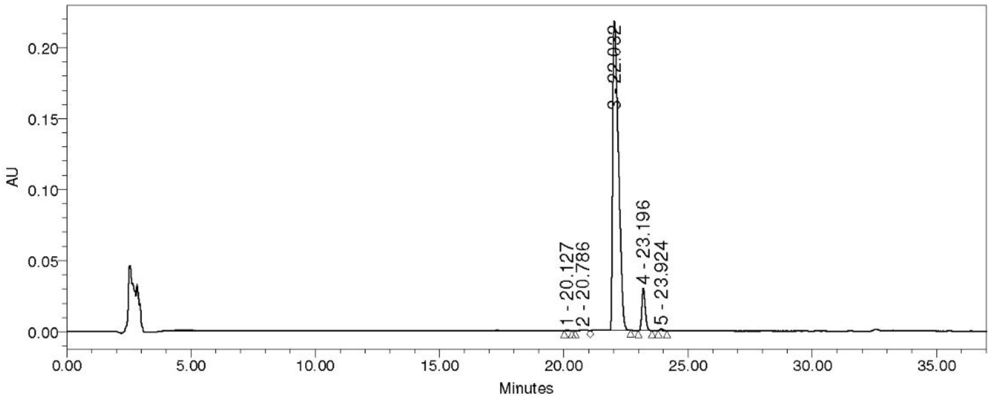

Channel: PDA Ch1 254nm@4.8nm; Processed Channel: PDA Ch1 254nm@4.8nm; Result Id: 4045; Processing Method: C18

	Peak Name	RT	Height	Area (µV*sec)	% Area
1	1	20.127	300	2821	0.07
2	2	20.786	666	10945	0.28
3	3	22.032	217899	3567805	91.88
4	4	23.196	29688	289937	7.47
5	5	23.924	1349	11632	0.30

Reported by User: System
Report Method: Injection Summary Report
Report Method ID: 3302
Page: 1 of 1

Project Name: Waters_2
Date Printed:
2016/7/21
02:43:42 Asia/Taipei

Figure S3. HPLC trace of **49**.

D-2000: Samples Series: 0386 Report Name: modified System: Sys 1

Hitachi D-2000 Elite HPLC System Manager Report

Analyzed Date and Time: 2018/06/27 10:20
Reported Date and Time: 2018/06/27 03:29
Data Path: C:\Win32app\D2000HSM\samples\DATA\0386\
Processing Method: Purity 2007/12/24 37min

Sample Name: EXP-18-AA0278-50 Vial Number: 166
Injection from this vial: 1 of 1 Volume: 20.0 ul
Sample Description:

Chrom Type: Fixed WL Chromatogram, 254 nm

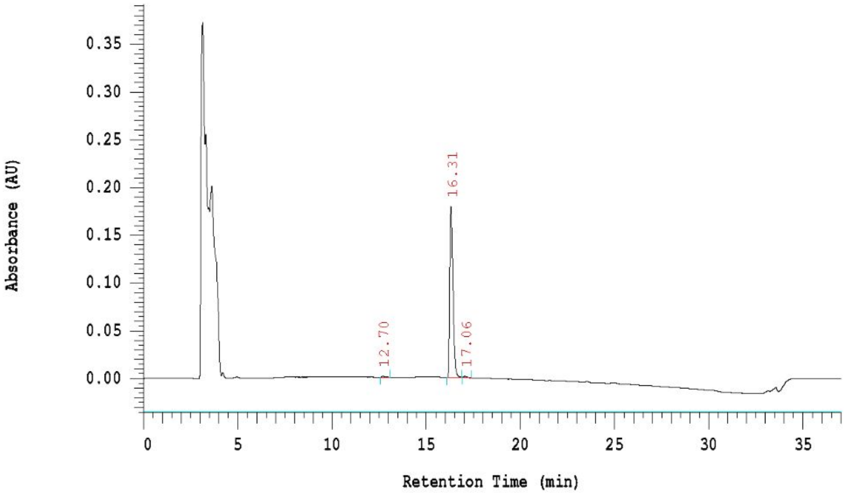

Processing Method: Purity 2007/12/24 37min
Column Type: Column Method Developer: Bob
Method Description:

Peak Quantitation: AREA
Calculation Method: AREA%

No.	RT	Area	Height	Conc 1
1	12.70	4768	555	0.451
2	16.31	1045592	89330	98.959
3	17.06	6227	631	0.589
		1056587	90516	100.000

Peak rejection level: 0

Page Indicator: 1 / 3

Figure S4. HPLC trace of **52**.

6. References

(1) Coumar, M. S.; Tsai, M.-T.; Chu, C.-Y.; Uang, B.-J.; Lin, W.-H.; Chang, C.-Y.; Chang, T.-Y.; Leou, J.-S.; Teng, C.-H.; Wu, J.-S.; Fang, M.-Y.; Chen, C.-H.; Hsu, J. T.-A.; Wu, S.-Y.; Chao, Y.-S.; Hsieh, H.-P. Identification, SAR Studies, and X-Ray Co-Crystallographic Analysis of a Novel Furanopyrimidine Aurora Kinase A Inhibitor. *ChemMedChem* **2010**, *5*, 255–267.

(2) Lin, S.-Y.; Chang Hsu, Y.; Peng, Y.-H.; Ke, Y.-Y.; Lin, W.-H.; Sun, H.-Y.; Shiao, H.-Y.; Kuo, F.-M.; Chen, P.-Y.; Lien, T.-W.; Chen, C.-H.; Chu, C.-Y.; Wang, S.-Y.; Yeh, K.-C.; Chen, C.-P.; Hsu, T.-A.; Wu, S.-Y.; Yeh, T.-K.; Chen, C.-T.; Hsieh, H.-P. Discovery of a Furanopyrimidine-Based Epidermal Growth Factor Receptor Inhibitor (DBPR112) as a Clinical Candidate for the Treatment of Non-Small Cell Lung Cancer. *J. Med. Chem.* **2019**, *62*, 10108–10123.
